# Pd-Catalyzed Organometallic-Free Homologation of Arylboronic Acids Enabled by Chemoselective Transmetalation

**DOI:** 10.1021/acscatal.3c00921

**Published:** 2023-05-09

**Authors:** Kane A. C. Bastick, Allan J. B. Watson

**Affiliations:** EaStCHEM, School of Chemistry, University of St Andrews, North Haugh, Saint Andrews, Fife KY16 9ST, U.K.

**Keywords:** boron, catalysis, chemoselectivity, cross-coupling, homologation, palladium

## Abstract

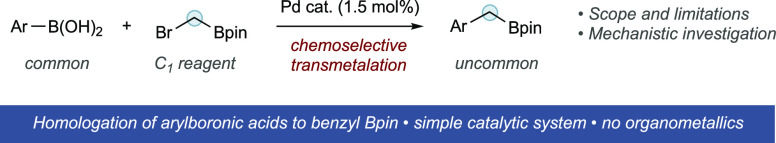

A Pd-catalyzed homologation of arylboronic acids is reported. Halomethylboronic acid pinacol esters (Bpin) undergo a remarkably facile, yet rare, oxidative addition enabled by an α-boryl effect. Simultaneous chemoselective transmetalation allows use of these metalloid reagents for formal C_1_ insertion to deliver benzyl Bpin products without the requirement for stoichiometric organometallic reagents. The utility of the process is demonstrated by stepwise C(sp^3^)–C(sp^2^) cross-coupling of the boronic ester products into diarylmethane pharmacophores and electrophile/nucleophile chemoselective cross-coupling. Control experiments that demonstrate the reactivity enhancement provided by the α-boryl effect are provided, along with a description of the limitations of the formal homologation process.

Organoboron compounds are valuable reagents that provide immediate access to a variety of synthetic transformations for C–C and C–X bond formation.^1−3^ While their widespread use is historically framed within transition metal-catalyzed cross-coupling reactions (e.g., Suzuki–Miyaura^4−6^ and Chan–Lam^7−11^), modern organoboron chemistry provides broad and bespoke reactivity as reagents, catalysts, additives, materials, and drug candidates.^1^ As such, the installation of boron functional groups continues to be a very active field of methodological development.

Classical approaches to boron installation, such as the use of organometallics^1,6,12−14^ and hydroboration,^1,6,15−18^ have been supplemented by contemporary methodologies including photoredox catalysis^19−22^ and C–H activation.^23−29^ A particularly powerful approach to the formation of complex organoboron compounds has been enabled through single carbon homologations.^30−32^ Pioneered by Matteson,^33^ this approach uses a carbenoid as the key reagent to induce a stereospecific 1,2-metallate rearrangement (Scheme 1a).

**Scheme 1:**
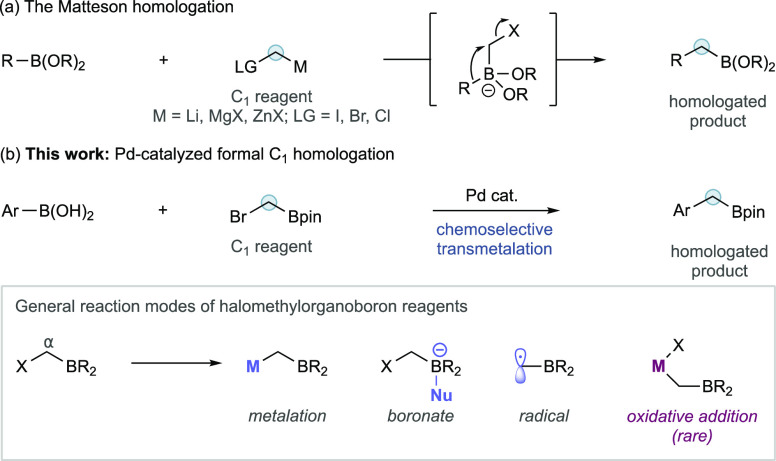
(a) General Representation of the Matteson Homologation; (b) This Work: Arylboronic Acid Homologation Using Halomethyl Bpin Enabled by Chemoselective Transmetalation

This general strategy has seen significant development in elegant work by Aggarwal, leading to powerful platforms for iterative synthesis.^34−38^ The first catalytic asymmetric approach to the 1,2-metallate rearrangement of lithium boronates was recently reported by Jacobsen.^39^

All existing metalate rearrangements require the use of stoichiometric organolithium, -magnesium, or -zinc reagents;^30−32,36^ however, it should be noted that metal-free processes based on diazoalkanes^40−43^ and carbenes^44^ have also been developed. Here, we show an alternative conceptual approach to organoboron homologation using chemoselective Pd-catalyzed cross-coupling. This method does not require stoichiometric organometallics and instead relies upon a relatively rare oxidative addition to halomethylorganoboron reagents combined with chemoselective transmetalation (Scheme 1b).

Halomethylorganoboron metalloids have been shown to display several different reactivity profiles, including metalation, boronate formation, and the formation of α-boryl radicals; however, examples of oxidative addition to this reagent class remain rare and are principally achieved using Ni catalysis. For example, Fu demonstrated stereoconvergent cross-coupling of racemic α-chloroboronic acid esters with organozinc reagents,^45,46^ while Martin first showed cross-electrophile coupling^47−49^ and alkene difunctionalization using α-bromoboronic acid esters.^50^

Within Pd catalysis, Gevorgyan has developed Heck reactions based on a single-electron transfer (SET) manifold.^51,52^ To our knowledge, the only example of a direct oxidative addition of Pd(0) to a halomethylboronic acid ester was shown by Falck.^53^ This process employed halomethylboronic acid esters with aryl and vinyl stannanes, exploiting the more rapid transmetalation of the organostannane, to deliver the homologated organoboron product.

We hypothesized that a formal C_1_ homologation of boronic acids could be achieved using a halomethylorganoboron reagent as the surrogate carbenoid. Selective engagement of this reagent as the electrophilic component in a chemoselective Suzuki–Miyaura cross-coupling with a boronic acid would deliver a benzylic Bpin product without the use of organometallic reagents; however, it should be noted that the halomethylorganoboron reagents do require organometallic reagents in their preparation.^54^ This approach would be contingent upon several key control elements: (i) chemoselective transmetalation of the arylboronic acid over the halomethyl Bpin, (ii) inhibition of organoboron group transesterification (speciation),^55−61^ (iii) inhibition of product transmetalation, which would lead to oligo- or polymerization, and (iv) inhibition of Bpin hydrolysis (starting material and product).

Control of Bpin hydrolysis was particularly important as the corresponding boronic acids are unstable and prone to rapid protodeboronation. Indeed, few benzylic boronic acids or esters are commercially available, especially compared to the equivalent aryl reagents.^62^ Enabling the formal homologation of arylboronic acids to benzyl Bpin would provide straightforward access to this underrepresented molecular space.

As a first demonstration of this concept, we established a benchmark system based on the Pd-catalyzed homologation of arylboronic acid **1** with the methylene donor **2-Br** to deliver benzylic Bpin product **3** (Table 1). Optimization established standard conditions that delivered **3** in good yield with low loadings of a simple Pd catalyst and the mass balance comprising the transesterification adduct **4** (entry 1). **2-Br** was completely consumed, which suggested competing hydrolysis to the boronic acid and protodeboronation. This was supported by control experiments (see ESI). Selected key parameters are indicated in Table 1. The surprising reactivity of **2-Br** was evident from the outset—the reaction required low loadings of Pd(PPh_3_)_4_ and attempts to use any more electron-rich phosphine system led to lower yields and increased speciation (e.g., entries 3–4; see ESI for a full ligand screen). Changing **2-Br** for the iodo- and chloro-analogues (**2-I** and **2-Cl**) delivered moderate yields with greater pinacol speciation to byproduct **4**, suggesting that **2-Br** had an optimal balance of reactivity and stability to allow effective cross-coupling (entries 5 and 6). As part of efforts to avoid speciation issues, we attempted to use the pinacol ester of **1** (*p*-tol–Bpin, **4**); however, interestingly, this was ineffective (entry 7), indicating that **4** does not undergo effective transmetalation under these conditions.^63^ Similarly, control experiments (see ESI) indicated that benzylic Bpin product **3** was unreactive toward transmetalation, eliminating possible oligo/polymerization. These data indicated that a chemoselective transmetalation process was operative, where boronic acid **1** was reactive to transmetalation and Bpins **2-Br**, **3**, and **4** were not. While this represents one of the key selectivity elements in the overall process, a consequence of this selectivity is that uncontrolled speciation (conversion of **1**–**4**) during the reaction effectively leads to reaction shutdown. It was therefore necessary to control speciation, which could be realized by tuning the nature and stoichiometry of the base and stoichiometry of H_2_O.^55−61^ This was effectively achieved using 3 equiv of K_3_PO_4_ with 10 equiv of H_2_O (entry 1). Subtle changes, for example, to K_2_CO_3_ led to poorer conversion to desired product **3**, with an increase in the transesterification product (entry 8). Other bases, water concentration, and reaction temperature had similar negative effects (see ESI).

**Table 1:**
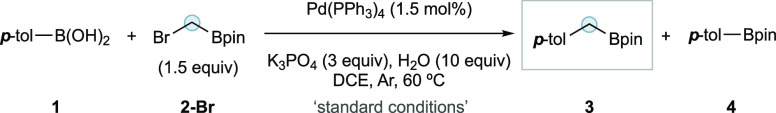
Reaction Development

entry	deviation from standard conditions	**3**/**4**(%)^a^
1	none	90 (88)^b^/10
2	Pd_2_(dba)_3_ (1.5 mol %) + PPh_3_ (6 mol %)	45/55
3	Pd(dppf)Cl_2_ (1.5 mol %)	8/88
4	Pd(OAc)_2_ (1.5 mol %) + SPhos (3 mol %)	8/58
5	iodomethyl Bpin (**2-I**)	66/34
6	chloromethyl Bpin (**2-Cl**)	49/51
7	*p*-tolBpin (**4**) instead of **1**	14/84
8	K_2_CO_3_	66/29

a Determined by ^1^H NMR analysis using an internal standard (see ESI for details).

b Isolated yield.

The generality of the formal homologation was assessed using a broad range of aryl and heteroarylboronic acids (Scheme 2a). The reaction accommodated variation of electronic and steric substitution including combinations thereof. Of note was the positive impact of *ortho*-substitution (e.g., **6**, **12**, **14**, **20**, **25**), which may be due to the influence of *ortho*-groups on competing esterification.^64^ Electrophile chemoselectivity was observed (**33**–**36**), which gave good yields of chloride products **33**–**35**, but a low yield for bromide adduct **36**. These yields agreed with the relative reactivity of **2-Br** vs Ar–X established in parallel (vide infra). Several heterocycles were well tolerated including thiophenes (**39**–**41**), pyridine (**42**), furans (**43**, **44**), and isoxazole (**45**). Some limitations of the boronic acid scope included specific functional groups (**46**, **47**), heterocycles (**48**, **49**), styrene boronic acid (**50**), and alkylboronic acids (**51**). In general, unprotected heteroatoms/coordinating groups were recalcitrant across the scope assessment based on a robustness screen evaluation (see ESI). Standard reaction scale was 0.2 mmol; however, reactions were effective at 2.5, 5, and 10 mmol scale (examples **42**, **15**, and **6**, respectively).

**Scheme 2:**
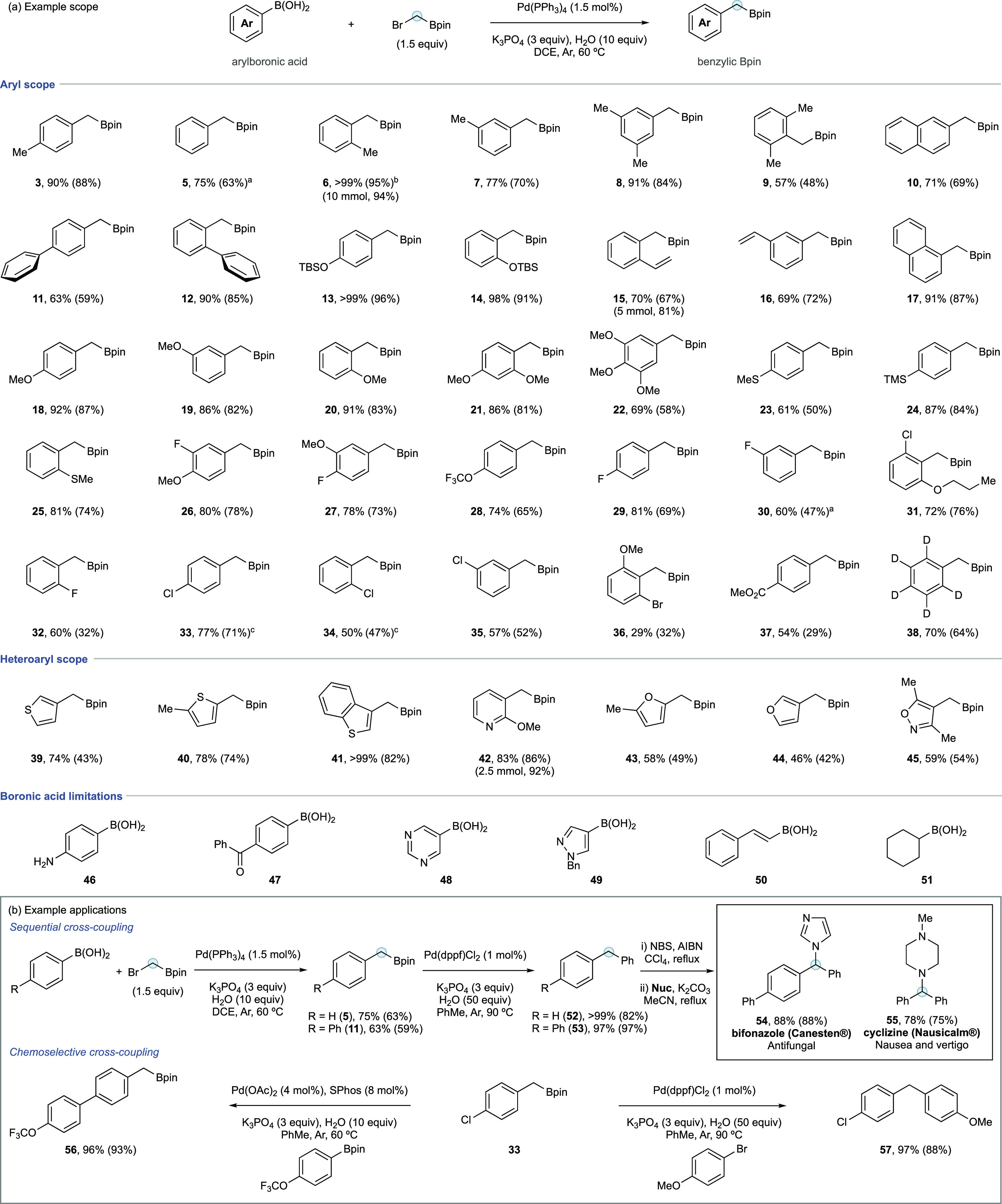
(a) Example Scope of the Formal Homologation Process; Variation of Arylboronic Acid Including Limitations; (b) Examples of Synthetic Applications Including Sequential and Chemoselective Cross-Coupling Solvent = PhOMe. Solvent = PhMe. Temperature = 45 °C. Yields determined by ^1^H NMR using an internal standard, isolated yields in brackets.

Benzylic Bpins are broadly useful in synthetic chemistry, especially within cross-coupling processes. To highlight the utility of the homologation process, we demonstrated application in two cross-coupling scenarios (Scheme 2b). Diarylmethanes are important pharmacophores that can be readily accessed through cross-coupling of benzylic Bpin. The homologation process gives access to diarylmethanes via sequential cross-coupling, delivering intermediates **5** and **11** and then, following Suzuki–Miyaura coupling, intermediate diarylmethanes **52** and **53**. These underwent bromination and alkylation with *N*-nucleophiles to deliver the bioactive agents bifonazole (**54**) and cyclizine (**55**).

The increased reactivity of bromomethyl Bpin in comparison to that of aryl chlorides (vide infra) gave access to chlorobenzyl Bpin **33**. This allows chemoselective Suzuki–Miyaura coupling at either the chloro or Bpin termini to deliver biaryl **56** or diarylmethane **57**.

A series of investigations were undertaken to explore the reactivity of the halomethyl Bpin reagent and to understand key limitations (Scheme 3). Regarding the enhanced electrophilicity of the C–Br bond imparted by the proximity of the boryl unit, Matteson approximated a ∼300 to 700-fold increase in reactivity in nucleophilic substitution reactions.^33^ Under the catalytic conditions above, the effect of the boron unit is immediately apparent from control and competition experiments.

**Scheme 3:**
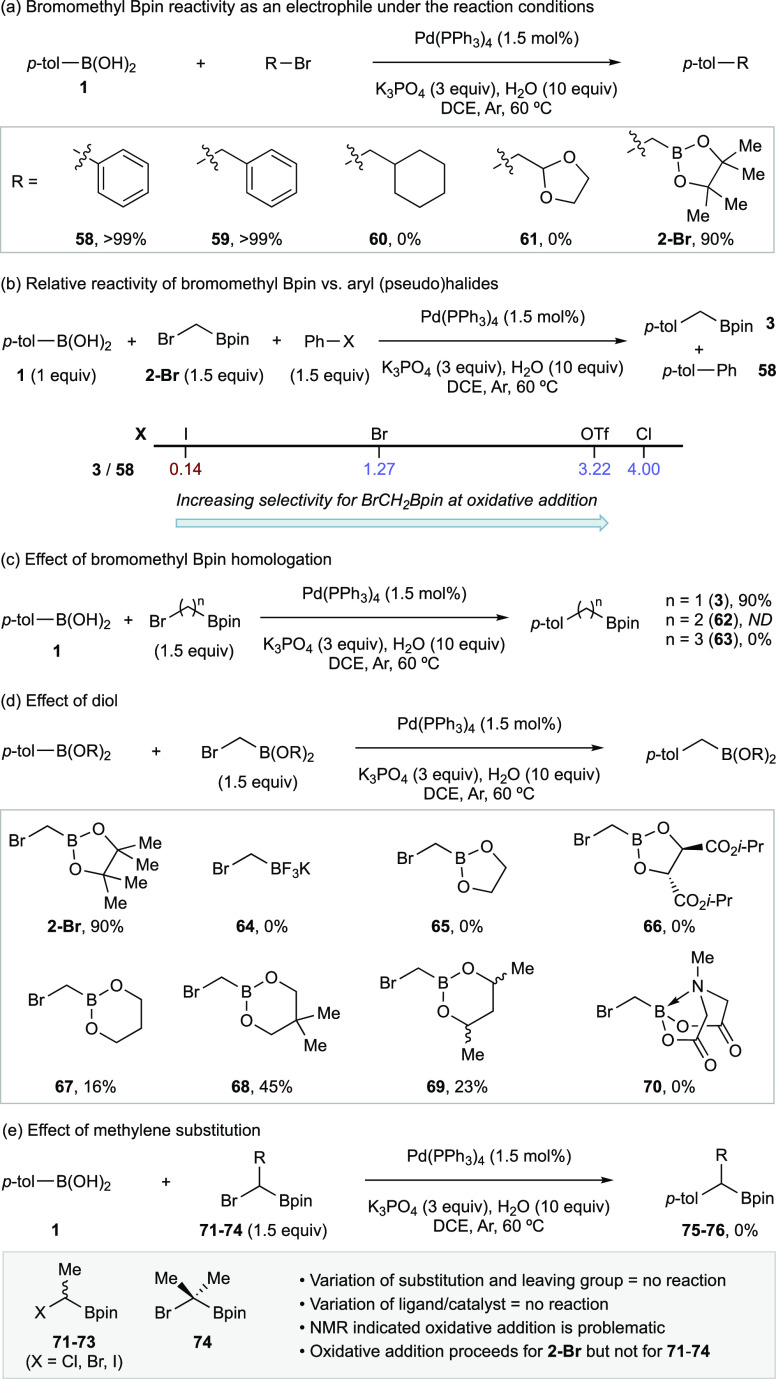
(a) Comparison of Electrophile Reactivity in Isolated Experiments; (b) Comparison of Electrophile Reactivity in Competition Experiments; (c) Effect of Homologation on Reactivity Imparted by the Boron Unit; (d) Effect of the Boronic Ester Diol on the Homologation Reaction; (e) Effect of Substitution on the Methylene

In comparison to several different bromide-derived electrophiles, **2-Br** has similar reactivity to bromobenzene and benzyl bromide in comparative Suzuki–Miyaura couplings with **1** (Scheme 3a). This was supported by competition experiments between **2-Br** and (pseudo)halobenzenes where **2-Br** was significantly more reactive than chlorobenzene and benzene trifluoromethylsulfonate, less reactive than iodobenzene, and exhibited similar reactivity to bromobenzene (Scheme 3b).

Control reactions indicated lack of reactivity under Gevorgyan’s conditions and performing the reaction in light or dark had no influence.^51,52^ In attempts to determine if the boryl unit can influence reactivity at greater distances from the C–B bond, we endeavored to examine the ethyl and propyl homologues (Scheme 3c). Unfortunately, 1,2-bromoethyl Bpin could not be prepared via any approach attempted by us and external suppliers (see ESI); however, 1,3-bromopropyl Bpin could be obtained (see ESI) but was unreactive to the coupling with **1**. The reaction was found to be highly sensitive to the ester diol (Scheme 3d).

Any change from the Bpin was detrimental—poor to moderate yields were observed only with propan-1,3-diol derivatives, with no product observed in any other case and this did not seem to track with established general stability of the boronic esters to protodeboronation;^65−67^ however, the lack of reactivity of **64** and **70** was perhaps expected based on the impact of boron hybridization on the stability of positive charge in the α-position.^68^ Otherwise, these effects are unclear. Finally, substitution on the methylene was not tolerated, restricting the reaction to a single unsubstituted methylene homologation: no reactivity was observed with **68**–**71** (Scheme 3e). This lack of reactivity was found to be due to sluggish oxidative addition. NMR investigations established that **2-Br** undergoes smooth oxidative addition, while no oxidative addition is observed when substitution was introduced to the methylene (see ESI). Attempts to improve oxidative addition with **68**–**71** by variation of reaction conditions and ligand/precatalysts were unsuccessful (see ESI).

In summary, a formal homologation of arylboronic acids has been developed based on the use of bromomethyl Bpin as carbenoid equivalent. The process allows for the direct synthesis of relatively rare benzylic boronic acid esters from aryl boronic acids without the use of organometallic reagents. Control experiments have provided information on the reactivity identified in this system, which support enhanced oxidative addition enabled by proximity to the C–B bond and a highly specific reactivity and stability profile of the halomethylboronic acid pinacol ester.

## Supporting Information Available

Experimental procedures, details of control experiments, and characterization data for all compounds (PDF)

## Abbreviations

AIBN: azobisisobutyronitrile
Ar: argon
dba: dibenzylideneacetone
dppf: 1,1′-bis(diphenylphosphino)ferrocene
DCE: 1,2-dichloroethane
ESI: electronic supporting information
NBS: *N*-bromosuccinimide
ND: not determined
NMR: nuclear magnetic resonance
pin: pinacol/pinacolato
Tf: trifluoromethanesulfonyl
tol: tolyl
