# Supplementary material for: Pd-Catalyzed Organometallic-Free Homologation of Arylboronic Acids Enabled by Chemoselective Transmetalation
Source: ACS Catal. 2023 May 9;13(10):7013–8. doi: 10.1021/acscatal.3c00921 (PMC10204063; doi:10.1021/acscatal.3c00921)
Supplement: Supplementary file 1 — cs3c00921_si_001.pdf [file cs3c00921_si_001.pdf]

A Pd-catalyzed organometallic-free homologation of arylboronic acids enabled by chemoselective transmetalation

Kane A. C. Bastick and Allan J. B. Watson*

EaSTCHEM, School of Chemistry, University of St Andrews, North Haugh, St Andrews, Fife, KY16 9ST, Scotland.

*Email: aw260@st-andrews.ac.uk

Supporting Information

Contents

1. General 2

2. Reaction optimization 4

3. General procedures 9

4. Characterization of starting materials and products 12

   4.1 Experimental procedures 12

   4.2 Summary of attempted syntheses of 1,2-bromoethyl BPin 48

   4.3 Copies of NMR spectra 50

5. Representative sample of unsuccessful substrates 172

6. Hammett parameter analysis of successful products 173

7. Procedures for control studies 174

   7.1 Measuring the electrophilicity of bromomethyl BPin **2-Br** (Scheme 3a) 174

   7.2 Competition study (Scheme 3b) 174

   7.3 Effect of halomethyl BPin homologation (Scheme 3c) 175

   7.4 Effect of diol (Scheme 3d) 175

   7.5 Linchpin selectivity 176

   7.6 Stability of carbenoid surrogate **2-Br** 177

   7.7 Proposed fate of excess **2-Br** 177

   7.8 Product stability 178

   7.9 Secondary methylene homologation trials 178

   7.10 Monitoring oxidative addition 180

8. Additive study 182

9. References 183

1. General

Reagents and solvents were obtained from commercial suppliers and were not purified further unless specified. All boronic acids were purchased from commercial suppliers (Fluorochem, Sigma Aldrich, Alfa Aesar, or Apollo Scientific) and were used as received. THF and PhMe were obtained from a PureSolv SPS-400-5 solvent purification system. DCE was stored over 4 Å molecular sieves for at least 24 h prior to degassing by freeze-pump-thaw (three cycles, Ar). K_3PO_4 was ground using a pestle and mortar then stored in a vacuum oven at 60 °C for at least 48 h prior to use. Triisopropyl borate (Fluorochem) was either distilled into a flask of 4 Å molecular sieves or stored on charged 4 Å molecular sieves for at least 24 h (<5% yield variation during lithiation-borylation protocols). $Pd(PPh_3)_4$ was stored in an Ar-filled glovebox and removed in 100 mg portions, which could be stored in an Ar-purged vial fitted with a septum for up to four weeks in a fridge.

Reactions were carried out in borosilicate round-bottomed flasks or microwave vials with septum caps. Glassware was flame-dried under a vacuum and cooled under an atmosphere of Ar. Microwave vials were stored in an oven at 180 °C prior to use. Room temperature was approximately 18 °C. Reactions at elevated temperatures were conducted using a temperature regulated hotplate with sand bath where the temperature indicated was the sand bath temperature. Reactions performed at temperatures −78 °C or under were done using an acetone slurry with LN_2. Degassing was performed by the freeze-pump-thaw technique over three cycles. Additions over times greater than 10 min were performed using a World Precision Instruments Aladdin-220 syringe pump.

TLC was carried out using Merck aluminum-backed silica plates coated with F_{254} fluorescent indicator, analysed under UV light and/or developed using ethanolic vanillin. Column chromatography was performed using silica gel (40−62 µm, Fluorochem) and was capped with boric acid for boronic esters according the procedure stated below. When used as eluent, Et_2O (Fisher Scientific, Honeywell) was distilled before use.

IR spectra were recorded on a Shimadzu IR Affinity-1 Fourier transform IR (FT-IR) spectrophotometer fitted with a Specac Quest ATR accessory (diamond puck). The spectra were recorded as specified in the procedure as films using CH_2Cl_2 or $CHCl_3$. Transmittance is recorded with maximal absorption wavenumbers given as cm^{-1}. Electrospray ionization (ESI) HRMS was recorded on either a Bruker Microtof II or a Bruker 12T FT mass spectrometer at the University of Edinburgh mass spectrometry facility (SIRCAMS). Electron impact ionization (EI) and chemical impact ionization (CI) HRMS were recorded on a Thermo Mat 99xl sector instrument at the University of Edinburgh mass spectrometry facility (SIRCAMS). ^{1}H, ^{13}C {^{1}H}, ^{19}F {^{1}H} and ^{31}P {^{1}H} spectra were recorded on either a Bruker AV-III HD 500 fitted with a SmartProbe BBFO+ probe (^{1}H 500 MHz; ^{13}C 126 MHz; ^{19}F 470 MHz, ^{31}P 202 MHz), or a Bruker AVIII 500 fitted with a CryoProbe Prodigy BBO

probe (^{1}H 500 MHz; ^{13}C 126 MHz). ^{11}B spectra were obtained on a Bruker AV 300 fitted with a BBFO probe (^{11}B 96 MHz).

All spectra were recorded at rt with the deuterated solvents used as a lock for spectra and internal reference ($CDCl_3$: ^{1}H, 7.26 ppm; ^{13}C, 77.16 ppm; CD_3CN: ^{1}H, 1.94 ppm, ^{13}C, 118.26 ppm; THF-d_8: ^{1}H, 3.58 ppm; ^{13}C, 67.57 ppm). For ^{11}B NMR, samples were run using a standard borosilicate tube and the spectra baselines corrected during processing unless the sample size prevented an acceptable signal-to-noise ratio. In these cases, quartz NMR tubes were used. All ^{11}B NMR spectra were externally referenced to F_3B•OEt_2 in $CDCl_3$. All chemical shifts (δ) are reported in parts per million (ppm) relative to the residual solvent peak. Multiplicity is given as br (broad), s (singlet), d (doublet), t (triplet), q (quartet), m (multiplet), and combinations thereof. Signals which overlap with one another are described as multiplets. All coupling constants, *J*, are quoted in Hz and are $^{3}J_{HH}$ unless otherwise stated.

2. Reaction optimization

Representative optimization procedure:

To an oven-dried microwave vial equipped with a 5 mm beveled ‘tic-tac’ stir bar was added in the following order: solid dried base, the boronic acid, and the catalyst. The vial was then capped and purged thrice with Ar prior to the addition of dry solvent, then $BrCH_2BPin$ (**1-Br**), then water. The reaction mixture was heated to the appropriate temperature (sand bath, immersion depth approx. twice the reaction volume) and the reaction stirred for the listed time. The vial was cooled to rt, decapped and the reaction diluted in CH_2Cl_2, then filtered through a pipette plug of Celite, eluting with CH_2Cl_2, and concentrated at reduced pressure. The residue was suspended in $CDCl_3$ and crude ^1H NMR yield was determined using trichloroethylene (TCE) as a standard (δ_H ($CDCl_3$) = 6.50 ppm). Throughout the displayed phase of optimization below, no arylboronic acid homocoupling was observed. A representative sample of the full optimization screen is shown.

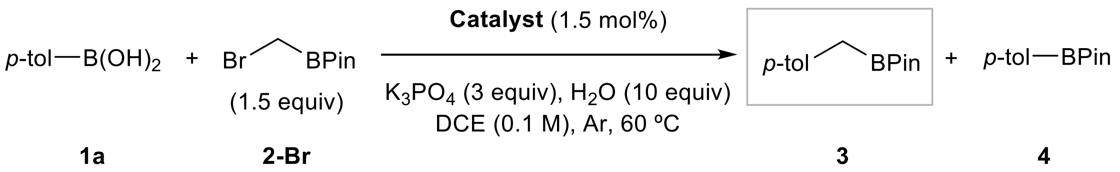

Entry	Catalyst	% Yield 3	% Yield 4
**1**	**Ph(PPh_3)$_4$**	**90 (86)[a]**	**10 (14)[a]**
2	Pd(dba)$_2$	7	93
3[b]	Pd(dba)$_2$ / PPh_3 1:2	91	9
4	Pd(dba)$_2$ / PPh_3 1:1	70	30
5	Pd(dba)$_2$ / PPh_3 1:3	34	68
6	Pd(dba)$_2$ / PPh_3 1:4	34	66
7	Pd(dba)$_2$ / P(*o*-Tol)$_3$ 1:2	71	35
8	Pd(dba)$_2$ / P(*p*-Tol)$_3$ 1:2	14	86
9	Pd(dba)$_2$ / P(Mes)$_3$ 1:2	14	86
10	Pd(dba)$_2$ / P(*p*-OMePh)$_3$ 1:2	20	80
11	Pd(dba)$_2$ / P(*p*-FPh)$_3$ 1:2	23	71
12	Pd(dba)$_2$ / P(Furyl)$_3$ 1:2	10	91
13	Pd(dba)$_2$ / SPhos 1:2	6	99

14	$Pd(dba)_2$ / XPhos 1:2	7	96
15	$Pd(dba)_2$ / $P(Cy)_3$ 1:2	48	52
16	$Pd(dba)_2$ / $P(t\text{-}Bu)_3{\bullet}HBF_4$ 1:2	31	69
17	$Pd(dba)_2$ / $P(n\text{-}Bu)_3{\bullet}HBF_4$ 1:2	13	83
18	$Pd(dba)_2$ / $P(n\text{-}Bu)_3$ 1:2	12	80
19	$Pd(dba)_2$ / $P(tcep)_3$ 1:2	5	86
20	$Pd(dba)_2$ / CataCXium A 1:2	34	70
21	$PdBr_2(PPh_3)_2$	31	69
22	$PdCl_2(PPh_3)_2$	16	86
23	$PdCl_2$ / PPh_3 1:2	17	83
24	$Pd(dppf)Cl_2$	8	88
25	$Pd(OAc)_2$ / SPhos 1:2	8	58
26	$Pd(OAc)_2$ / PPh_3 1:2	64	37
27	XphosPdG2	4	94
28	SPhosPdG2	0	96
29	XantPhosPdG3	0	93
30	Pd(NHC-I)	0	107
31	Pd(NHC-II)	0	100

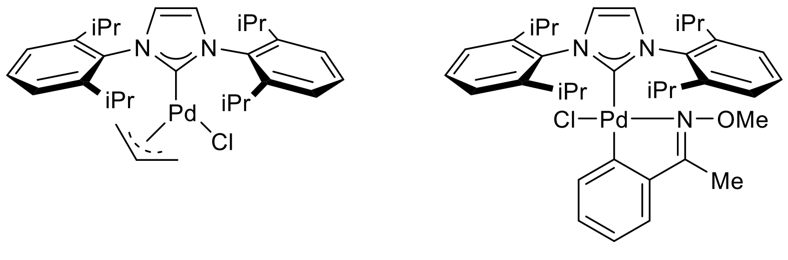

Pd(NHC-I) **Pd(NHC-II)**

Scheme S1: Catalyst screen. [a]Reaction performed in the dark (tin foil wrapped microwave vial). [b]While Entry 3 afforded a slightly greater yield than Entry 1, $Pd(PPh_3)_4$ was selected out of simplicity. We have also found using the conditions according to Entry 3 to be less reproducible, which was attributed to the relative purity of $Pd(dba)_2$. See the additive study (Section 8) for further details.

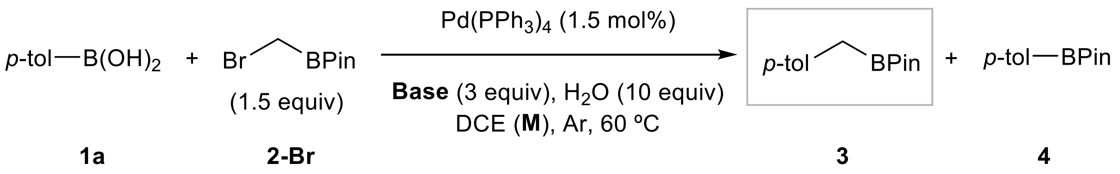

Entry	Base	[Reaction] / M	% Yield 3	% Yield 4
**1**	**K_3PO_4**	**0.10**	**90**	**10**
2	K_3PO_4	0.05	40	49
3	K_2CO_3	0.10	41	50
4	K_2CO_3	0.05	53	35
5	Cs_2CO_3	0.10	38	41
6	Cs_2CO_3	0.05	52	49

Scheme S2: Screen of base and concentration.

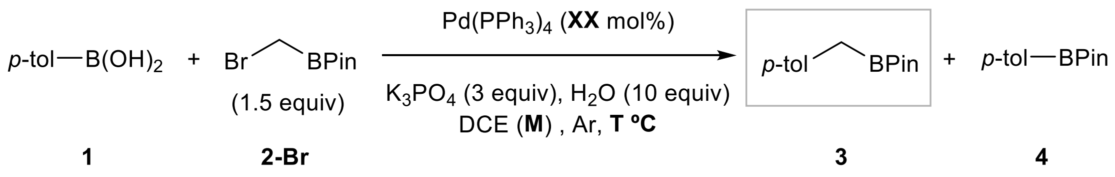

Entry	x mol%	[Reaction] / M	T / °C	% Yield 3	% Yield 4
1	1.5	0.25	60	70	39
2	1.5	0.25	60	70	39
**3**	**1.5**	**0.10**	**60**	**90**	**10**
4	1.5	0.50	60	57	51
5	2.0	0.25	60	64	39
6	2.0	0.10	60	64	54
7	2.0	0.50	60	44	66
8	4.0	0.25	60	69	35
9	4.0	0.10	60	56	44
10	4.0	0.50	60	67	28
11	1.5	0.25	50	72	20
12	1.5	0.10	50	74	21
13	1.5	0.50	50	67	33
14	2.0	0.25	50	67	34
15	2.0	0.10	50	76	57
16	2.0	0.50	50	50	62
17	4.0	250	50	74	47
18	4.0	100	50	77	54
19	4.0	500	50	60	51

Scheme S3: Trivariable screen of catalyst loading, concentration, and temperature.

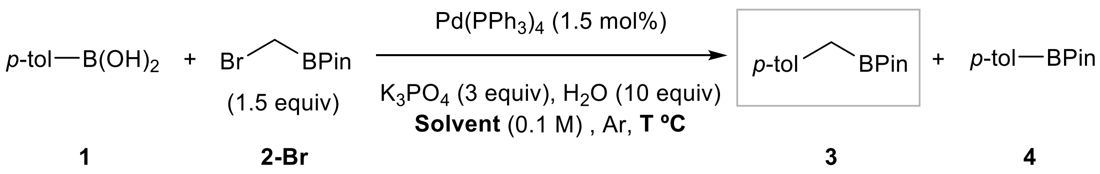

Entry	T / °C	Solvent	% Yield 3	% Yield 4
1	60	PhOMe	70	31
2	60	PhMe	70	39
3	60	2-MeTHF	68	19
4	60	1,4-Dioxane	30	19
5	60	EtOAc	36	76
**6**	**60**	**DCE**	**90**	**10**
7	70	PhOMe	67	32
8	70	PhMe	57	50
9	70	2-MeTHF	31	85
10	70	1,4-Dioxane	35	72
11	70	EtOAc	42	64
12	70	DCE	74	41
13	80	PhOMe	56	45
14	80	PhMe	57	46
15	80	2-MeTHF	44	52
16	80	1,4-Dioxane	39	65
17	80	EtOAc	45	65
18	80	DCE	70	28

Scheme S4: Bivariable screen of temperature and solvent.

3. General procedures

General Procedure A: Preparation of halomethylboronic esters

For example, 2-(bromomethyl)-4,4,5,5-tetramethyl-1,3,2-dioxaborolane (**2-Br**)

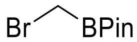

This procedure is adapted from Aggarwal and coworkers.[1] To a flame-dried three-necked flask backfilled under an atmosphere of Ar was added dry THF (~0.3 M), the dihalide e.g. dibromomethane (1.20 equiv), then triisopropyl borate (1.10 equiv). The solution was cooled to <–80 °C (LN_2/acetone slush bath, internal thermometer temperature) then *n*BuLi in hexanes (1.00 equiv) was added dropwise using a syringe pump over 2 h at <–80 °C. *We have found that the purity and yield of the final product is significantly affected if the internal temperature is allowed to rise above ~ –75 °C at this stage. nBuLi should be added by dropping directly into the reaction mixture, not by running down the side of the flask.* The resulting mixture was stirred for 1 h at −80 °C, and then the cooling bath was removed, allowed to warm to rt (~ 30 min), then stirred for 2 h at rt. The reaction mixture was cooled to 0 °C (ice / brine) and methanesulfonic acid (1.00 equiv) was added dropwise over 10 min using a syringe pump, then the reaction mixture was warmed to rt, stirred for 1 h, and the diol, e.g. pinacol (1.00 equiv) was added in a single portion. The septum was returned, and the reaction was stirred overnight at rt whereupon the volatiles were removed at reduced pressure. The cream residue was triturated with CH_2Cl_2 (30 mL) and the suspension filtered, washing the LiBr filter cake with CH_2Cl_2 (3 x 30 mL). The liquor was concentrated at reduced pressure to yield the crude. Desired products were purified by vacuum distillation with the bay and FC lights turned off.

General Procedure B: Preparation of arylboronic esters

For example, 4,4,5,5-tetramethyl-2-(*p*-tolyl)-1,3,2-dioxaborolane

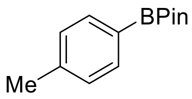

An open flask fitted with a stir bar was charged with the boronic acid, e.g. 4-tolyboronic acid (1.00 equiv), the diol, e.g. pinacol (1.10 equiv), and Na_2SO_4 (2.5 equiv) followed by Et_2O (100 mL, ~0.2 M) and the flask fitted with a septum and needle inlet (air). The flask was stirred at ambient temperature overnight (16–24 h), filtered and concentrated at reduced pressure, then resuspended in CH_2Cl_2 (100 mL) and warm water (~40 °C, 50 mL, *solubilises excess diol*). The organics were

extracted with CH_2Cl_2 (2 x) then dried (Na_2SO_4), filtered, and concentrated at reduced pressure. If column chromatography was required, silica gel was used.

General Procedure C: Optimized homologation of boronic acids

For example, 4,4,5,5-tetramethyl-2-(4-methylbenzyl)-1,3,2-dioxaborolane (**3**)

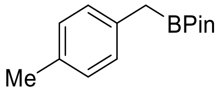

To an oven-dried microwave vial equipped with a stir bar was added in the following order: dry K_3PO_4 (127 mg, 0.60 mmol, 3.0 equiv), the boronic acid, e.g. Compound **1** (27.2 mg, 0.20 mmol, 1.0 equiv), and $Pd(PPh_3)_4$ (3.5 mg, 0.015 mmol, 1.5 mol%), then the vial was capped and purged with Ar prior to the addition of DCE (2.0 mL, 0.1 M), then $BrCH_2BPin$ (**2-Br**) (54 µL, 0.30 mmol, 1.5 equiv), then water (36 µL, 2.0 mmol, 10 equiv) and the microwave vial capped, heated to 60 °C and stirred for 24 h (unless otherwise stated). The reaction mixture was cooled to rt and diluted in CH_2Cl_2 (5 mL) then filtered through Celite, eluting with CH_2Cl_2 and concentrated at reduced pressure. Crude ^1H NMR yield was determined using trichloroethylene as a standard prior to purification on B-SiO_2.

General Procedure D: Scaleup homologations of boronic acids (≥ 2.5 mmol)

For example, 2-([1,1'-biphenyl]-4-ylmethyl)-4,4,5,5-tetramethyl-1,3,2-dioxaborolane (**11**)

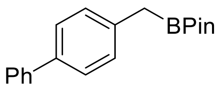

To a flame-dried two-necked flask equipped with a reflux condenser and septum cooled under an atmosphere of Ar was added dry K_3PO_4 (3.0 equiv), the boronic acid, e.g. 4-biphenylboronic acid (1.00 equiv), and $Pd(PPh_3)_4$ (1.5 mol%), then the flask evacuated and backfilled with Ar thrice prior to the addition of DCE (0.1 M), then $BrCH_2BPin$ (**2-Br**) (1.5 equiv), then water (10 equiv). The reaction mixture was heated to 60 °C and stirred for 24 h. The reaction mixture was diluted in CH_2Cl_2 and filtered through Celite, eluting with CH_2Cl_2, then concentrated at reduced pressure to afford the crude which was purified by B-SiO_2 chromatography.

General Procedure E: Benzylic cross-couplings

For example, diphenylmethane (**52**)

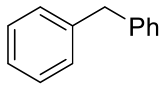

To an oven-dried microwave vial fitted with a stir bar was added dried K_3PO_4 (3.0 equiv), then the BPin ester (3.0 equiv) then Pd(dppf)Cl_2 (1.0 mol%) and the vial capped and purged thrice with Ar prior to the addition of PhMe (0.25 M), then the halide, bromobenzene (1.00 equiv), then water (50 equiv) and the reaction mixture stirred at 90 °C for 24 h. The vial was decapped and the crude reaction mixture was filtered through a short pad of Celite, eluting with CH_2Cl_2, and concentrated at reduced pressure. The crude was purified by column chromatography using silica gel (see General).

Purification of homologated BPins using boric acid-capped silica (B-SiO_2)

In many instances, purification of the crude reaction mixture by standard chromatographic techniques on silica gel failed to separate the desired homologation product from the undesired transesterified product, formed by speciation of pinacol from the homologation reagent to the substrate boronic acid; despite separation being visible by silica gel TLC using vanillin stain (see below). When both products are observed, the R_f value of the desired homologated product is typically lower (R_f = 0.3–0.4) than that of the undesired transesterified product (R_f = 0.4–0.5).

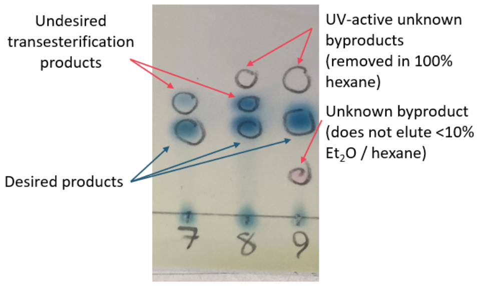

Figure S1: Stained TLC plates (vanillin) of three crude reaction mixtures.

To remedy products from streaking, the separation of any compound containing a BPin ester was achieved using B-SiO_2, which also maintained good isolated yields of the products in comparison to obtained NMR yields (yield loss typically <15%). B-SiO_2 for column chromatography was prepared according to Snaddon with silica gel TLC plates used for visualization of fractions upon staining with ethanolic vanillin.[2]

4. Characterization of starting materials and products

4.1 Experimental procedures

2-(3-bromopropyl)-4,4,5,5-tetramethyl-1,3,2-dioxaborolane (S1)

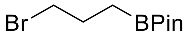

Based on a procedure by Thomas and coworkers.[3] Allyl bromide (0.44 mL, 5.0 mmol, 1.0 equiv) and HBPin (0.80 mL, 5.5 mmol, 1.1 equiv) were added neat to a flame dried Young's tube containing $LiAlH_4$ (19 mg, 0.50 mmol, 10 mol%) at rt, in this order as single portions, under an atmosphere of Ar. *Safety: gas evolution*. The reaction mixture was stirred for 4 h at 110 °C, then cooled to rt, opened to air, and filtered through a short pad of silica eluting with CH_2Cl_2 20 mL. The crude was subject to column chromatography on standard silica gel (0−10% EtOAc in hexane) to afford the desired product as a straw coloured oil (750 mg, 60%).

^{1}H NMR (500 MHz, $CDCl_3$) δ 3.42 (t, *J* = 6.9 Hz, 2 H), 2.01–1.92 (m, 2 H), 1.24 (s, 12 H,), 0.94−0.90 (td, *J* = 7.5, 2.3 Hz, 2 H).

^{13}C NMR (126 MHz, $CDCl_3$) δ 83.4, 36.4, 27.7, 25.0. The boron-bearing carbon was not observed due to quadrupolar relaxation.

^{11}B NMR (96 MHz, $CDCl_3$) δ 34.03.

These data were consistent with that of the literature.[3]

2-(*p*-tolyl)-1,3,2-dioxaborolane (S2)

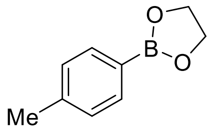

To an open flask charged with a stir bar was added Compound **1** (680 mg, 5.0 mmol, 1.00 equiv), PhMe (20 mL), then ethylene glycol (313 mg, 5.05 mmol, 1.01 equiv). The flask was fitted with a Dean−Stark apparatus charged with PhMe then reaction mixture was heated to vigorous reflux (hotplate 140 °C) and stirred for 16 h, cooled to rt, then concentrated at reduced pressure to afford the desired product as a glassy solid (786 mg, 97%). No further purification was required.

^{1}H NMR (500 MHz, $CDCl_3$) δ 7.72–7.70 (m, 2 H), 7.22–7.20 (m, 2 H), 4.37 (s, 4 H), 2.38 (s, 3 H).

^{13}C NMR (126 MHz, $CDCl_3$) δ 141.8, 135.0, 128.8, 66.1, 21.9. The boron-bearing carbon was not observed due to quadrupolar relaxation.

^{11}B NMR (96 MHz, $CDCl_3$) δ 31.75.

These data were consistent with that of the literature.[4]

2-(*p*-tolyl)-1,3,2-dioxaborinane (S3)

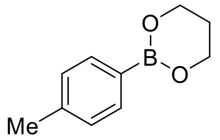

Prepared according to General Procedure B using 4-tolyboronic acid (680 mg, 5.0 mmol, 1.00 equiv) 1,3-propanediol (0.49 mL, 5.05 mmol, 1.01 equiv) and Na_2SO_4 (1.25 g, 12.5 mmol, 2.50 equiv). Following workup, the crude was subject to column chromaotghraphy on B-SiO_2 (3% Et_2O in hexane) to afford the desired product as a straw coloured oil (1.43 g, 65%).

^{1}H NMR (500 MHz, $CDCl_3$) δ 7.70–7.62 (m, 2 H), 7.16 (d, *J* = 7.4 Hz, 2 H), 4.21–4.11 (m, 4 H), 2.36 (s, 3 H), 2.09–2.01 (tt, *J* = 6.2, 5.1 Hz, 2 H).

^{13}C NMR (126 MHz, $CDCl_3$) δ 140.7, 133.8, 128.5, 62.1, 27.6, 21.8. The boron-bearing carbon was not observed due to quadrupolar relaxation.

^{11}B NMR (96 MHz, $CDCl_3$) δ 27.55.

These data were consistent with that of the literature.[5]

4,6-dimethyl-2-(*p*-tolyl)-1,3,2-dioxaborinane (S4)

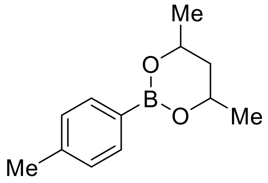

Prepared according to General Procedure B using 4-tolyboronic acid (680 mg, 5.0 mmol, 1.00 equiv) *rac*-2,4-pentanediol (1.11 mL, 10.1 mmol, 2.02 equiv) and Na_2SO_4 (1.25 g, 12.5 mmol, 2.50 equiv). Following workup, the crude was subject to column chromaotghraphy (2% Et_2O in hexane) to afford the desired product as a colourless oil (1.02 g, quant., *syn:anti* 56:54). The diastereomers were not separated.

IR (film) ν 2361, 2342, 1308 cm^{-1}.

syn

^{1}H NMR (500 MHz, $CDCl_3$) δ 7.77–7.68 (m, 2 H), 7.23–7.12 (m, 2 H), 4.28 (dqd, *J* = 11.3, 6.2, 2.7 Hz, 2 H), 2.38 (s, 3 H), 1.85 (t, *J* = 5.3 Hz, 2 H), 1.36 (d, *J* = 6.2 Hz, 6 H).

^{13}C NMR (126 MHz, CDCl$_3$) δ 140.5, 134.0, 128.4, 68.2, 42.7, 23.4, 21.8. The boron-bearing carbon was not observed due to quadrupolar relaxation.

anti

^{1}H NMR (500 MHz, CDCl$_3$) δ 7.77–7.68 (m, 2 H), 7.23–7.12 (m, 2H), 4.42 (dqd, *J* = 11.7, 6.4, 5.2 Hz, 2 H), 2.38 (s, 3 H), 2.00 (dt, *J* = 13.9, 2.8 Hz, 2 H), 1.39 (d, *J* = 6.4 Hz, 6 H).

^{13}C NMR (126 MHz, CDCl$_3$) δ 140.5, 133.9, 128.5, 64.8, 39.4, 22.9, 21.8. The boron-bearing carbon was not observed due to quadrupolar relaxation.

^{11}B NMR (96 MHz, CDCl$_3$) δ 27.23.

HRMS (EI) Exact mass calcd. for $C_{12}H_{17}BO_2$ $[M]^{+\bullet}$ *m/z* = 204.1316; found 204.1315.

5,5-dimethyl-2-(*p*-tolyl)-1,3,2-dioxaborinane (S5)

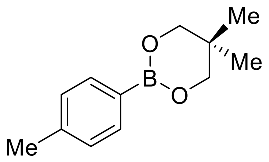

Prepared according to General Procedure B using 4-tolyboronic acid (680 mg, 5.0 mmol, 1.00 equiv), neopentylglycol (526 mg, 5.05 mmol, 1.01 equiv) and Na_2SO_4 (1.25 g, 12.5 mmol, 2.50 equiv). Following workup, the crude was subject to column chromaotghraphy (2% Et_2O in hexane) to afford the desired product as a white solid (878 mg, 86%).

^{1}H NMR (500 MHz, CDCl$_3$) δ 7.72–7.67 (m, 2 H), 7.18–7.16 (m, 2 H), 3.76 (s, 4 H,), 2.36 (s, 3 H), 1.02 (s, 6 H).

^{13}C NMR (126 MHz, CDCl$_3$) δ 140.8, 134.0, 128.6, 72.4, 32.0, 22.1, 21.8. The boron-bearing carbon was not observed due to quadrupolar relaxation.

^{11}B NMR (96 MHz, CDCl$_3$) δ 26.99.

These data were consistent with that of the literature.[6]

4,4,5,5-tetramethyl-2-(4-(trifluoromethoxy)phenyl)-1,3,2-dioxaborolane (S6)

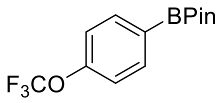

Prepared according to General Procedure B using 4-(trifluoromethoxy)-phenylboronic acid (1.03 g, 5.00 mmol, 1.00 equiv), pinacol (597 mg, 5.05 mmol, 1.10 equiv) and Na_2SO_4 (1.78 g, 12.5 mmol, 2.5

equiv). Following workup, the desired product was obtained as a colourless oil where no further purification was required (1.43 g, 99%)

^{1}H NMR (500 MHz, CDCl$_3$) δ 7.89–7.80 (m, 2 H), 7.22–7.19 (m, 2 H), 1.34 (s, 12 H).

^{13}C NMR (126 MHz, CDCl$_3$) δ 151.8, 136.7, 120.6 (q, $^{1}J_{CF}$ = 257.4 Hz), 120.0, 84.2, 25.0. The boron-bearing carbon was not observed due to quadrupolar relaxation.

^{11}B NMR (96 MHz, CDCl$_3$) δ 30.78.

^{19}F NMR (470 MHz, CDCl$_3$) δ −57.57.

These data were consistent with that of the literature.[7]

2-(bromomethyl)-4,4,5,5-tetramethyl-1,3,2-dioxaborolane (2-Br)

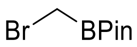

Prepared according to General procedure A using THF (500 mL), dibromomethane (11.9 mL, 169 mmol, 1.20 equiv), triisopropyl borate (35.8 mL, 155 mmol, 1.10 equiv) and *n*BuLi 2.45 M in hexanes (57.6 mL, 141 mmol, 1.00 equiv). Following the addition procedure, methanesulfonic acid (9.15 mL, 141 mmol, 1.00 equiv) and pinacol (15.6 g, 141 mmol, 1.00 equiv) were used. Following workup, the desired product was purified by vacuum distillation (55–57 °C, 6–7 mbar, lit 42–44 °C, 3.5–5.1 mbar[1]) and was stored in the freezer in the absence of light as a colourless liquid (26.8 g, 86%). <u>*Safety: At this scale the product is a lachrymator.*</u>

^{1}H NMR (500 MHz, CDCl$_3$ δ 2.59 (s, 2 H), 1.29 (s, 12 H).

^{13}C NMR (126 MHz, CDCl$_3$) δ 84.7, 24.7. The boron-bearing carbon was not observed due to quadrupolar relaxation.

^{11}B NMR (96 MHz, CDCl$_3$) δ 31.09.

The spectral data were consistent with that of the literature.[1]

2-(iodomethyl)-4,4,5,5-tetramethyl-1,3,2-dioxaborolane (2-I)

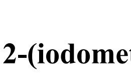

To compound **2-Br** (2.25 g, 10.2 mmol, 1.00 equiv) in acetone (20 mL), was added NaI (2.44 g, 16.3 mmol, 1.6 equiv) in one portion at rt, and the mixture stirred at rt in the dark (tin foil wrapped rbf) for 2 h. Insoluble salts were filtered off and the flask rinsed with acetone (40 mL), and the solvents were evaporated under reduced pressure to afford a residue that was triturated in hexane (50 mL) and the insoluble material filtered off, washing with hexane (2 x 25 mL). The liquor was concentrated under

reduced pressure to obtain the product as a pale yellow liquid (2.55 g, 93%) which was stored in the freezer in the absence of light.

^{1}H NMR (500 MHz, CDCl$_3$) δ 2.16 (2 H, s), 1.27 (12 H, s).

^{13}C NMR (126 MHz, CDCl$_3$) δ 84.3, 24.5.

^{11}B NMR (96 MHz, CDCl$_3$) δ 31.83. The boron-bearing carbon was not observed due to quadrupolar relaxation.

The spectral data were consistent with that of the literature.[8]

2-(chloromethyl)-4,4,5,5-tetramethyl-1,3,2-dioxaborolane (2-Cl)

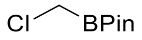

Prepared according to General Procedure A using in THF (250 mL), bromochloromethane (2.34 mL, 36.0 mmol, 1.20 equiv), triisopropyl borate (7.62 mL, 33.0 mmol, 1.10 equiv) and *n*BuLi 2.46 M in hexanes (12.2 mL, 30.0 mmol, 1.00 equiv). Following the addition procedure, methanesulfonic acid (1.95 mL, 30.0 mmol, 1.00 equiv) and pinacol (3.55 g, 30.0 mmol, 1.00 equiv) were used. Following workup, the desired product was purified by vacuum distillation (42–44 °C, 5 mbar, lit 80–82 °C, 19 mbar) and was stored in the freezer in the absence of light as a colourless liquid (7.77 g, 83%).

^{1}H NMR (500 MHz, CDCl$_3$) δ 2.96 (s, 2 H), 1.29 (s, 12 H).

^{13}C NMR (126 MHz, CDCl$_3$) δ 84.7, 24.7. The boron-bearing carbon was not observed due to quadrupolar relaxation.

^{11}B NMR (96 MHz, CDCl$_3$) δ 31.56.

The spectral data were consistent with that of the literature.[9]

4,4,5,5-tetramethyl-2-(4-methylbenzyl)-1,3,2-dioxaborolane (3)

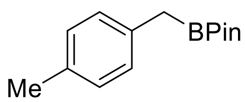

Prepared according to General Procedure C using Compound **1** (27.2 mg, 0.20 mmol). The crude residue (90% ^{1}H NMR yield) was subject to column chromatography on silica gel (0–2% Et_2O in hexane) to afford the title compound as a colorless oil (40.9 mg, 88%).

^{1}H NMR (500 MHz, CDCl$_3$) δ 7.06 (m, 4 H), 2.30 (s, 3 H), 2.25 (s, 2 H), 1.23 (s, 12 H).

^{13}C NMR (126 MHz, CDCl$_3$) δ 135.5, 134.3, 129.1, 129.0, 83.5, 24.9, 21.1. The boron-bearing carbon was not observed due to quadrupolar relaxation.

^{11}B NMR (96 MHz, $CDCl_3$) δ 33.00

The spectral data were consistent with that of the literature.[10]

4,4,5,5-tetramethyl-2-(*p*-tolyl)-1,3,2-dioxaborolane (4)

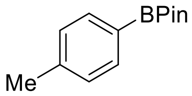

Prepared according to General Procedure B using 4-tolyboronic acid (3.00 g, 22.1 mmol, 1.00 equiv) and pinacol (2.87 g, 24.3 mmol, 1.10 equiv) and Na_2SO_4 (7.84 g, 55.2 mmol, 2.5 equiv) with THF as the solvent. Following workup, the desired product was obtained as a white solid and no further purification was required (4.98 g, quant.).

^{1}H NMR (500 MHz, $CDCl_3$) δ 7.73–7.68 (m, 2 H), 7.21–7.16 (m, 2 H), 2.36 (s, 3 H), 1.34 (s, 12 H).

^{13}C NMR (126 MHz, $CDCl_3$) δ 141.6, 134.9, 128.7, 83.8, 25.0, 21.9. The boron-bearing carbon was not observed due to quadrupolar relaxation.

^{11}B NMR (96 MHz, $CDCl_3$) δ 31.24.

These data were consistent with that of the literature.[11]

2-benzyl-4,4,5,5-tetramethyl-1,3,2-dioxaborolane (5)

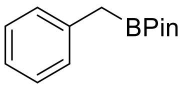

Prepared according to General Procedure C using phenylboronic acid (24.4 mg, 0.20 mmol) and PhMe (2.0 mL, 0.10 M) was the solvent. The crude residue (75% ^{1}H NMR yield) was subject to column chromatography on silica gel (1–2% Et_2O in hexane) to afford the title compound as a colorless oil (27.5 mg, 63%).

^{1}H NMR (500 MHz, $CDCl_3$) δ 7.24 (t, J = 7.5 Hz, 2 H), 7.21–7.16 (m, 2 H), 7.15–7.10 (m, 1 H), 2.30 (s, 2 H), 1.24 (s, 12 H).

^{13}C NMR (126 MHz, $CDCl_3$) δ 138.8, 129.1, 128.4, 125.0, 83.5, 24.9, 20.4. Due to quadrupolar relaxation, the boron-bearing carbon was identified by the HSQC-crosspeak.

^{11}B NMR (96 MHz, $CDCl_3$) δ 33.26.

The spectral data were consistent with that of the literature.[12]

4,4,5,5-tetramethyl-2-(2-methylbenzyl)-1,3,2-dioxaborolane (6)

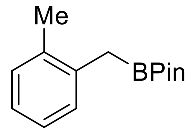

Prepared according to General Procedure C using 2-tolylboronic acid (27.2 mg, 0.20 mmol). Anisole (0.8 mL, 0.25 M) were used as the solvent and the reaction was complete in 6 h. The crude residue (>99% ^{1}H NMR yield) was subject to column chromatography on silica gel (0–2% Et_2O in hexane) to afford the title compound as a colorless oil (41.1mg, 95%).

^{1}H NMR (500 MHz, $CDCl_3$) δ 7.09 (m, 4 H), 2.27 (s, 3 H), 2.25 (s, 2 H), 1.22 (s, 12 H).

^{13}C NMR (126 MHz, $CDCl_3$) δ 137.7, 136.1, 129.9, 129.6, 126.0, 125.3, 83.5, 24.9, 20.2. The boron-bearing carbon was not observed due to quadrupolar relaxation.

^{11}B NMR (96 MHz, $CDCl_3$) δ 32.96.

The spectral data were consistent with that of the literature.[10]

4,4,5,5-tetramethyl-2-(3-methylbenzyl)-1,3,2-dioxaborolane (7)

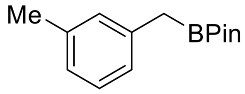

Prepared according to General Procedure C using 3-tolylboronic acid (27.2 mg, 0.20 mmol). The crude residue (78% ^{1}H NMR yield) was subject to column chromatography on silica gel (0–2% Et_2O in hexane) to afford the title compound as a colorless oil (17.9 mg, 39%).

^{1}H NMR (500 MHz, $CDCl_3$) δ 7.13 (t, J = 7.4 Hz, 1 H), 6.99 (m, 2 H), 6.94 (d, J = 7.6 Hz, 1 H), 2.30 (s, 3 H), 2.26 (s, 2 H), 1.24 (s, 12 H).

^{13}C NMR (126 MHz, $CDCl_3$) δ 138.6, 137.9, 130.0, 128.3, 126.1, 125.7, 83.5, 24.9, 21.6. The boron-bearing carbon was not observed due to quadrupolar relaxation.

^{11}B NMR (96 MHz, $CDCl_3$) δ 32.97.

The spectral data were consistent with that of the literature.[10]

2-(3,5-dimethylbenzyl)-4,4,5,5-tetramethyl-1,3,2-dioxaborolane (8)

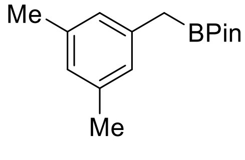

Prepared according to General Procedure C using (3,5-dimethylphenyl)boronic acid (39.6 mg, 0.20 mmol). The crude residue (91% ^{1}H NMR yield) was subject to column chromatography on silica gel (0–2% Et_2O in hexane) to afford the title compound as a colorless oil (23.8 mg, 48%).

^{1}H NMR (500 MHz, $CDCl_3$) δ 6.81 (s, 2 H), 6.77 (s, 1 H), 2.27 (s, 6 H), 2.22 (s, 2 H), 1.24 (s, 12 H).

^{13}C NMR (126 MHz, $CDCl_3$) δ 138.5, 137.7, 127.0, 126.7, 83.5, 24.8, 21.4, 19.8. Due to quadrupolar relaxation, the boron-bearing carbon was identified by the HSQC-crosspeak.

^{11}B NMR (96 MHz, $CDCl_3$) δ 32.92.

The spectral data were consistent with that of the literature.[13]

2-(2,6-dimethylbenzyl)-4,4,5,5-tetramethyl-1,3,2-dioxaborolane (9)

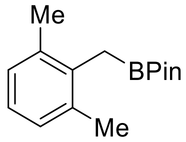

Prepared according to General Procedure C using (2,6-dimethylphenyl)boronic acid (30.0 mg, 0.20 mmol). The crude residue (57% ^{1}H NMR yield) was subject to column chromatography on silica gel (0.1–0.5% Et_2O in hexane) to afford the title compound as a white solid (21.9 mg, 44%).

^{1}H NMR (500 MHz, $CDCl_3$) δ 7.01–6.92 (m, 3 H), 2.39 (s, 6 H), 2.25 (s, 2 H), 1.21 (s, 12 H).

^{13}C NMR (126 MHz, $CDCl_3$) δ 136.7, 135.8, 127.7, 124.6, 83.4, 24.8, 21.0, 14.8. Due to quadrupolar relaxation, the boron-bearing carbon was identified by the HSQC-crosspeak.

^{11}B NMR (96 MHz, $CDCl_3$) δ 33.06.

The spectral data were consistent with that of the literature.[10]

4,4,5,5-tetramethyl-2-(naphthalen-2-yl)-1,3,2-dioxaborolane (10)

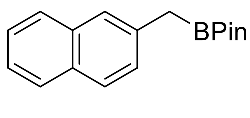

Prepared according to General Procedure C using 2-naphthaleneboronic acid (24.0 mg, 0.14 mmol). The crude residue (71% ^{1}H NMR yield) was subject to column chromatography on silica gel (0–2% Et_2O in hexane) to afford the title compound as a white solid (25.8 mg, 69%).

^{1}H NMR (500 MHz, $CDCl_3$) δ 7.81–7.70 (m, 3 H), 7.68–7.59 (m, 1 H), 7.46–7.30 (m, 3 H), 2.46 (s, 2 H), 1.24 (s, 12 H).

^{13}C NMR (126 MHz, CDCl$_3$)** δ 136.3, 133.8, 131.5, 128.2, 127.7, 127.56, 127.3, 126.6, 125.7, 124.70, 83.5, 20.3, 24.8. The boron-bearing carbon was identified by the HSQC-crosspeak due to quadrupolar relaxation.

^{11}B NMR (96 MHz, CDCl$_3$) δ 33.43.

The spectral data were consistent with that of the literature.[14]

2-([1,1'-biphenyl]-4-ylmethyl)-4,4,5,5-tetramethyl-1,3,2-dioxaborolane (11)

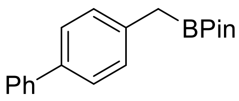

Prepared according to General Procedure C using 4-biphenylboronic acid (39.6 mg, 0.20 mmol). The crude residue (63% ^{1}H NMR yield) was subject to column chromatography on silica gel (0–2% Et_2O in hexane) to afford the title compound as a white solid (34.7 mg, 59%).

The substrate was also prepared according to General Procedure D using 4-biphenylboronic acid (990 mg, 5.0 mmol). The crude residue was subject to column chromatography on silica gel (0–1% Et_2O in hexane) to afford the title compound as a white solid (691 mg, 47%). The loss of yield on scaleup was accounted for by coelution with the starting material pinacol ester byproduct.

^{1}H NMR (500 MHz, CDCl$_3$) δ 7.66–7.55 (m, 2 H), 7.52–7.46 (m, 2 H), 7.46–7.38 (m, 2 H), 7.36–7.28 (m, 1 H), 7.28–7.23 (m, 2 H) 2.34 (s, 2 H), 1.26 (s, 12 H).

^{13}C NMR (126 MHz, CDCl$_3$) δ 141.4, 138.0 , 137.9 , 129.5, 128.8, 127.2, 127.1, 127.0, 83.6, 24.9, 19.8. Due to quadrupolar relaxation, the boron-bearing carbon was identified by the HSQC-crosspeak.

^{11}B NMR (96 MHz, CDCl$_3$) δ 32.89.

The spectral data were consistent with that of the literature.[15]

2-([1,1'-biphenyl]-2-ylmethyl)-4,4,5,5-tetramethyl-1,3,2-dioxaborolane (12)

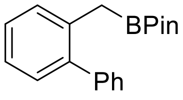

Prepared according to General Procedure C using 4-biphenylboronic acid (39.6 mg, 0.20 mmol). The crude residue (90% ^{1}H NMR yield) was subject to column chromatography on silica gel (0–2% Et_2O in hexane) to afford the title compound as a colorless oil (50.0 mg, 85%).

^{1}H NMR (500 MHz, CDCl$_3$) 7.42–7.29 (m, 5 H), 7.23–7.17 (m, 4 H), 2.29 (s, 2 H), 1.15 (s, 12 H).

^{13}C NMR (126 MHz, CDCl$_3$) 142.4, 141.8, 136.7, 130.3, 130.1, 129.6, 128.1, 127.4, 126.8, 125.3, 83.4, 24.9, 18.7. Due to quadrupolar relaxation, the boron-bearing carbon was identified by the HSQC-crosspeak.

^{11}B NMR (96 MHz, CDCl$_3$) δ 32.58.

The spectral data were consistent with that of the literature.[10]

***tert*-butyldimethyl(4-((4,4,5,5-tetramethyl-1,3,2-dioxaborolan-2-yl)methyl)phenoxy)silane (13)**

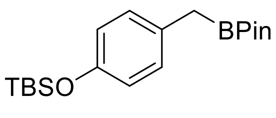

Prepared according to General Procedure C using 4-(*tert*-butyldimethylsilyl)phenylboronic acid (50.4 mg, 0.20 mmol). The crude residue (93% ^{1}H NMR yield) was subject to column chromatography on silica gel (0–2% Et_2O in hexane) to afford the title compound as a white solid (58.6 mg, 84%).

IR (ATR, film) υ_{max} 2930, 2859, 2361, 2342, 1508, 1256, 1144, 916, 839 cm^{-1}.

^{1}H NMR (500 MHz, CDCl$_3$) δ 7.05–7.00 (m, 2 H), 6.74–6.68 (m, 2 H), 2.21 (s, 2 H), 1.22 (s, 12 H), 0.97 (s, 9 H), 0.17 (s, 6 H).

^{13}C NMR (126 MHz, CDCl$_3$) δ 153.1, 131.2, 129.9, 120.0, 83.5, 25.9, 24.8, 19.0, 18.3, −4.3. Due to quadrupolar relaxation, the boron-bearing carbon was identified by the HSQC-crosspeak.

^{11}B NMR (96 MHz, CDCl$_3$) δ 32.96.

HRMS (EI) Exact mass calculated for $C_{19}H_{33}BO_4Si$ $[M+O]^{+\bullet}$ *m/z* = 364.2236; found 364.2225.

***tert*-butyldimethyl(2-((4,4,5,5-tetramethyl-1,3,2-dioxaborolan-2-yl)methyl)phenoxy)silane (14)**

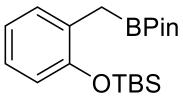

Prepared according to General Procedure C using 2-((tert-butyldimethylsilyl)oxy)phenyl)boronic acid (50.4 mg, 0.20 mmol). The crude residue (98% ^{1}H NMR yield) was subject to column chromatography on silica gel (0–1% Et_2O in hexane) to afford the title compound as a white residue (62.1 mg, 91%).

The substrate was also prepared according to General Procedure D using 2-((tert-butyldimethylsilyl)oxy)phenyl)boronic acid (504 mg, 2.0 mmol). The crude residue was subject to column chromatography on silica gel (1–2% Et_2O in hexane) to afford the title compound as a colorless oil (990 mg, 81%).

IR (ATR, film) υ_{max} 2361, 2342, 1489, 1327, 1252, 1144, 924, 837, 779 cm^{-1}.

^{1}H NMR (500 MHz, CDCl$_3$) δ 7.15 (dd, *J* = 7.5, 1.8 Hz, 1 H), 7.01 (m, 1 H), 6.85 (m, 1 H), 6.76 (d, *J* = 8.0 Hz, 1 H), 2.24 (s, 2 H), 1.22 (s, 12 H), 1.01 (s, 9 H), 0.22 (s, 6 H).

^{13}C NMR (126 MHz, CDCl$_3$) δ 153.4, 130.8, 130.1, 126.0, 121.1, 118.6, 83.3, 26.1, 24.9, 18.5, 14.7 (br.), −4.0. Due to quadrupolar relaxation, the boron-bearing carbon has a weak signal-to-noise ratio and was confirmed by the HSQC-crosspeak.

^{11}B NMR (96 MHz, CDCl$_3$) δ 34.07.

HRMS (EI) Exact mass calculated for $C_{19}H_{33}BO_3Si$ $[M]^{+\bullet}$ *m/z* = 348.22865; found 348.22810.

4,4,5,5-tetramethyl-2-(2-vinylbenzyl)-1,3,2-dioxaborolane (15)

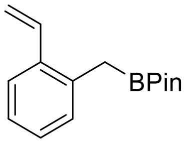

Prepared according to General Procedure C using 2-vinylphenylboronic acid (29.6 mg, 0.20 mmol). The crude residue (70% ^{1}H NMR yield) was subject to column chromatography on silica gel (0.5–1% Et_2O in hexane) to afford the title compound as a colorless oil (32.8 mg, 67%).

The substrate was also prepared according to General Procedure D using 2-vinylphenylboronic acid (990 mg, 5.0 mmol). The crude residue was subject to column chromatography on silica gel (0–1% Et_2O in hexane) to afford the title compound as a colorless oil (990 mg, 81%).

IR (ATR, film) υ_{max} 2978, 2361, 2342, 1329, 1141, 966, 847 cm^{-1}.

^{1}H NMR (500 MHz, CDCl$_3$) δ 7.45 (d, *J* = 7.2 Hz, 1 H), 7.14 (m, 3 H), 6.96 (m, 1 H), 5.64–5.57 (m, 1 H), 5.26 (d, *J* = 10.9 Hz, 1 H), 2.33 (s, 2 H), 1.22 (s, 12 H).

^{13}C NMR (126 MHz, CDCl$_3$) δ 136.8, 136.7, 135.5 , 130.2 , 127.9, 125.7 , 125.6 , 115.3, 83.6, 24.9, 18.4. Due to quadrupolar relaxation, the boron-bearing carbon was identified by the HSQC-crosspeak.

^{11}B NMR (96 MHz, CDCl$_3$) δ 33.16.

HRMS (EI) Exact mass calculated for $C_{15}H_{21}BO_2$ $[M]^{+\bullet}$ *m/z* = 244.1629; found 244.1631.

4,4,5,5-tetramethyl-2-(3-vinylbenzyl)-1,3,2-dioxaborolane (16)

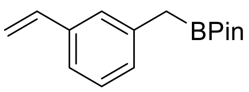

Prepared according to General Procedure C using 3-vinylphenylboronic acid (29.6 mg, 0.20 mmol). The crude residue (69% ^{1}H NMR yield) was subject to column chromatography on silica gel (0.5% Et_2O in hexane) to afford the title compound as a colorless oil (35.2 mg, 72%).

IR (ATR, film) υ_{max} 2978, 2926, 2361, 2342, 1329, 1141, 989, 669 cm^{-1}.

^{1}H NMR (500 MHz, $CDCl_3$) δ 7.23 (br s, 1 H), 7.23–7.15 (m, 2 H), 7.09 (dt, *J* = 6.9, 2.0 Hz, 1 H), 6.68 (dd, *J* = 17.6, 10.9 Hz, 1 H), 5.72 (dd, *J* = 17.6, 1.0 Hz, 1 H), 5.20 (dd, *J* = 10.9, 1.0 Hz, 1 H), 2.29 (s, 2 H), 1.23 (s, 12 H).

^{13}C NMR (126 MHz, $CDCl_3$) δ 139.0, 137.6, 137.3, 128.7, 128.6, 127.1, 123.0, 113.5, 83.6, 24.9, 20.0. Due to quadrupolar relaxation, the boron-bearing carbon was identified by the HSQC-crosspeak.

^{11}B NMR (96 MHz, $CDCl_3$) δ 33.32.

HRMS (EI) Exact mass calculated for $C_{15}H_{21}BO_2$ $[M]^{+\bullet}$ *m/z* = 244.1629; found 244.1634.

4,4,5,5-tetramethyl-2-(naphthalen-1-ylmethyl)-1,3,2-dioxaborolane (17)

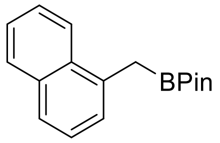

Prepared according to General Procedure C using 1-naphthylboronic acid (34.4 mg, 0.20 mmol). The crude residue (91% ^{1}H NMR yield) was subject to column chromatography on silica gel (0–5% Et_2O in hexane) to afford the title compound as a colorless oil (46.7 mg, 87%).

^{1}H NMR (500 MHz, $CDCl_3$) δ 8.03 (dd, *J* = 8.2, 1.6 Hz, 1 H), 7.84 (dd, *J* = 7.6, 1.7 Hz, 1 H), 7.70–7.65 (m, 1 H), 7.48 (m, 2 H), 7.42–7.33 (m, 2 H), 2.71 (s, 2 H), 1.21 (s, 12 H).

^{13}C NMR (126 MHz, $CDCl_3$) δ 135.7, 133.9, 132.6, 128.6, 126.6, 125.9, 125.7, 125.5, 125.5, 124.6, 83.7, 24.8 . The boron-bearing carbon was not observed due to quadrupolar relaxation.

^{11}B NMR (96 MHz, $CDCl_3$) δ 33.28.

The spectral data were consistent with that of the literature.[16]

2-(4-methoxybenzyl)-4,4,5,5-tetramethyl-1,3,2-dioxaborolane (18)

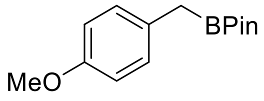

Prepared according to General Procedure C using 4-methoxybenzeneboronic acid (30.4 mg, 0.20 mmol). The crude residue (92% ^{1}H NMR yield) was subject to column chromatography on silica gel (0.5–1% Et_2O in hexane) to afford the title compound as a white solid (43.0 mg, 87%).

^{1}H NMR (500 MHz, $CDCl_3$) δ 7.13–7.06 (m, 2 H), 6.82–6.76 (m, 2 H), 3.77 (s, 3 H), 2.22 (s, 2 H), 1.23 (s, 12 H).

^{13}C NMR (126 MHz, $CDCl_3$) δ 157.2, 130.6, 129.9, 113.9, 83.5, 55.3, 24.9, 19.1. The boron-bearing carbon at 19.1 ppm was weak due to quadrupolar relaxation and confirmed by the HSQC-crosspeak.

^{11}B NMR (96 MHz, $CDCl_3$) δ 33.47.

The spectral data were consistent with that of the literature.[10]

2-(3-methoxybenzyl)-4,4,5,5-tetramethyl-1,3,2-dioxaborolane (19)

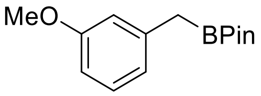

Prepared according to General Procedure C using 3-methoxybenzeneboronic acid (30.4 mg, 0.20 mmol). The crude residue (86% ^{1}H NMR yield) was subject to column chromatography on silica gel (0.5–1% Et_2O in hexane) to afford the title compound as a colorless residue (40.7 mg, 82%).

^{1}H NMR (500 MHz, $CDCl_3$) δ 7.15 (t, J = 7.8 Hz, 1 H), 6.80–6.73 (m, 2 H), 6.68 (ddd, J = 8.2, 2.6, 0.9 Hz, 1 H), 3.78 (s, 3 H), 2.27 (s, 2 H), 1.24 (s, 12 H).

^{13}C NMR (126 MHz, $CDCl_3$) δ 159.7, 140.3, 129.3, 121.7, 114.8, 110.6, 83.6, 55.2, 24.9, 20.2. Due to quadrupolar relaxation, the boron-bearing carbon was identified by the HSQC-crosspeak.

^{11}B NMR (96 MHz, $CDCl_3$) δ 33.13.

The spectral data were consistent with that of the literature.[14]

2-(2-methoxybenzyl)-4,4,5,5-tetramethyl-1,3,2-dioxaborolane (20)

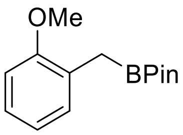

Prepared according to General Procedure C using 2-methoxybenzeneboronic acid (24.0 mg, 0.14 mmol). The crude residue (91% ^{1}H NMR yield) was subject to column chromatography on silica gel (1–4% Et_2O in hexane) to afford the title compound as a white solid (41.0 mg, 83%).

^{1}H NMR (500 MHz, CDCl$_3$) δ 7.15 (app. t, J = 7.4 Hz, 2 H) 6.88 (td, J = 7.4, 1.2 Hz, 1 H), 6.84–6.79 (m, 1 H), 3.82 (s, 3 H), 2.21 (s, 2 H), 1.26 (s, 12 H).

^{13}C NMR (126 MHz, CDCl$_3$) δ 157.2, 130.5, 128.0, 126.3, 120.5, 109.7, 83.1, 55 .1, 24.7, 15.3 (br.). The boron-bearing carbon was weak and confirmed by the HSQC-crosspeak due to quadrupolar relaxation.

^{11}B NMR (96 MHz, CDCl$_3$) δ 33.57.

The spectral data were consistent with that of the literature.[13]

2-(2,4-dimethoxybenzyl)-4,4,5,5-tetramethyl-1,3,2-dioxaborolane (21)

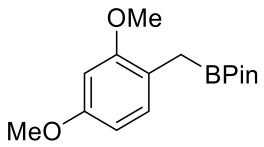

Prepared according to General Procedure C using 2,4-dimethoxybenzeneboronic acid (36.4 mg, 0.20 mmol). The crude residue (86% ^{1}H NMR yield) was subject to column chromatography on silica gel (1–4% Et_2O in hexane) to afford the title compound as a white solid (45.1 mg, 81%).

IR (ATR, film) υ_{max} 2361, 2342, 1558, 1506, 1207, 1146 cm^{-1}.

^{1}H NMR (500 MHz, CDCl$_3$) δ 7.06–7.00 (m, 1 H), 6.41 (d, J = 2.5 Hz, 1 H), 6.39 (dd, J = 8.1, 2.5 Hz, 1 H), 3.77 (br. s 6 H), 2.10 (s, 2 H), 1.23 (s, 12 H).

^{13}C NMR (126 MHz, CDCl$_3$) δ 158.7, 158.0, 130.5, 120.3, 103.9, 98.4, 83.2, 55.4, 55.2, 24.8, 14.3. Due to quadrupolar relaxation, the boron-bearing carbon was identified by the HSQC-crosspeak.

^{11}B NMR (96 MHz, CDCl$_3$) δ 34.34.

HRMS (EI) Exact mass calculated for $C_{15}H_{23}BO_4$ $[M]^{+\bullet}$ m/z = 294.1633; found 294.1643.

4,4,5,5-tetramethyl-2-(3,4,5-trimethoxybenzyl)-1,3,2-dioxaborolane (22)

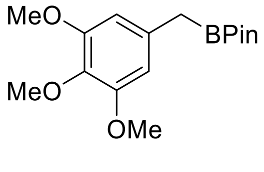

Prepared according to General Procedure C using 3,4,5-trimethoxyphenylboronic acid (42.4 mg, 0.20 mmol). The crude residue (69% ^{1}H NMR yield) was subject to column chromatography on silica gel (0.5–4% Et_2O in hexane) to afford the title compound as a colorless residue (35.7 mg, 58%).

IR (ATR, film) υ_{max} 2976, 2930, 2361, 2342, 1587, 1506, 1456, 1323, 1123, 1009, 962, 847 cm^{-1}.

^{1}H NMR (500 MHz, CDCl$_3$) δ 6.44 (s, 2 H), 3.86 (s, 6 H), 3.84 (s, 3 H), 2.26 (s, 2 H), 1.28 (s, 12 H).

^{13}C NMR (126 MHz, CDCl$_3$) δ 153.1, 135.5, 134.3, 106.0, 83.6, 61.0, 56.1, 24.9, 20.4. Due to quadrupolar relaxation, the boron-bearing carbon was identified by the HSQC-crosspeak.

^{11}B NMR (96 MHz, CDCl$_3$) δ 33.32.

HRMS (EI) Exact mass calculated for $C_{16}H_{25}BO_5$ $[M+O]^{+\bullet}$ *m/z* = 324.1734; found 324.1749.

4,4,5,5-tetramethyl-2-(4-(methylthio)benzyl)-1,3,2-dioxaborolane (23)

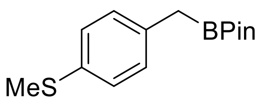

Prepared according to General Procedure C using 4-(methylthio)benzeneboronic acid (33.6 mg, 0.20 mmol). The crude residue (61% ^{1}H NMR yield) was subject to column chromatography on silica gel (0–2% Et_2O in hexane) to afford the title compound as a colorless oil (26.4 mg, 50%).

^{1}H NMR (500 MHz, CDCl$_3$) δ 7.19–7.14 (m, 2 H), 7.14–7.08 (m, 2 H), 2.45 (s, 3 H), 2.25 (s, 2 H), 1.23 (s, 12 H).

^{13}C NMR (126 MHz, CDCl$_3$) δ 135.9, 134.0, 129.6, 127.4, 83.5, 24.7, 19.4, 16.5. Due to quadrupolar relaxation, the boron-bearing carbon was identified by the HSQC-crosspeak.

^{11}B NMR (96 MHz, CDCl$_3$) δ 33.26.

The spectral data were consistent with that of the literature.[17]

trimethyl(4-((4,4,5,5-tetramethyl-1,3,2-dioxaborolan-2-yl)methyl)phenyl)silane (24)

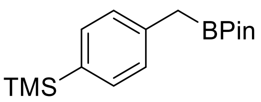

Prepared according to General Procedure C using 4-(trimethylsilyl)phenylboronic acid (38.8 mg, 0.20 mmol). The crude residue (87% ^{1}H NMR yield) was subject to column chromatography on silica gel (0.2% Et_2O in hexane) to afford the title compound as a colorless oil (48.8 mg, 84%).

^{1}H NMR (500 MHz, CDCl$_3$) δ 7.43–7.33 (m, 2 H), 7.22–7.14 (m, 2 H), 2.29 (s, 2 H), 1.24 (s, 12 H), 0.24 (s, 9 H).

^{13}C NMR (126 MHz, CDCl$_3$) δ 139.5, 136.1, 133.5, 128.7, 83.6, 24.9, 20.1, −0.9. Due to quadrupolar relaxation, the boron-bearing carbon was identified by the HSQC-crosspeak.

^{11}B NMR (96 MHz, CDCl$_3$) δ 33.60.

The spectral data were consistent with that of the literature.[12]

4,4,5,5-tetramethyl-2-(2-(methylthio)benzyl)-1,3,2-dioxaborolane (25)

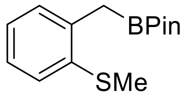

Prepared according to General Procedure C using 2-(methylthio)phenylboronic acid (33.6 mg, 0.20 mmol). The crude residue (81% ^{1}H NMR yield) was subject to column chromatography on silica gel (0.5% Et_2O in hexane) to afford the title compound as a colorless residue (38.9 mg, 74%).

^{1}H NMR (500 MHz, $CDCl_3$) δ 7.22 (dd, *J* = 7.8, 1.4 Hz, 1 H), 7.14 (m, 2 H), 7.07 (m, 1 H), 2.44 (s, 3H), 2.37 (s, 2 H), 1.24 (s, 12 H).

^{13}C NMR (126 MHz, $CDCl_3$) δ 138.4, 137.0, 129.8, 126.8, 126.1, 125.5, 83.6, 24.9, 16.7. The boron-bearing carbon was not observed due to quadrupolar relaxation.

^{11}B NMR (96 MHz, $CDCl_3$) δ 33.05.

The spectral data were consistent with that of the literature.[18]

2-(3-fluoro-4-methoxybenzyl)-4,4,5,5-tetramethyl-1,3,2-dioxaborolane (26)

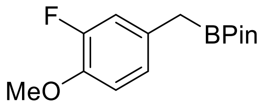

Prepared according to General Procedure C using 3-fluoro-4-methoxyphenylboronic acid (34.0 mg, 0.20 mmol). The crude residue (80% ^{1}H NMR yield) was subject to column chromatography on silica gel (0.5% Et_2O in hexane) to afford the title compound as a colorless residue (41.5 mg, 78%).

IR (ATR, film) υ_{max} 2979, 2927, 2355, 1514, 1348, 1328, 1269, 1142, 846 cm^{-1}.

^{1}H NMR (500 MHz, $CDCl_3$) δ 6.93 (dd, *J* = 12.6, 2.0 Hz, 1 H), 6.86 (dd, *J* = 8.5, 2.0 Hz, 1 H), 6.83 (t, *J* = 8.2 Hz, 1 H), 3.84 (s, 3 H), 2.21 (s, 2 H), 1.23 (s, 12 H).

^{13}C NMR (126 MHz, $CDCl_3$) δ 152.3 (d, $^{1}J_{CF}$ = 244.4 Hz), 145.1 (d, $^{2}J_{CF}$ = 10.9 Hz), 131.8 (d, $^{3}J_{CF}$ = 6.7 Hz), 124.5 (d, $^{3}J_{CF}$ = 3.5 Hz), 116.9 (d, $^{2}J_{CF}$ = 18.1 Hz), 113.6 (d, $^{4}J_{CF}$ = 2.4 Hz), 83.7, 56.5, 24.9, 19.0. Due to quadrupolar relaxation, the boron-bearing carbon was identified by the HSQC-crosspeak.

^{11}B NMR (96 MHz, $CDCl_3$) δ 32.94.

^{19}F NMR (470 MHz, $CDCl_3$) δ −136.07.

HRMS (EI) Exact mass calculated for $C_{14}H_{20}BFO_3$ $[M]^{+\bullet}$ *m*/*z* = 266.14840; found 266.1245.

2-(4-fluoro-3-methoxybenzyl)-4,4,5,5-tetramethyl-1,3,2-dioxaborolane (27)

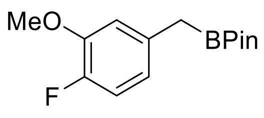

Prepared according to General Procedure C using 4-fluoro-3-methoxyphenylboronic acid (34.0 mg, 0.20 mmol). The crude residue (78% ^{1}H NMR yield) was subject to column chromatography on silica gel (0.5% Et_2O in hexane) to afford the title compound as a colorless residue (38.9 mg, 73%).

IR (ATR, film) υ_{max} 2978, 2933, 2361, 2342, 1608, 1516, 1329, 1142, 1036, 847 cm^{-1}.

^{1}H NMR (500 MHz, $CDCl_3$) δ 6.92 (dd, *J* = 11.4, 8.2 Hz, 1 H), 6.79 (dd, *J* = 8.3, 2.1 Hz, 1 H), 6.68 (ddd, *J* = 8.2, 4.3, 2.1 Hz, 1 H), 3.86 (s, 3 H), 2.24 (s, 2 H), 1.24 (s, 12 H).

^{13}C NMR (126 MHz, $CDCl_3$) δ 150.5 (d, $^{1}J_{CF}$ = 241.6 Hz), 147.2 (d, $^{2}J_{CF}$ = 10.8 Hz), 134.9 (d, $^{4}J_{CF}$ = 3.8 Hz) 121.1 (d, $^{3}J_{CF}$ = 6.4 Hz), 115.7 (d, $^{2}J_{CF}$ = 17.9 Hz), 114.3 (d, $^{3}J_{CF}$ = 1.5 Hz), 83.7, 56.2, 24.9, 19.9. Due to quadrupolar relaxation, the boron-bearing carbon was identified by the HSQC-crosspeak.

^{11}B NMR (96 MHz, $CDCl_3$) δ 32.99.

^{19}F NMR (470 MHz, $CDCl_3$) δ −141.34.

HRMS (ESI+) Exact mass calculated for $C_{14}H_{21}BFO_3$ $[M+H]^+$ *m/z* = 267.1562; found 267.1571.

4,4,5,5-tetramethyl-2-(4-(trifluoromethoxy)benzyl)-1,3,2-dioxaborolane (28)

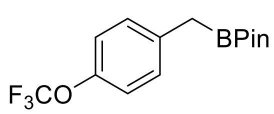

Prepared according to General Procedure C using 4-(trifluoromethoxy)phenylboronic acid (36.4 mg, 0.20 mmol). The crude residue (74% ^{1}H NMR yield) was subject to column chromatography on silica gel (0.5% Et_2O in hexane) to afford the title compound as a white solid (39.3 mg, 65%).

^{1}H NMR (500 MHz, $CDCl_3$) δ 7.23–7.15 (m, 2 H), 7.11–7.04 (m, 2 H), 2.29 (s, 2 H), 1.24 (s, 12 H).

^{13}C NMR (126 MHz, $CDCl_3$) δ 146.9, 137.6, 130.3, 121.0, 120.7 (q, $^{1}J_{CF}$ = 256.24 Hz) 83.7, 24.9 19.5. Due to quadrupolar relaxation, the boron-bearing carbon was identified by the HSQC-crosspeak.

^{11}B NMR (96 MHz, $CDCl_3$) δ 33.03.

^{19}F NMR (470 MHz, $CDCl_3$) δ −57.91.

The spectral data were consistent with that of the literature.[14]

2-(4-fluorobenzyl)-4,4,5,5-tetramethyl-1,3,2-dioxaborolane (29)

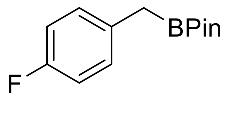

Prepared according to General Procedure C using 4-fluorobenzeneboronic acid (28.0 mg, 0.20 mmol). The crude residue (81% ^{1}H NMR yield) was subject to column chromatography on silica gel (0–5% Et_2O in hexane) to afford the title compound as a colorless oil (32.6 mg, 69%).

^{1}H NMR (500 MHz, $CDCl_3$) δ 7.17–7.08 (m, 2 H), 6.97–6.88 (m, 2 H), 2.25 (s, 2 H), 1.23 (s, 12 H).

^{13}C NMR (126 MHz, $CDCl_3$) δ 160.9 (d, $^1J_{CF}$ = 242.0 Hz), 134.2 (d, $^4J_{CF}$ = 3.4 Hz), 130.3 (d, $^3J_{CF}$ = 7.6 Hz) 115.1 (d, $^2J_{CF}$ = 21.2 Hz), 83.6, 24.9. The boron-bearing carbon was not observed due to quadrupolar relaxation.

^{11}B NMR (96 MHz, $CDCl_3$) δ 32.69.

^{19}F NMR (376 MHz, $CDCl_3$) δ −119.37.

The spectral data were consistent with that of the literature.[10]

2-(3-fluorobenzyl)-4,4,5,5-tetramethyl-1,3,2-dioxaborolane (30)

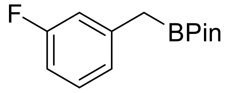

Prepared according to General Procedure C using 3-fluorobenzeneboronic acid (28.0 mg, 0.20 mmol). Anisole (0.8 mL, 0.25 M) were used as the solvent and the reaction time was 6 h. The crude residue (60% ^{1}H NMR yield) was subject to column chromatography on silica gel (0–10% Et_2O in hexane) to afford the title compound as a colorless residue (22.0 mg, 47%).

^{1}H NMR (500 MHz, $CDCl_3$) δ 7.18 (td, J = 7.9, 6.2 Hz, 1 H), 6.97–6.92 (m, 1 H, C_6), 6.90 (dt, J = 10.3, 2.1 Hz, 1 H), 6.81 (td, J = 8.4, 2.4 Hz, 1 H), 2.29 (s, 2 H), 1.24 (s, 12 H).

^{13}C NMR (126 MHz, $CDCl_3$) δ 163.0 (d, $^1J_{CF}$ = 244.3 Hz), 141.4 (d, $^3J_{CF}$ = 7.7 Hz), 129.6 (d, $^3J_{CF}$ = 8.6 Hz), 124.8 (d, $^4J_{CF}$ = 2.7 Hz), 116.0 (d, $^2J_{CF}$ = 21.0 Hz), 111.9 (d, $^2J_{CF}$ = 21.1 Hz), 83.7, 20.0, 24.9. Due to quadrupolar relaxation, the boron-bearing carbon was identified by the HSQC-crosspeak.

^{11}B NMR (96 MHz, $CDCl_3$) δ 32.77.

^{19}F NMR (470 MHz, $CDCl_3$) δ −114.32. Decomposition was observed during characterization as noted previously in the literature, resulting in a peak at −57.91 ppm. Ratio ($F_{product}$:$F_{decomp.}$) = 84:16.

The spectral data were consistent with that of the literature.[16]

2-(2-chloro-6-propoxybenzyl)-4,4,5,5-tetramethyl-1,3,2-dioxaborolane (31)

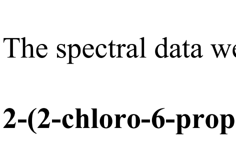

Prepared according to General Procedure C using 2-chloro,6-propoxyphenylboronic acid (42.9 mg, 0.20 mmol). The crude residue (72% ^{1}H NMR yield) was subject to column chromatography on silica gel (0.5% Et_2O in hexane) to afford the title compound as a colorless oil (40.0 mg, 64%).

IR (ATR, film) υ_{max} 2976, 2930, 2361, 2342, 1348, 1327, 1144, 982, 662 cm^{-1}.

^{1}H NMR (500 MHz, $CDCl_3$) δ 7.10 (d, *J* = 2.6 Hz, 1 H), 7.04 (dd, *J* = 8.6, 2.7 Hz, 1 H), 6.69 (d, *J* = 8.6 Hz, 1 H), 3.87 (t, *J* = 6.6 Hz, 2 H), 2.18 (s, 2 H), 1.80 (m, 2 H), 1.23 (s, 12 H), 1.04 (t, *J* = 7.4 Hz, 3 H).

^{13}C NMR (126 MHz, $CDCl_3$) δ 155.5, 130.3, 130.2, 125.9, 125.0, 112.0, 83.4, 69.9, 24.9, 22.9, 15.0, 10.7. Due to quadrupolar relaxation, the boron-bearing carbon was identified by the HSQC-crosspeak.

^{11}B NMR (96 MHz, $CDCl_3$) δ 33.60.

HRMS (EI) Exact mass calculated for $C_{16}H_{24}BClO_4$ $[M+O]^{+\bullet}$ *m/z* = 326.1451; 326.1462.

2-(2-fluorobenzyl)-4,4,5,5-tetramethyl-1,3,2-dioxaborolane (32)

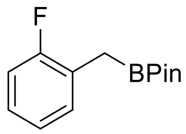

Prepared according to General Procedure C using 3-fluorobenzeneboronic acid (28.0 mg, 0.20 mmol). The crude residue (60% ^{1}H NMR yield) was subject to column chromatography on silica gel (0.5–1.5% Et_2O in hexane) to afford the title compound in 74:26 ratio mixture with the undesired starting material pinacol ester (20.7 mg, 32% yield of desired product). Loss of yield was caused by partial decomposition during chromatography. Attempts to completely isolate the desired product from the starting material speciated pinacol ester (0.1% Et_2O in hexane) resulted in full decomposition upon concentration, so the mixture was characterized.

^{1}H NMR (500 MHz, $CDCl_3$) δ 7.22–7.16 (m, 1 H), 7.14–7.07 (m, 1 H), 7.04–6.95 (m, 2 H), 2.26 (s, 2 H), 1.24 (s, 12 H).

^{13}C NMR (126 MHz, $CDCl_3$) δ 161.2 (d, $^{1}J_{CF}$ = 243.6 Hz), 131.5 (d, $^{3}J_{CF}$ = 4.9 Hz), 126.8 (d, $^{3}J_{CF}$ = 7.9 Hz), 126.2 (d, $^{2}J_{CF}$ = 16.9 Hz), 124.0 (d, $^{4}J_{CF}$ = 3.6 Hz), 115.0 (d, $^{2}J_{CF}$ = 22.1 Hz), 83.7, 24.8, 13.1. Due to quadrupolar relaxation, the boron-bearing carbon was identified by the HSQC-crosspeak.

^{11}B NMR (96 MHz, $CDCl_3$) 33.40.

^{19}F NMR (470 MHz, $CDCl_3$) δ −116.87. BPin ester of starting material δ_F = 102.59 ppm.

The spectral data were consistent with that of the literature.[10]

2-(4-chlorobenzyl)-4,4,5,5-tetramethyl-1,3,2-dioxaborolane (33)

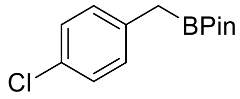

Prepared according to General Procedure C using 4-chlorobenzeneboronic acid (31.3 mg, 0.20 mmol) at 45 °C for 36 h. The crude residue (65% ^{1}H NMR yield) was subject to column chromatography on silica gel (0.5% Et_2O in hexane) to afford the title compound as a colorless residue (29.3 mg, 58%).

^{1}H NMR (500 MHz, $CDCl_3$) 7.23–7.16 (m, 2 H), 7.14–7.07 (m, 2 H), 2.25 (s, 2 H), 1.23 (s, 12 H).

^{13}C NMR (126 MHz, $CDCl_3$) δ 137.3, 130.7, 130.4, 128.4, 83.7, 24.9, 19.6. Due to quadrupolar relaxation, the boron-bearing carbon has a weak signal-to-noise ratio and was confirmed by the HSQC-crosspeak.

^{11}B NMR (96 MHz, $CDCl_3$) δ 32.87.

The spectral data were consistent with that of the literature.[16]

2-(2-chlorobenzyl)-4,4,5,5-tetramethyl-1,3,2-dioxaborolane (34)

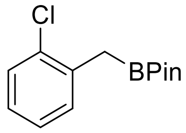

Prepared according to General Procedure C using 2-chlorobenzeneboronic acid (31.3 mg, 0.20 mmol) at 45 °C for 36 h. The crude residue (45% ^{1}H NMR yield) was subject to column chromatography on silica gel (0.5% Et_2O in hexane) to afford the title compound as a white solid (21.6 mg, 43%).

^{1}H NMR (500 MHz, $CDCl_3$) δ 7.31 (dd, J = 7.8, 1.4 Hz, 1 H), 7.22 (dd, J = 7.5, 1.8 Hz, 1 H), 7.14 (td, J = 7.4, 1.4 Hz, 1 H), 7.08 (td, J = 7.6, 1.8 Hz, 1 H), 2.38 (s, 2 H), 1.24 (s, 12 H).

^{13}C NMR (126 MHz, $CDCl_3$) δ 137.7, 134.0, 131.0, 129.2, 126.8, 126.6, 83.7, 24.9, 19.1. Due to quadrupolar relaxation, the boron-bearing carbon was identified by the HSQC-crosspeak.

^{11}B NMR (96 MHz, $CDCl_3$) δ 33.09.

The spectral data were consistent with that of the literature.[16]

2-(3-chlorobenzyl)-4,4,5,5-tetramethyl-1,3,2-dioxaborolane (35)

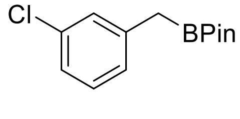

Prepared according to General Procedure C using 3-chlorobenzeneboronic acid (31.3 mg, 0.20 mmol) at 45 °C for 36 h. The crude residue (54% ^{1}H NMR yield) was subject to column chromatography on silica gel (0.5% Et_2O in hexane) to afford the title compound as a white solid (21.2 mg, 42%).

^{1}H NMR (500 MHz, $CDCl_3$) δ 7.16–7.18 (m, 1 H), 7.15 (br. d, *J* = 7.7 Hz, 1 H), 7.10 (dt, *J* = 8.1, 1.7 Hz, 1 H), 7.06 (ddt, *J* = 7.5, 1.7, 0.8 Hz, 1 H), 2.27 (s, 2 H), 1.23 (s, 12 H).

^{13}C NMR (126 MHz, $CDCl_3$) δ 140.9, 134.0, 129.6, 129.2, 127.3, 125.2, 83.8, 24.9, 20.0 (br.). Due to quadrupolar relaxation, the boron-bearing carbon has a weak signal-to-noise ratio and was confirmed by the HSQC-crosspeak.

^{11}B NMR (96 MHz, $CDCl_3$) δ 32.83.

The spectral data were consistent with that of the literature.[14]

2-(2-bromo-6-methoxybenzyl)-4,4,5,5-tetramethyl-1,3,2-dioxaborolane (36)

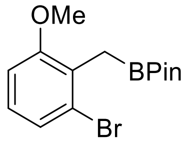

Prepared according to General Procedure C using 2-bromo,6-methoxyphenylboronic acid (46.2 mg, 0.20 mmol). The crude residue (29% ^{1}H NMR yield) was subject to column chromatography on silica gel (0.2–2% Et_2O in hexane) to afford the title compound as a colorless oil (20.9 mg, 32%).

IR (ATR, film) υ_{max} 2978, 2928, 2359, 2342, 1489, 1348, 1242, 669 cm^{-1} .

^{1}H NMR (500 MHz, $CDCl_3$) δ 7.28–7.18 (m, 2 H), 6.66 (d, *J* = 8.6 Hz, 1 H), 3.77 (s, 3 H), 2.14 (s, 2 H), 1.23 (s, 12 H).

^{13}C NMR (126 MHz, $CDCl_3$) δ 156.5, 133.2 , 130.6, 129.0 , 112.8, 111.4 , 83.4, 55.4, 24.8, 15.3. Due to quadrupolar relaxation, the boron-bearing carbon was identified by the HSQC-crosspeak.

^{11}B NMR (96 MHz, $CDCl_3$) δ 33.52.

HRMS (EI) Exact mass calculated for $C_{14}H_{21}BBrO_3$ $[M+H]^{+\bullet}$ *m/z* = 327.0762; found 327.0762.

methyl 4-((4,4,5,5-tetramethyl-1,3,2-dioxaborolan-2-yl)methyl)benzoate (37)

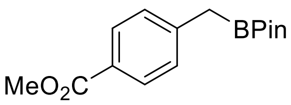

Prepared according to General Procedure C using 4-methoxycarbonylphenyl boronic acid (36.0 mg, 0.20 mmol): The crude residue (54% ^{1}H NMR yield) was subject to column chromatography on silica gel (0.5–1% Et_2O in hexane) to afford the title compound as a white solid (16.0 mg, 29%).

^{1}H NMR (500 MHz, $CDCl_3$) δ 7.95–7.86 (m, 2 H), 7.25–7.22 (m, 2 H), 3.89 (s, 3 H), 2.35 (s, 2 H), 1.22 (s, 12 H).

^{13}C NMR (126 MHz, $CDCl_3$) δ 167.5, 144.9, 129.8, 129.1, 127.0, 83.8, 52.1, 24.8, 20.4. The boron-bearing carbon was not observed due to quadrupolar relaxation.

^{11}B NMR (96 MHz, $CDCl_3$) δ 33.30.

The spectral data were consistent with that of the literature.[14]

4,4,5,5-tetramethyl-2-((phenyl-*d*5)methyl)-1,3,2-dioxaborolane (38)

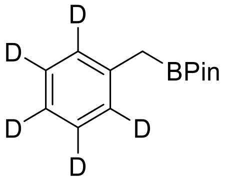

Prepared according to General Procedure C using phenylboronic acid-d_5 (25.3 mg, 0.20 mmol). The crude residue (81% ^{1}H NMR yield) was subject to column chromatography on silica gel (0.5–1% Et_2O in hexane) to afford the title compound as a colorless oil (26.8 mg, 64%).

^{1}H NMR (500 MHz, $CDCl_3$) δ 2.29 (s, 2 H), 1.23 (s, 12 H).

^{2}H NMR (77 MHz, $CHCl_3$ unlocked) δ 7.17–7.29 (br. m, 5 D).

^{13}C NMR (126 MHz, $CDCl_3$) δ 138.6, 128.7 (t, $^1J_{CD}$ = 23.8 Hz), 127.9 (t, $^1J_{CD}$ = 23.3 Hz), 124.4 (t, $^1J_{CD}$ = 23.8 Hz), 83.5, 24.9. The boron-bearing carbon was not observed due to quadrupolar relaxation.

^{11}B NMR (96 MHz, $CDCl_3$) δ 33.36.

The spectral data were consistent with that of the literature.[19]

4,4,5,5-tetramethyl-2-(thiophen-3-ylmethyl)-1,3,2-dioxaborolane (39)

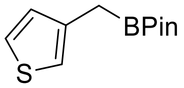

Prepared according to General Procedure C using thiophene-3-boronic acid (25.6 mg, 0.20 mmol). The crude residue (74% ^{1}H NMR yield) was subject to column chromatography on silica gel (0.5–1% Et_2O in hexane) to afford the title compound as a colorless residue (19.4 mg, 43%).

^{1}H NMR (500 MHz, $CDCl_3$) δ 7.20 (dd, J = 4.9, 3.0 Hz, 1 H), 6.98–6.91 (m, 2 H), 2.28 (s, 2 H), 1.25 (s, 12 H).

^{13}C NMR (126 MHz, $CDCl_3$) δ 137.7, 129.6, 124.9, 120.2, 83.6, 24.9, 13.9. Due to quadrupolar relaxation, the boron-bearing carbon was identified by the HSQC-crosspeak.

^{11}B NMR (96 MHz, $CDCl_3$) δ 32.94.

The spectral data were consistent with that of the literature.[17]

4,4,5,5-tetramethyl-2-((3-methylthiophen-2-yl)methyl)-1,3,2-dioxaborolane (40)

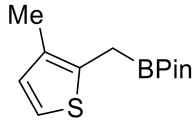

Prepared according to General Procedure C using 5-methylthiophene-2-boronic acid (28.4 mg, 0.20 mmol). The crude residue (78% ^{1}H NMR yield) was subject to column chromatography on silica gel (0.5–1% Et_2O in hexane) to afford the title compound as a colorless residue (33.8 mg, 71%).

IR (ATR, film) υ_{max} 2978, 2361, 2342, 1363, 1333, 1141, 996, 669 cm^{-1}.

^{1}H NMR (500 MHz, $CDCl_3$) d 6.56 (d, J = 3.3 Hz, 1 H), 6.51 (d, J = 3.4 Hz, 1 H), 2.40 (br. s, 5 H), 1.27 (s, 12 H).

^{13}C NMR (126 MHz, $CDCl_3$) δ 138.0, 137.1, 125.0, 124.7, 83.8, 24.9, 15.4. The boron-bearing carbon could not be identified due to quadrupolar relaxation and overlapping signals with Me-thiophenyl by HSQC.

^{11}B NMR (96 MHz, $CDCl_3$) δ 34.23. Boric acid (δ_B = 22.53 ppm) can be seen in the spectrum caused by partial leaching from B-SiO_2 during column chromatography.

HRMS (EI) Exact mass calculated for $C_{12}H_{19}BO_2S$ $[M]^{+\bullet}$ m/z = 238.1193; found 238.10325.

2-(benzo[b]thiophen-3-ylmethyl)-4,4,5,5-tetramethyl-1,3,2-dioxaborolane (41)

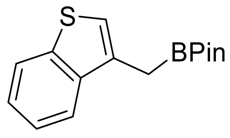

Prepared according to General Procedure C using benzo[b]thien-3-ylboronic acid (35.6 mg, 0.2 mmol). The crude residue (80% ^{1}H NMR yield) was subject to column chromatography on silica gel (0.5–1% Et_2O in hexane) to afford the title compound as a pale yellow oil (37.0 mg, 67%).

IR (ATR, film) υ_{max} 2361, 2342, 1146, 763, 669, 656, 650 cm^{-1}.

^{1}H NMR (500 MHz, $CDCl_3$) δ 7.83 (d, *J* = 7.9 Hz, 1 H), 7.75 (d, *J* = 7.9 Hz, 1 H), 7.43–7.33 (m, 1 H), 7.36–7.29 (m, 1 H), 7.19 (s, 1 H), 2.44 (s, 2 H), 1.25 (s, 12 H).

^{13}C NMR (126 MHz, $CDCl_3$) δ 140.3, 139.9, 132.4, 124.0, 123.7, 122.8, 122.1, 121.3, 83.8, 24.9, 12.2. Due to quadrupolar relaxation, the boron-bearing carbon was identified by the HSQC-crosspeak.

^{11}B NMR (96 MHz, $CDCl_3$) δ 32.98.

HRMS (EI) Exact mass calculated for $C_{15}H_{19}BO_2S$ $[M]^{+\bullet}$ *m/z* = 274.1193; found 227.1200.

2-methoxy-3-((4,4,5,5-tetramethyl-1,3,2-dioxaborolan-2-yl)methyl)pyridine (42)

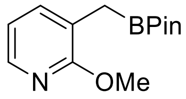

Prepared according to General Procedure C using 2-methoxy-3-pyridinylboronic acid (30.6 mg, 0.20 mmol). The crude residue (83% ^{1}H NMR yield) was subject to column chromatography on silica gel (CH_2Cl_2) to afford the title compound as a pale yellow oil (42.8 mg, 86%).

The substrate was also prepared according to General Procedure D from 2-methoxy-3-pyridinylboronic acid (382 mg, 2.50 mmol). The crude residue was subject to column chromatography on silica gel (CH_2Cl_2) to afford the title compound as a colorless oil (573 mg, 92%).

IR (ATR, film) υ_{max} 2978, 2359, 1587, 1252, 1414, 1350, 1142, 1109, 846, 773 cm^{-1}.

^{1}H NMR (500 MHz, $CDCl_3$) δ 7.96 (dd, *J* = 5.1, 1.9 Hz, 1 H), 7.38 (ddt, *J* = 7.1, 1.8, 0.8 Hz, 1 H), 6.77 (dd, *J* = 7.1, 5.1 Hz, 1 H), 3.92 (s, 3 H), 2.13 (s, 2 H), 1.24 (s, 12 H).

^{13}C NMR (126 MHz, $CDCl_3$) δ 162.2, 143.5, 138.3, 122.5, 117.0, 83.5, 53.2, 24.8, 14.9. Due to quadrupolar relaxation, the boron-bearing carbon was identified by the HSQC-crosspeak.

^{11}B NMR (96 MHz, $CDCl_3$) δ 33.54.

HRMS (EI) Exact mass calculated for $C_{13}H_{20}BNO_3$ $[M]^{+\bullet}$ *m/z* = 249.1531; found 249.15372.

4,4,5,5-tetramethyl-2-((3-methylfuran-2-yl)methyl)-1,3,2-dioxaborolane (43)

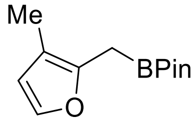

Prepared according to General Procedure C using 5-methylfuran-2-boronic acid (25.2 mg, 0.20 mmol). The crude residue (58% ^{1}H NMR yield) was subject to column chromatography on silica gel (0.5% Et_2O in hexane) to afford the title compound as a colorless residue (21.8 mg, 49%).

IR (ATR, film) υ_{max} 3210, 2922, 2361, 2342, 1458, 764, 750, 669 cm^{-1}.

^{1}H NMR (500 MHz, CDCl$_3$) δ 5.92 (dd, J = 2.9, 1.3 Hz, 1 H), 5.82 (dd, J = 2.9, 1.3 Hz, 1 H), 2.25 (s, 2 H), 2.23 (s, 3 H), 1.27 (s, 12 H).

^{13}C NMR (126 MHz, CDCl$_3$) 150.5, 150.1, 106.2, 106.1, 83.8, 24.9, 13.7, 11.8. Due to quadrupolar relaxation, the boron-bearing carbon was identified by the HSQC-crosspeak.

^{11}B NMR (96 MHz, CDCl$_3$) δ 32.70. Boric acid (δ_B = 22.47 ppm) can be seen in the spectrum caused by partial leaching from B-SiO_2 during column chromatography.

HRMS (CI) Exact mass calculated for $C_{12}H_{20}BO_3$ $[M+H]^+$ m/z = 223.1506; found 223.1508.

2-(furan-3-ylmethyl)-4,4,5,5-tetramethyl-1,3,2-dioxaborolane (44)

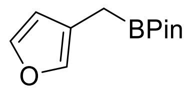

Prepared according to General Procedure C using furan-3-boronic acid (22.4 mg, 0.20 mmol). The crude residue (46% ^{1}H NMR yield) was subject to column chromatography on silica gel (0.5% Et_2O in hexane) to afford the title compound as a colorless residue (17.5 mg, 42%).

IR (ATR, film) υ_{max} 2920, 2851, 2359, 2342, 1261, 750, 669 cm^{-1}.

^{1}H NMR (500 MHz, CDCl$_3$) δ 7.32 (t, J = 1.7 Hz, 1 H), 7.27–7.24 (br m, 1 H), 6.30–6.24 (m, 1 H), 2.01 (s, 2 H), 1.26 (s, 12 H).

^{13}C NMR (126 MHz, CDCl$_3$) δ 142.5, 139.4, 120.2, 112.6, 83.6, 24.9. The boron-bearing carbon was not observed due to quadrupolar relaxation.

^{11}B NMR (96 MHz, CDCl$_3$) δ 33.13.

HRMS (CI) Exact mass calculated for $C_{11}H_{18}BO_3$ $[M+H]^+$ m/z = 209.1344; found 209.1343.

3,5-dimethyl-4-((4,4,5,5-tetramethyl-1,3,2-dioxaborolan-2-yl)methyl)isoxazole (45)

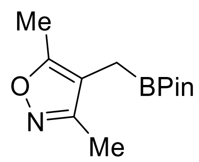

Prepared according to General Procedure C using 3,5-dimethylisoxazole-4-boronic acid (28.2 mg, 0.20 mmol). The crude residue (59% ^{1}H NMR yield) was subject to column chromatography on silica gel (1–5% Et_2O in hexane) to afford the title compound as a colorless residue (25.8 mg, 54%).

IR (ATR, film) υ_{max} 2978, 2924, 2361, 2342, 1352, 1167, 1141, 669 cm^{-1}.

^{1}H NMR (500 MHz, CDCl$_3$) δ 2.28 (s, 3 H), 2.19 (s, 3 H), 1.81 (s, 2 H), 1.23 (s, 12 H).

^{13}C NMR (126 MHz, CDCl$_3$) δ 163.9, 160.0, 109.9, 83.8, 24.9, 11.2, 10.5. The boron-bearing carbon was not observed due to quadrupolar relaxation.

^{11}B NMR (96 MHz, CDCl$_3$) δ 32.76.

HRMS (EI) Exact mass calculated for $C_{12}H_{20}BNO_3$ $[M+]^{+\bullet}$ *m/z* = 237.1531; found 237.11267.

diphenylmethane (52)

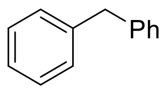

To an oven-dried microwave vial fitted with a stir bar was added dried K_3PO_4 (1.27 g, 6.0 mmol, 3.0 equiv), then Compound **5** (131 mg, 6.0 mmol, 3.0 equiv) then Pd(dppf)Cl_2 (16.3 mg, 0.02 mmol, 1.0 mol%) and the vial capped and purged thrice with Ar prior to the addtion of PhMe (8.00 mL), bromobenzene (0.21 mL, 2.0 mmol, 1.00 equiv) then water (1.8 mL, 100 mmol, 50 equiv) and the reaction mixture stirred at 90 °C for 24 h. The vial was decaleapped and the crude reaction mixture was filtered through a short pad of Celite, eluting with CH_2Cl_2, and concentrated at reduced pressure to afford the crude (>99% ^{1}H NMR yield) which was subject to column chromatography on silica gel (2% EtOAc in hexane) to afford the desired product as a white solid (276 mg, 82%).

^{1}H NMR (500 MHz, CDCl$_3$) δ 7.34–7.20 (m, 4 H), 7.20–7.23 (m, 6 H), 4.00 (s, 2 H).

^{13}C NMR (126 MHz, CDCl$_3$) δ 141.3, 129.1, 128.6, 126.2, 42.1.

The spectral data were consistent with the literature.[20]

4-benzyl-1,1'-biphenyl (53)

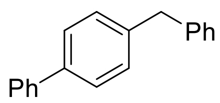

Prepared according to General Procedure E using Compound **11** (221 mg, 0.75 mmol, 3.0 equiv), Pd(dppf)Cl_2 (1.8 mg, 0.025 mmol, 1.0 mol%) and bromobenzene (26.3 μL, 0.25 mmol, 1.00 equiv). The crude (97% ^{1}H NMR yield) was subject to column chromatography on silica gel (hexane) to afford the desired product as a white solid (59.1 mg, 97%).

^{1}H NMR (500 MHz, CDCl$_3$) δ 7.59–7.55 (m, 2 H), 7.55–7.50 (m, 2 H), 7.43 (dd, *J* = 8.5, 7.0 Hz, 2 H), 7.35–7.29 (m, 3 H), 7.29–7.19 (m, 5 H), 4.03 (s, 2 H).

^{13}C NMR (126 MHz, CDCl$_3$) δ 141.1, 140.4, 139.2, 129.5, 129.1, 129.0, 128.9, 128.7, 127.4, 127.2, 127.2, 126.3, 41.7.

The spectral data were consistent with the literature.[21]

bifonazole (54)

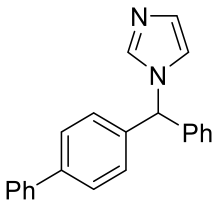

To an oven dried flask fitted with a stir bar that was evacuated and backfilled under Ar was added Compound **53** (44.0 mg, 0.18 mmol, 1.00 equiv), NBS (32.0 mg, 0.18 mmol, 1.00 equiv), AIBN (29.6 mg, 0.18 mmol, 1.00 equiv) and the flask purged three times under Ar. The flask was quickly transferred to a reflux condenser fitted with a septum and Ar balloon prior to the addition of CCl_4 (2.25 mL) and the solution was refluxed for 1 h. After cooling to rt, the reaction mixture was diluted in hexane (3 mL) and insoluble solids filtered off, washing with hexane then the liquor concentrated at reduced pressure. To the flask containing the crude yellow solid was added dry K_2CO_3 (87.1 mg, 0.63 mmol, 3.5 equiv), imidazole (123 mg, 1.80 mmol, 10 equiv) and MeCN (4.50 mL). The reaction mixture was refluxed for 2 hours. After cooling to rt, the reaction was diluted in MeCN (5 mL), filtered through Celite, and the liquor concentrated at reduced pressure. The crude (88% ^{1}H NMR yield) was subject to column chromatography on silica gel (20–40% EtOAc in hexane) to afford the desired product as a white solid (49.1 mg, 88%).

^{1}H NMR (500 MHz, $CDCl_3$) δ 7.58 (d, *J* = 7.8 Hz, 4 H), 7.48–7.42 (m, 3 H), 7.42–7.32 (m, 4 H), 7.20–7.10 (m, 5 H), 6.90 (br. s, 1 H), 6.57 (br. s, 1 H).

^{13}C NMR (126 MHz, $CDCl_3$) δ 141.4, 140.3, 139.2, 138.2, 137.6, 129.6, 129.0, 129.0, 128.6, 128.6, 128.2, 127.76, 127.7, 127.2, 119.5, 64.9.

The spectral data were consistent with the literature.[22]

cyclizine (55)

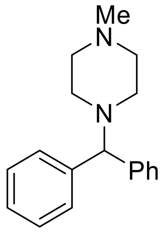

To an oven dried flask fitted with a stir bar that was evacuated and backfilled under Ar was added Compound **55** (33.6 mg, 0.18 mmol, 1.00 equiv), NBS (32.0 mg, 0.18 mmol, 1.00 equiv), AIBN (29.6 mg, 0.18 mmol, 1.00 equiv) and the flask purged three times under Ar. The flask was quickly transferred to a reflux condenser fitted with a septum and Ar balloon prior to the addition of CCl_4

(2.25 mL) and the solution was refluxed for 1 h. After cooling to rt, the reaction mixture was diluted in hexane 3 mL and insoluble solids filtered off, washing with hexane (2 x) then the liquor concentrated at reduced pressure. To the flask containing the crude yellow solid was added dry K_2CO_3 (87.1 mg, 0.63 mmol, 3.5 equiv), *N*-methylpiperazine (0.20 mL, 2.0 mmol, 10 equiv) and MeCN (4.50 mL). The reaction mixture was refluxed for 2 hours. After cooling to rt, the reaction was diluted in MeCN 5 mL, filtered through Celite, and the liquor concentrated at reduced pressure. The crude (78% ^{1}H NMR yield) was subject to column chromatography on silica gel (CH_2Cl_2) to afford the desired product as a colorless residue (40.0 mg, 75%).

^{1}H NMR (500 MHz, $CDCl_3$) δ 7.43–7.38 (m, 4 H), 7.29–7.23 (m, 4 H), 7.22–7.13 (m, 2 H), 4.23 (s, 1 H), 2.73–2.12 (br, 11 H).

^{13}C NMR (126 MHz, $CDCl_3$) δ 142.6, 128.7, 127.9, 127.2, 76.2, 55.2, 51.3, 45.5.

The spectral data were consistent with the literature.[23]

4,4,5,5-tetramethyl-2-((4'-(trifluoromethoxy)-[1,1'-biphenyl]-4-yl)methyl)-1,3,2-dioxaborolane (56)

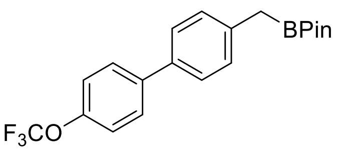

An oven-dried 1 mL microwave vial was charged with K_3PO_4 (127 mg, 0.600 mmol, 3.00 equiv), $Pd(OAc)_2$ (1.8 mg, 8.0 µmol, 4.0 mol%), SPhos (6.6 mg, 16 µmol, 8.0 mol%) and the vial capped and purged thrice with Ar prior to the addition of PhMe (0.80 mL), then Compound **S6** (50.5 mg, 0.200 mmol, 1.00 equiv), then **Compound 33** (60.5 mg, 0.210 mmol, 1.10 equiv) then water (36 µL, 2.0 mmol, 10 equiv). The reaction mixture was stirred at 90 °C for 4 h then cooled to rt and the vial decapped then the reaction mixture diluted in CH_2Cl_2, filtered through a short pad of silica gel, eluting with CH_2Cl_2, and concentrated at reduced pressure. The crude (96% ^{1}H NMR yield)was subject to column chromatography on B-SiO_2 (0.1–2% Et_2O in hexane) to afford the desired product as a white residue (70.3 mg, 93%).

IR (film) ν 2361, 2342, 1258, 1143, 669 cm^{-1}.

^{1}H NMR (500 MHz, $CDCl_3$) δ 7.68–7.50 (m, 2 H), 7.47–7.41 (m, 2 H), 7.32–7.23 (m, 4 H), 2.34 (s, 2 H), 1.25 (s, 12 H).

^{13}C NMR (126 MHz, $CDCl_3$) δ 148.5, 140.2, 138.5, 136.5, 129.7, 128.3, 127.5, 127.1, 121.30, 120.67 (q, $^1J_{CF}$ = 256.9 Hz), 83.7, 24.9. The boron-bearing carbon was not observed due to quadrupolar relaxation.

^{11}B NMR (96 MHz, $CDCl_3$) δ 32.20.

^{19}F NMR (470 MHz, $CDCl_3$) δ −57.81.

HRMS (ESI+) Exact mass calcd. for $C_{20}H_{23}BF_3O_3$ $[M+H]^{+\bullet}$ m/z = 379.16869; found 379.1683.

1-chloro-4-(4-methoxybenzyl)benzene (57)

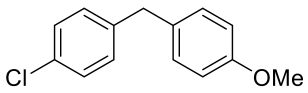

Prepared according to General Procedure E using 4-bromoanisole (25.1 µL, 0.20 mmol, 1.00 equiv) and Compound **33** (152 mg, 0.60 mmol). The crude (87% 1H NMR yield) was subject to column chromatography on silica gel (1–5% Et_2O in hexane) to afford the desired product as a colourless residue (41 mg, 88%).

^{1}H NMR (500 MHz, $CDCl_3$) δ 7.26–7.21 (m, 2 H), 7.13–7.04 (m, 4 H), 6.87–6.79 (m, 2 H), 3.89 (s, 2 H), 3.79 (s, 3 H).

^{13}C NMR (126 MHz, $CDCl_3$) δ 158.2, 140.2, 132.8, 131.9, 130.3, 123.0, 128.7, 114.1, 55.4, 40.5.

The spectral data were consistent with the literature.[24]

potassium bromomethyltetrafluoroborate (64)

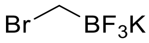

A modified version of General procedure A. A solution of dibromoethane (1.75 mL, 25.0 mmol, 1.2 equiv) and triisopropyl borate (5.31 mL, 23.0 mmol, 1.10 equiv) in dry THF (75 mL) was cooled to −80 °C. *n*BuLi 2.32 M in hexanes (9.05 mL, 21.0 mmol, 1.00 equiv) was added dropwise over 1 h. After the addition, the solution was stirred vigorously for 1 h at −78 °C then stirred at rt for 1 h. The reaction mixture was cooled back to −80 °C whereupon KHF_2 (4.08 g, 52.9 mmol, 2.50 equiv) was added in a single portion. The septum was returned and water (15 mL) was added dropwise over 10 min. The reaction was warmed to 25 °C, then vigorously stirred for 30 min. The cloudy suspension was transferred to a single necked flask, rinsing with THF, concentrated, then left on high vacuum overnight to afford an off-white solid. The crude was dissolved an excess of acetone and the insoluble salts (excess KHF_2, KF) filtered off, then the liquor was concentrated to retrieve a white solid. The desired compound was purified by dissolving in a minimum volume of hot dried acetone (*ca.* 45 °C), then precipitating with ice-cold diethyl ether. The desired compound was filtered off then dried on the high vacuum to afford a fine white solid which was stored in the freezer in the absence of light (2.25 g, 53%).

^{1}H NMR (500 MHz, $(CD_3)_2CO$) δ 2.28–2.09 (m, 2 H).

^{13}C NMR (126 MHz, $(CD_3)_2CO$) δ no peaks. The boron-bearing carbon was not observed due to quadrupolar relaxation.

^{11}B NMR (96 MHz, $(CD_3)_2CO$) δ 2.59 (q, $^{1}J_{BF}$ = 50.7 Hz).

^{19}F NMR (470 MHz, $(CD_3)_2CO$) δ −145.69 (q, $^{1}J_{FB}$ = 49.4 Hz).

The spectral data were consistent with the literature.

2-(bromomethyl)-1,3,2-dioxaborolane (65)

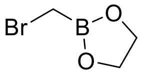

Prepared according to General Procedure A using in THF (40 mL), dibromomethane (0.84 mL, 12.0 mmol, 1.20 equiv), triisopropyl borate (2.42 mL, 10.5 mmol, 1.05 equiv) and *n*BuLi 11 M in hexanes (0.91 mL, 10.0 mmol, 1.00 equiv). Following the addition procedure, methanesulfonic acid (0.65 mL, 10.0 mmol, 1.00 equiv) and ethyleneglycol (0.56 mL, 10.0 mmol, 1.00 equiv) were used. Following workup, the desired product was purified by vacuum distillation (33–35 °C, 1.2 mbar) and was stored in the freezer in the absence of light as a colourless liquid (290 mg, 17%). *Note: partial loss of material occurred during vacuum distilation.*

IR (film) ν 2357, 2342, 1418, 1325, 764, 750, 669 cm^{-1}.

^{1}H NMR (500 MHz, $CDCl_3$) δ 4.31 (s, 4 H), 2.66 (s, 2 H).

^{13}C NMR (126 MHz, $CDCl_3$) δ 66.5. The boron-bearing carbon was not observed due to quadrupolar relaxation.

^{11}B NMR (96 MHz, $CDCl_3$) δ 32.01.

HRMS (CI) Exact mass calcd. for $C_3H_6BO_2$ $[M-Br]^+$ *m/z* = 85.0455; found 85.0458.

diisopropyl (4*R*, 5*R*)-2-(bromomethyl)-1,3,2-dioxaborolane-4,5-dicarboxylate (66)

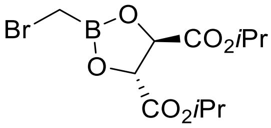

Prepared according to General Procedure A using in THF (30 mL), dibromomethane (1.77 mL, 25.2 mmol, 1.20 equiv), triisopropyl borate (5.33 mL, 23.1 mmol, 1.10 equiv) and *n*BuLi 1.98 M in hexanes (10.6 mL, 21.0 mmol, 1.00 equiv). Following the addition procedure, methanesulfonic acid (1.36 mL, 21.0 mmol, 1.00 equiv) and (+)-diisopropyl *L*-tartrate, 99% *e.e.* (4.92 g in 10 mL dry THF, 21.0 mmol, 1.00 equiv) were used. Following workup, the desired product was purified by vacuum distillation (6–7 mbar, 150–152 °C) afforded the product as a straw-coloured liquid (3.71 g, 50%).

$[\alpha]_D^{20}$ = – 41.0 ° (c = 1.0, $CHCl_3$).

ν_{max} (film) 2361, 2342, 1736, 1271, 1103, 669 cm^{-1}.

^{1}H NMR (500 MHz, $CDCl_3$) δ 5.13 (hept, J = 6.3 Hz, 2 H), 4.87 (s, 2 H), 2.75 (s, 2 H), 1.31 (d, J = 6.3 Hz, 12 H).

^{13}C NMR (126 MHz, $CDCl_3$) δ 168.6, 78.3, 70.5, 21.8, 21.7. The boron-bearing carbon was not observed due to quadrupolar relaxation.

^{11}B NMR (96 MHz, $CDCl_3$) δ 32.40.

HRMS (EI) exact mass calcd. for $[M]^+$ ($C_{11}H_{18}{}^{11}B^{79}BrO_6$) requires m/z 336.03743; found m/z 336.03668.

2-(bromomethyl)-1,3,2-dioxaborinane (67)

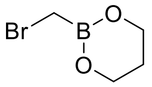

Prepared according to General Procedure A using in THF (40 mL), dibromomethane (0.84 mL, 12.0 mmol, 1.20 equiv), triisopropyl borate (2.42 mL, 10.5 mmol, 1.05 equiv) and *n*BuLi 11 M in hexanes (0.91 mL, 10.0 mmol, 1.00 equiv). Following the addition procedure, methanesulfonic acid (0.65 mL, 10.0 mmol, 1.00 equiv) and 1,3 propanediol (0.72 mL, 10.0 mmol, 1.00 equiv) were used. Following workup, the desired product was purified by vacuum distillation (39–41 °C, 1.2 mbar) and was stored in the freezer in the absence of light as a colourless liquid (862 mg, 48%). *Note: partial loss of material occurred during vacuum distilation.*

IR (film) ν 2953, 2899, 2361, 2342, 1431, 1279, 1155, 932, 758 cm^{-1}.

^{1}H NMR (500 MHz, $CDCl_3$) δ 4.06 (t, J = 5.5 Hz, 4 H), 2.52 (s, 2 H), 1.98 (p, J = 5.5 Hz, 2 H).

^{13}C NMR (126 MHz, $CDCl_3$) δ 62.5, 27.1. The boron-bearing carbon was not observed due to quadrupolar relaxation.

^{11}B NMR (96 MHz, $CDCl_3$) δ 27.80.

HRMS (EI) Exact mass calcd. for $C_4H_8BBrO_2$ $[M]^{+\bullet}$ m/z = 177.9780; found 177.9801.

2-(bromomethyl)-5,5-dimethyl-1,3,2-dioxaborinane (68)

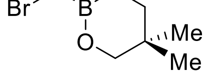

Prepared according to General Procedure A using in THF (40 mL), dibromomethane (0.84 mL, 12.0 mmol, 1.20 equiv), triisopropyl borate (2.42 mL, 10.5 mmol, 1.05 equiv) and *n*BuLi 11 M in hexanes (0.91 mL, 10.0 mmol, 1.00 equiv). Following the addition procedure, methanesulfonic acid (0.65 mL, 10.0 mmol, 1.00 equiv) and neopentylglycol (1.04 g, 10.0 mmol, 1.00 equiv) were used. Following workup, the desired product was purified by vacuum distillation (56–58 °C, 1.2 mbar) and was stored in the freezer in the absence of light as a colourless liquid (1.68 g, 81%).

IR (film) ν 2963, 2361, 2342, 1479, 1292,1260, 1146, 1007, 814, 745, 654, 546 cm^{-1}.

^{1}H NMR (500 MHz, $CDCl_3$) δ 3.65 (s, 4 H), 2.53 (s, 2 H), 0.97 (s, 6 H).

^{13}C NMR (126 MHz, $CDCl_3$) δ 72.6, 31.9, 21.8. The boron-bearing carbon was not observed due to quadrupolar relaxation.

^{11}B NMR (96 MHz, $CDCl_3$) δ 27.39.

HRMS (EI) Exact mass calcd. for $C_6H_{12}BBrO_2$ $[M]^{+\bullet}$ *m/z* = 206.0108; found 206.0103.

2-(bromomethyl)-4,6-dimethyl-1,3,2-dioxaborinane (69)

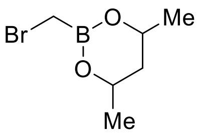

Prepared according to General Procedure A using in THF (40 mL), dibromomethane (0.84 mL, 12.0 mmol, 1.20 equiv), triisopropyl borate (2.42 mL, 10.5 mmol, 1.05 equiv) and *n*BuLi 11 M in hexanes (0.91 mL, 10.0 mmol, 1.00 equiv). Following the addition procedure, methanesulfonic acid (0.65 mL, 10.0 mmol, 1.00 equiv) and 2,4-pentanediol (2.19 mL, 10.0 mmol, 1.00 equiv) were used. Following workup, the desired product was purified by vacuum distillation (45–47 °C, 4 mbar) and was stored in the freezer in the absence of light as a colourless liquid (1.59 g, 77%, *syn*:*anti* 53:47).

IR (film) ν 2361, 2342, 1418, 1290, 1152, 750, 679 cm^{-1}.

syn

^{1}H NMR (500 MHz, $CDCl_3$) δ 4.31 (dp, *J* = 12.3, 5.9 Hz, 2 H), 2.52 (s, 2 H), 1.74–1.77 (m, 2 H), 1.27 (d, *J* = 6.2 Hz, 6 H).

^{13}C NMR (126 MHz, $CDCl_3$) δ 68.9, 42.3, 23.0. The boron-bearing carbon was not observed due to quadrupolar relaxation.

anti

^{1}H NMR (500 MHz, $CDCl_3$) δ 4.15–4.21 (m, 2 H), 2.52 (s, 2 H), 1.89–1.95 (m, 2 H), 1.30 (d, *J* = 6.4 Hz, 6 H).

^{13}C NMR (126 MHz, $CDCl_3$) δ. 65.4, 39.0, 22.5. The boron-bearing carbon was not observed due to quadrupolar relaxation.

^{11}B NMR (96 MHz, $CDCl_3$) δ 27.62.

HRMS (EI) Exact mass calcd. for $C_6H_{12}BBrO_2$ $[M]^{+\bullet}$ *m*/*z* = 206.0114; found 206.0116.

***N*-methyliminodiacetyl (bromomethyl)boronate (70)**

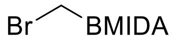

Prepared according to General procedure A with some modifications. To a flame-dried three-necked flask backfilled under an atmosphere of Ar was added dry THF (13 mL), dibromomethane (0.84 mL, 12.0 mmol, 1.20 equiv), then triisopropyl borate (2.54 mL, 11.0 mmol, 1.10 equiv). The solution was cooled to <–80 °C then *n*BuLi 2.25 M in hexanes (4.45 mL, 10.0 mmol, 1.00 equiv) was added dropwise at <–80 °C. The resulting mixture was stirred for 1 h at −80 °C, and then the cooling bath was removed and stirred for 2 h at rt. In a separate three-necked flame-dried flask cooled under Ar was added *N*-methyliminodiacetic acid (2.57 g, 10.5 mmol, 1.05 equiv) and dry DMSO (20 mL), and the solution heated to 50 °C (mantle temperature). The flask was fitted with two dropping funnels and a distillation apparatus. To each of the dropping funnels were added dry PhMe (45 mL) and the reaction mixture, transferred by cannula over a positive pressure of Ar, which was added to the DMSO solution dropwise and the mixture kept at gentle reflux. The distilled THF was replaced by the simultaneous dropwise addition of PhMe (see Figure S2). The previous reaction vessel was rinsed with dry PhMe (10 mL) which was added through the reaction mixture dropping funnel as a single portion. The distillation apparatus was replaced with a reflux condenser. The reaction mixture was heated to vigorous reflux (150 °C mantle temperature) and stirred for 18 h. Volatile solvents were removed at reduced pressure to afford a red liquid which was diluted in a 3:2 mixture EtOAc / acetone (120 mL) and the organics were washed with water (6 x 50 mL) and brine (3 x 50 mL), dried (Na_2SO_4), then concentrated at reduced pressure to afford an orange sticky residue. The residue was dissolved in a minimum volume of hot EtOAc and precipitated from cold Et_2O, which was filtered and washed with hexane, to afford the product as an off-white solid (838 mg, 34%). Triisopropylborate is a contaminent which could not be removed by aqueous workup or high vacuum.

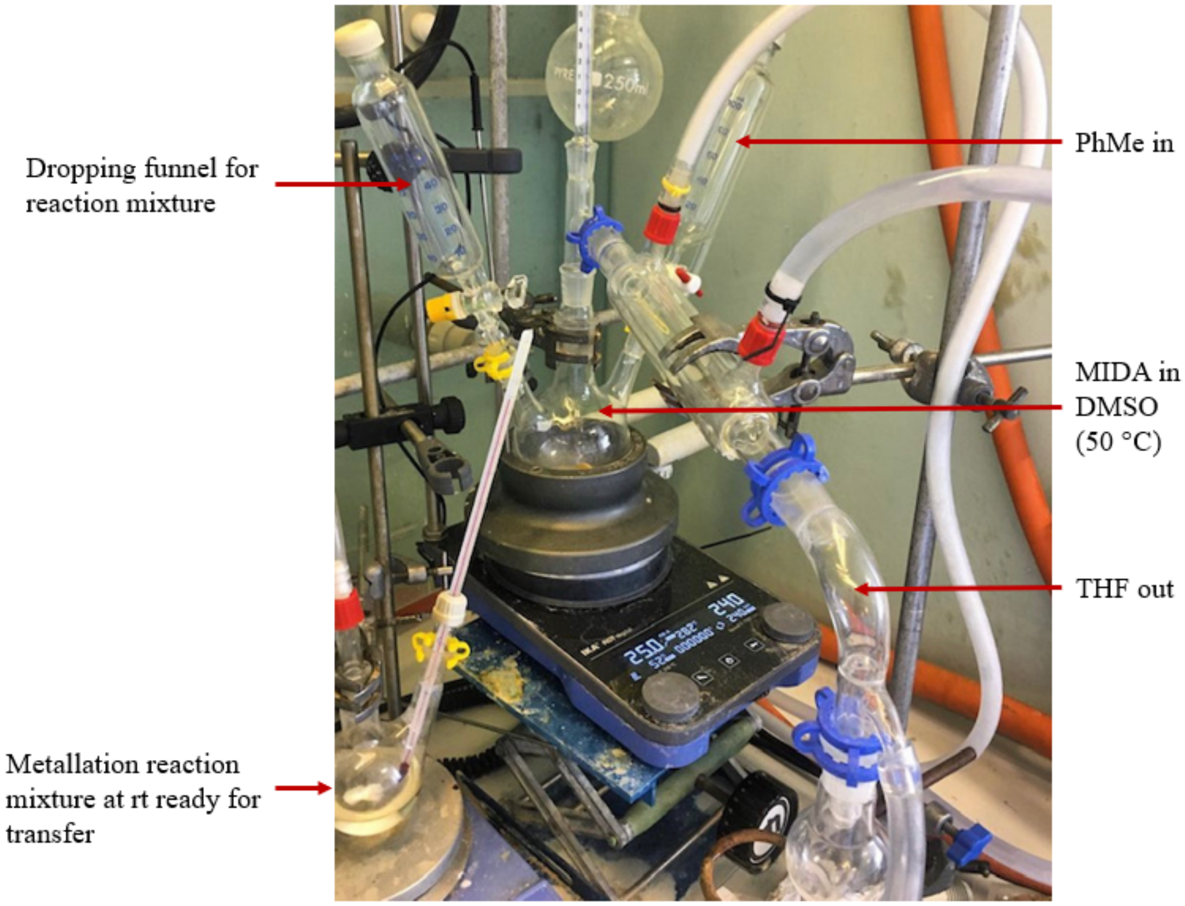

Figure S2: Setup for inert solvent switch and esterification with MIDA to prepare Compound **70**.

ν_{max} (solid) 1773, 1748, 1292, 1113, 1055, 1026 cm^{-1}.

^{1}H NMR (500 MHz, $(CD_3)_2SO$) δ 4.21 (d, J = 16.9 Hz, 2 H), 3.86 (d, J = 16.9 Hz, 2 H,), 2.97 (s, 2 H, H_1), 2.94 (s, 3 H).

^{13}C NMR (126 MHz, $(CD_3)_2SO$) δ 169.0, 61.8, 45.5. The boron-bearing carbon was not observed due to quadrupolar relaxation.

^{11}B NMR (96 MHz, $(CD_3)_2SO$) δ 11.28.

HRMS (ESI) exact mass calcd. for $[M{-}Br]^+$ ($C_6H_9{}^{11}BNO_4$) requires m/z 170.06191; found m/z 170.06260.

2-(1-chloroethyl)-4,4,5,5-tetramethyl-1,3,2-dioxaborolane (71)

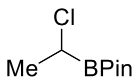

To a flame dried three necked flasked equipped with a stir bar, cooled under Ar and fitted with a thermometer and septum, was added a solution of dry CH_2Cl_2 (3.53 mL, 55.0 mmol, 1.10

equiv) in THF (100 mL) which was cooled to −110 °C in an absolute EtOH/ liq. N_2 slush bath. *n*BuLi 11.0 M in hexane (4.55 mL, 50.0 mmol, 1.00 equiv) was precooled to 0 °C and lightly shaken before used to ensure homogeneity, then added dropwise by hand over 15 min. *The needle containing the nBuLi was placed such that the solution ran down the side of the flask before making contact with the reaction mixture to ensure adequate cooling*. After 30 min stirring at −110 °C,* a solution of MeBPin (7.46 g, 52.5 mmol, 1.05 equiv) in THF (5 mL), precooled to ~ −80 °C (acetone/ liq. N_2 slush bath), was added to the centre of the reaction flask in one portion. The solution was stirred for a further 15 min at −110 °C, then the cooling bath was removed and the reaction mixture was stirred at rt overnight (16 h). The solution was concentrated at reduced pressure then resuspended in pentane 150 mL and insoluble salts were filtered off, washing the LiCl filter cake with pentane (2 x 40 mL). The solution was concentrated at reduced pressure to afford a cream which was subject to vacuum distillation (68–70 °C, 11 mbar, lit. 94–95 °C, 15.3 mbar[5]) to afford a colourless liquid (6.47 g, 68%).

*Technical note: After the *n*BuLi addition and stirring for 30 min the reaction mixture should remain colourless or straw yellow. A darker yellow or orange colour can be indicative of carbenoid degradation and the reaction must be restarted. Formation of the lithiated carbenoid can be tested by removing an aliquot of the reaction mixture and watching the carbenoid solution flashing from colourless to black in the syringe barrel, which is discarded.

^{1}H NMR (500 MHz, $CDCl_3$) δ 3.51 (q, J = 7.6 Hz, 1 H), 1.54 (d, J = 7.6 Hz, 3 H), 1.29 (s, 12 H).

^{13}C NMR (126 MHz, $CDCl_3$) δ 84.5, 24.7, 20.5. The boron-bearing carbon was not observed due to quadrupolar relaxation.

^{11}B NMR (96 MHz, $CDCl_3$) δ 31.51.

These data were consistent with that of the literature.[25]

2-(1-bromoethyl)-4,4,5,5-tetramethyl-1,3,2-dioxaborolane (72)

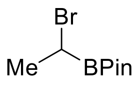

To a round bottomed flask wrapped in foil to exclude light and equipped with a stir bar was added compound **71** (952 mg, 5.00 mmol, 1.00 equiv) which was dissolved in Et_2O (10.0 mL) prior to the addition of LiBr (2.17 g, 25.0 mmol, 5.00 equiv) in one portion. The flask was stirred at rt for 24 h, then diluted in Et_2O and insoluble salts filtered off and concentrated at reduced pressure, then resuspended in hexane 15 mL and any remaining salts filtered off, then concentrated again to afford the deisred product as a pale yellow oil (975 mg, 83%) which was stored in the freezer in the absence of light.

IR (film) ν 2980, 2361, 2342, 1371, 1167, 1134, 970, 841, 669 cm^{-1}.

^{1}H NMR (500 MHz, $CDCl_3$) δ 3.43 (q, *J* = 7.6 Hz, 1 H), 1.70 (d, *J* = 7.5 Hz, 3 H), 1.28 (d, *J* = 1.3 Hz, 12 H).

^{13}C NMR (126 MHz, $CDCl_3$) δ 84.4, 24.6 (2 x s), 20.7. Two observed pinacolate signals are consistent with *syn* and *anti* BPin CH_3 wrt C−Br bond. The boron-bearing carbon was not observed due to quadrupolar relaxation.

^{11}B NMR (96 MHz, $CDCl_3$) δ 31.37.

HRMS (EI) Exact mass calcd. for $C_8H_{16}BBrO_2$ $[M]^{+\bullet}$ *m/z* = 234.0421; found 234.0414.

2-(1-iodoethyl)-4,4,5,5-tetramethyl-1,3,2-dioxaborolane (73)

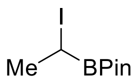

To a round bottom flask wrapped in tin foil and equipped with a stir bar was added NaI (3.75 g, 25.0 mmol, 5.00 equiv) and acetone (10.0 mL) and the reaction stirred at rt for ~3 min until compound **71** (952 mg, 5.00 mmol, 1.00 equiv) was added in one portion. The reaction mixture was stirred at rt with the fume cupboard light turned off for 16 h then the reaction mixture filtered and concentrated at reduced pressure (30 °C, flask wrapped in tin foil) to afford a yellow solid which was triturated with CH_2Cl_2 (25 mL) and the solid filtered off, washing with CH_2Cl_2 (10 mL portions until the filtered solid turned white, excess NaI). The liquor was concentrated at reduced pressure to afford the desired product as a yellow oil (958 mg, 68%).

IR (film) ν 2978, 2361, 2342, 1366, 1331, 1144, 1105, 970, 841, 669 cm^{-1}.

^{1}H NMR (500 MHz, $CDCl_3$) δ 3.36 (q, *J* = 7.5 Hz, 1 H), 1.84 (d, *J* = 7.5 Hz, 3 H), 1.27 (s, 12 H).

^{13}C NMR (126 MHz, $CDCl_3$) δ 84.1, 24.5, 24.4, 21.8. Two observed pinacolate signals are consistent with *syn* and *anti* BPin CH_3 wrt C−I bond. The boron-bearing carbon was not observed due to quadrupolar relaxation.

^{11}B NMR (96 MHz, $CDCl_3$) δ 32.07.

HRMS (EI) Exact mass calcd. for $C_8H_{16}BIO_2$ $[M]^{+\bullet}$ *m/z* = 282.0283; found 282.0292.

2-(2-bromopropan-2-yl)-4,4,5,5-tetramethyl-1,3,2-dioxaborolane (74)

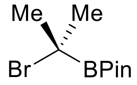

Based on a procedure by Morken and coworkers.[26] A flame-dried two necked flask cooled under an atmosphere of Ar was fitted with a septum and an aq. $NaHCO_3$ scrubber then charged with $CHCl_3$ (5.0 mL) and isopropylBPin (0.57 mL, 3.00 mmol, 1.00 equiv). Br_2 (0. 39 mL, 7.5 mmol, 2.5 equiv), was added in a single portion at rt. The flask was stirred at rt for 16 h then diluted in CH_2Cl_2 10 mL, transferred to a single necked flask and concentrated at reduced pressure. The oil was filtered through a pipette pad of silica gel (3 cm), eluting with CH_2Cl_2 10 mL, then concentrated to afford the desired product as a pale orange oil (412 mg, 55%) which was stored in the freezer in the absence of light. Yield loss was attributed to the instability of the product to silica gel which were used to remove residual Br_2. The compound is known but full characterization was incomplete. Partial degradation of the material occurred during NMR characterization.[26]

IR (film) ν 1728, 1462, 1364, 1364, 1140, 854 cm^{-1}.

^{1}H NMR (500 MHz, $CDCl_3$) δ 1.77 (s, 6 H), 1.28 (s, 12 H).

^{13}C NMR (126 MHz, $CDCl_3$) δ 84.3, 30.4, 24.5. The boron-bearing carbon was not observed due to quadrupolar relaxation.

^{11}B NMR (96 MHz, $CDCl_3$) δ 31.48.

HRMS (CI) Exact mass calcd. for $C_9H_{18}BBrO_2$ $[M]^+$ *m/z* = 249.0656; found 249.0648.

4.2 Summary of attempted syntheses of 1,2-bromoethyl BPin

Several general routes were attempted to prepare $BrCH_2CH_2BPin$.

Attempted Matteson-type homologations using halomethyllithiums and **2-Br** or **2-I** returned mixtures of the starting material or the opposite halide (halomethyl:haloethyl typically 80:20, **Scheme S5a**).

A transition-metal free borylation using conditions reported by Mo and coworkers was attempted but afforded complex mixtures (**Scheme S5b**).[27]

Hydrobromination of vinylBPin by addition of HBr in a variety of solvents and loadings of radical initiator failed. Hydrometalation using Schwartz reagent and subsequent interception with bromine resulted in degradation (**Scheme S5c**).

Finally, we prepared vinyl bromide fresh and attempted to hydroborate (**Scheme S5d**). This was the route selected by a commercial supplier who were also unable to provide us with the desired product using HBPin or BH_3•THF.

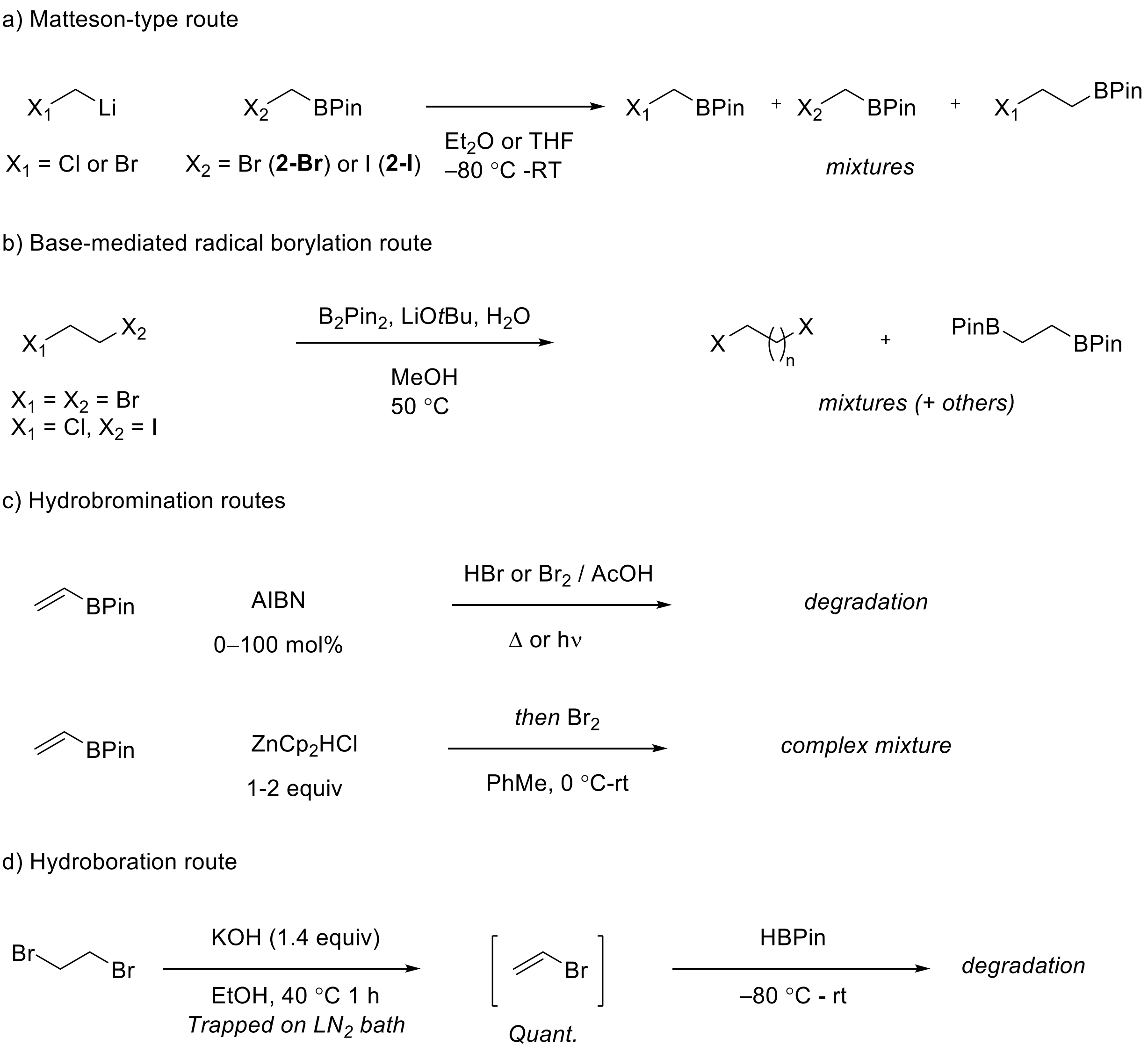

Scheme S5: Summary of attempted routes to 1,2-bromoethylBPin.

4.3 Copies of NMR spectra

2-(3-bromopropyl)-4,4,5,5-tetramethyl-1,3,2-dioxaborolane (S1)

^{1}H NMR (500 MHz, CDCl$_3$)

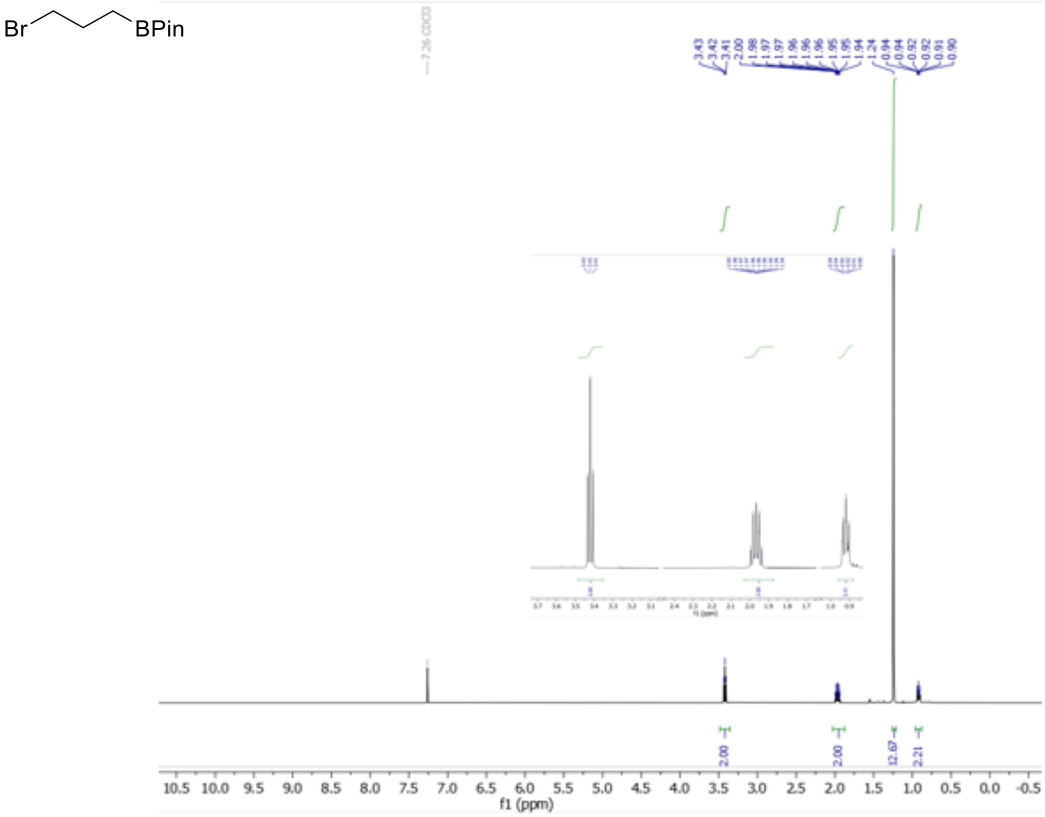

^{13}C NMR (126 MHz, CDCl$_3$)

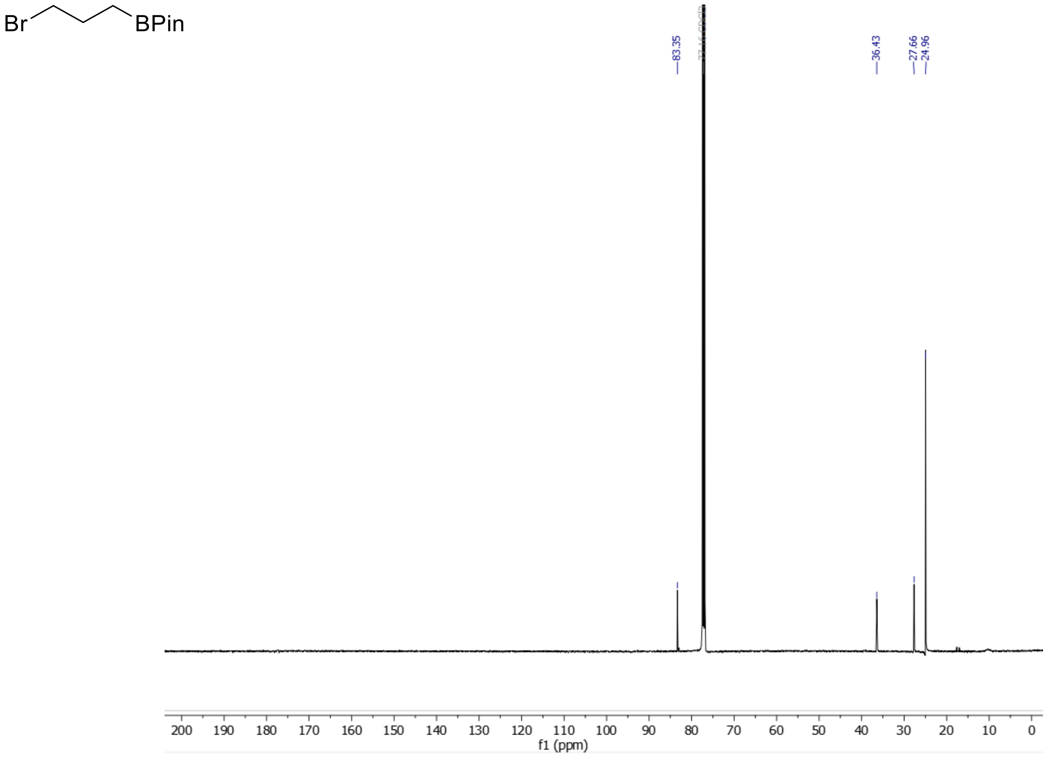

^{11}B NMR (96 MHz, $CDCl_3$)

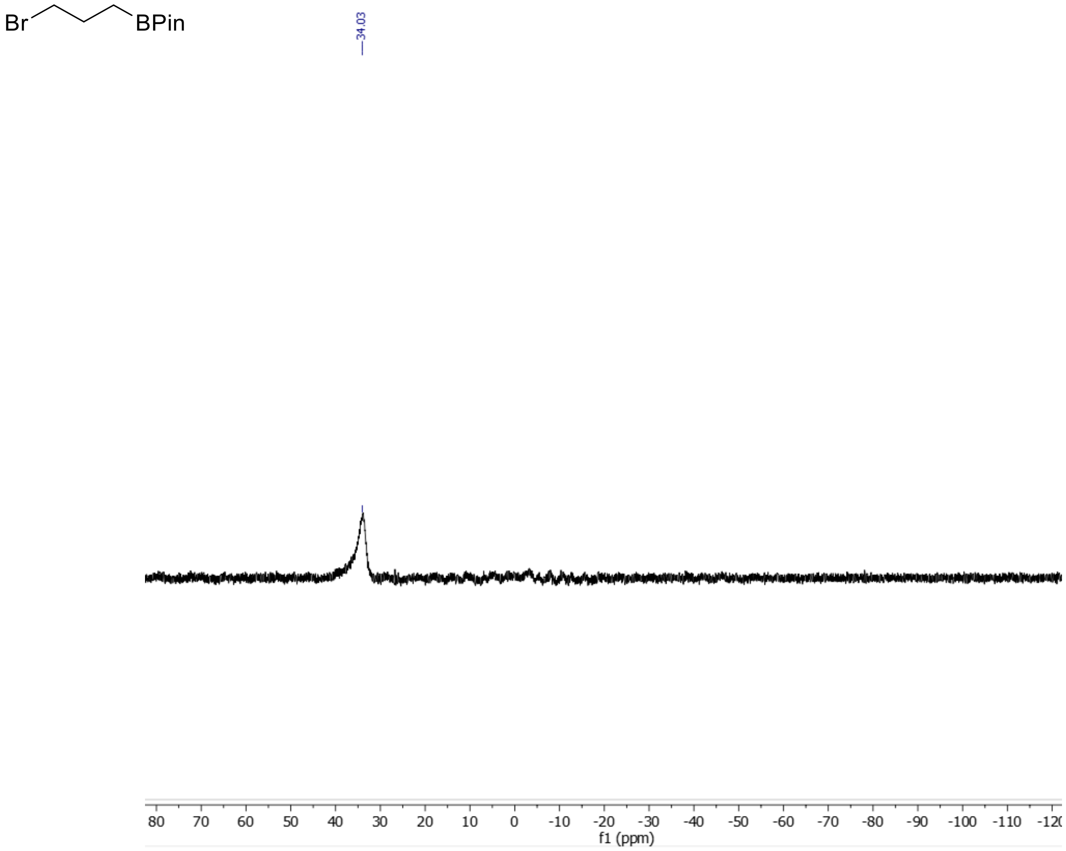

2-(p-tolyl)-1,3,2-dioxaborolane (S2)

^{1}H NMR (500 MHz, $CDCl_3$)

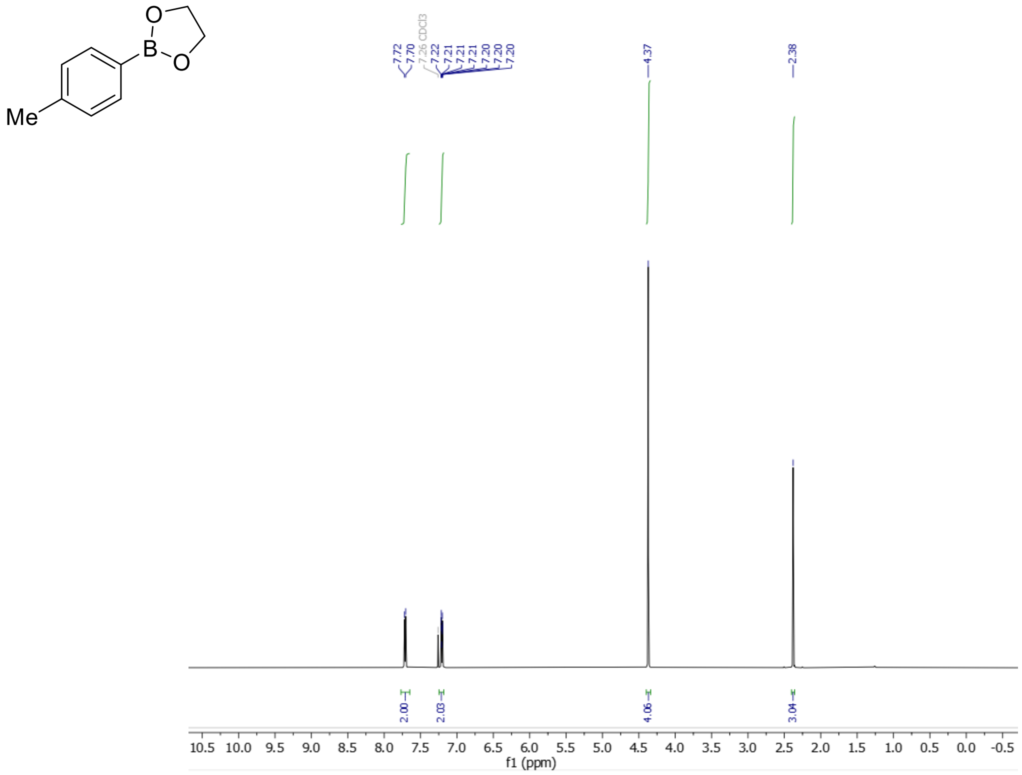

^{13}C NMR (126 MHz, $CDCl_3$)

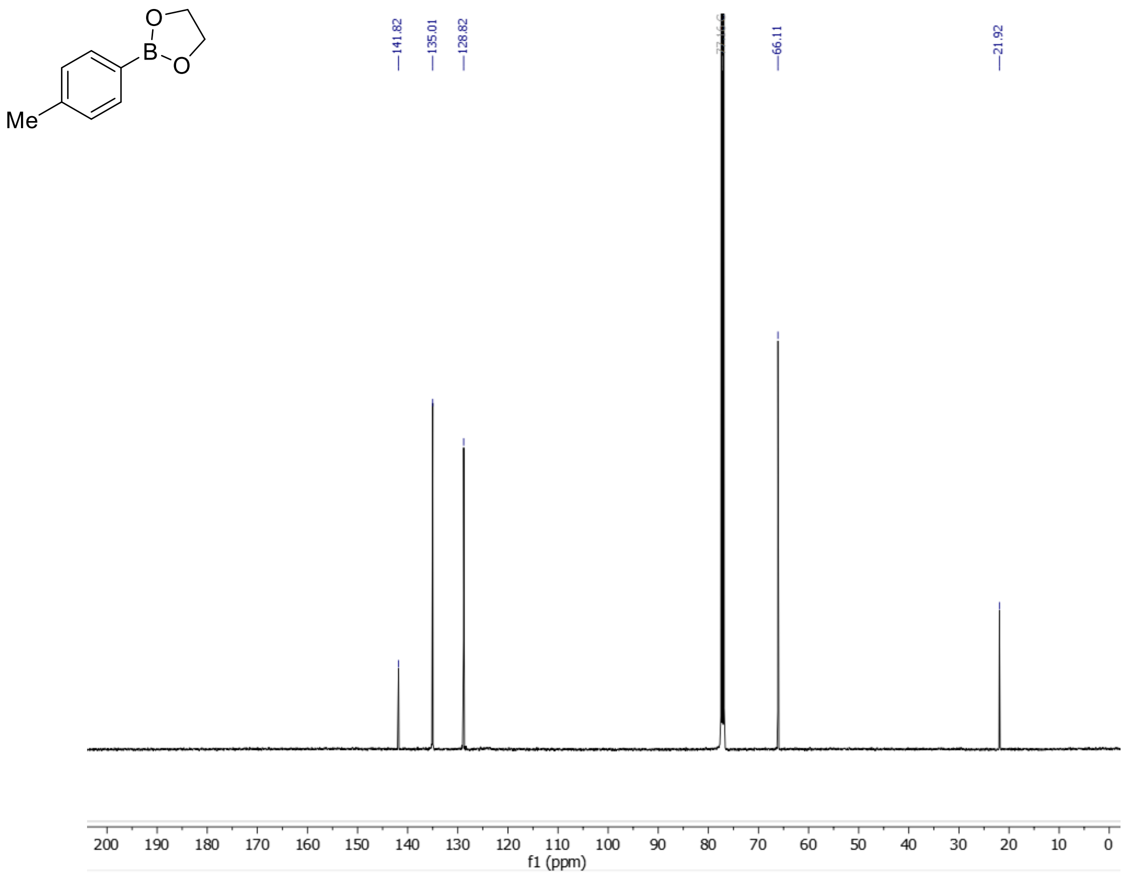

^{11}B NMR (96 MHz, $CDCl_3$)

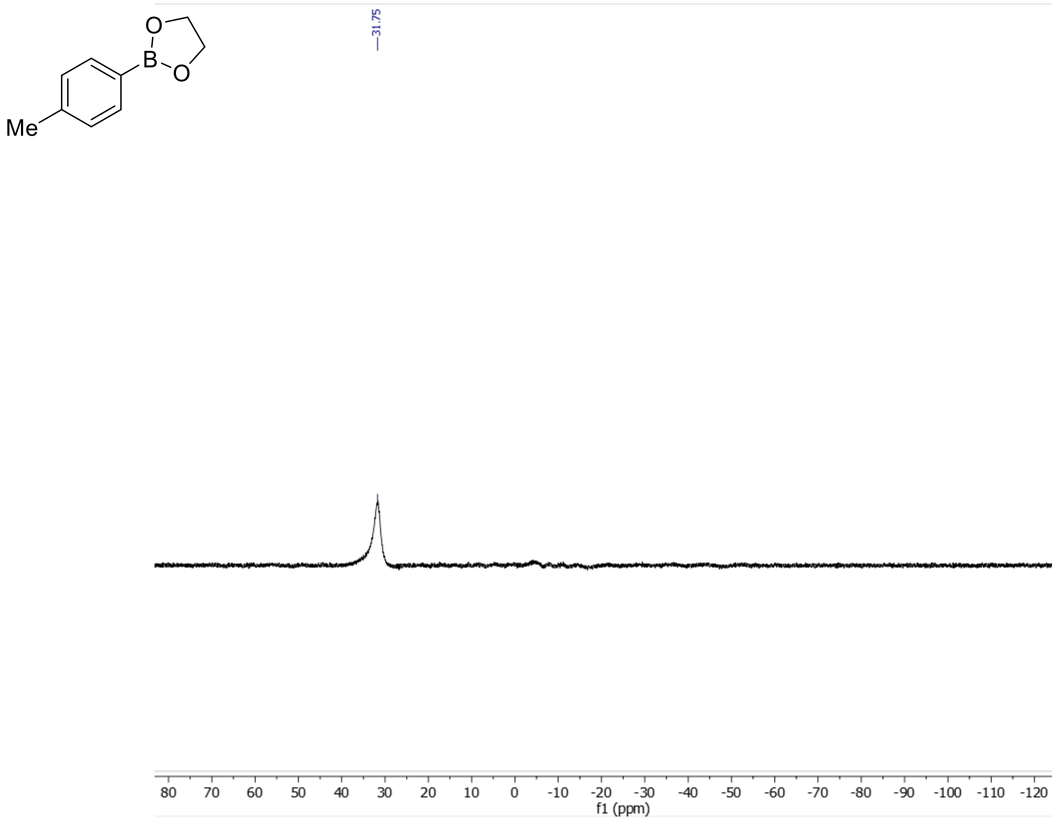

2-(p-tolyl)-1,3,2-dioxaborinane (S3)

^{1}H NMR (500 MHz, $CDCl_3$)

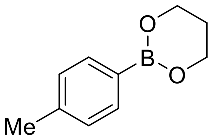

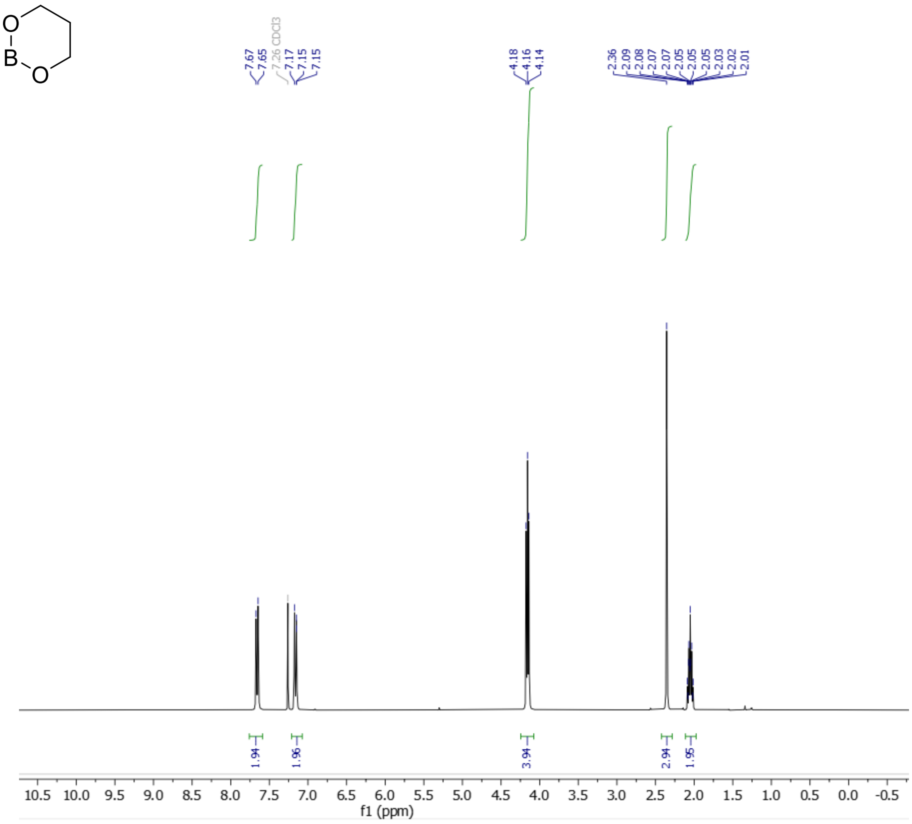

^{13}C NMR (126 MHz, $CDCl_3$)

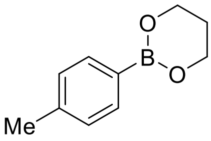

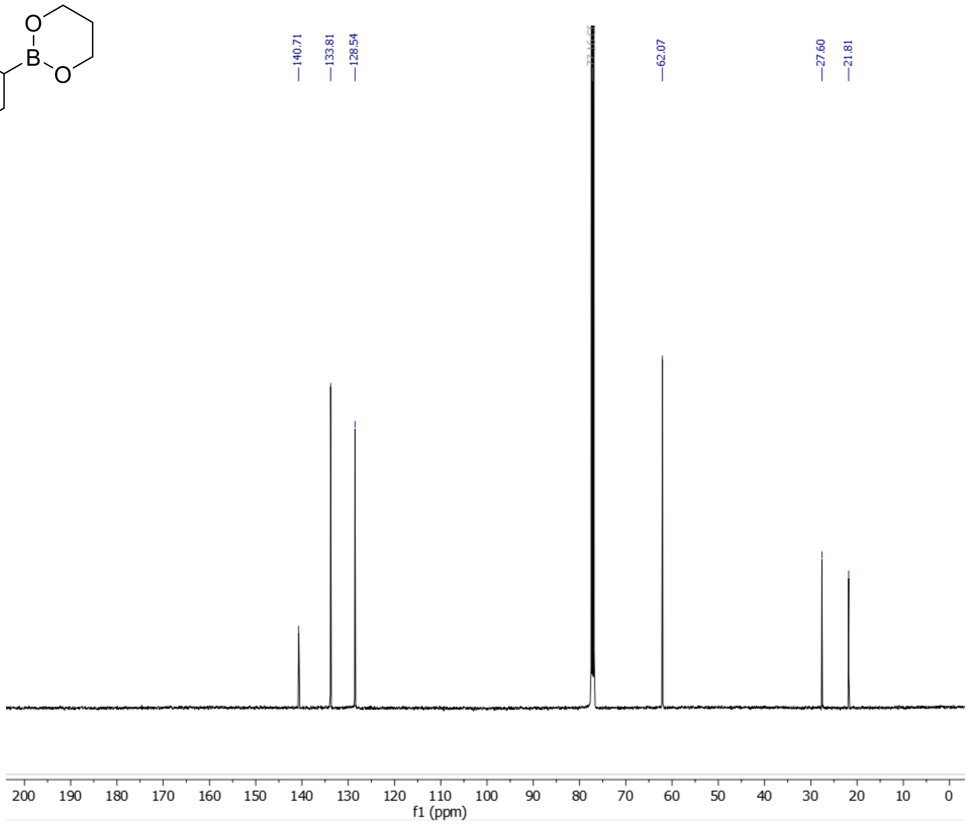

^{11}B NMR (96 MHz, CDCl$_3$)

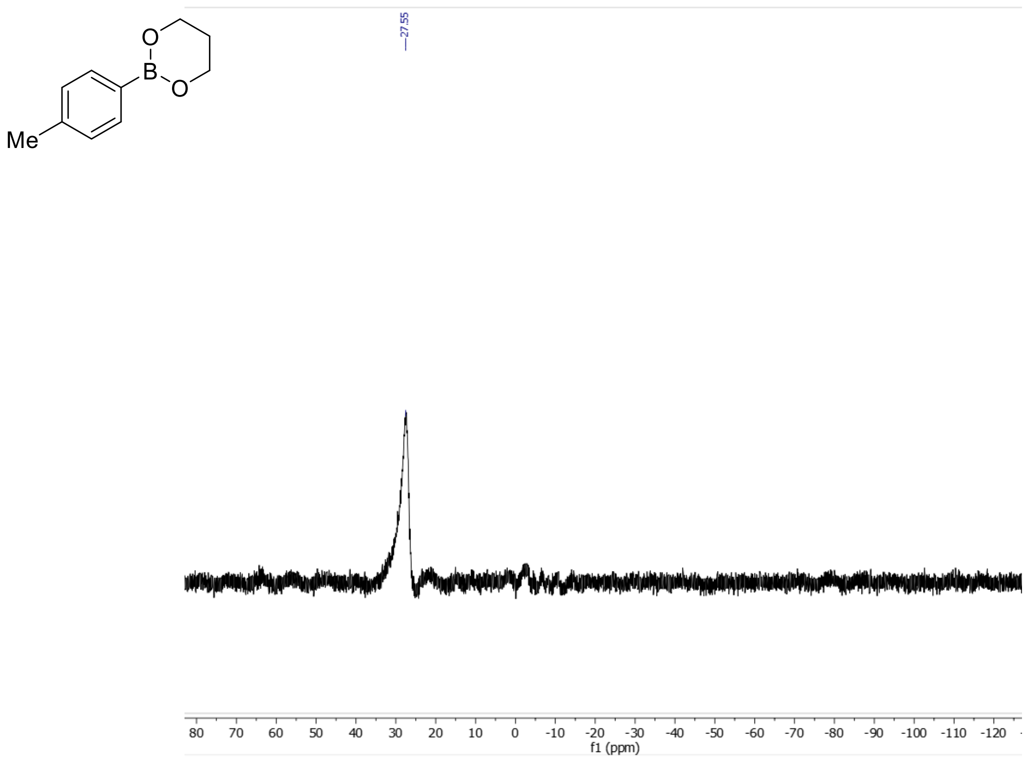

4,6-dimethyl-2-(p-tolyl)-1,3,2-dioxaborinane (S4)

^{1}H NMR (500 MHz, CDCl$_3$)

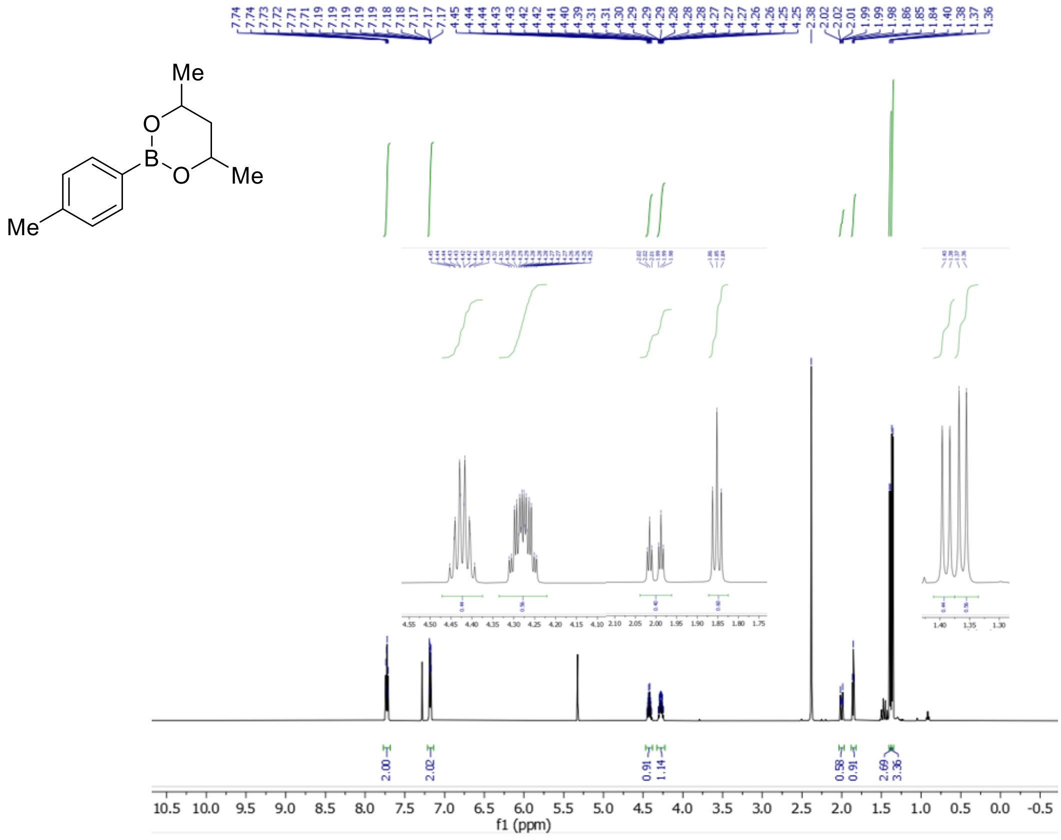

^{13}C NMR (126 MHz, $CDCl_3$)

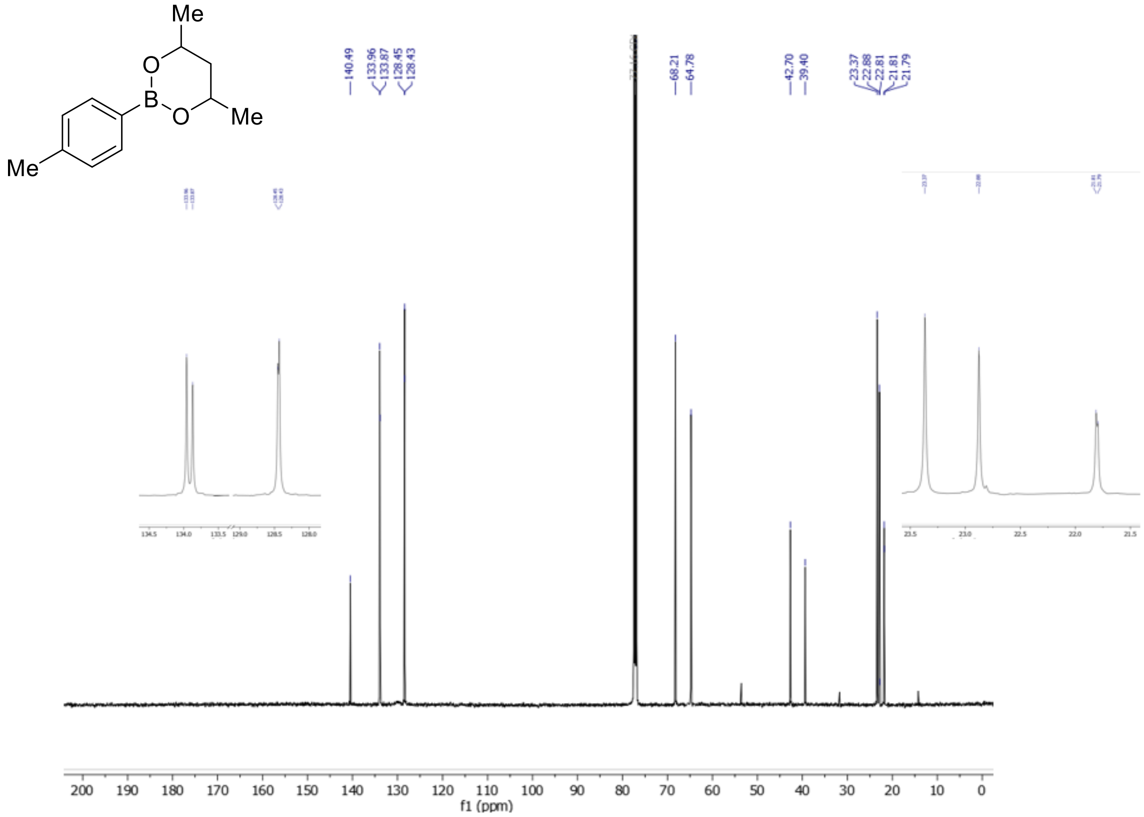

^{11}B NMR (96 MHz, $CDCl_3$)

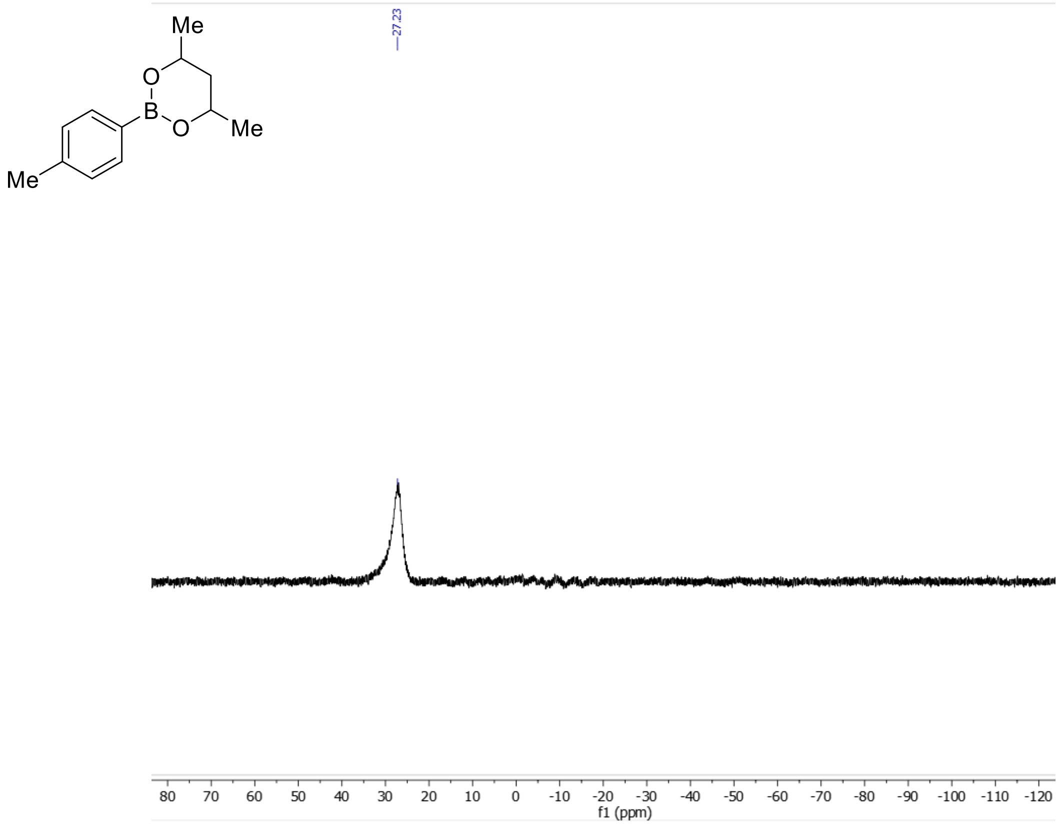

5,5-dimethyl-2-(p-tolyl)-1,3,2-dioxaborinane (S5)

^{1}H NMR (500 MHz, $CDCl_3$)

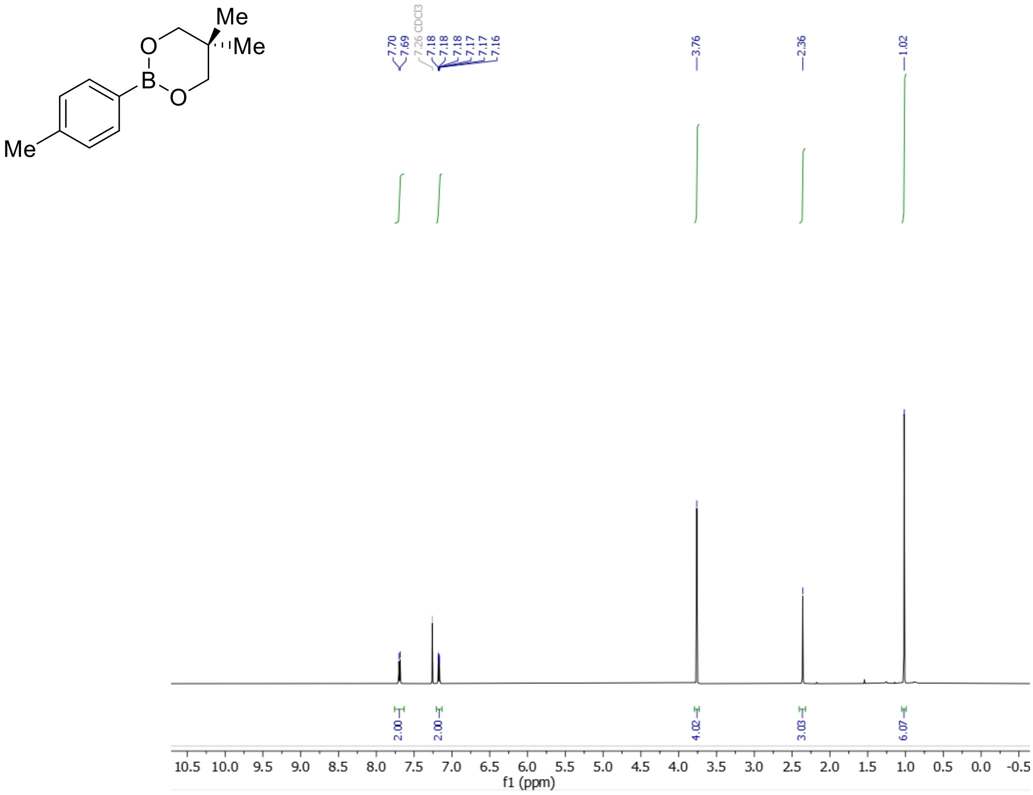

^{13}C NMR (126 MHz, $CDCl_3$)

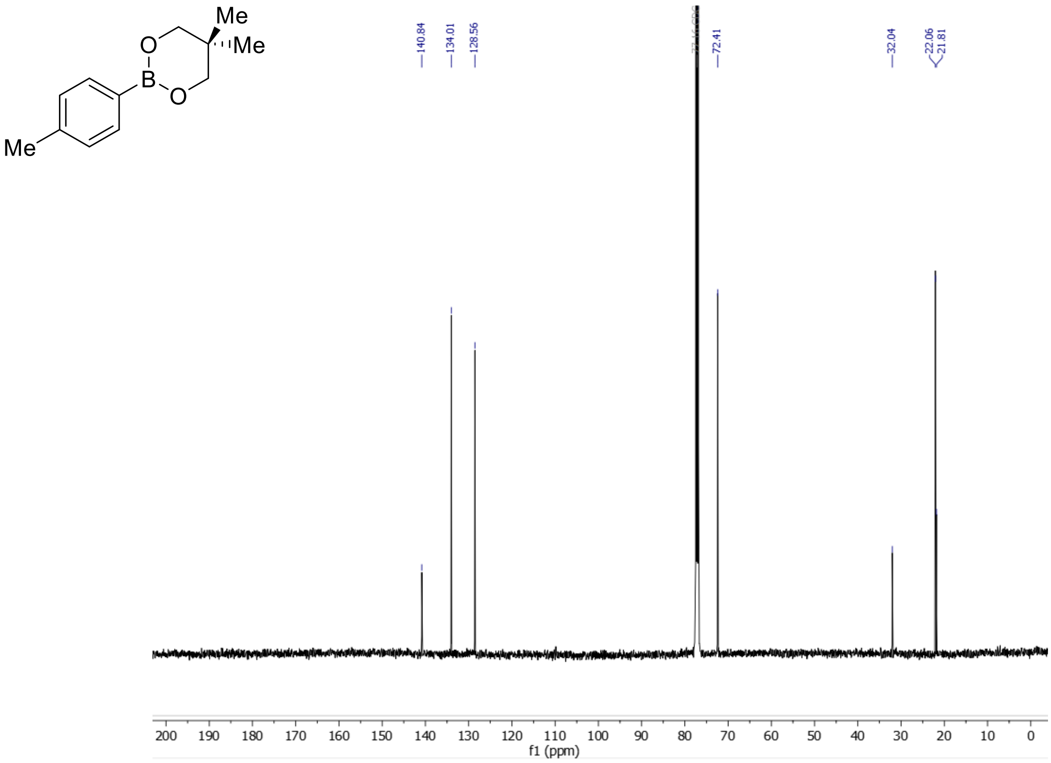

^{11}B NMR (96 MHz, $CDCl_3$)

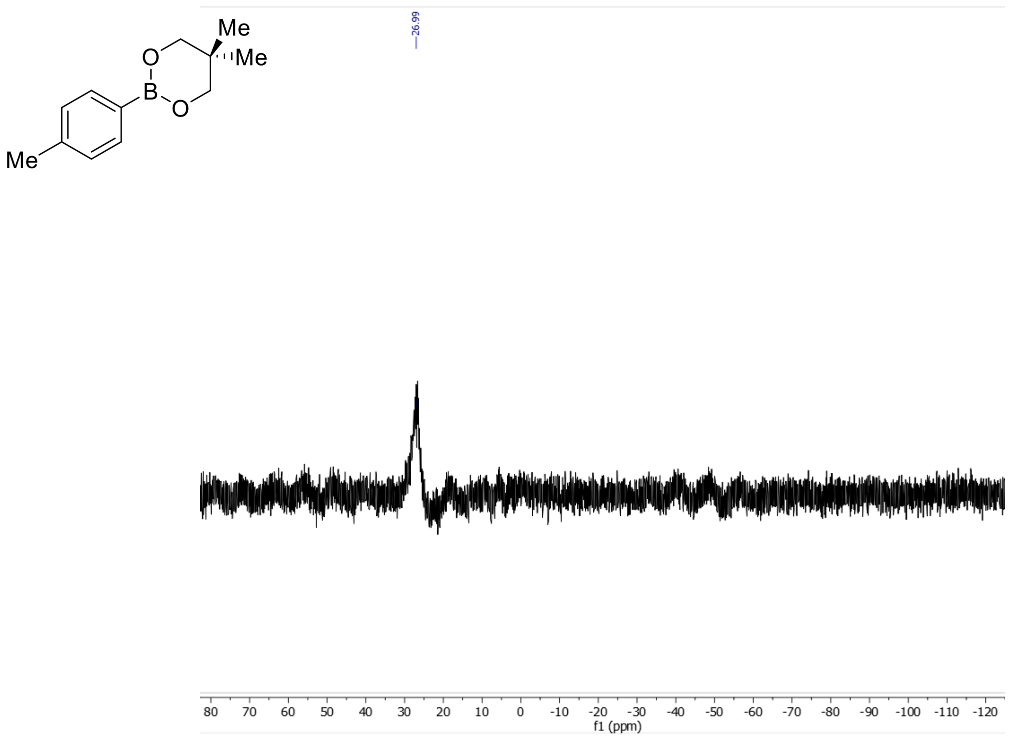

4,4,5,5-tetramethyl-2-(4-(trifluoromethoxy)phenyl)-1,3,2-dioxaborolane (S6)

^{1}H NMR (500 MHz, $CDCl_3$)

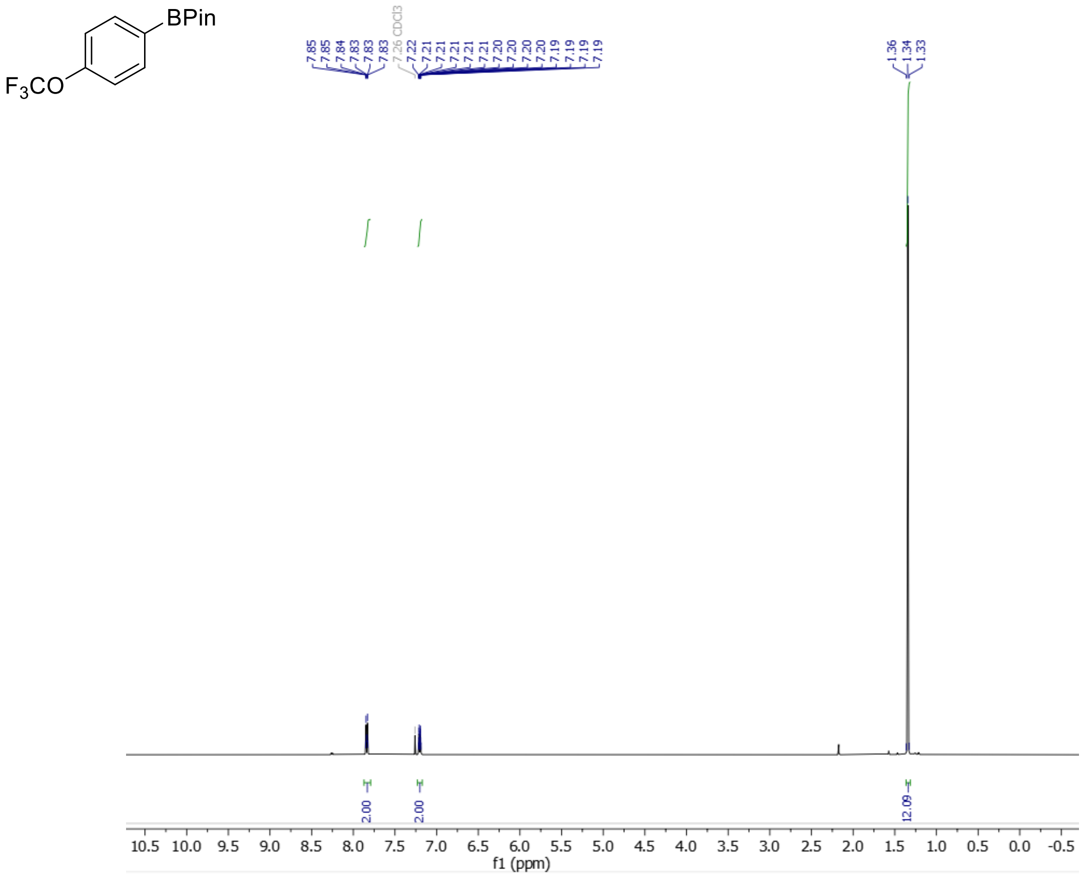

^{13}C NMR (126 MHz, CDCl$_3$)

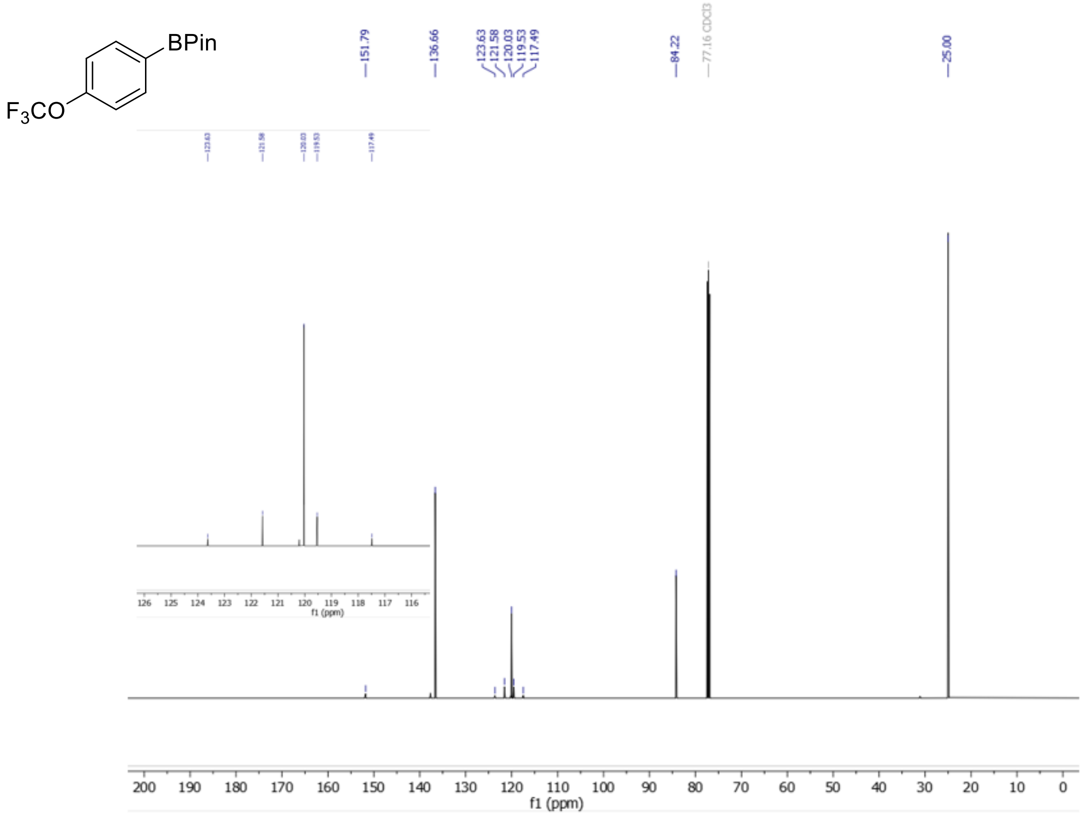

^{11}B NMR (96 MHz, CDCl$_3$)

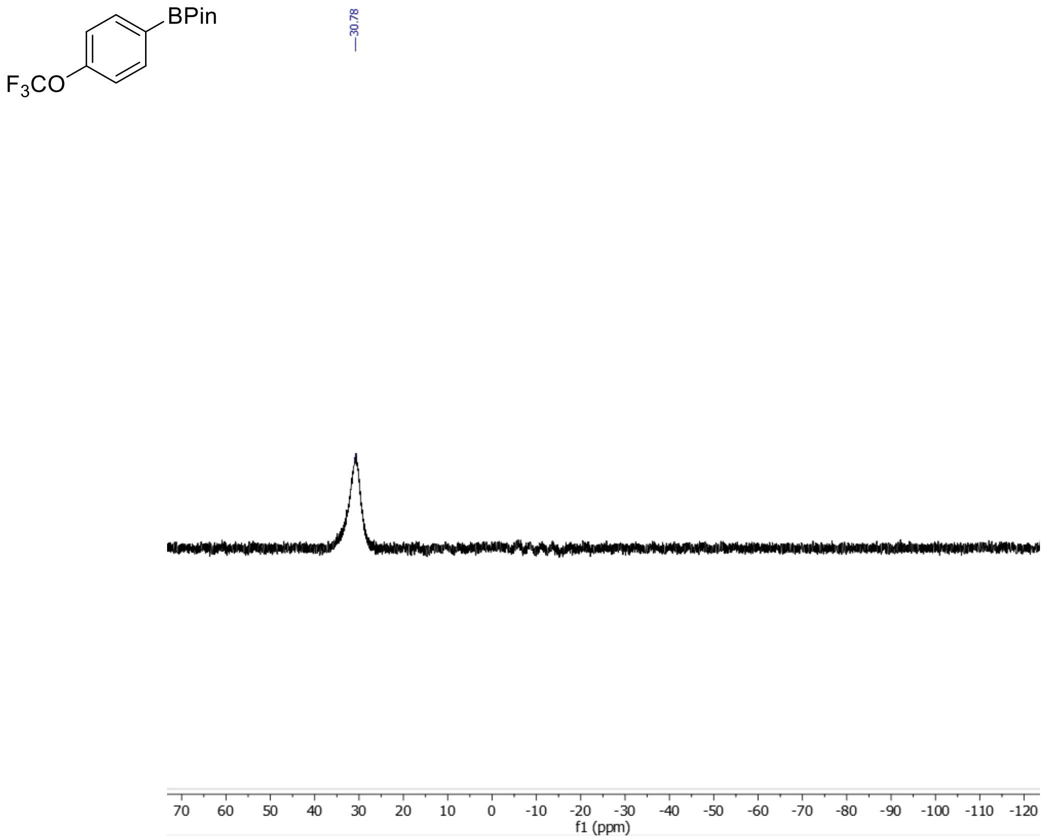

^{19}F NMR (470 MHz, $CDCl_3$)

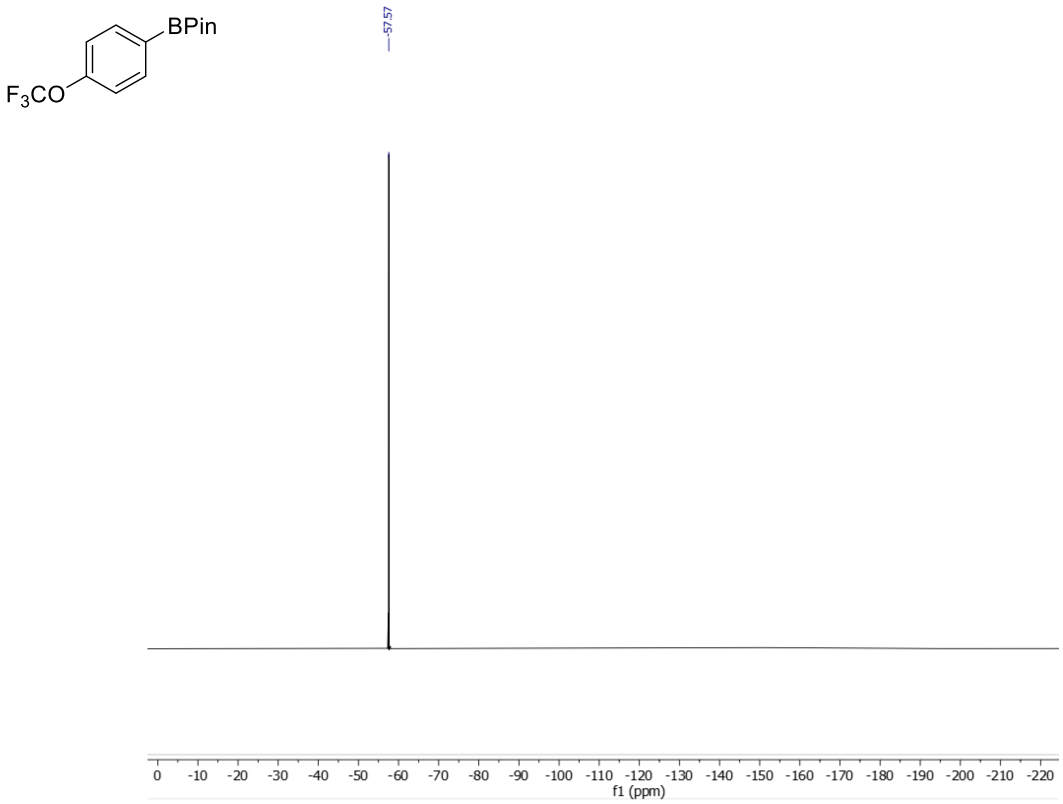

2-(bromomethyl)-4,4,5,5-tetramethyl-1,3,2-dioxaborolane (2-Br)

^{1}H NMR (500 MHz, $CDCl_3$)

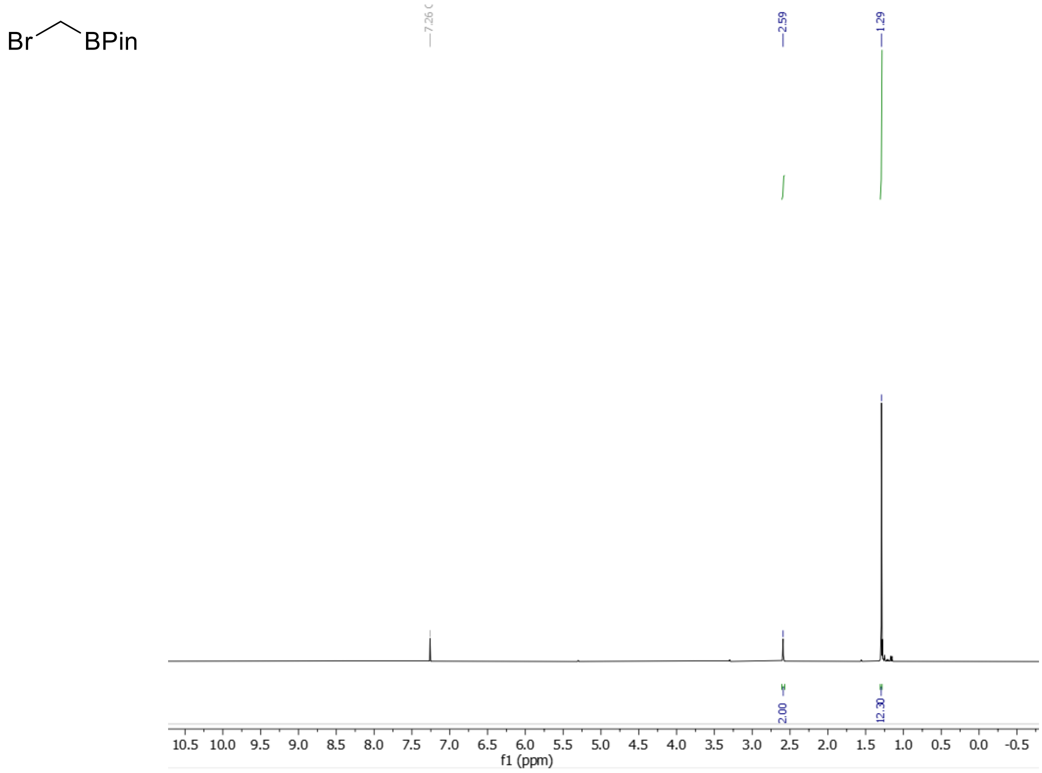

^{13}C NMR (126 MHz, $CDCl_3$)

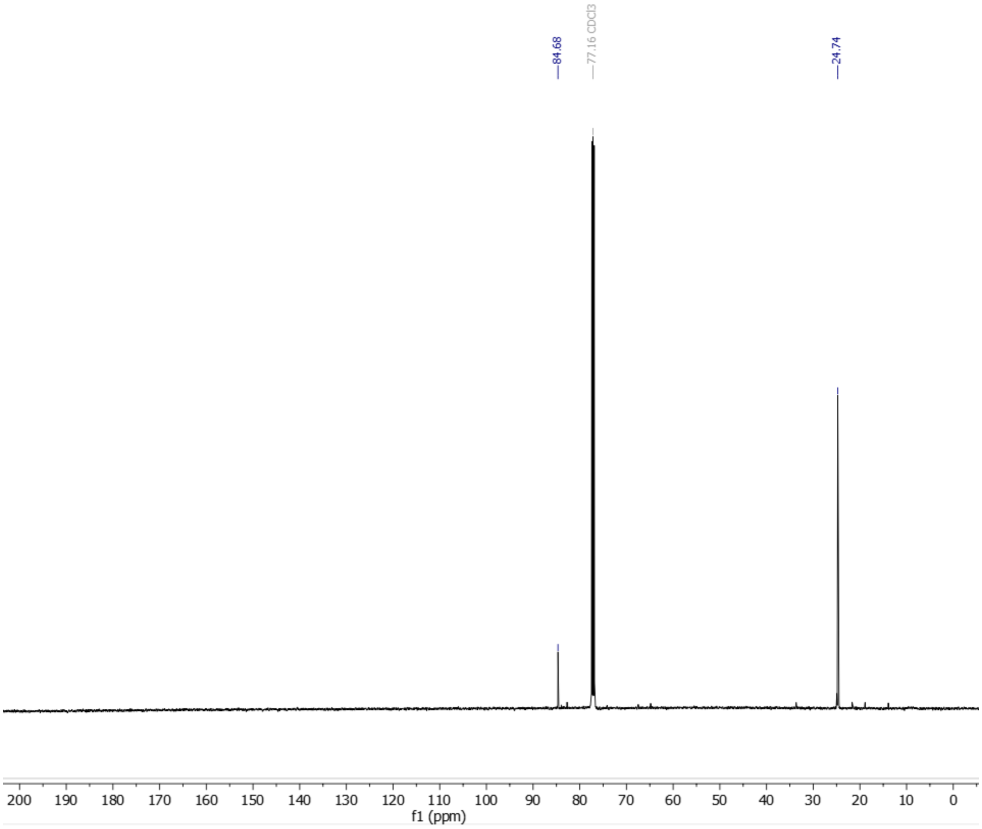

^{11}B NMR (96 MHz, $CDCl_3$)

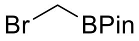

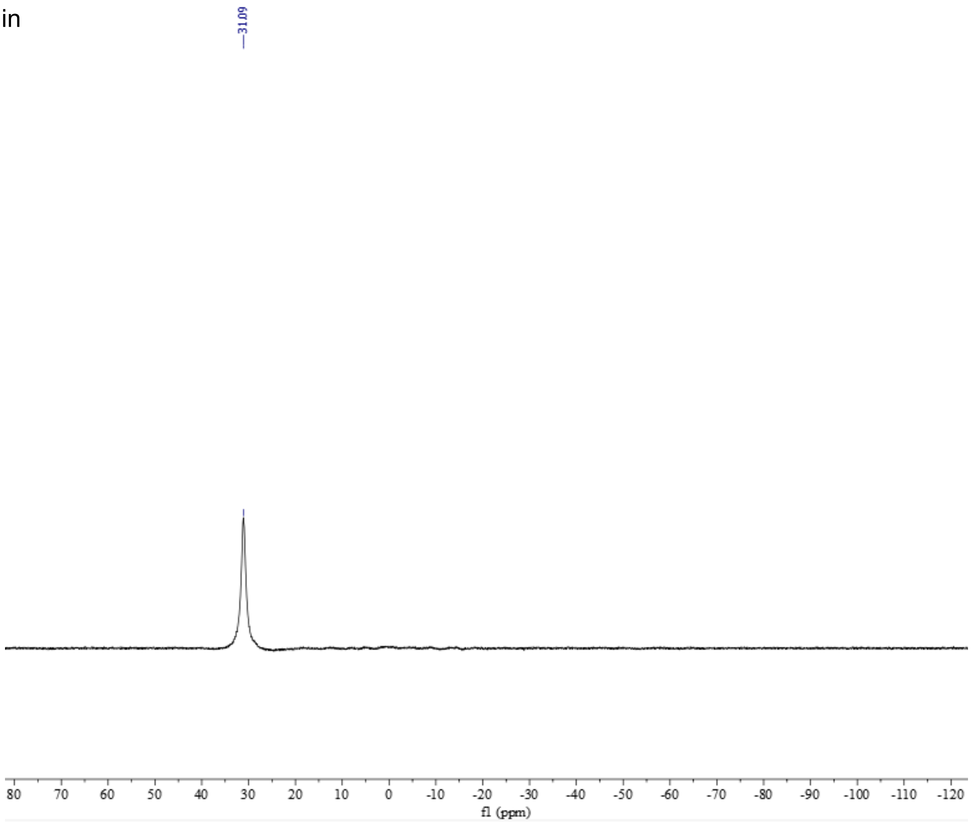

2-(iodomethyl)-4,4,5,5-tetramethyl-1,3,2-dioxaborolane (2-I)

^{1}H NMR (500 MHz, $CDCl_3$)

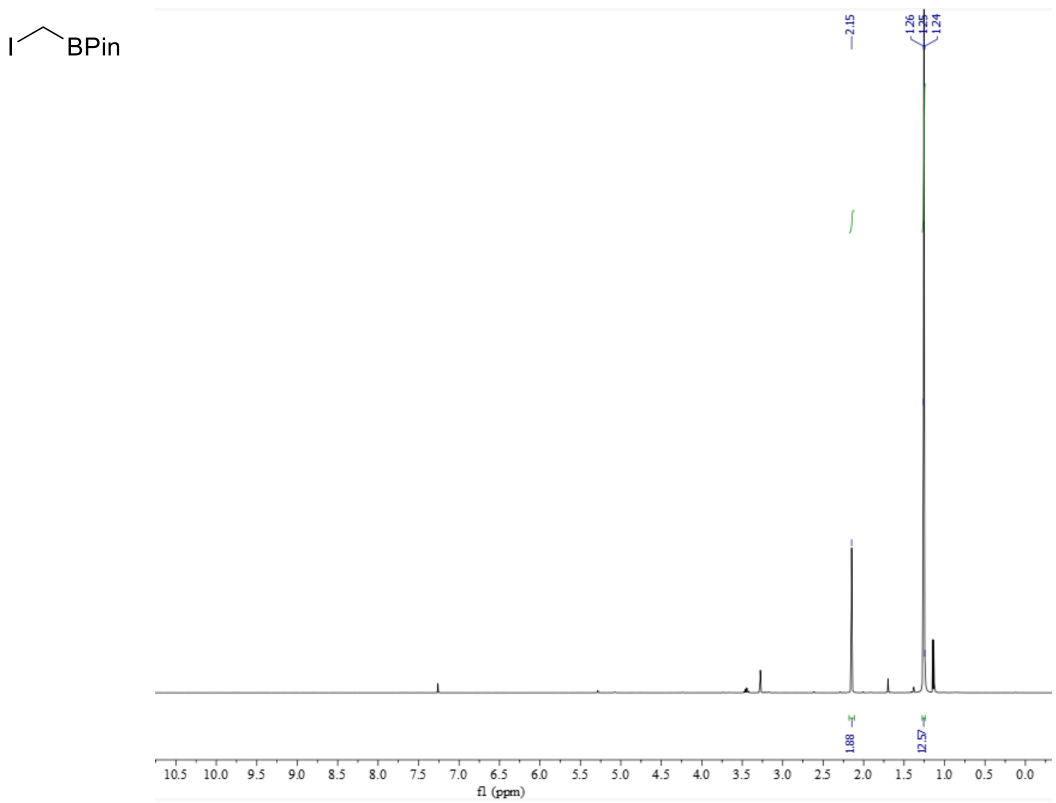

^{13}C NMR (126 MHz, $CDCl_3$)

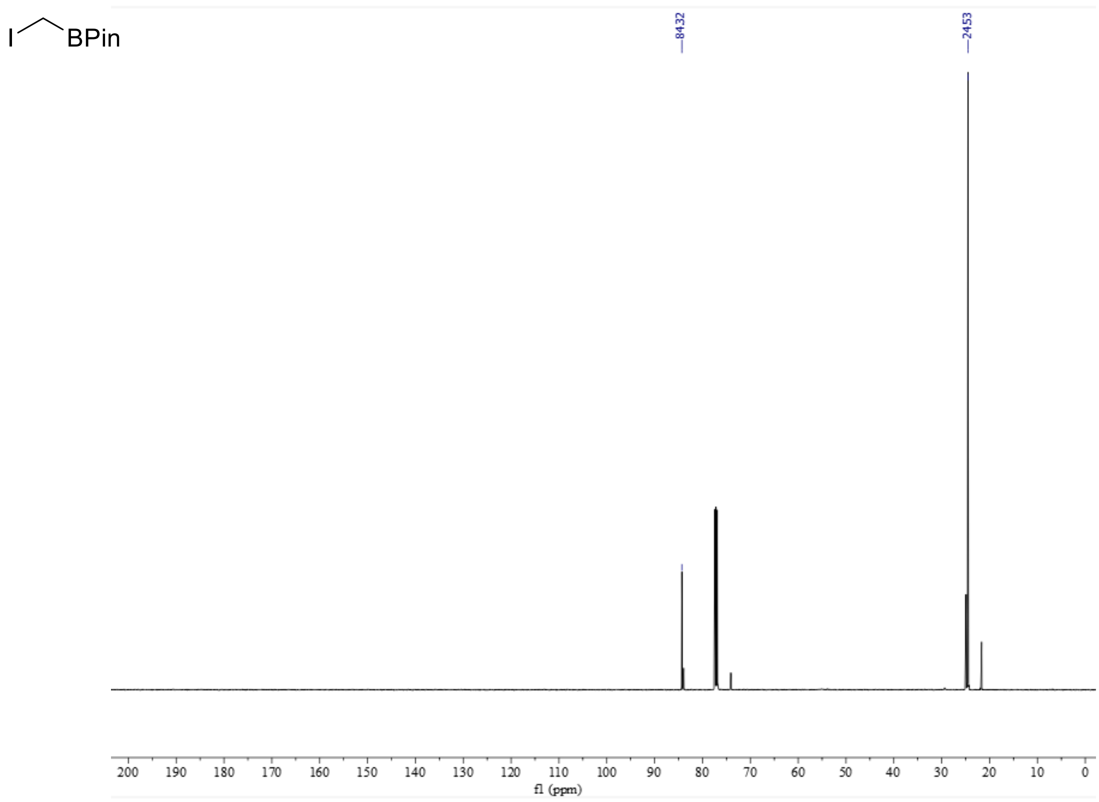

^{11}B NMR (96 MHz, $CDCl_3$)

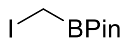

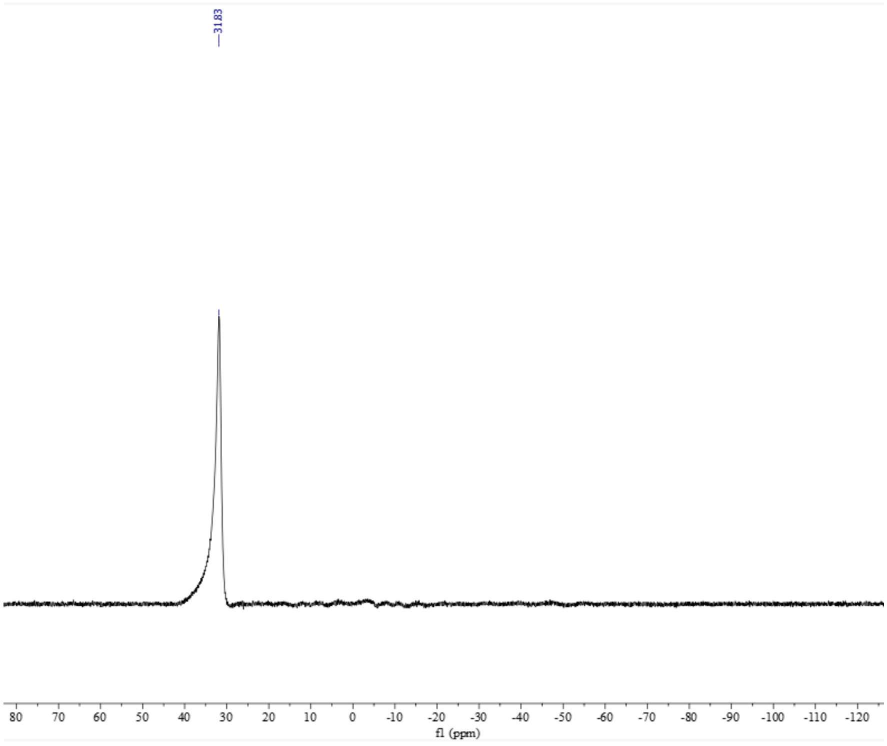

2-(chloromethyl)-4,4,5,5-tetramethyl-1,3,2-dioxaborolane (2-Cl)

^{1}H NMR (500 MHz, $CDCl_3$)

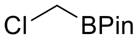

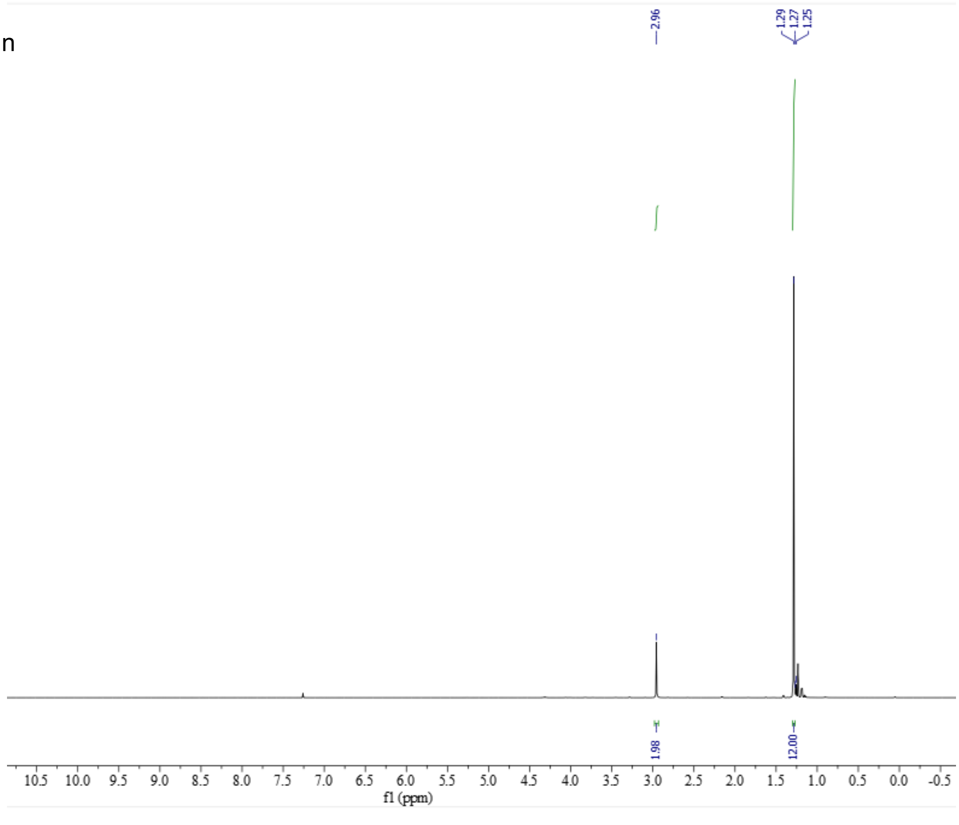

^{13}C NMR (126 MHz, CDCl$_3$)

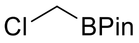

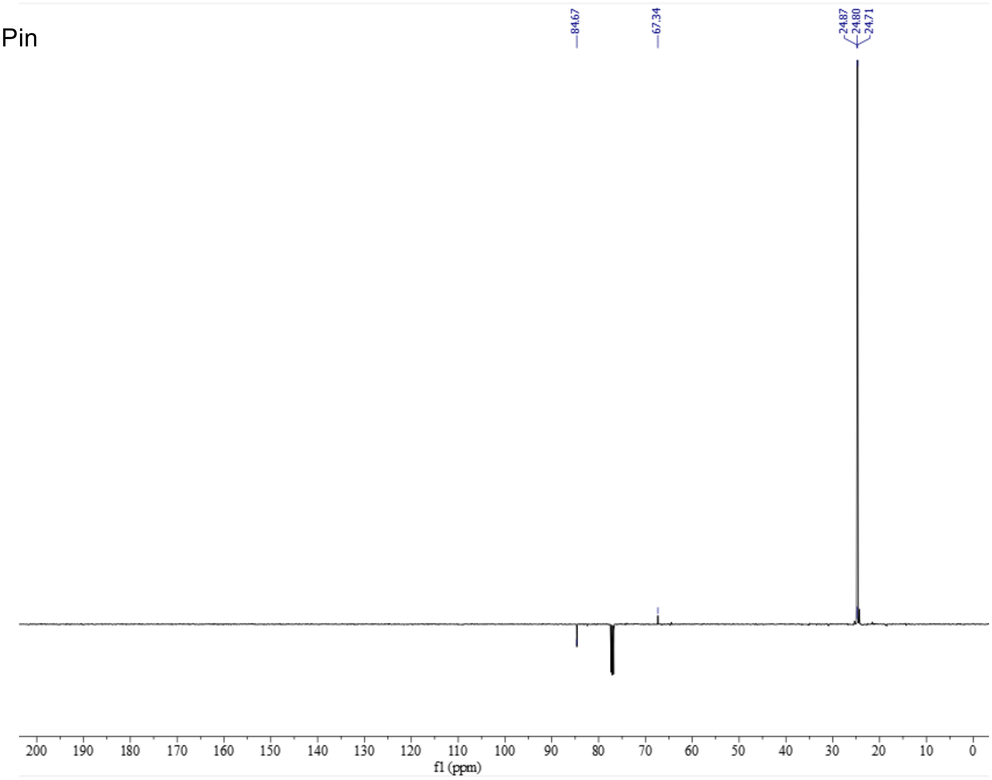

^{11}B NMR (96 MHz, CDCl$_3$)

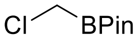

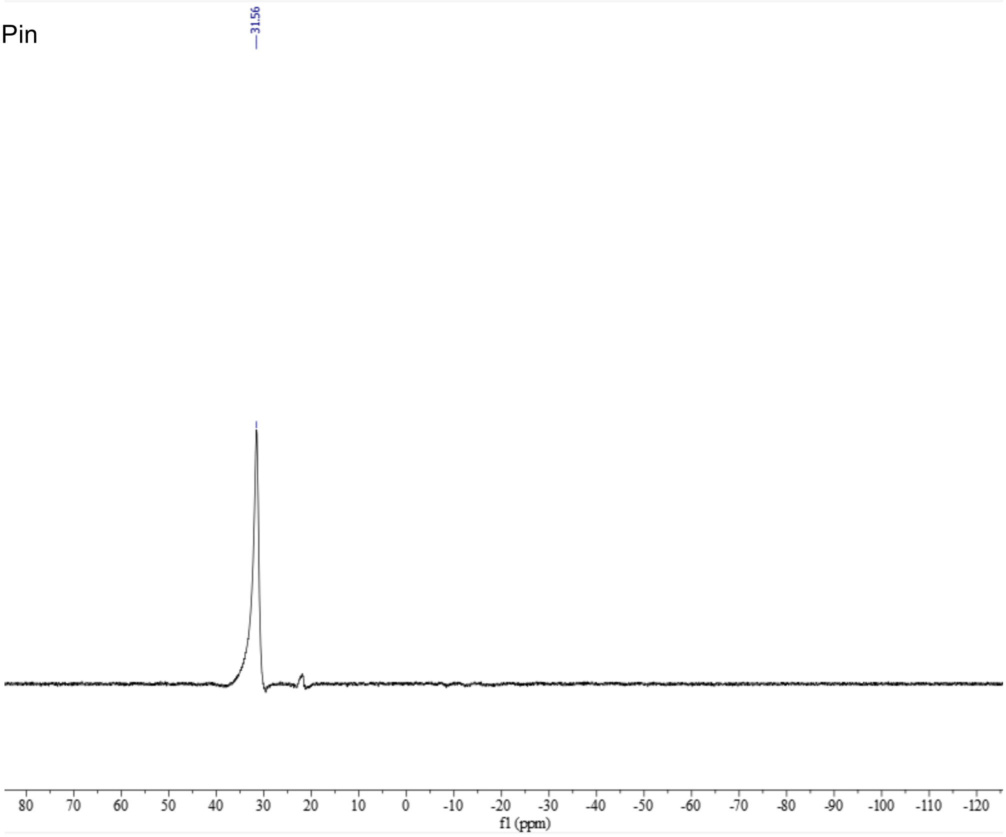

4,4,5,5-tetramethyl-2-(4-methylbenzyl)-1,3,2-dioxaborolane (3)

^{1}H NMR (500 MHz, CDCl$_3$)

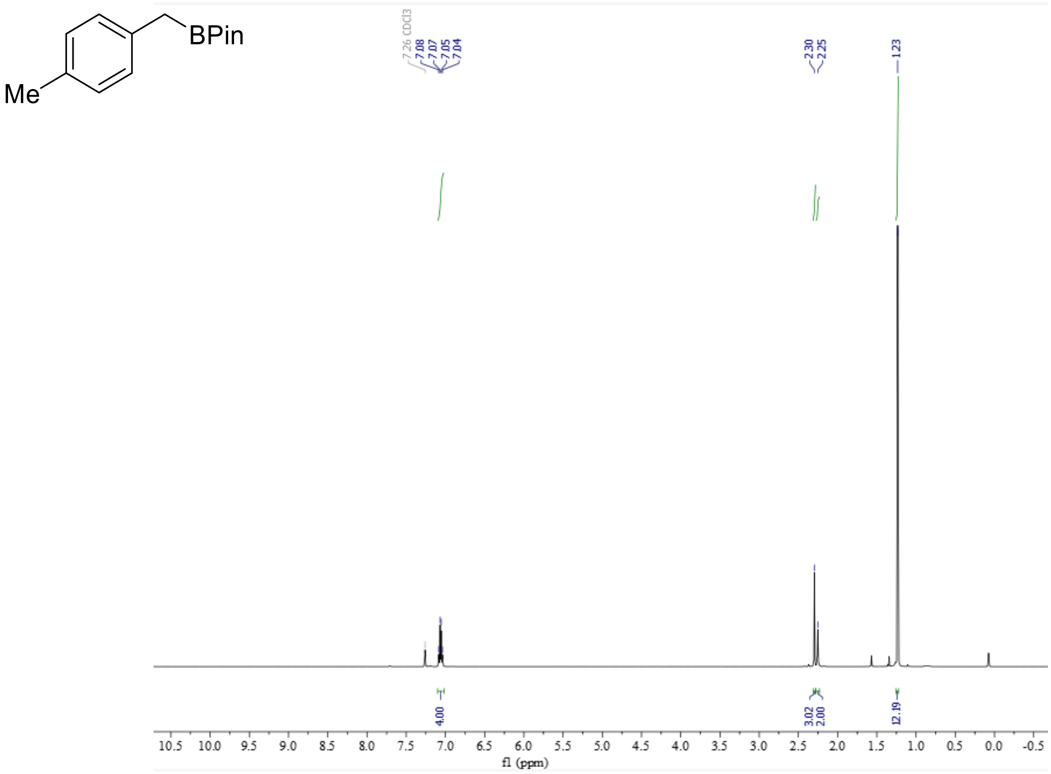

^{13}C NMR (126 MHz, CDCl$_3$)

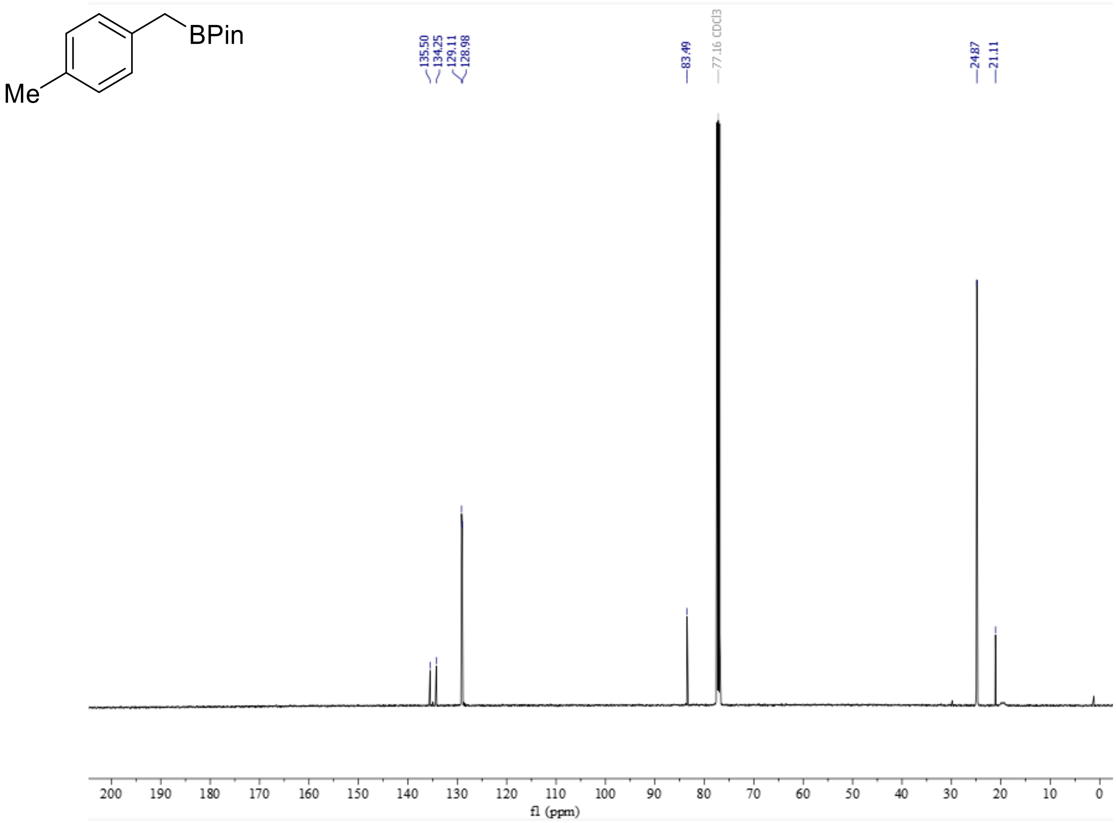

^{11}B NMR (96 MHz, $CDCl_3$)

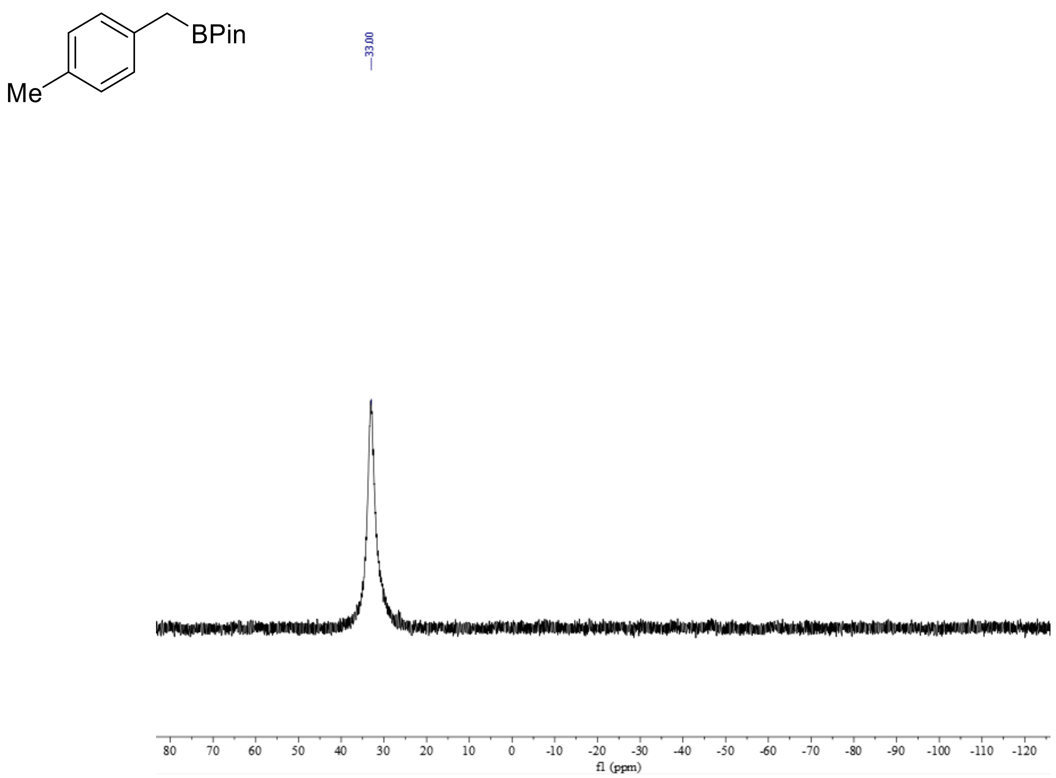

4,4,5,5-tetramethyl-2-(p-tolyl)-1,3,2-dioxaborolane (4)

^{1}H NMR (500 MHz, $CDCl_3$)

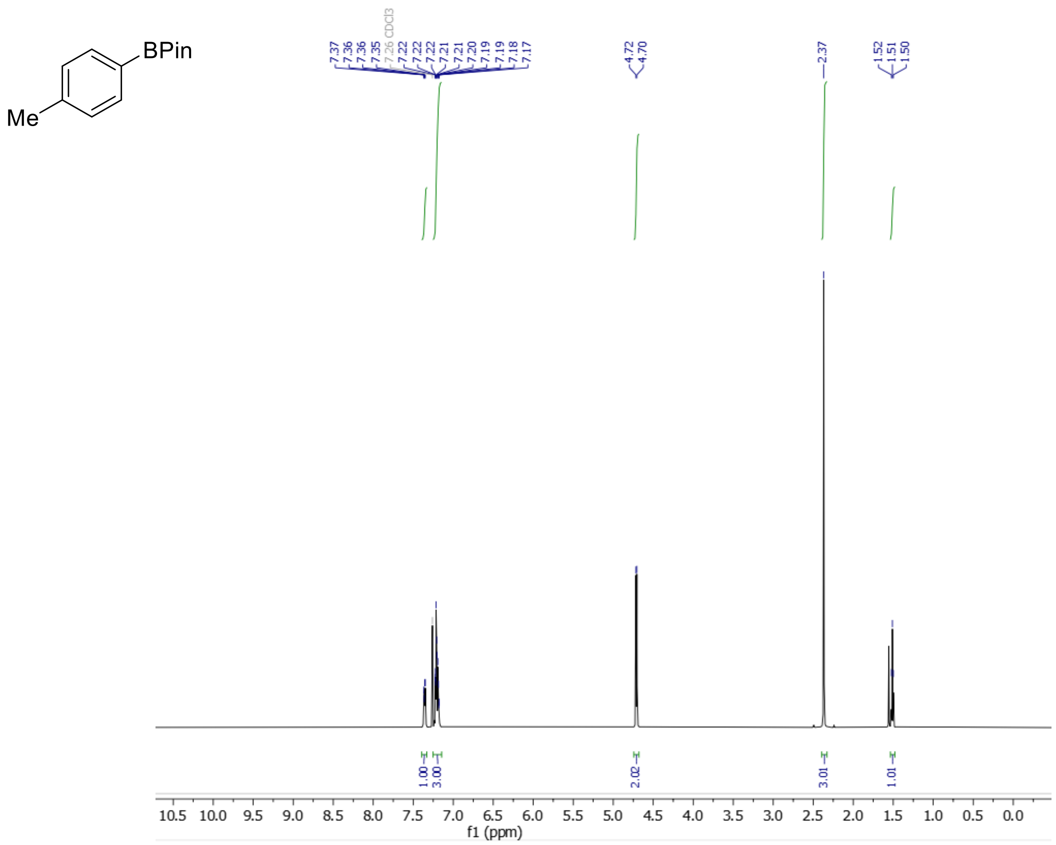

^{13}C NMR (126 MHz, $CDCl_3$)

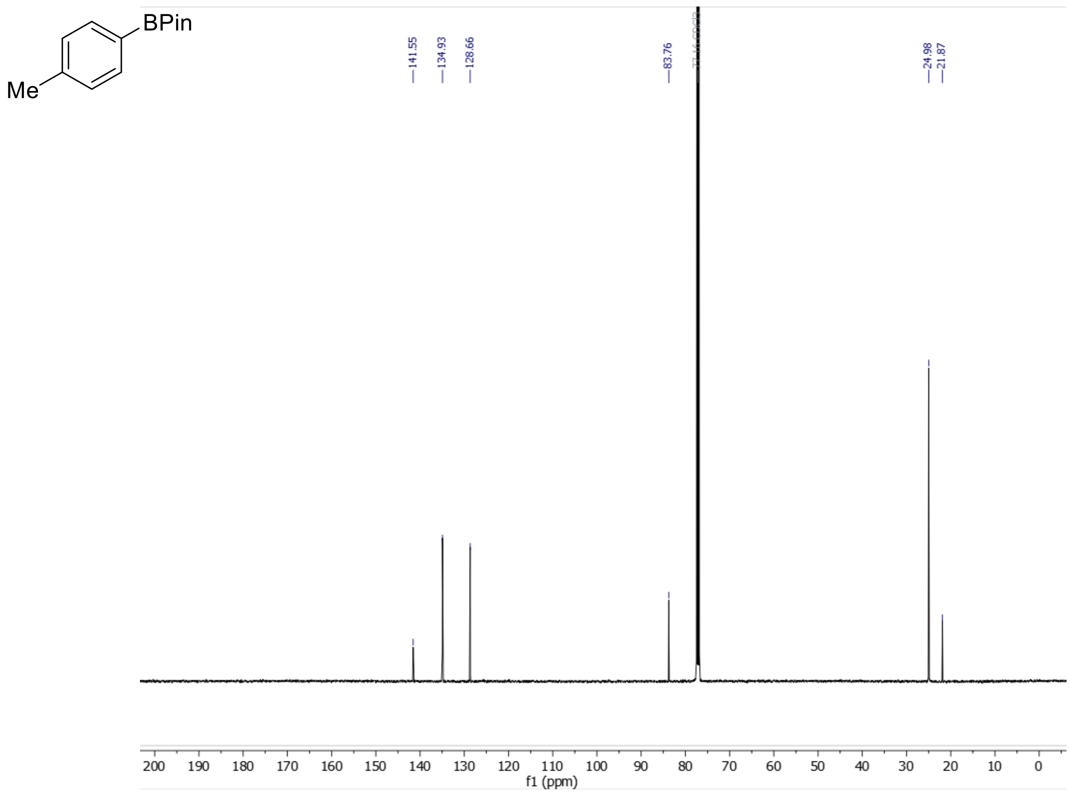

^{11}B NMR (96 MHz, $CDCl_3$)

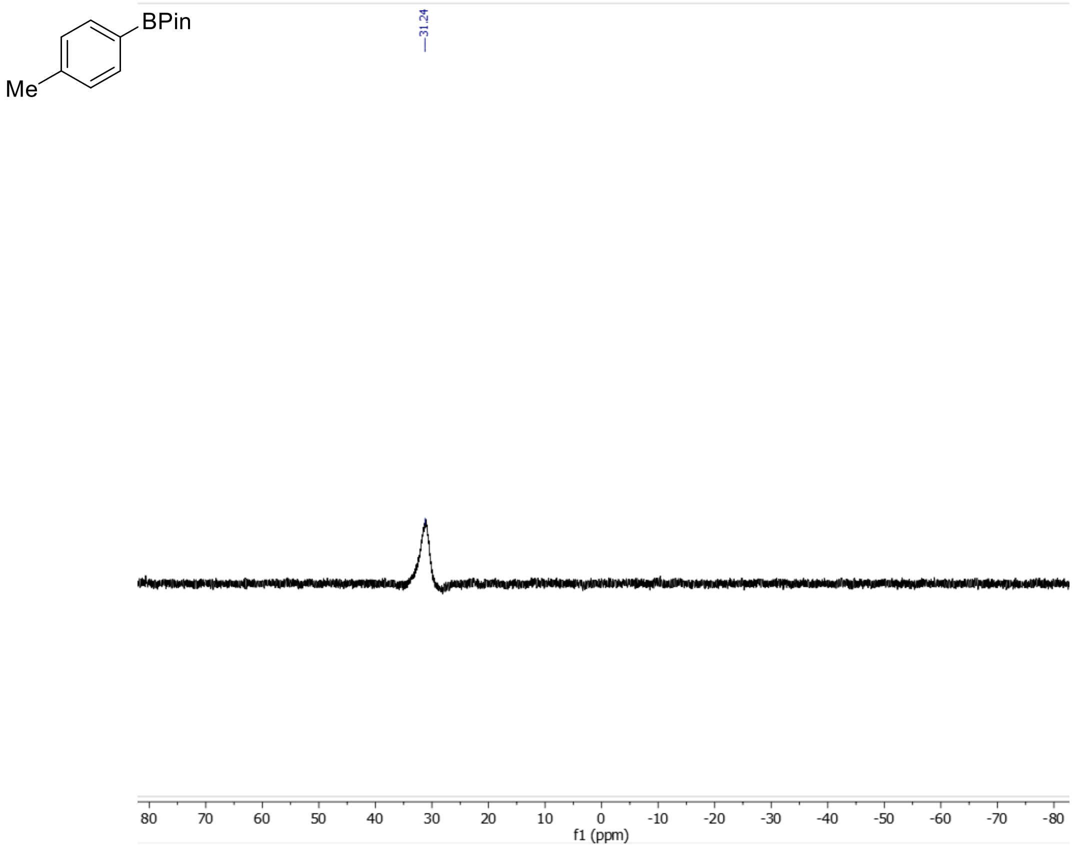

2-benzyl-4,4,5,5-tetramethyl-1,3,2-dioxaborolane (5)

^{1}H NMR (500 MHz, $CDCl_3$)

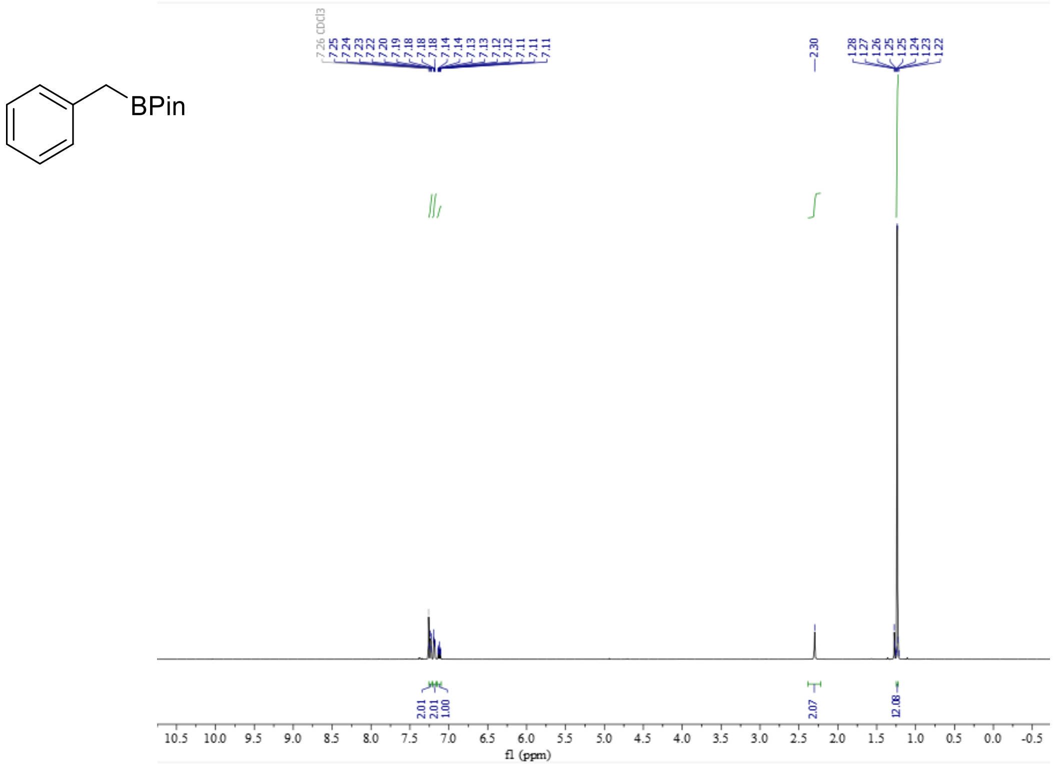

^{13}C NMR (126 MHz, $CDCl_3$)

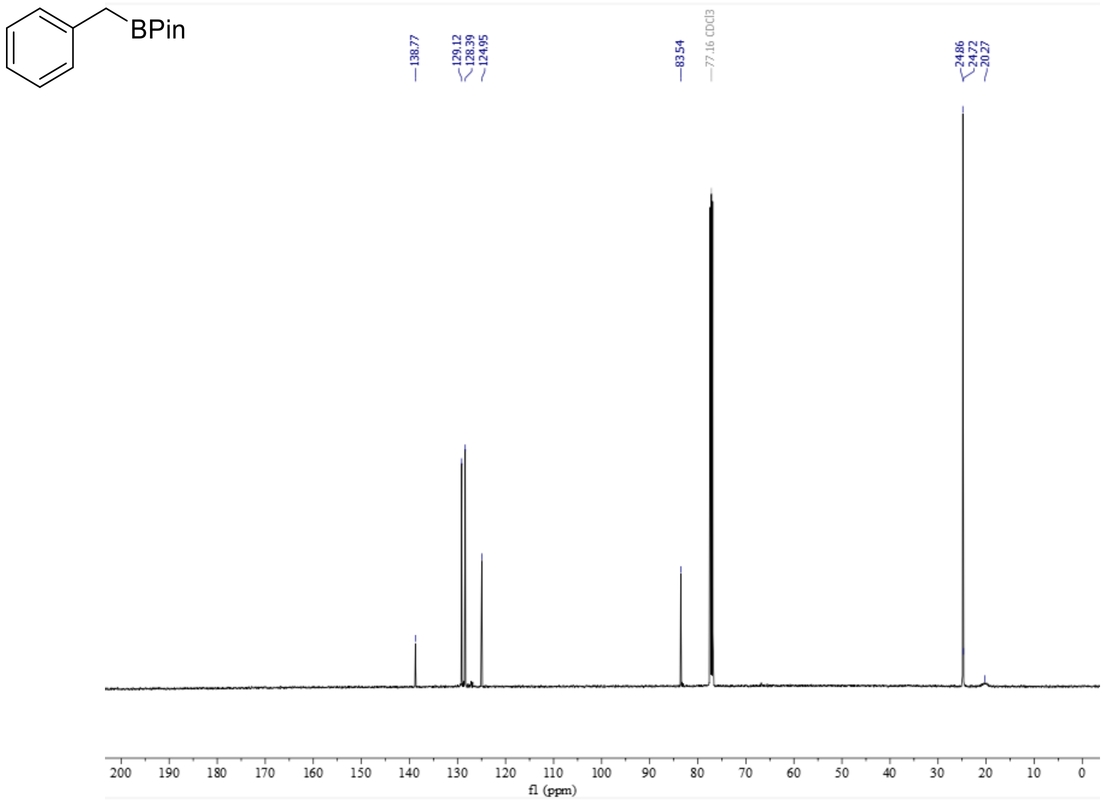

^{1}H-^{13}C-HSQC NMR – *α-boryl carbon atom highlighted*

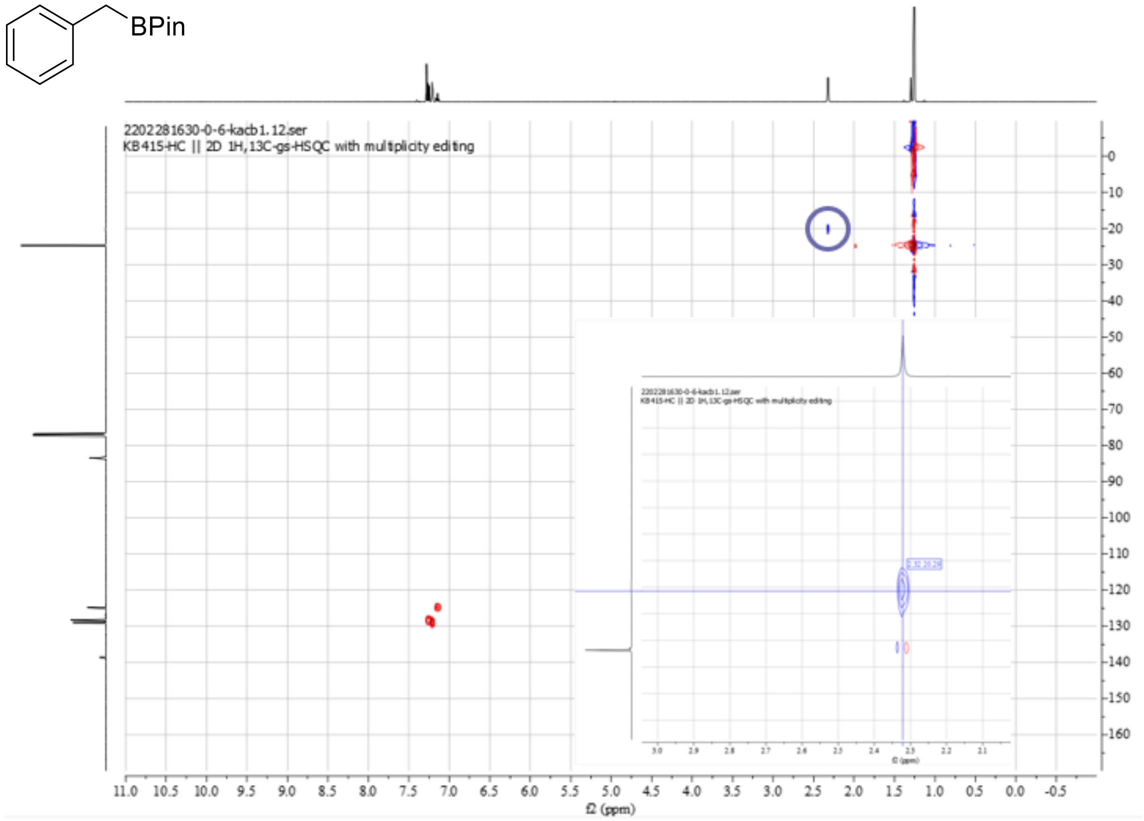

^{11}B NMR (96 MHz, $CDCl_3$)

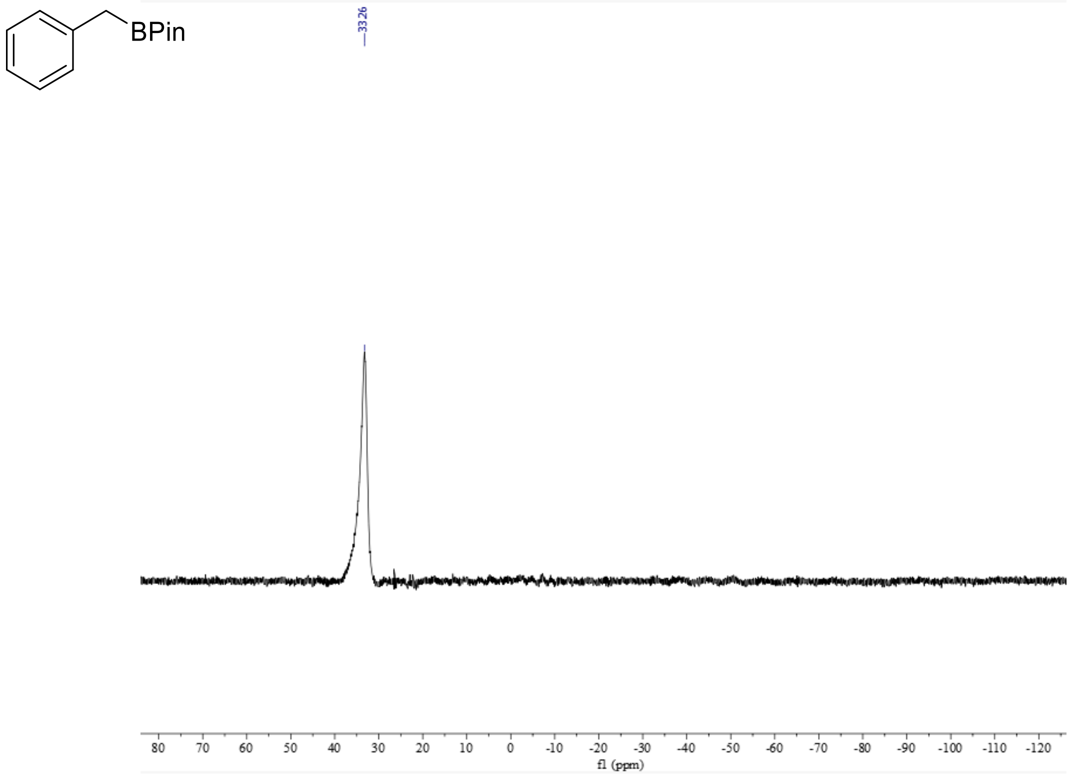

4,4,5,5-tetramethyl-2-(2-methylbenzyl)-1,3,2-dioxaborolane (6)

^{1}H NMR (500 MHz, $CDCl_3$)

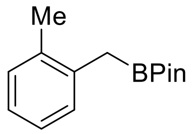

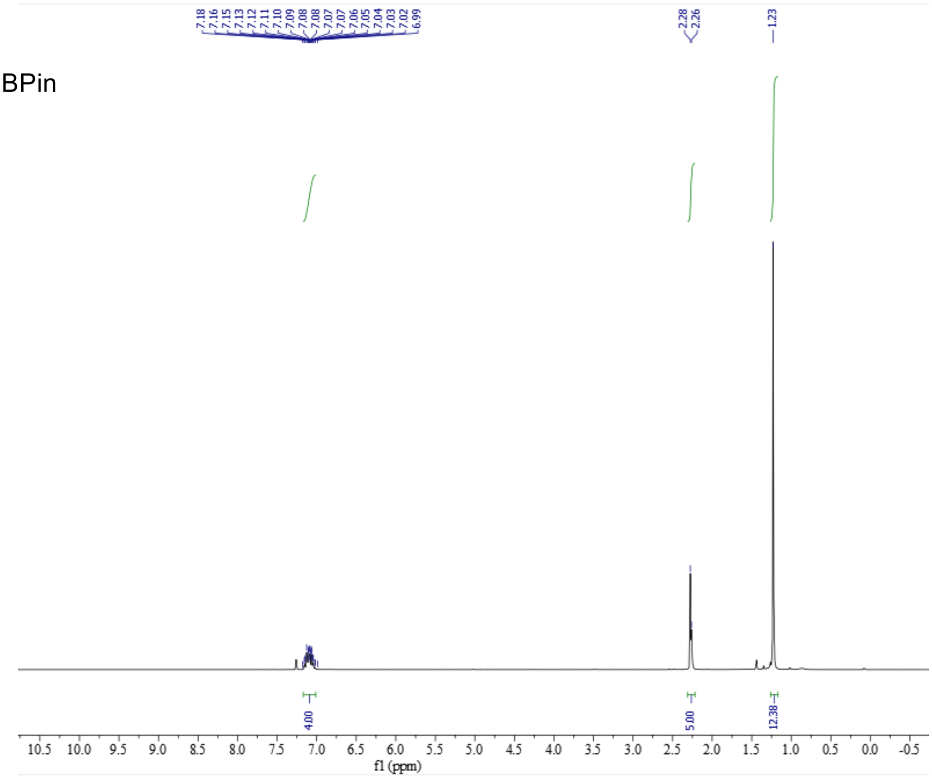

^{13}C NMR (126 MHz, $CDCl_3$)

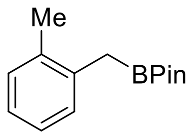

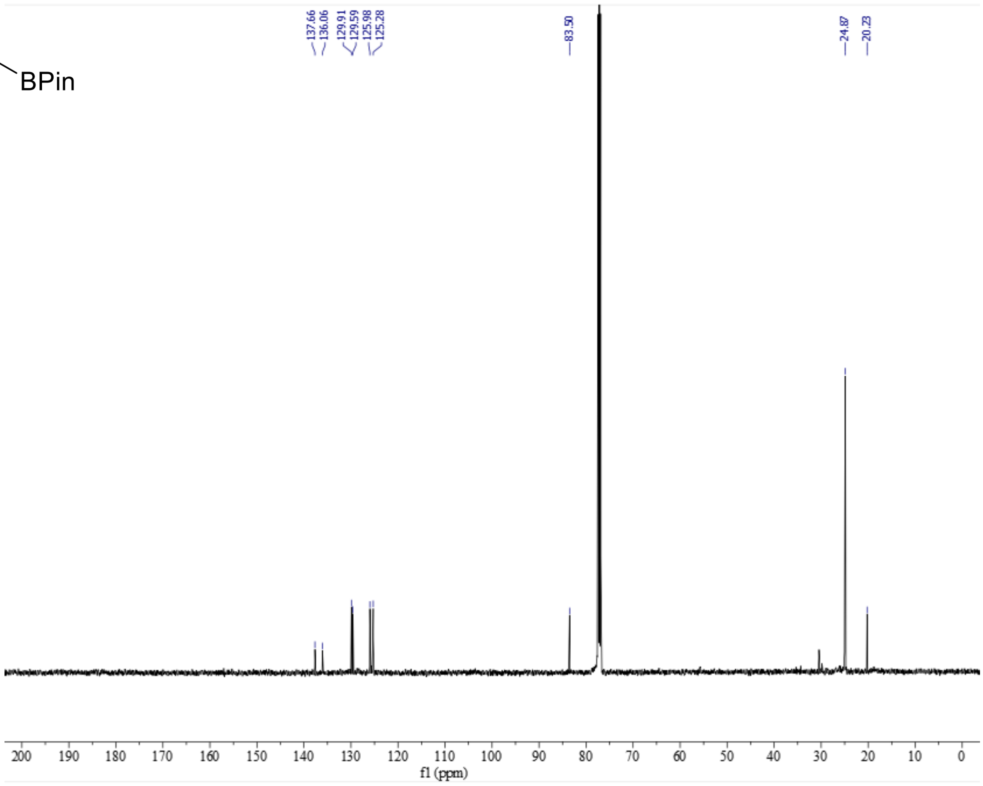

^{11}B NMR (96 MHz, $CDCl_3$)

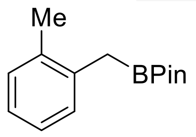

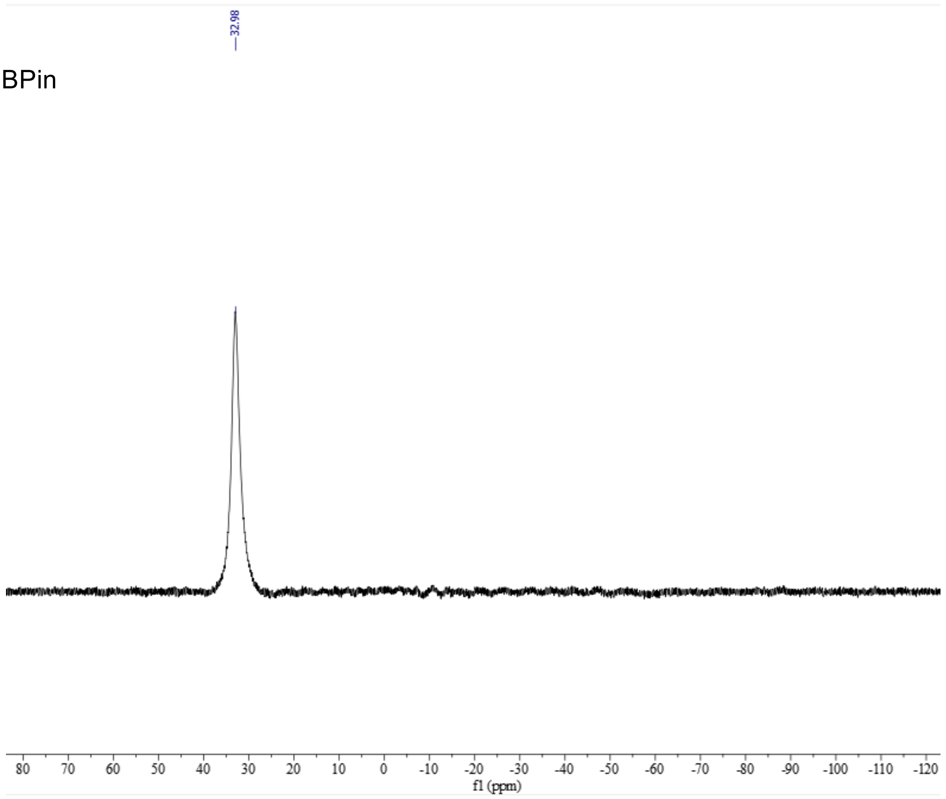

4,4,5,5-tetramethyl-2-(3-methylbenzyl)-1,3,2-dioxaborolane (7)

^{1}H NMR (500 MHz, $CDCl_3$)

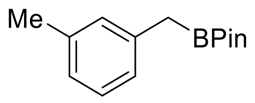

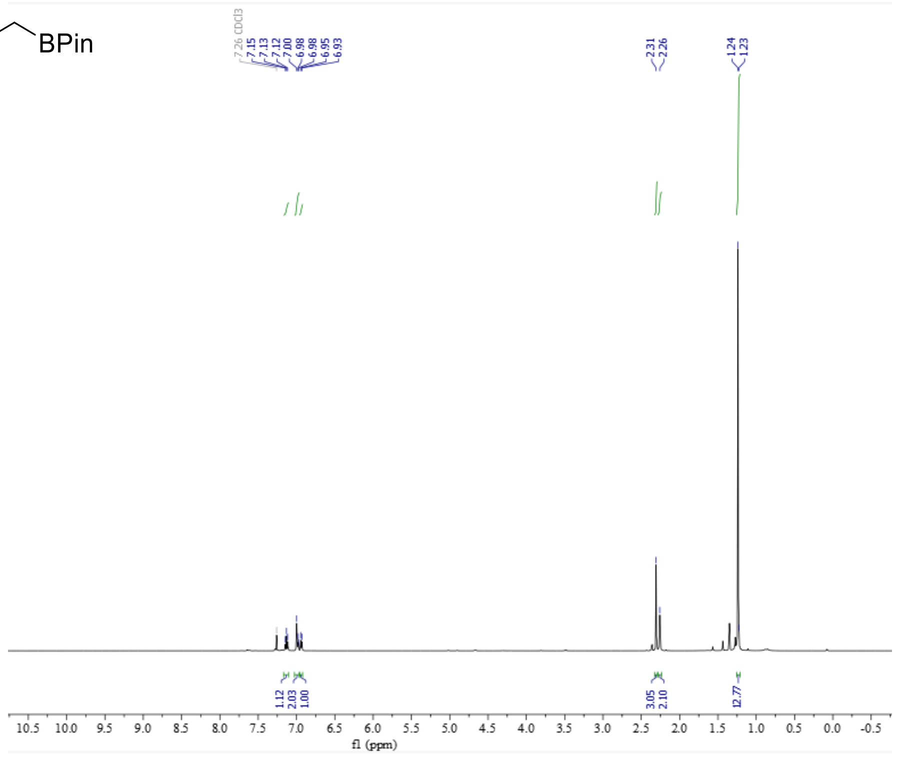

^{13}C NMR (126 MHz, CDCl$_3$)

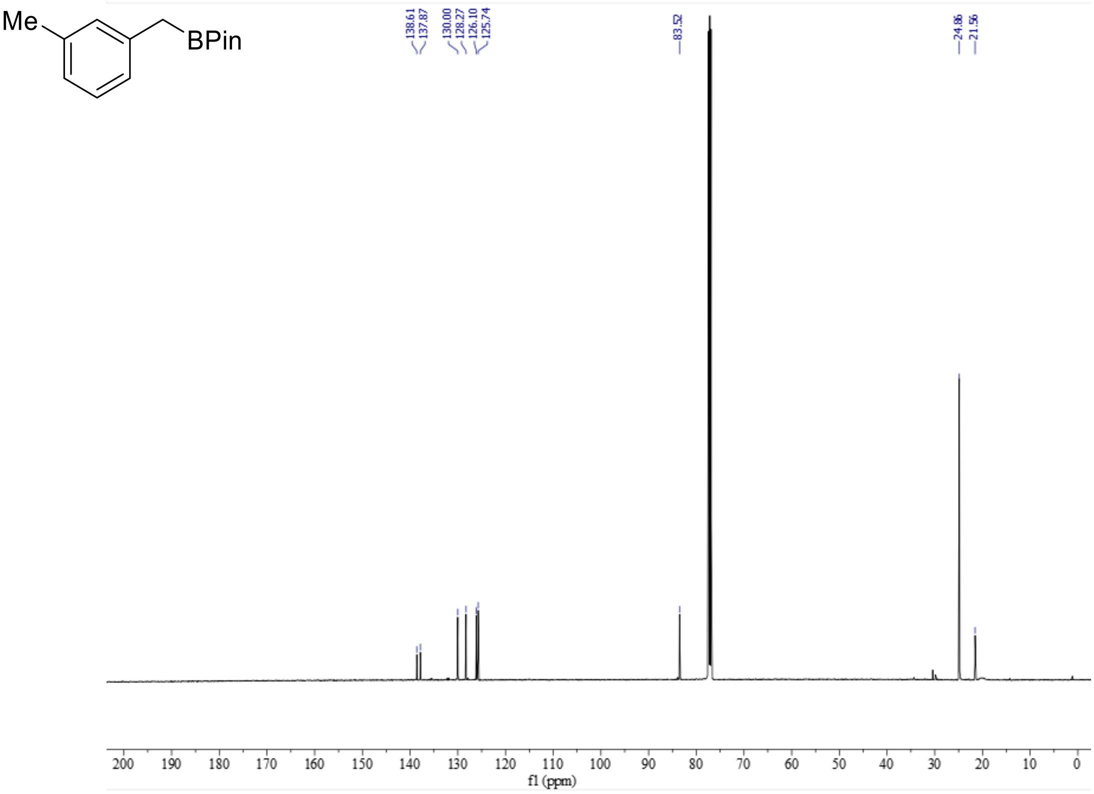

^{11}B NMR (96 MHz, CDCl$_3$)

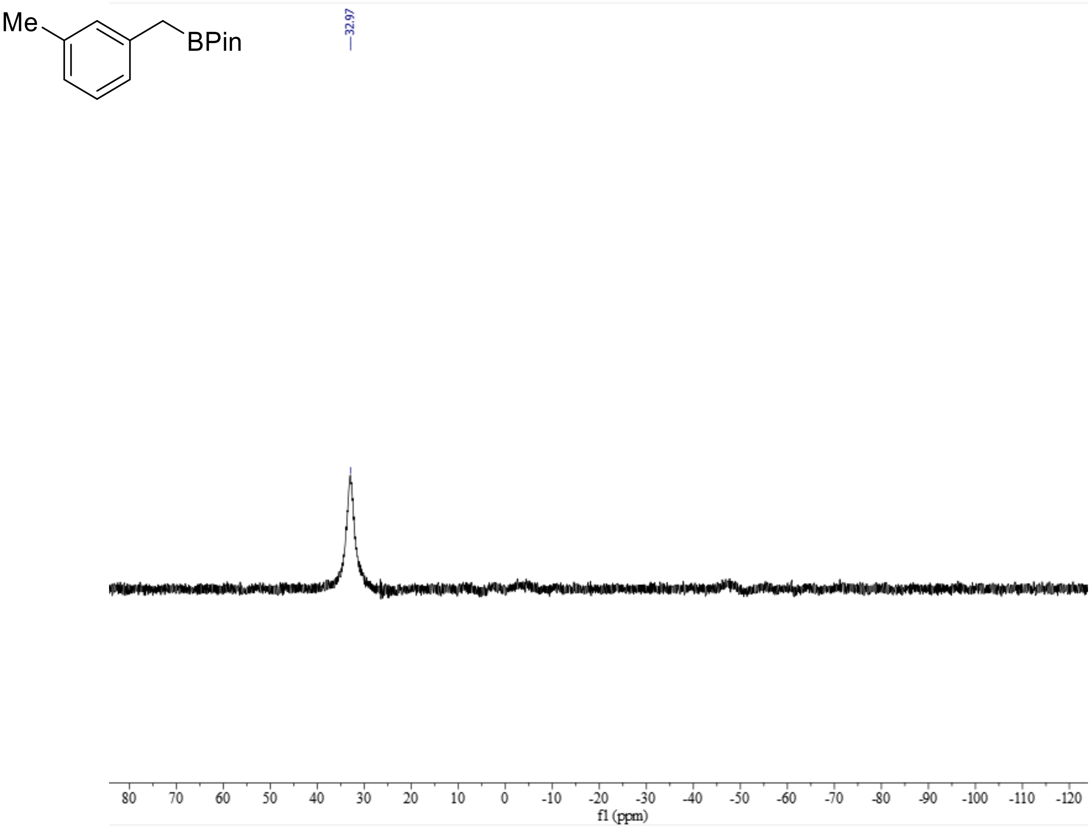

2-(3,5-dimethylbenzyl)-4,4,5,5-tetramethyl-1,3,2-dioxaborolane (8)

^{1}H NMR (500 MHz, $CDCl_3$)

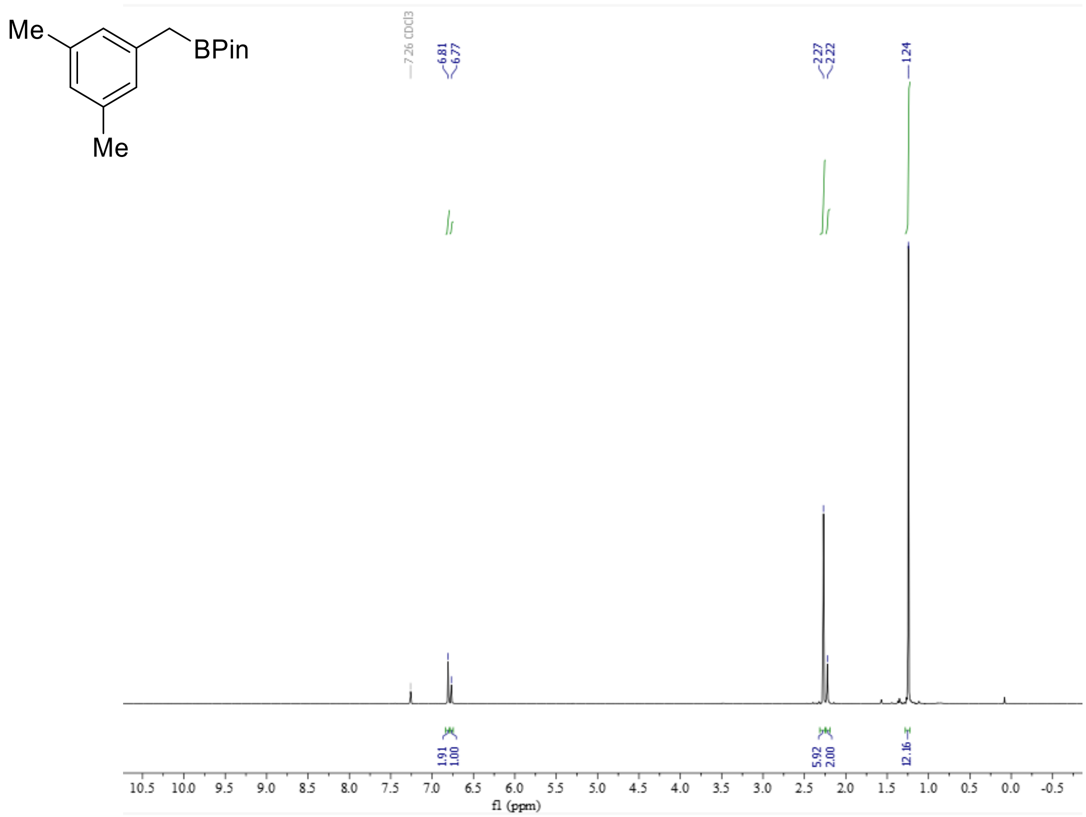

^{13}C NMR (126 MHz, $CDCl_3$)

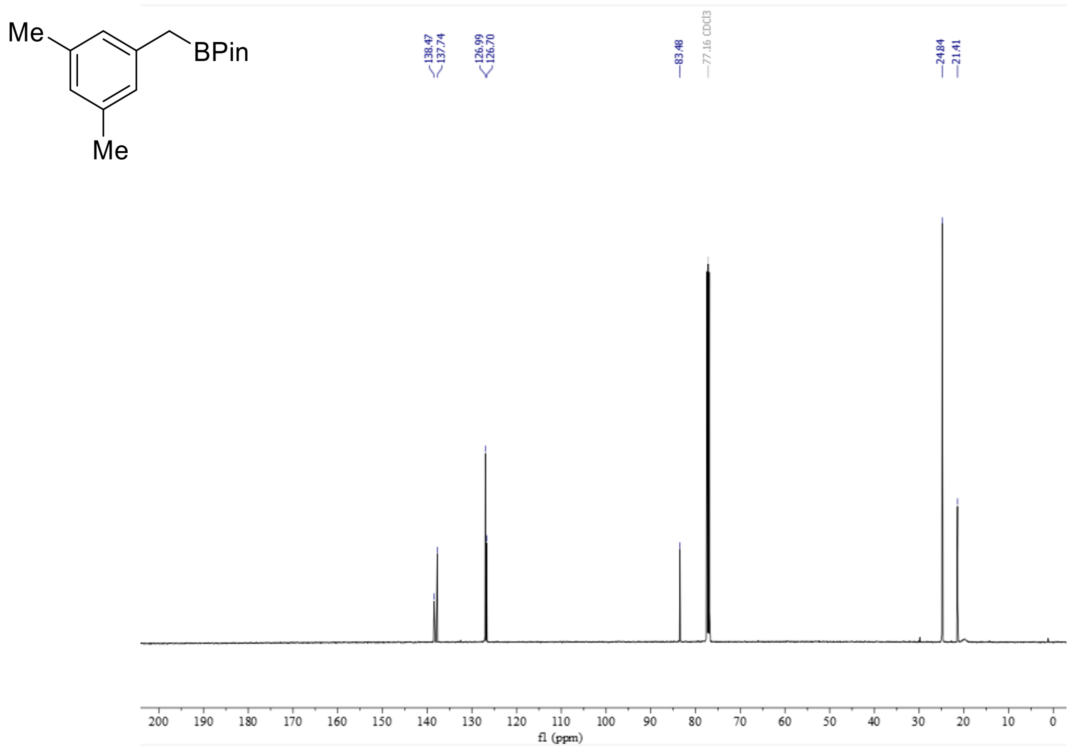

^{1}H-^{13}C-HSQC NMR – *α-boryl carbon atom highlighted*

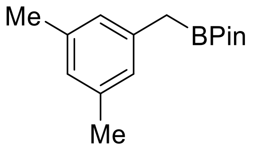

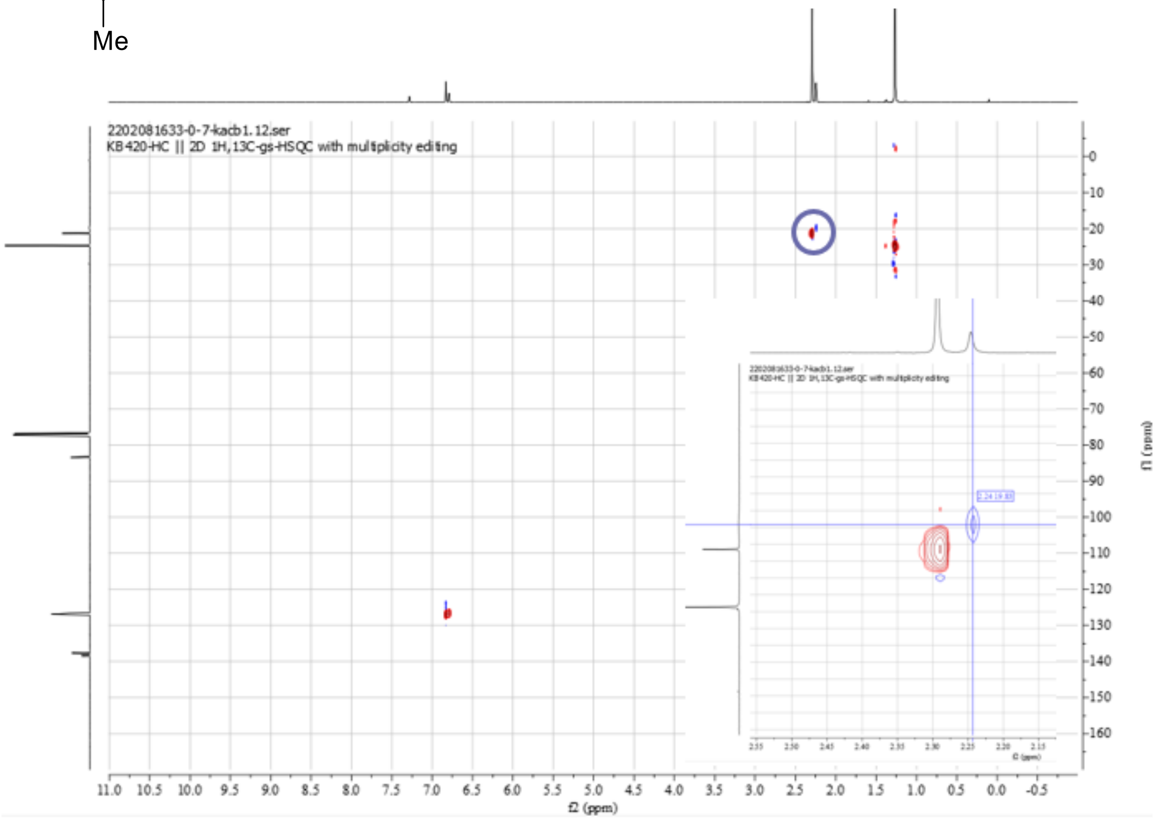

^{11}B NMR (96 MHz, $CDCl_3$)

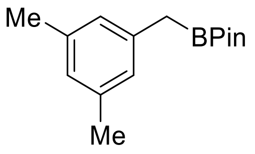

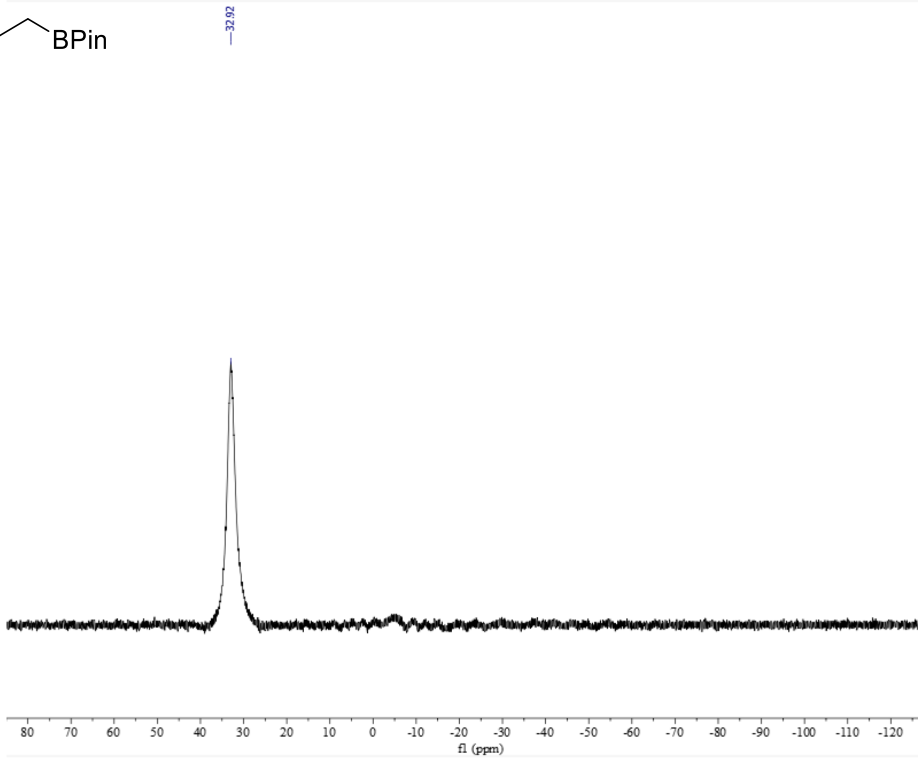

2-(2,6-dimethylbenzyl)-4,4,5,5-tetramethyl-1,3,2-dioxaborolane (9)

^{1}H NMR (500 MHz, $CDCl_3$)

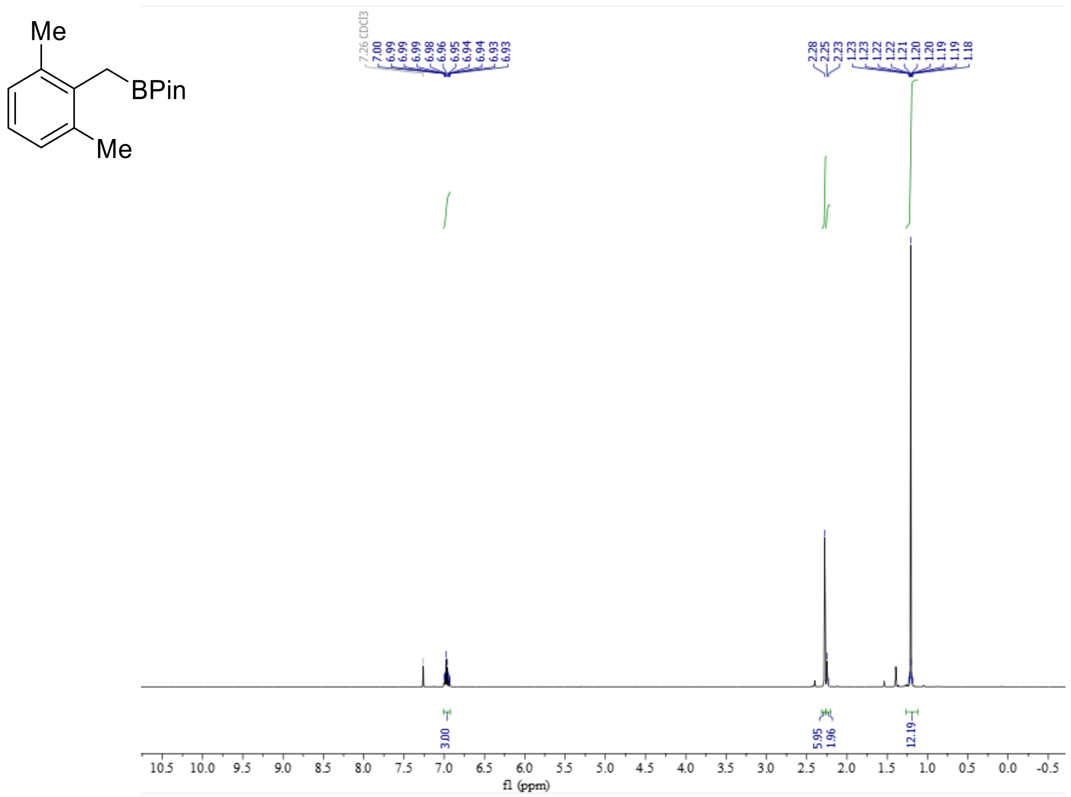

^{13}C NMR (126 MHz, $CDCl_3$)

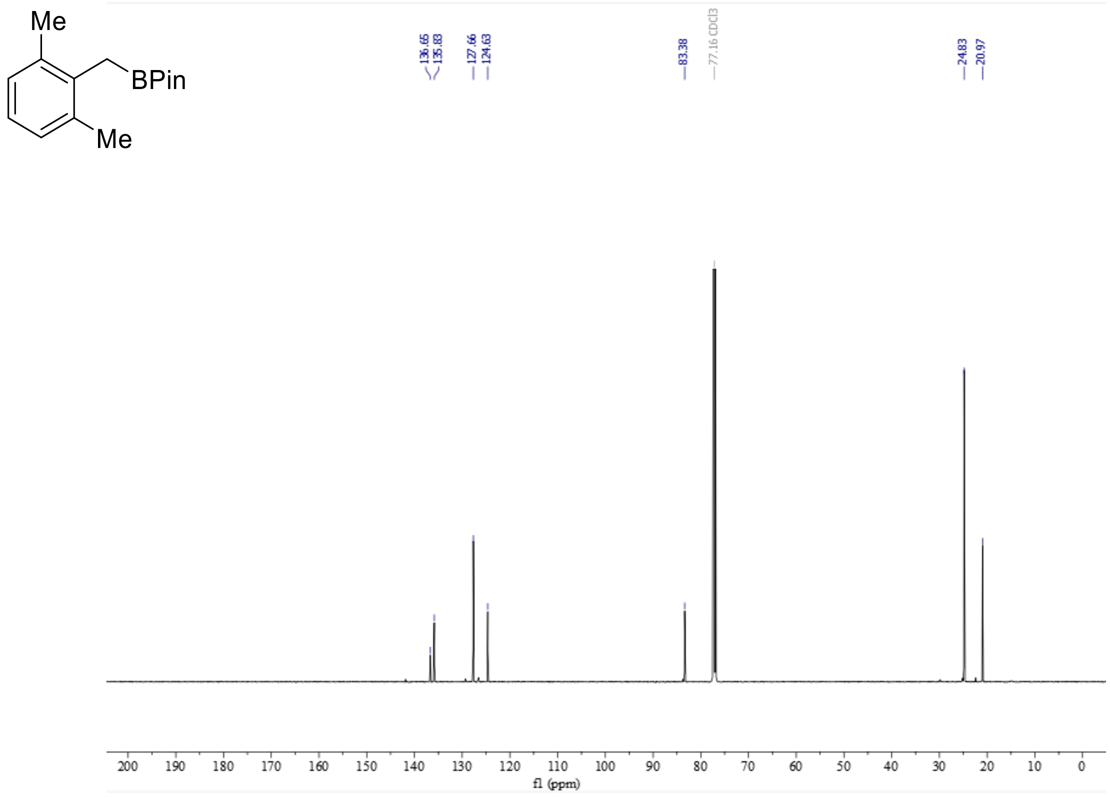

H-^{13}C-HSQC NMR – *α-boryl carbon atom highlighted*

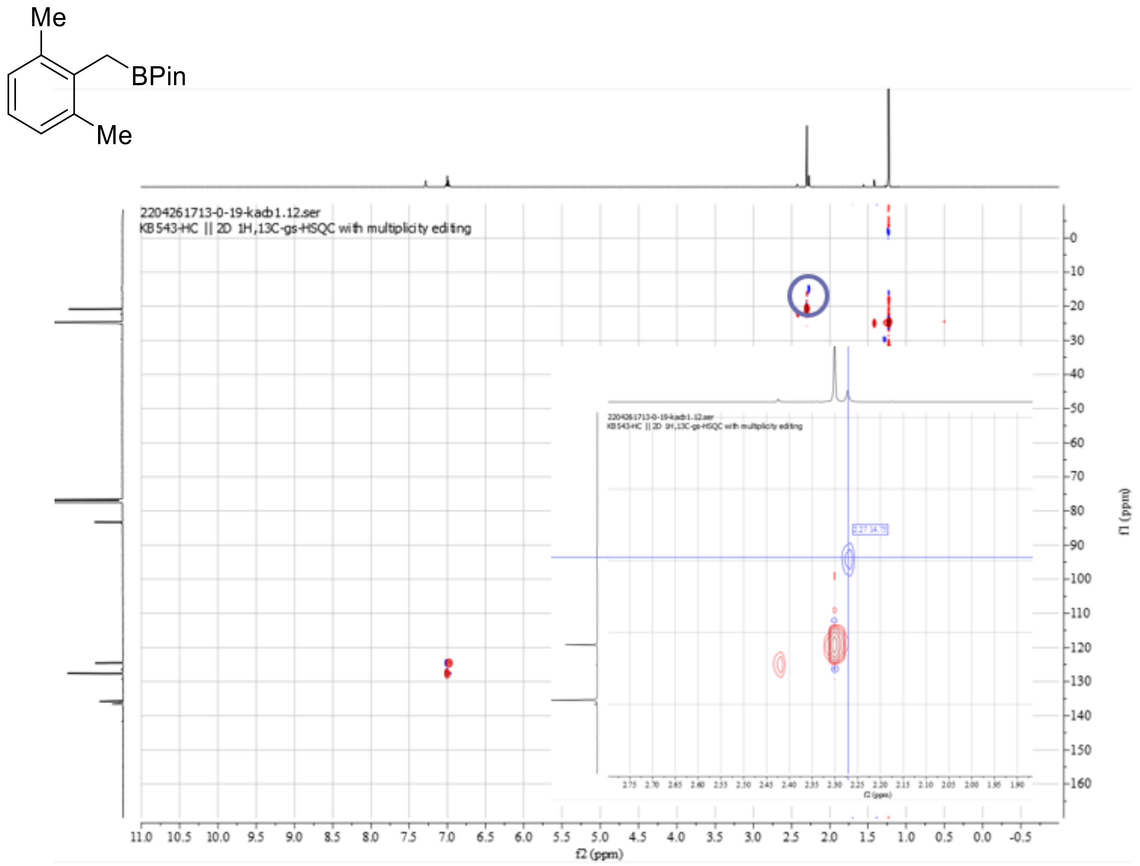

^{11}B NMR (96 MHz, $CDCl_3$)

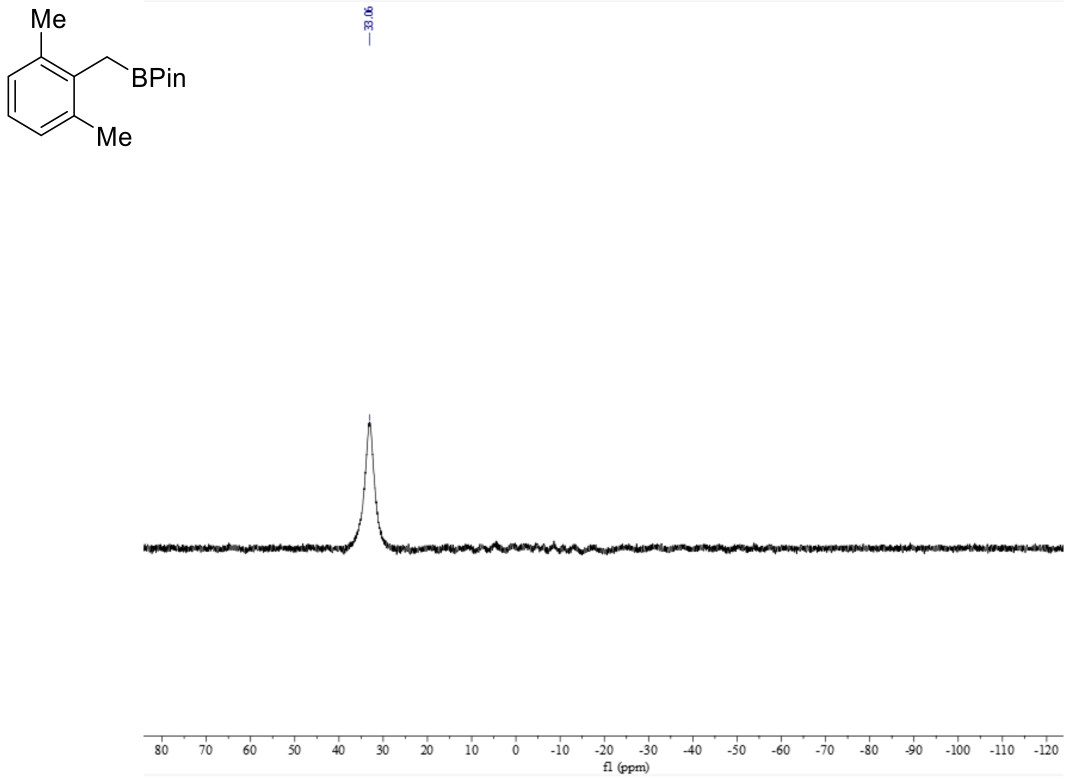

4,4,5,5-tetramethyl-2-(naphthalen-2-yl)-1,3,2-dioxaborolane (10)

^{1}H NMR (500 MHz, $CDCl_3$)

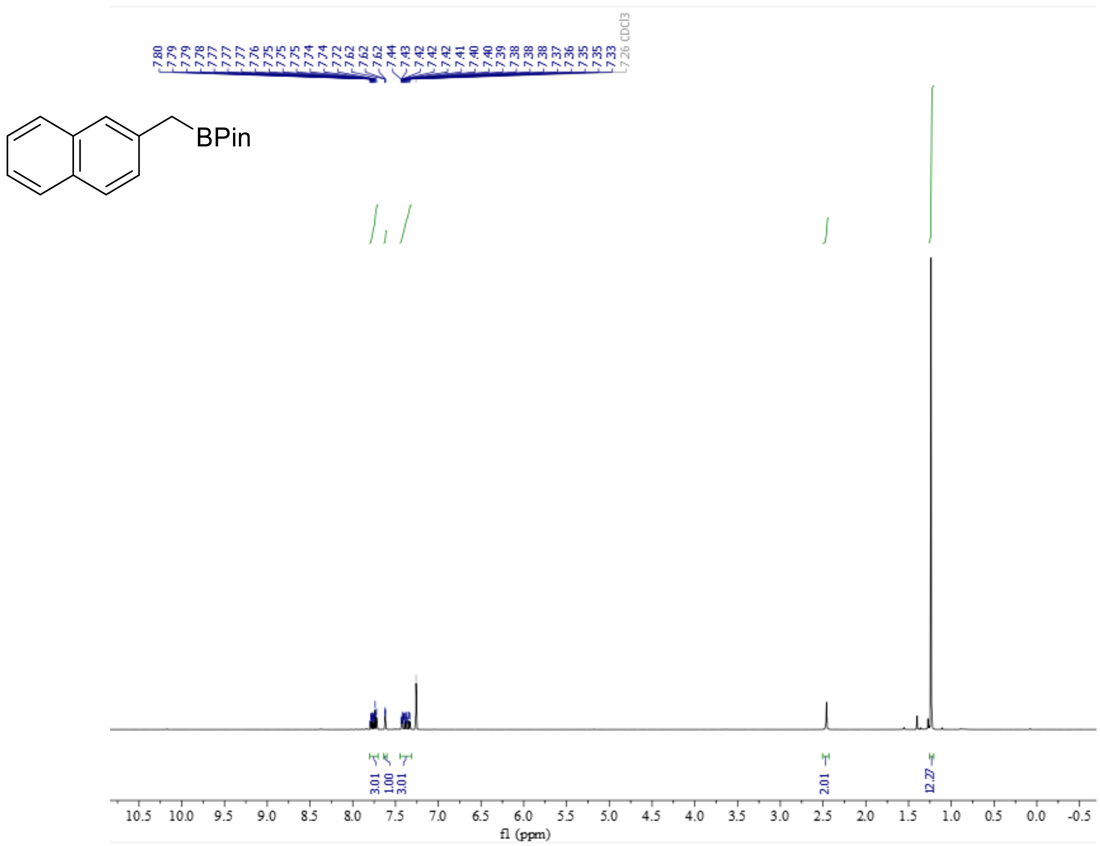

^{13}C NMR (126 MHz, $CDCl_3$)

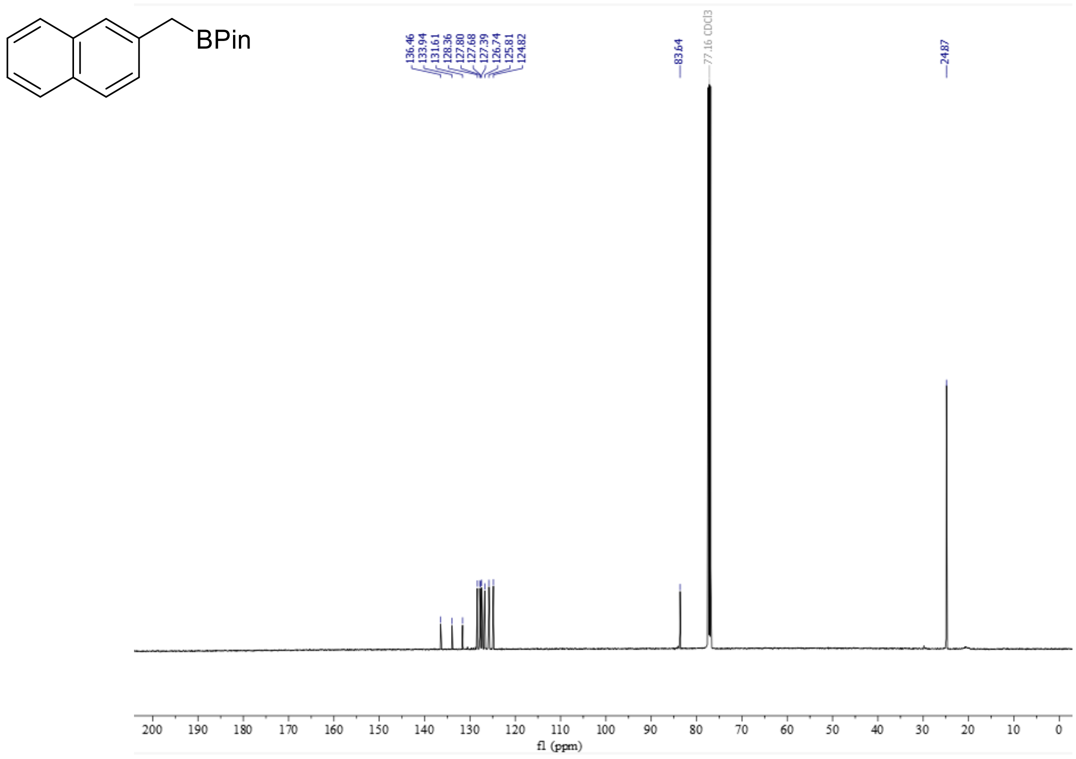

^{1}H-^{13}C-HSQC NMR – *α-boryl carbon atom highlighted*

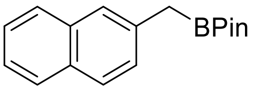

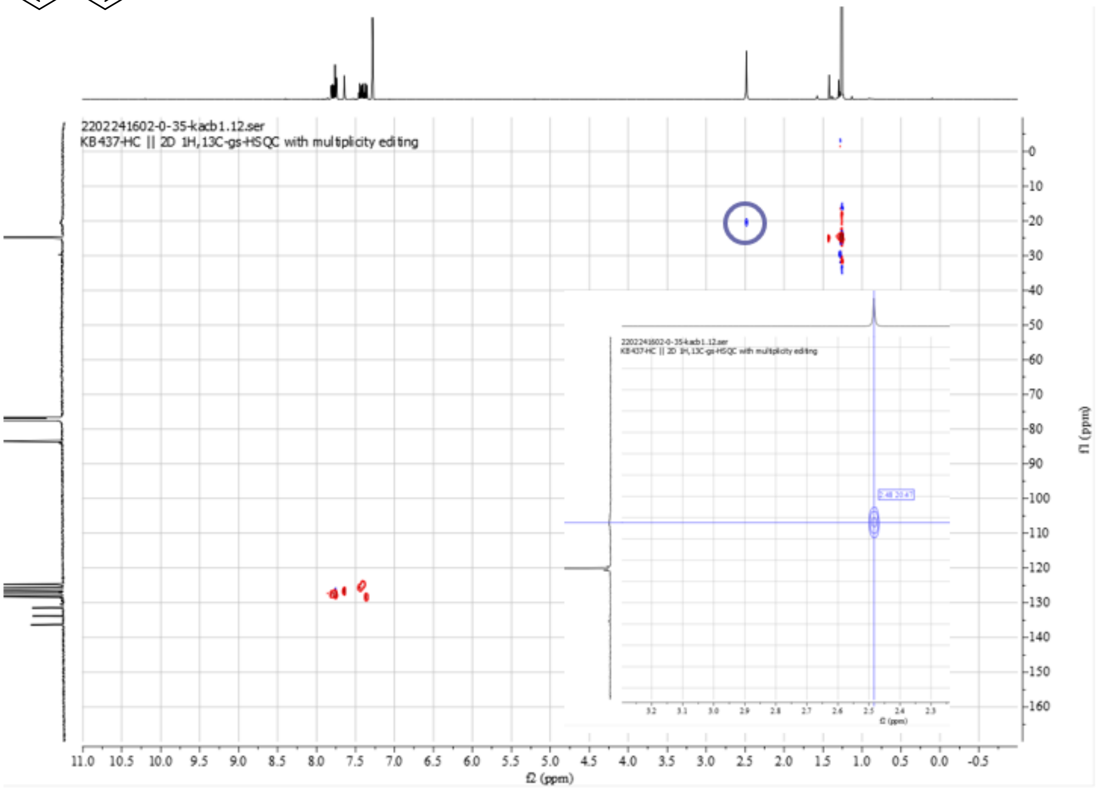

^{11}B NMR (96 MHz, $CDCl_3$)

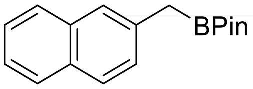

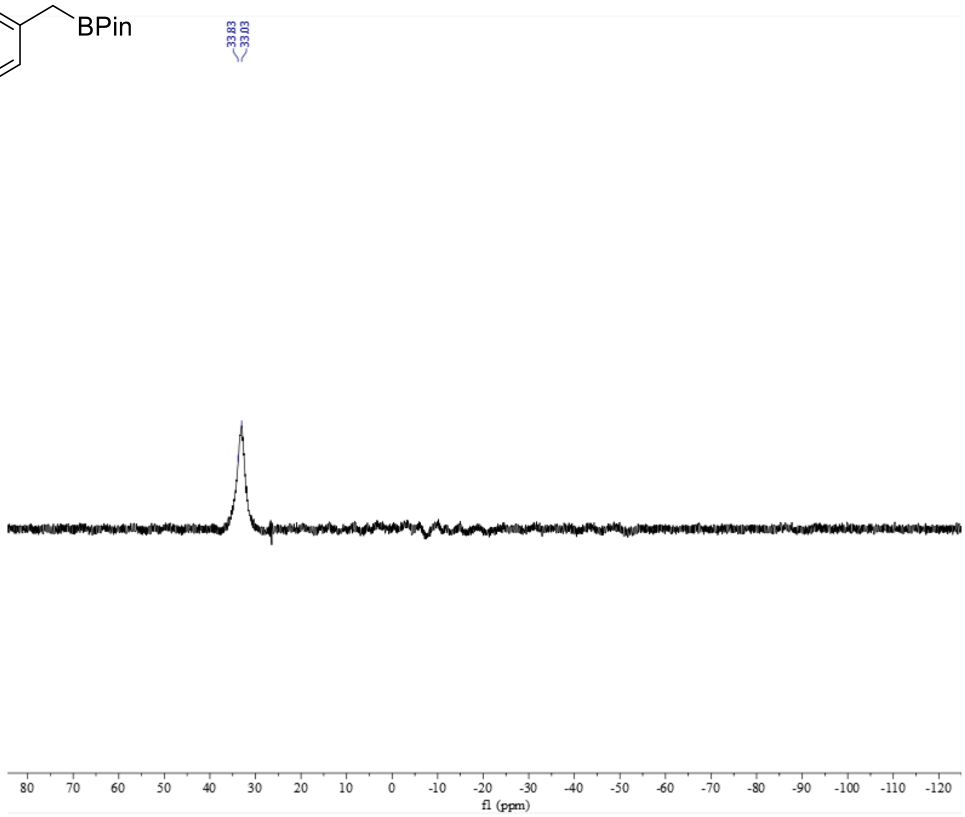

2-([1,1'-biphenyl]-4-ylmethyl)-4,4,5,5-tetramethyl-1,3,2-dioxaborolane (11)

^{1}H NMR (500 MHz, $CDCl_3$)

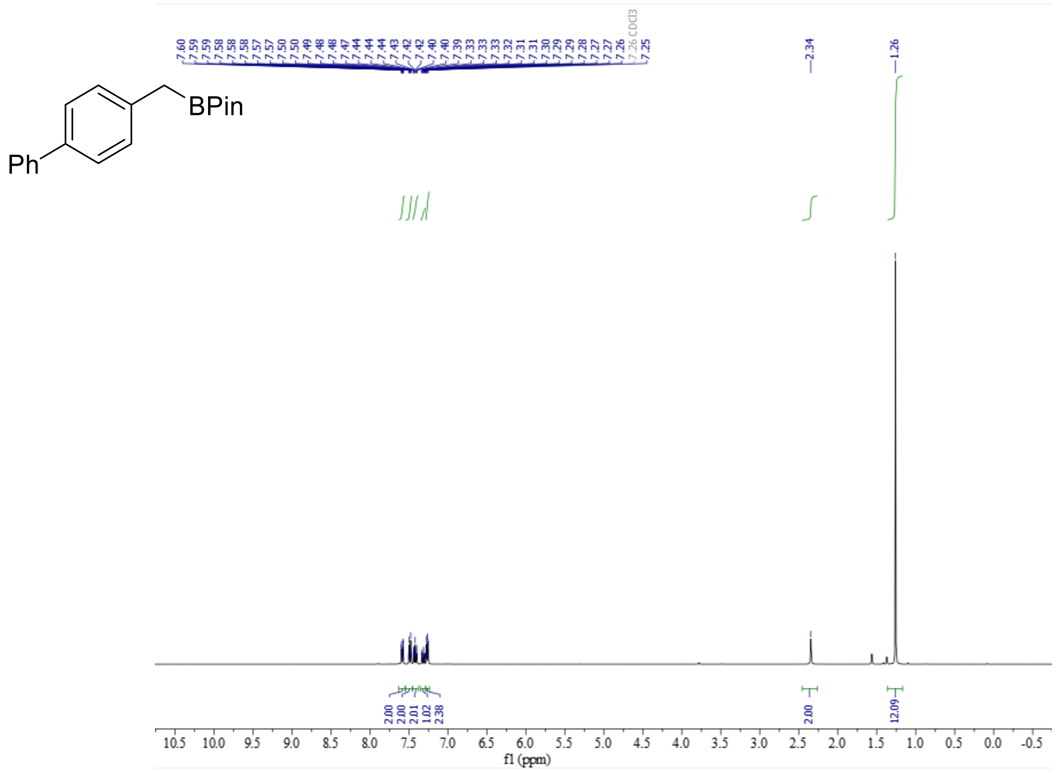

^{13}C NMR (126 MHz, $CDCl_3$)

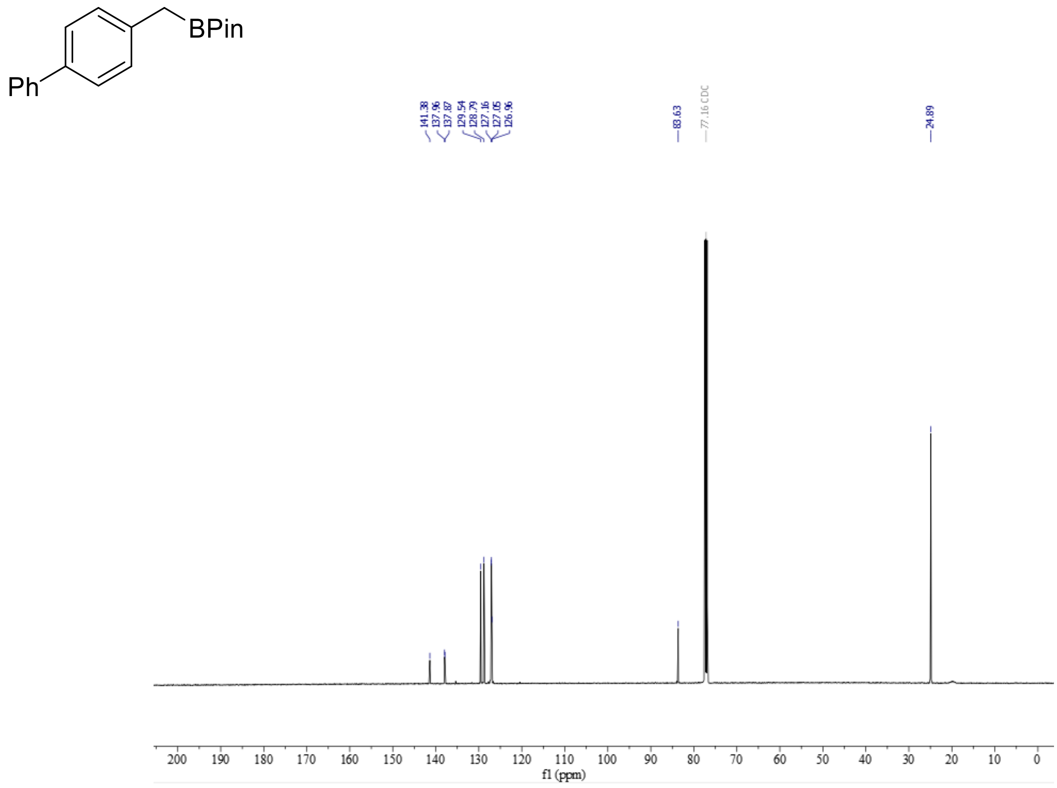

^{1}H-^{13}C-HSQC NMR – *α-boryl carbon atom highlighted*

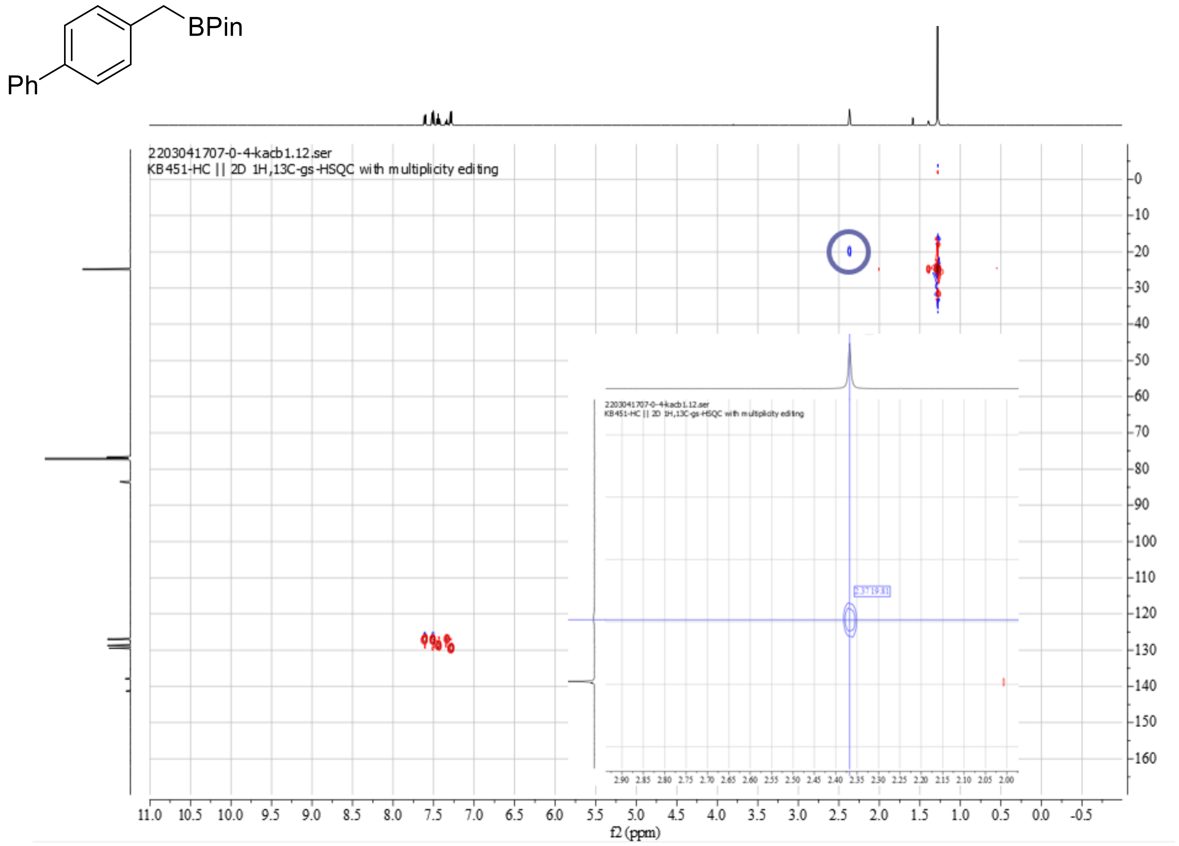

^{11}B NMR (96 MHz, CDCl$_3$)

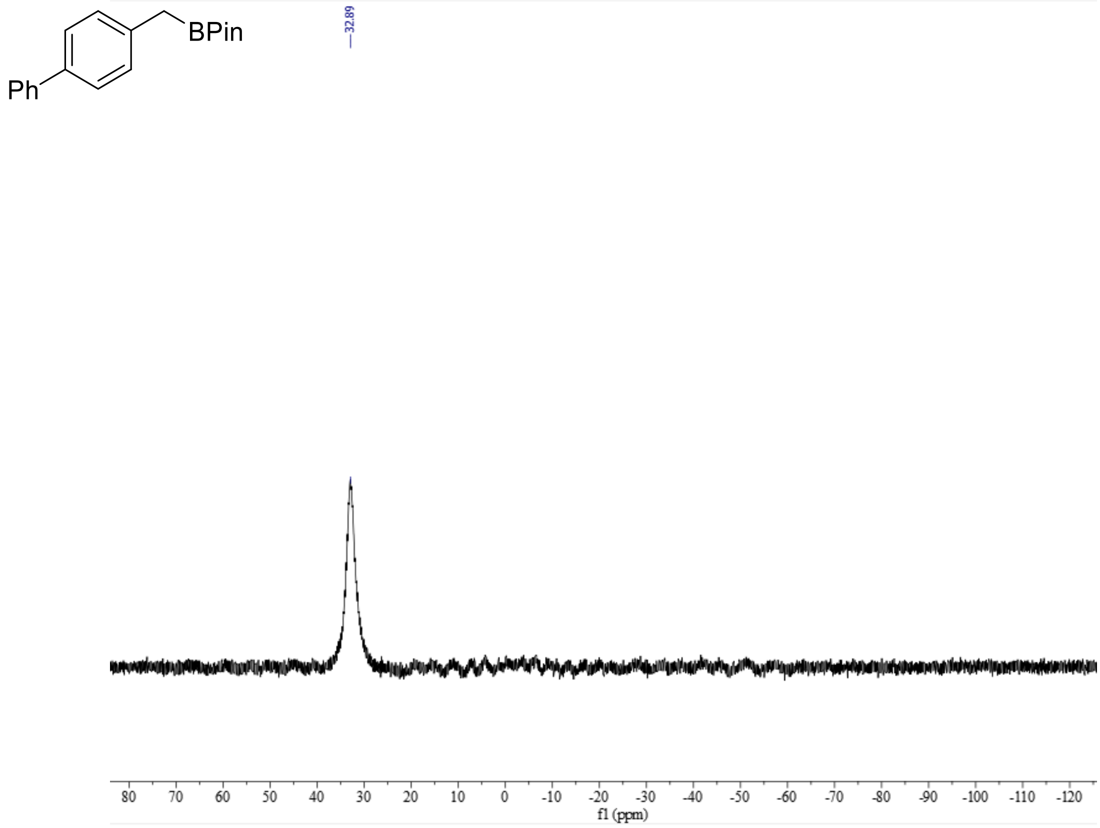

2-([1,1'-biphenyl]-2-ylmethyl)-4,4,5,5-tetramethyl-1,3,2-dioxaborolane (12)

^{1}H NMR (500 MHz, $CDCl_3$)

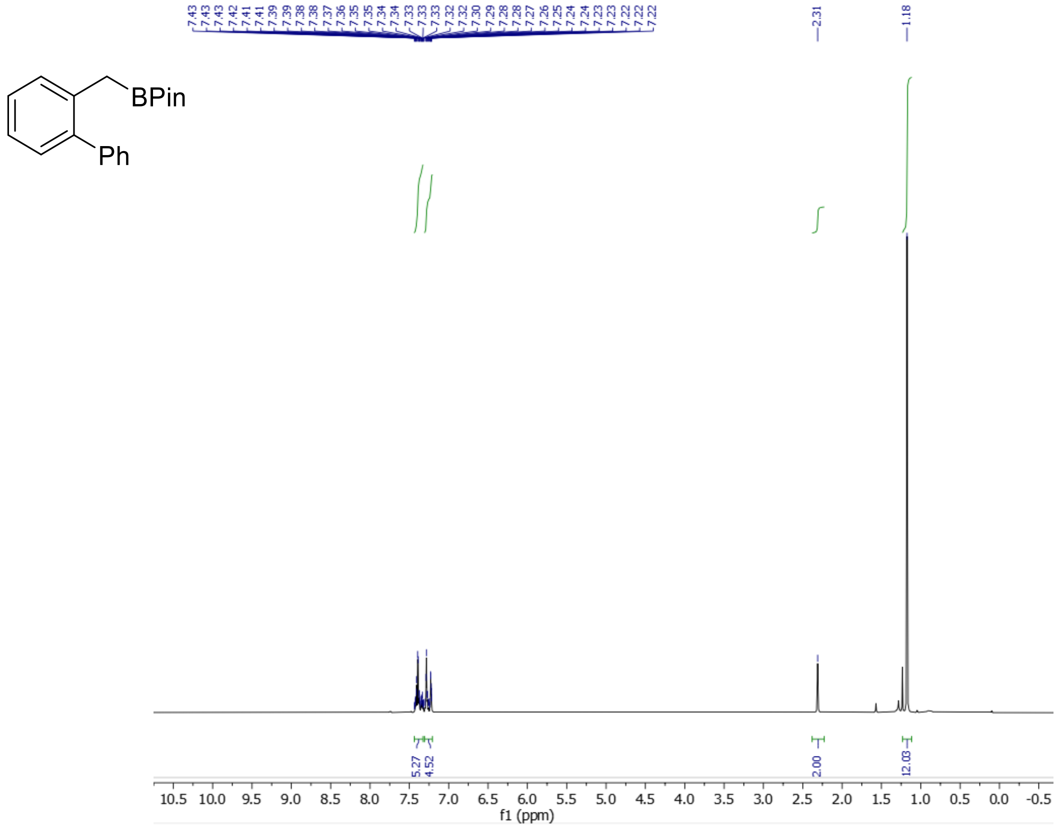

^{13}C NMR (126 MHz, $CDCl_3$)

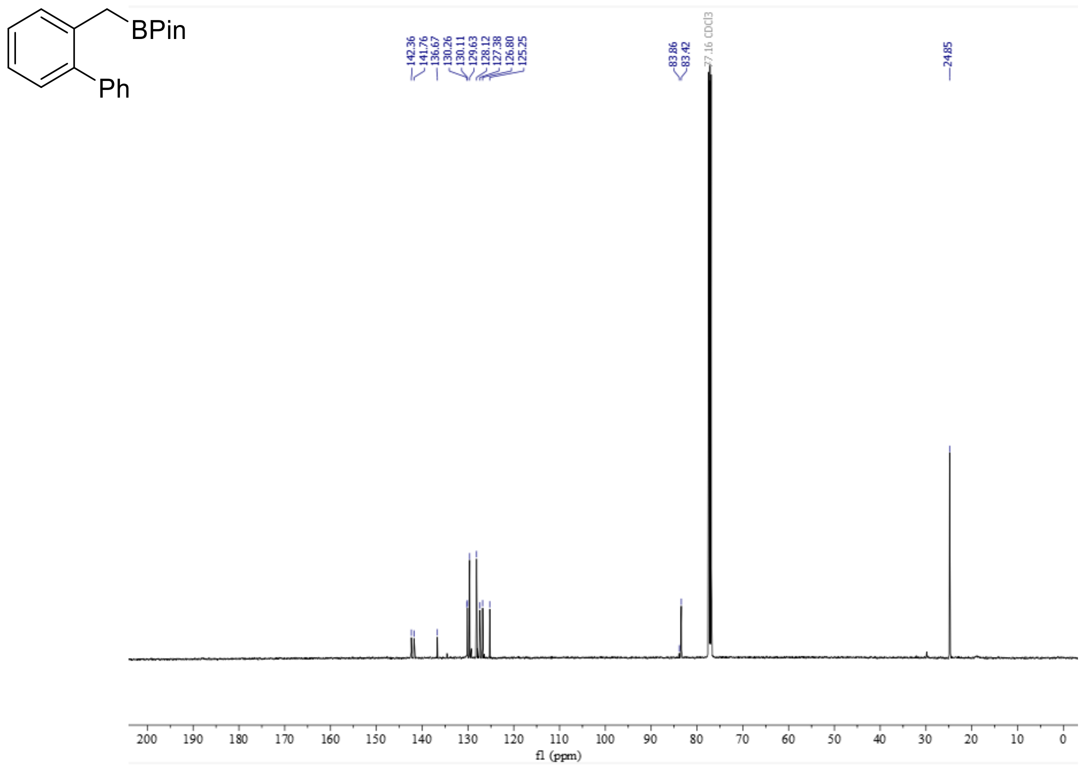

^{1}H-^{13}C-HSQC NMR – *α-boryl carbon atom highlighted*

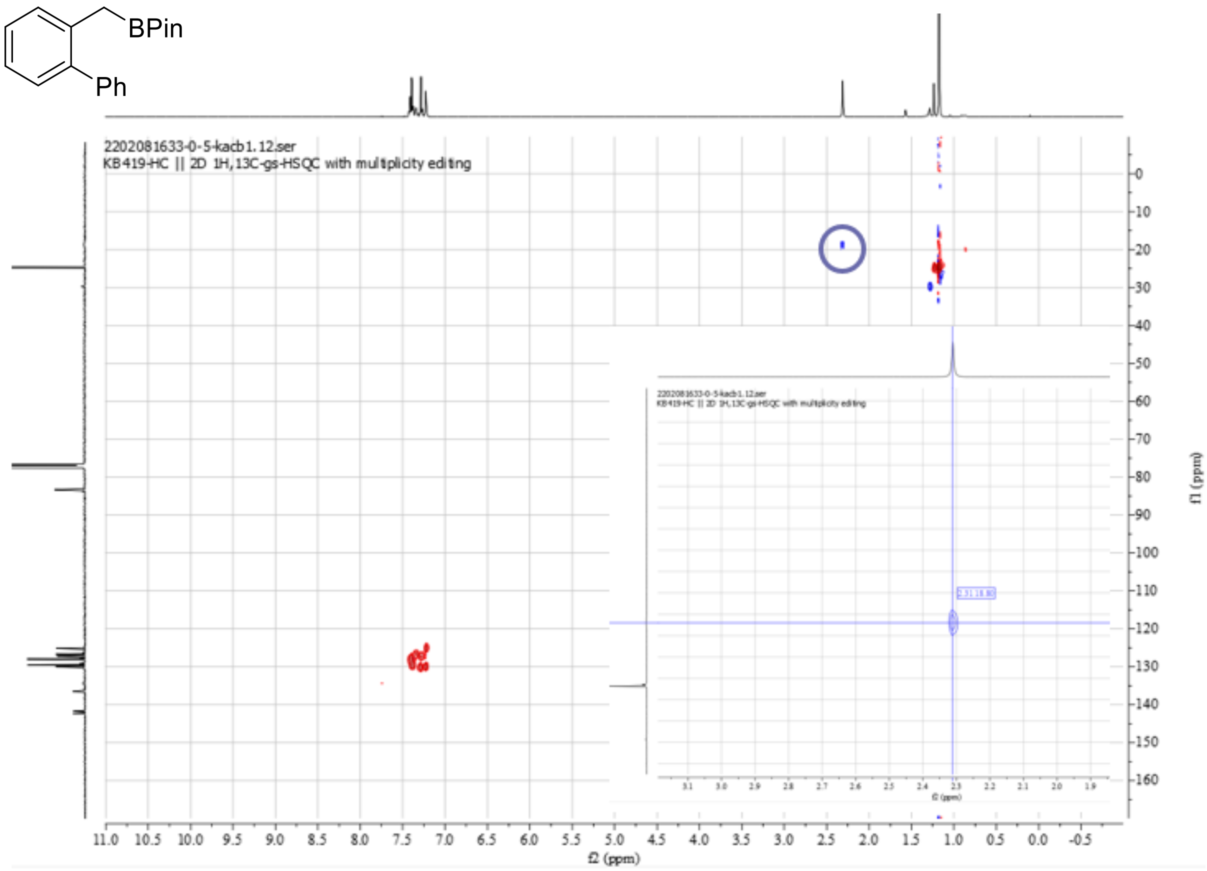

^{11}B NMR (96 MHz, $CDCl_3$)

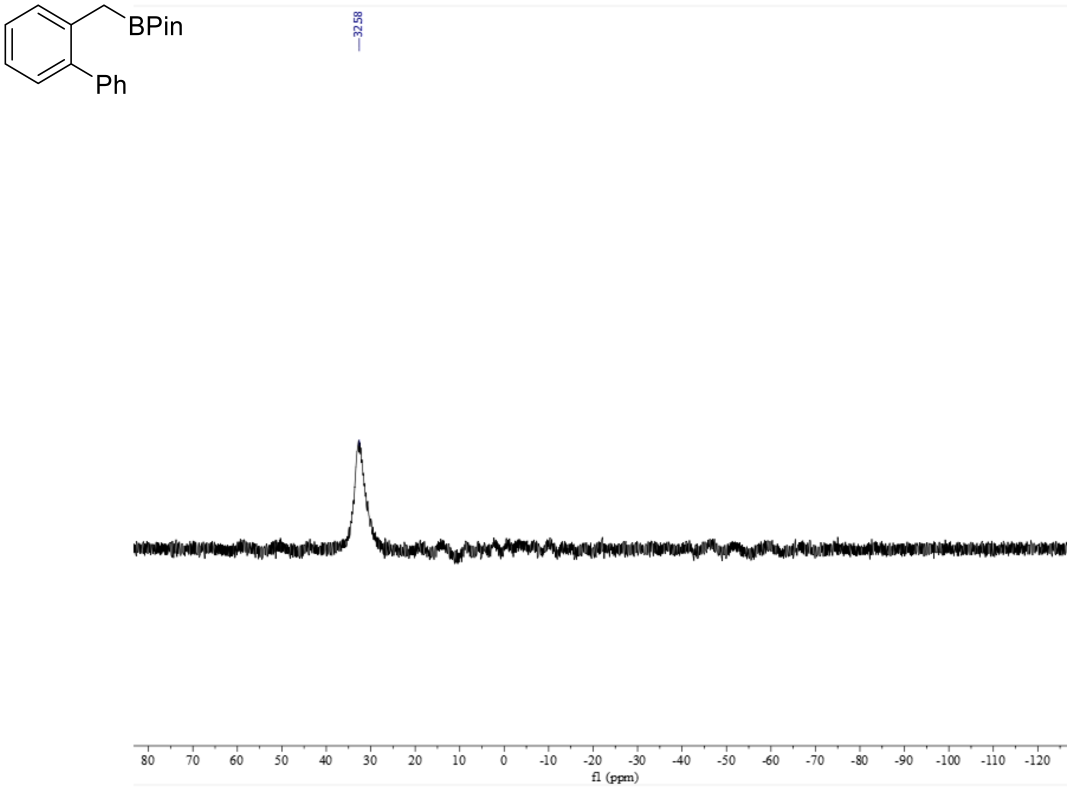

***tert*-Butyldimethyl(4-((4,4,5,5-tetramethyl-1,3,2-dioxaborolan-2-yl)methyl)phenoxy)silane (13)**

^{1}H NMR (500 MHz, $CDCl_3$)

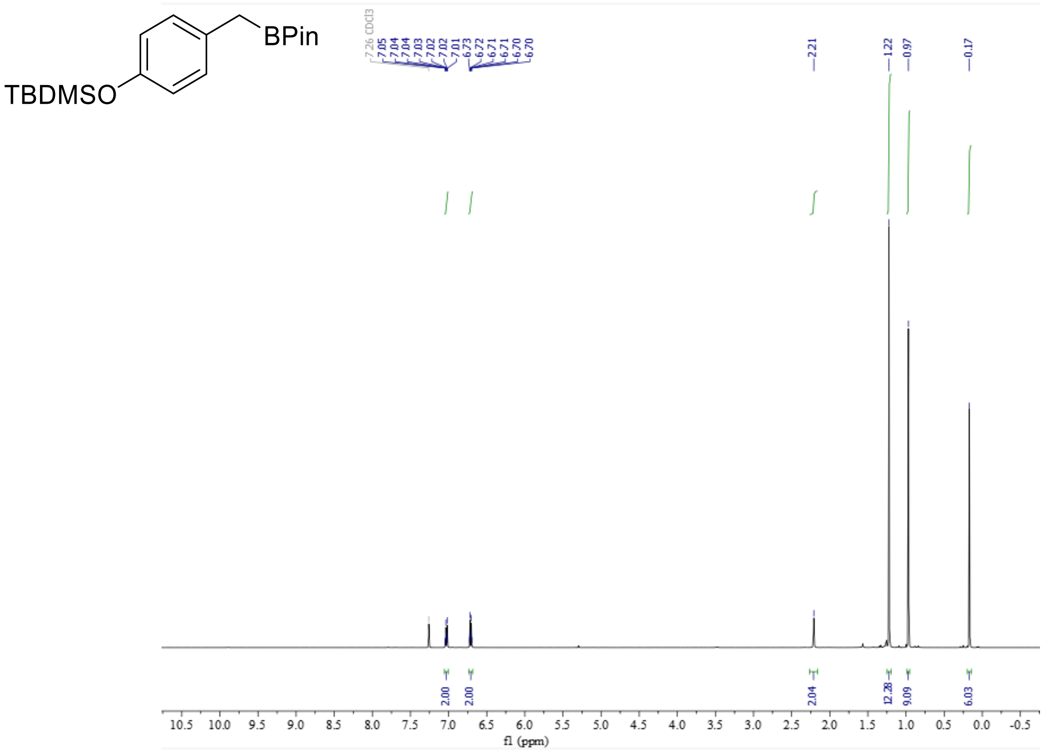

^{13}C NMR (126 MHz, $CDCl_3$)

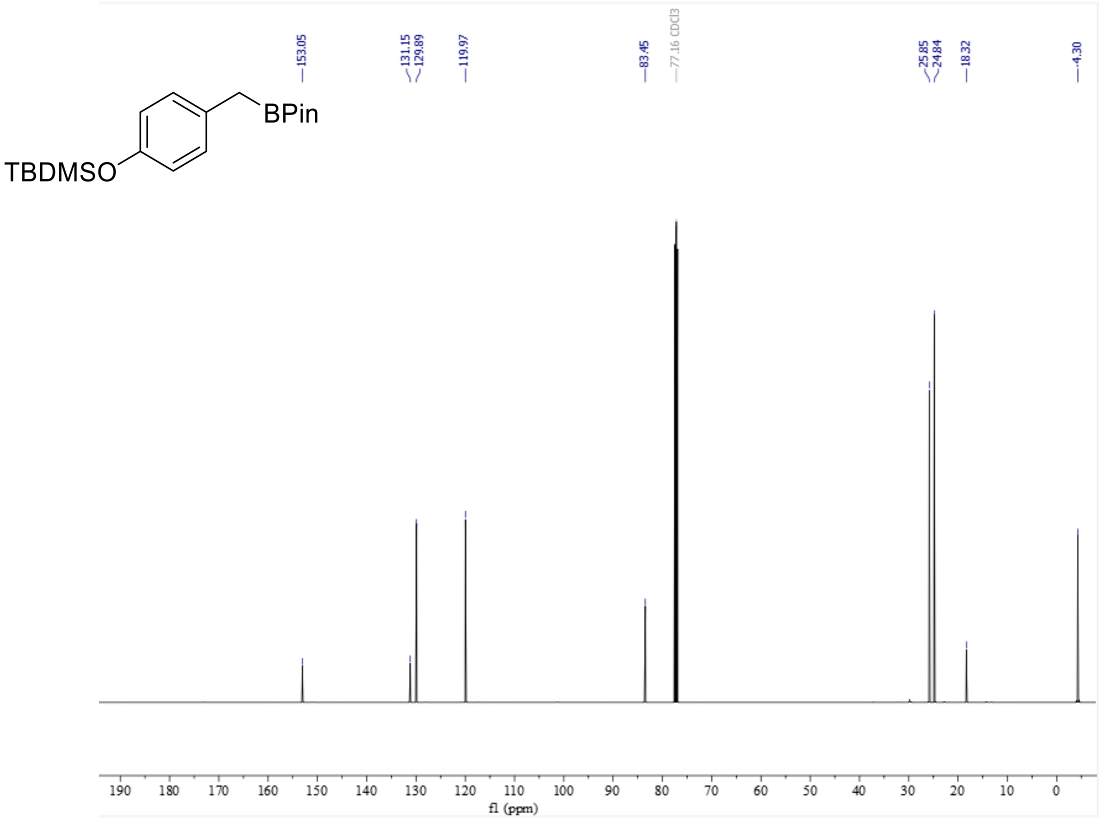

^{1}H-^{13}C-HSQC NMR – *α-boryl carbon atom highlighted*

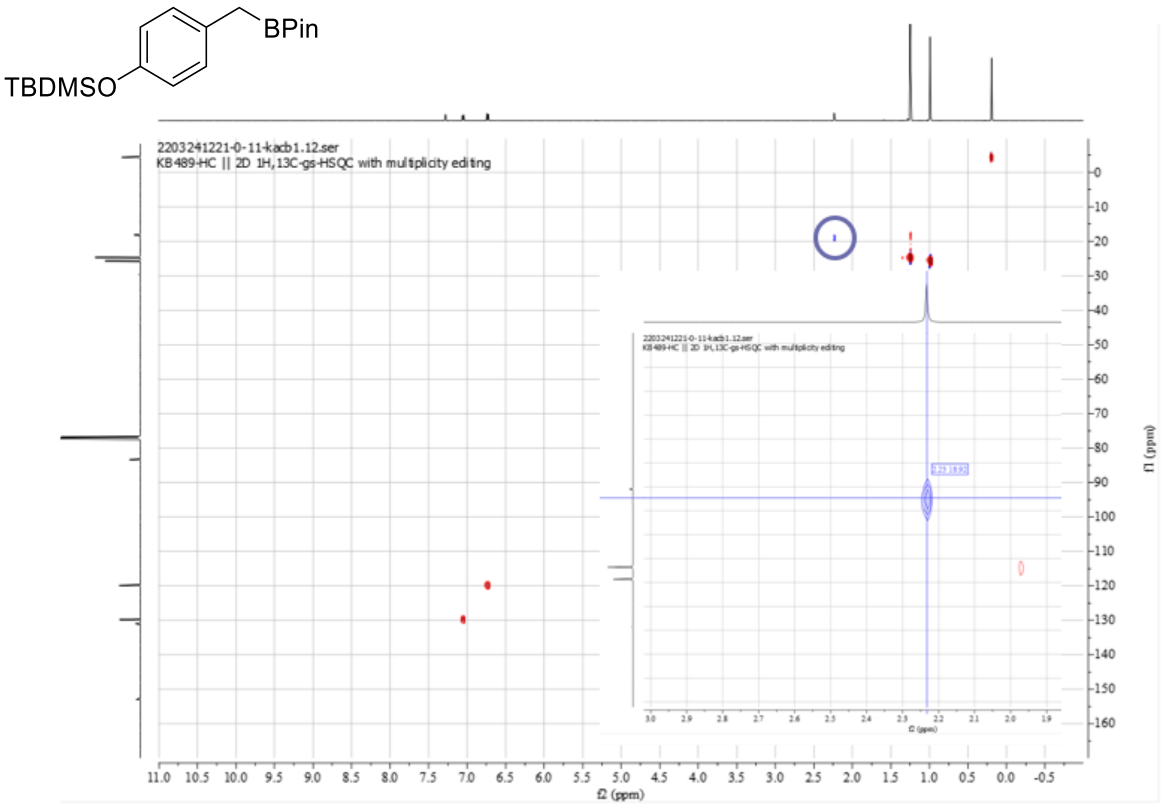

^{11}B NMR (96 MHz, $CDCl_3$)

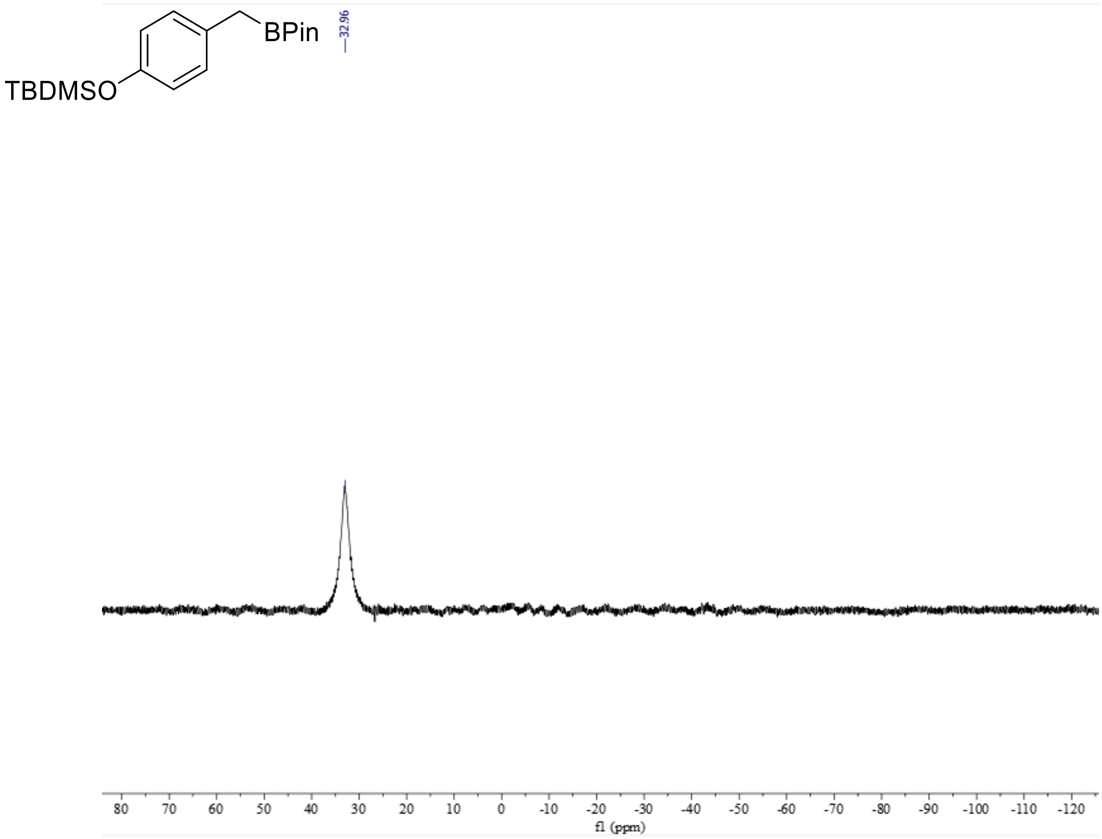

***tert*-butyldimethyl(2-((4,4,5,5-tetramethyl-1,3,2-dioxaborolan-2-yl)methyl)phenoxy)silane (14)**

^{1}H NMR (500 MHz, $CDCl_3$)

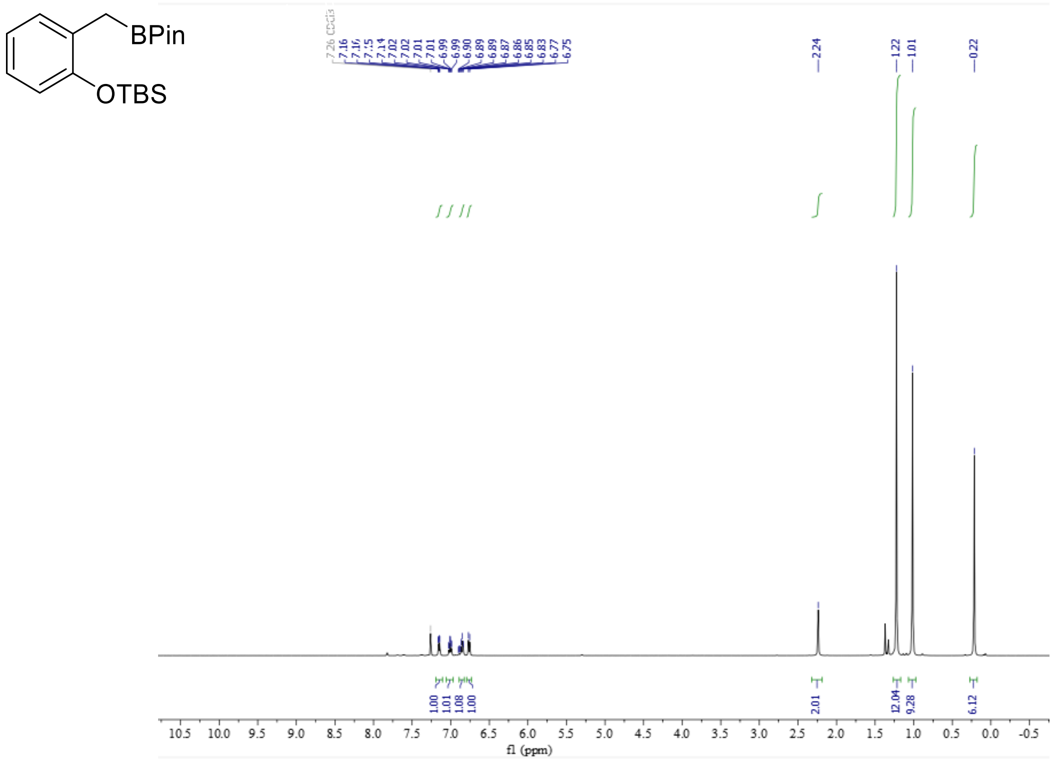

^{13}C NMR (126 MHz, $CDCl_3$)

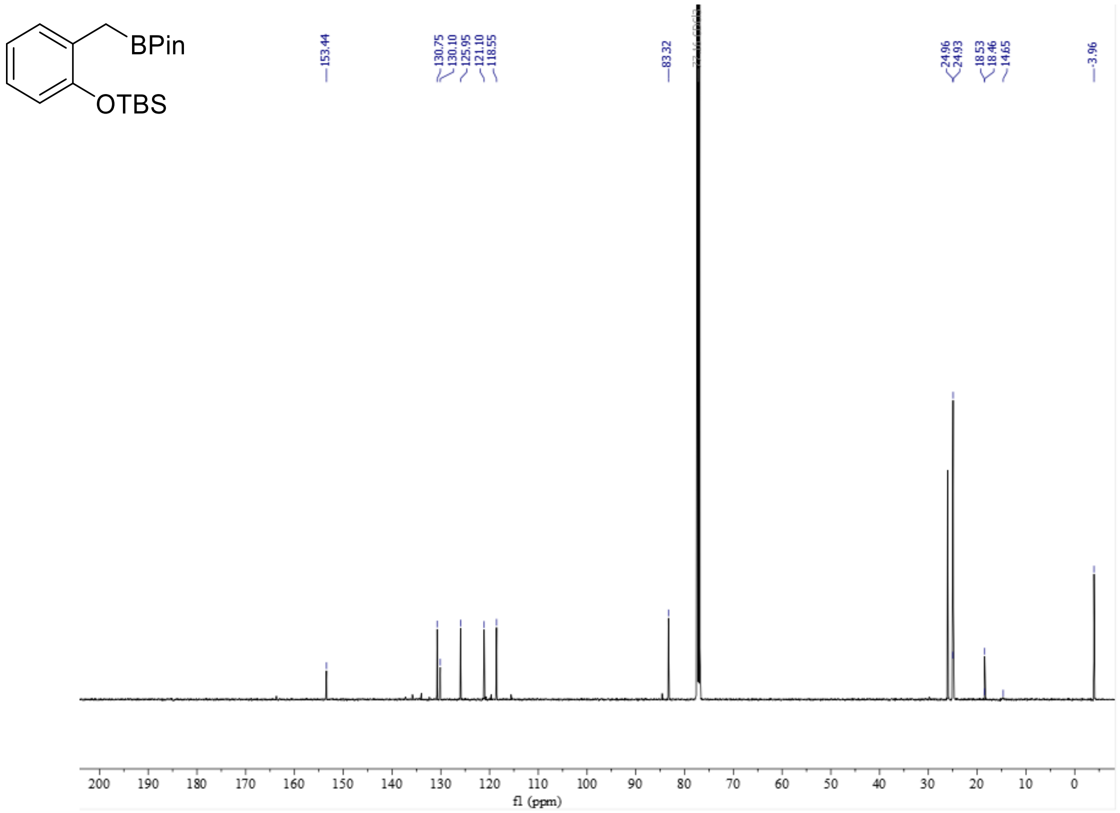

^{11}B NMR (96 MHz, $CDCl_3$)

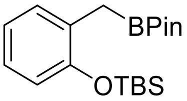

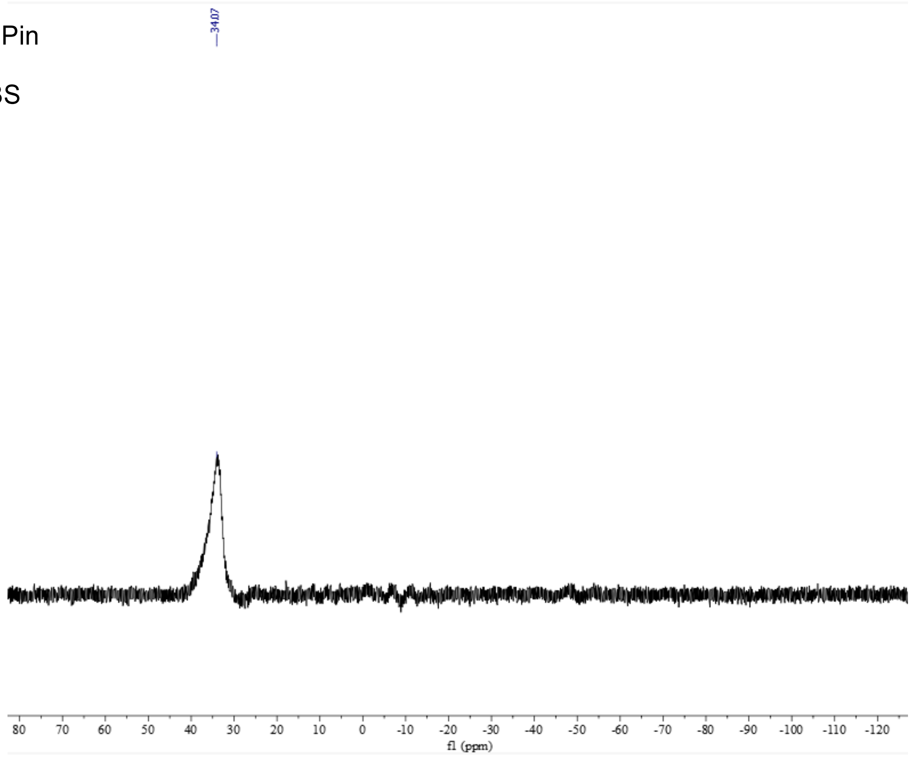

4,4,5,5-tetramethyl-2-(2-vinylbenzyl)-1,3,2-dioxaborolane (15)

^{1}H NMR (500 MHz, $CDCl_3$)

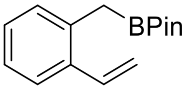

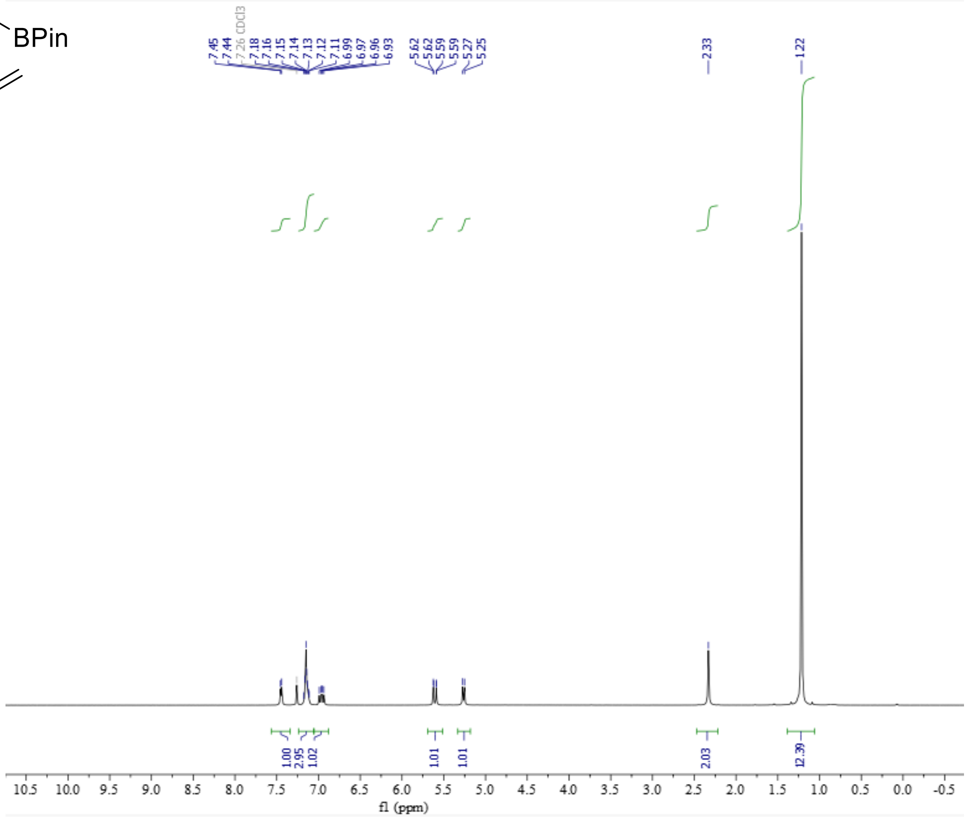

^{13}C NMR (126 MHz, $CDCl_3$)

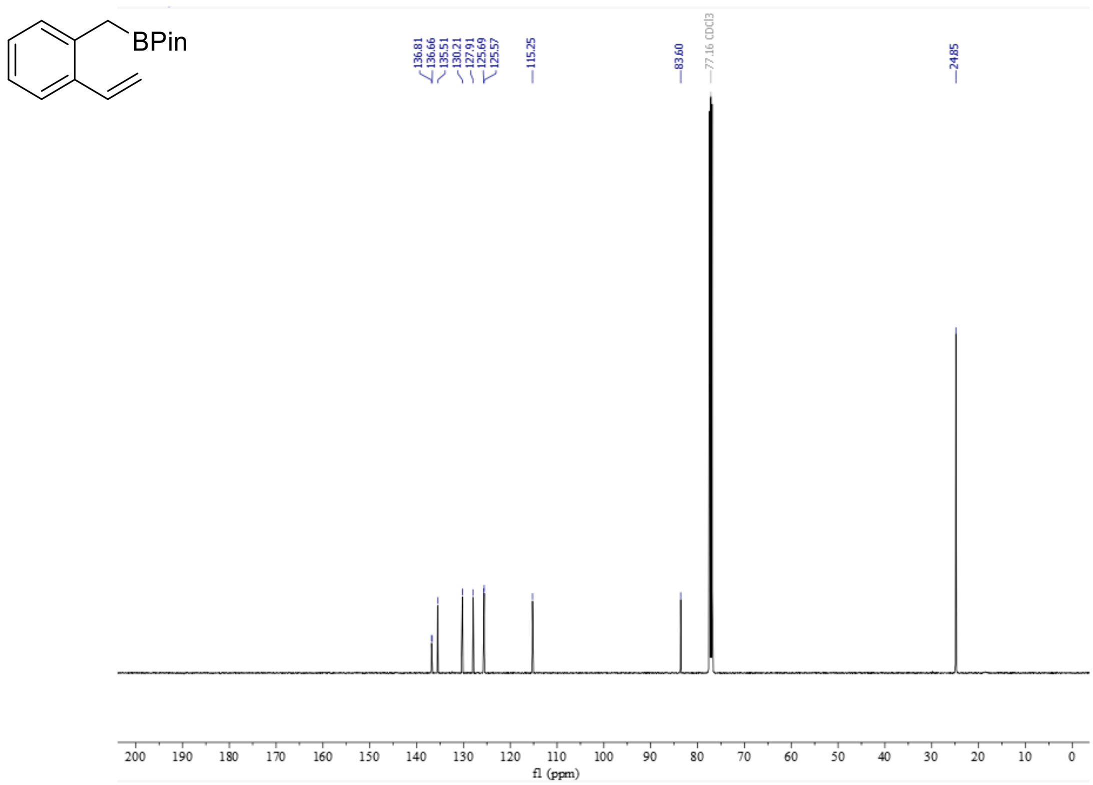

^{1}H-^{13}C-HSQC NMR – *α-boryl carbon atom highlighted*

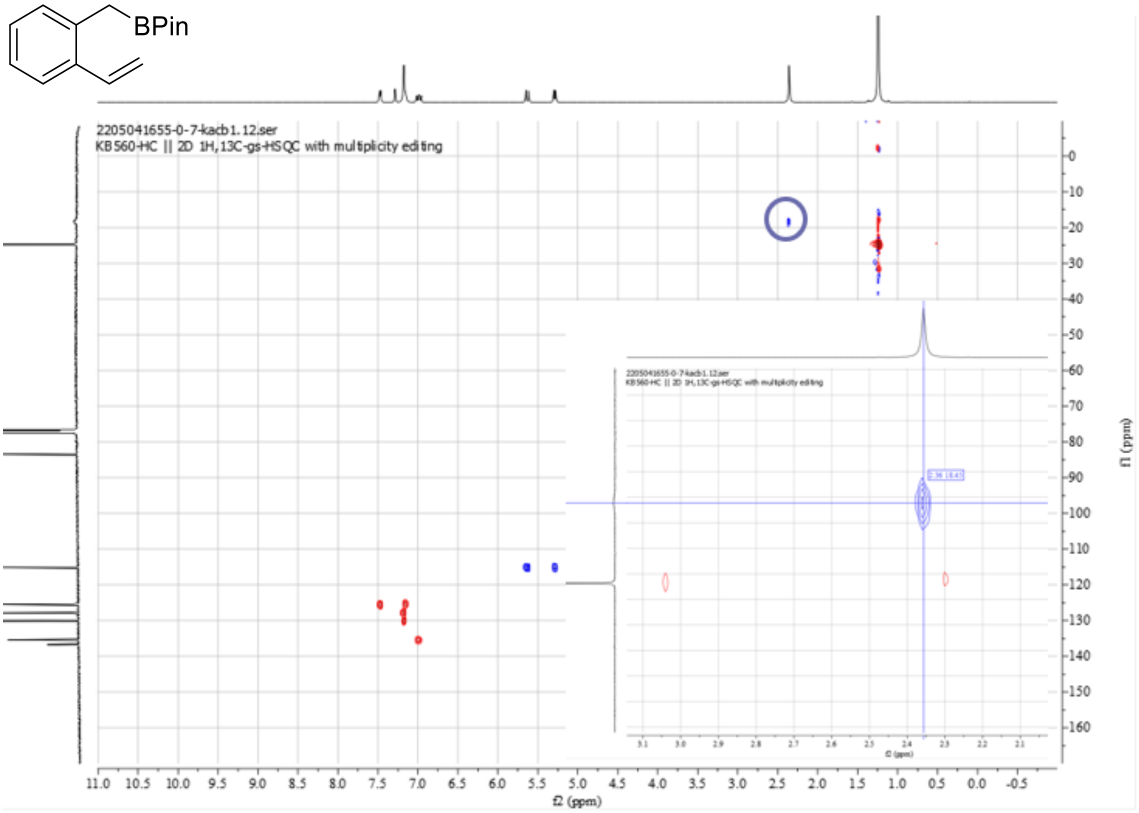

^{11}B NMR (96 MHz, $CDCl_3$)

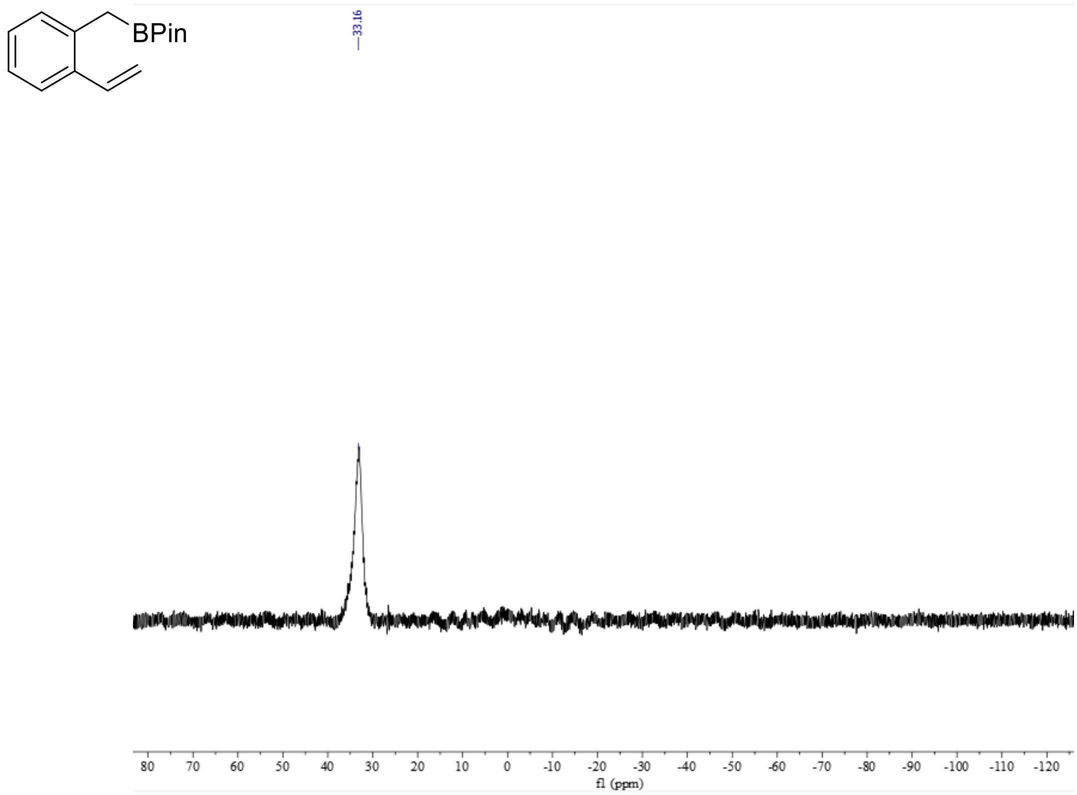

4,4,5,5-tetramethyl-2-(3-vinylbenzyl)-1,3,2-dioxaborolane (16)

^{1}H NMR (500 MHz, $CDCl_3$)

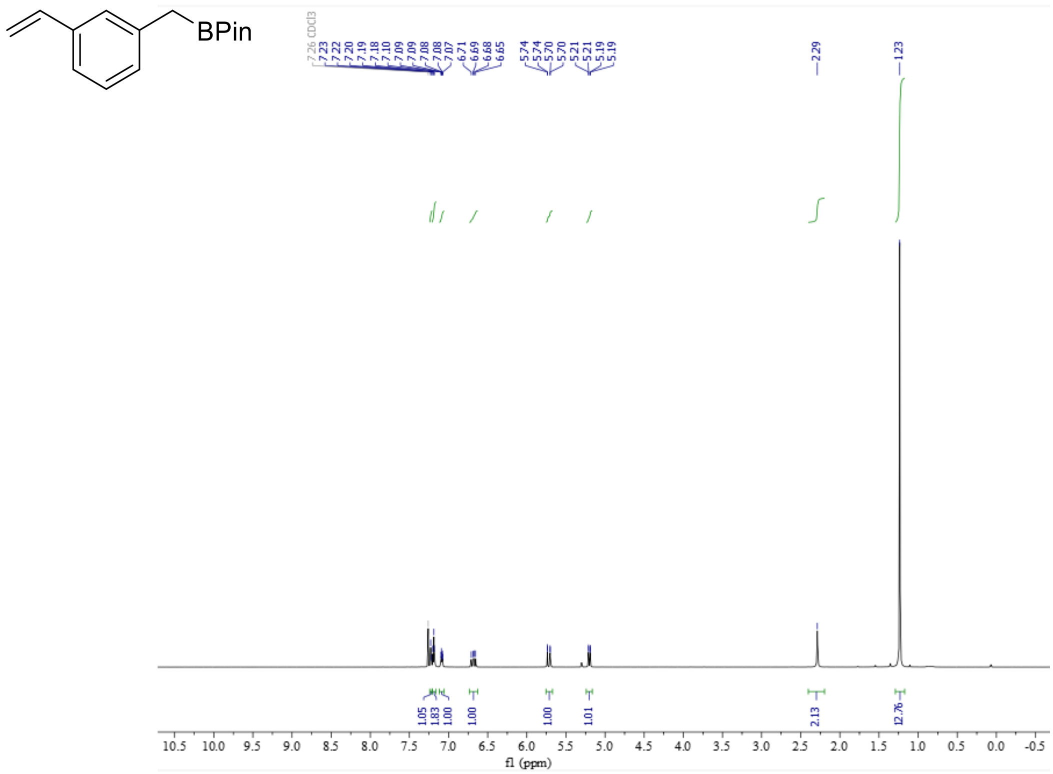

^{13}C NMR (126 MHz, CDCl$_3$)

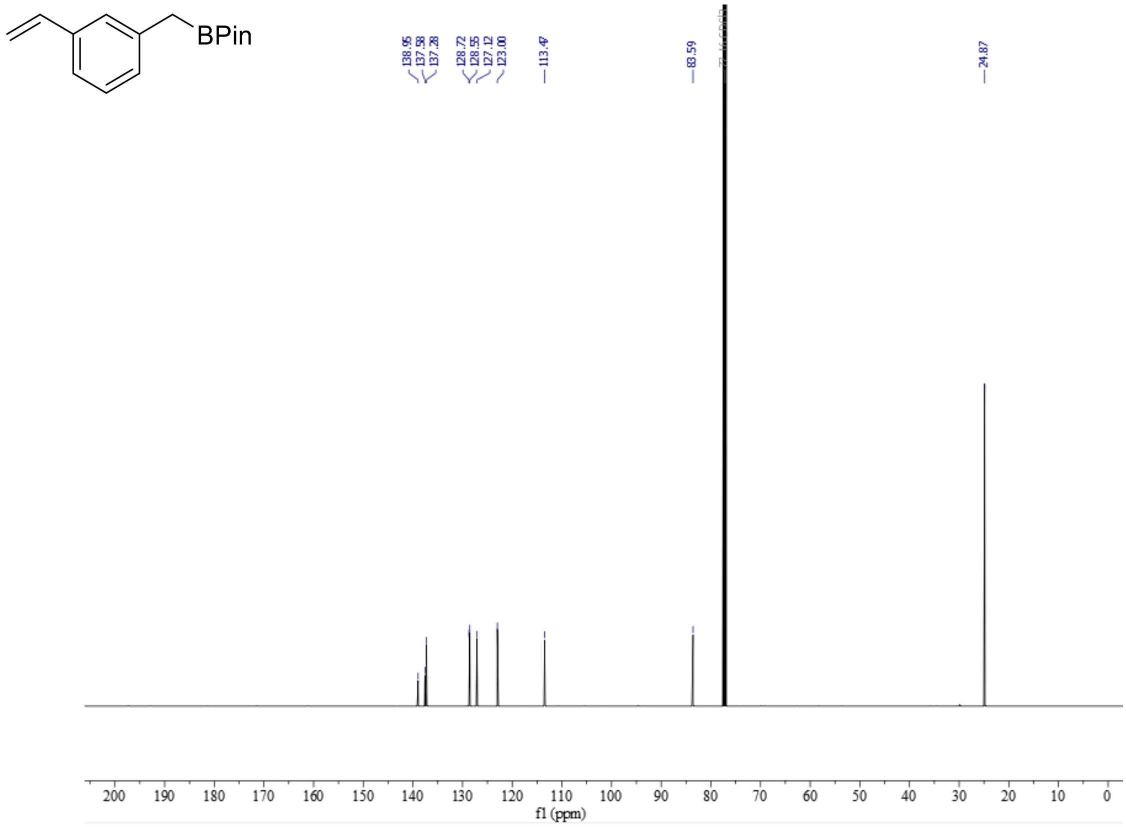

^{1}H-^{13}C-HSQC NMR – *α-boryl carbon atom highlighted*

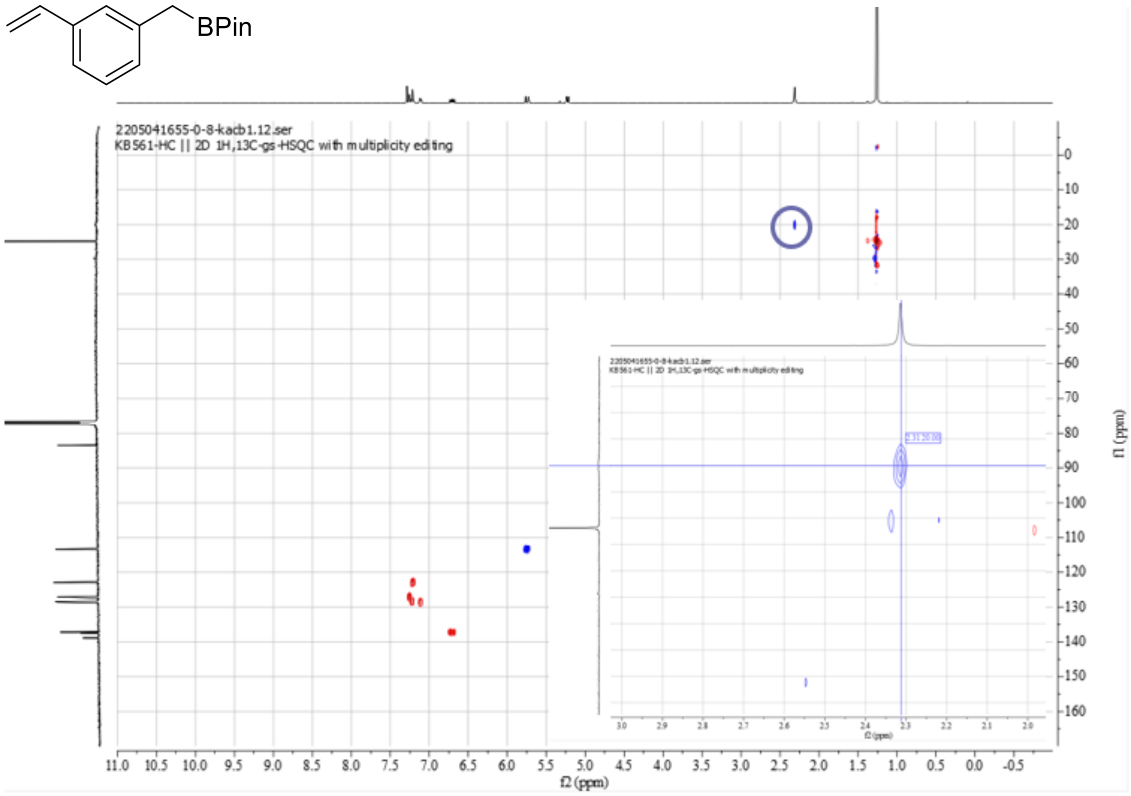

^{11}B NMR (96 MHz, $CDCl_3$)

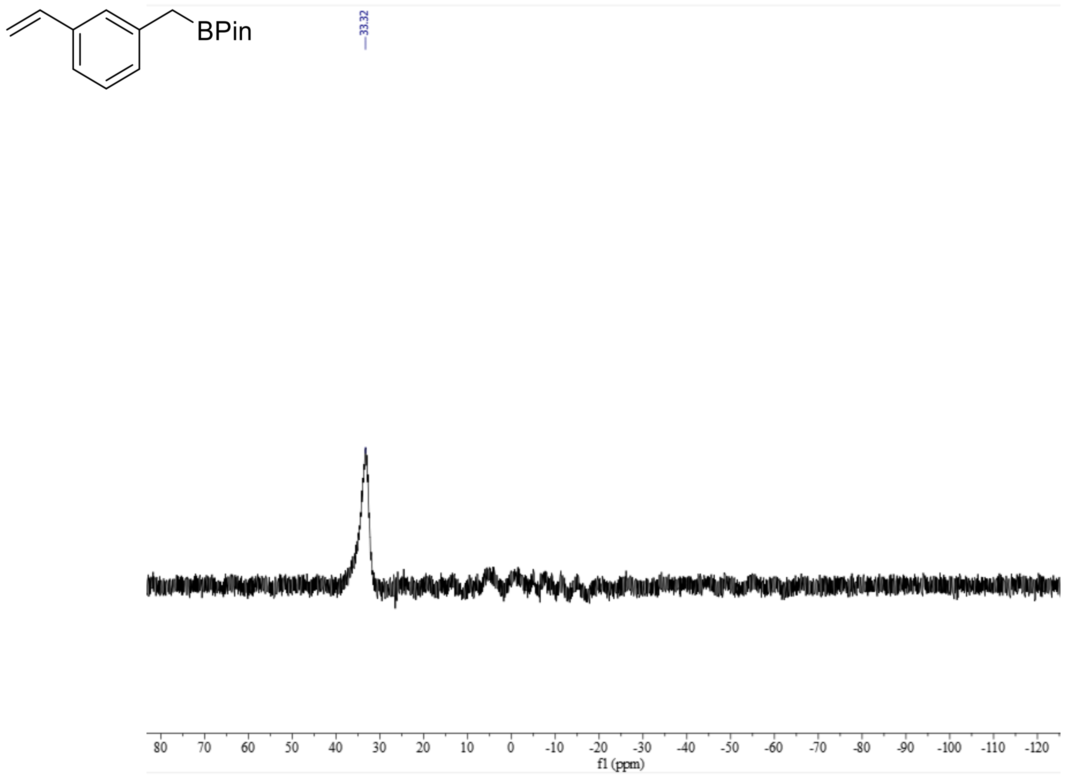

4,4,5,5-tetramethyl-2-(naphthalen-1-ylmethyl)-1,3,2-dioxaborolane (17)

^{1}H NMR (500 MHz, $CDCl_3$)

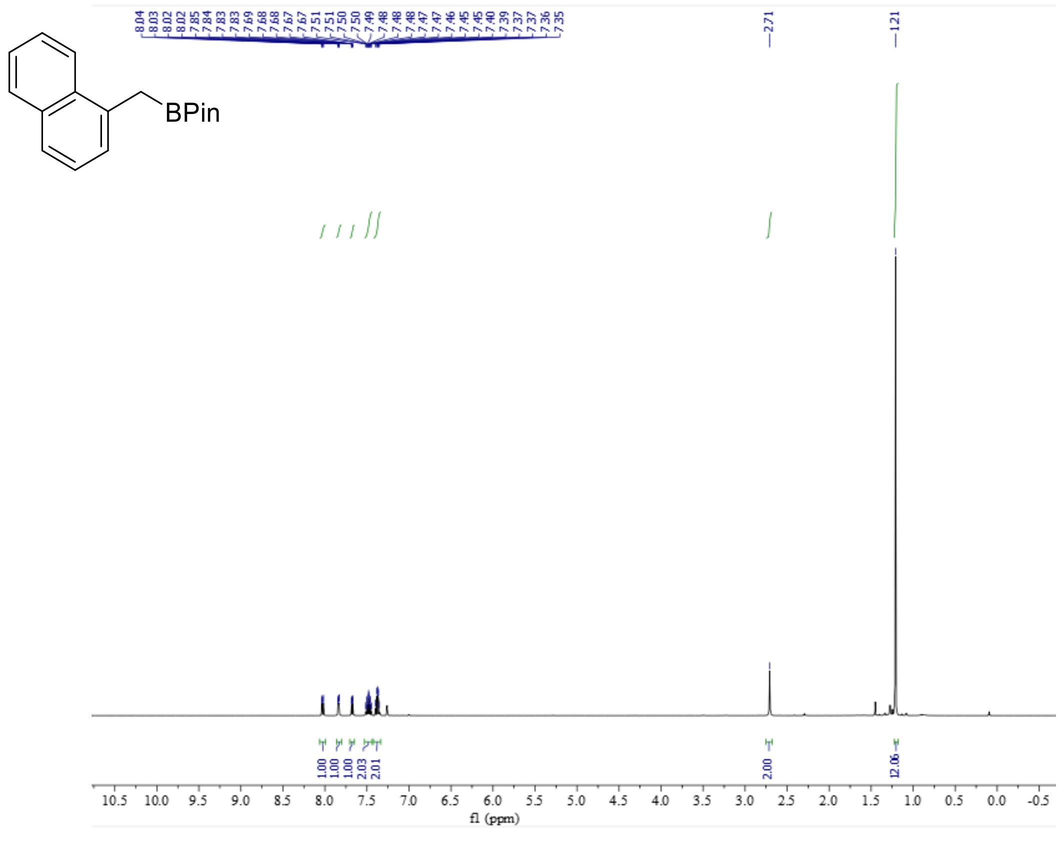

^{13}C NMR (126 MHz, $CDCl_3$)

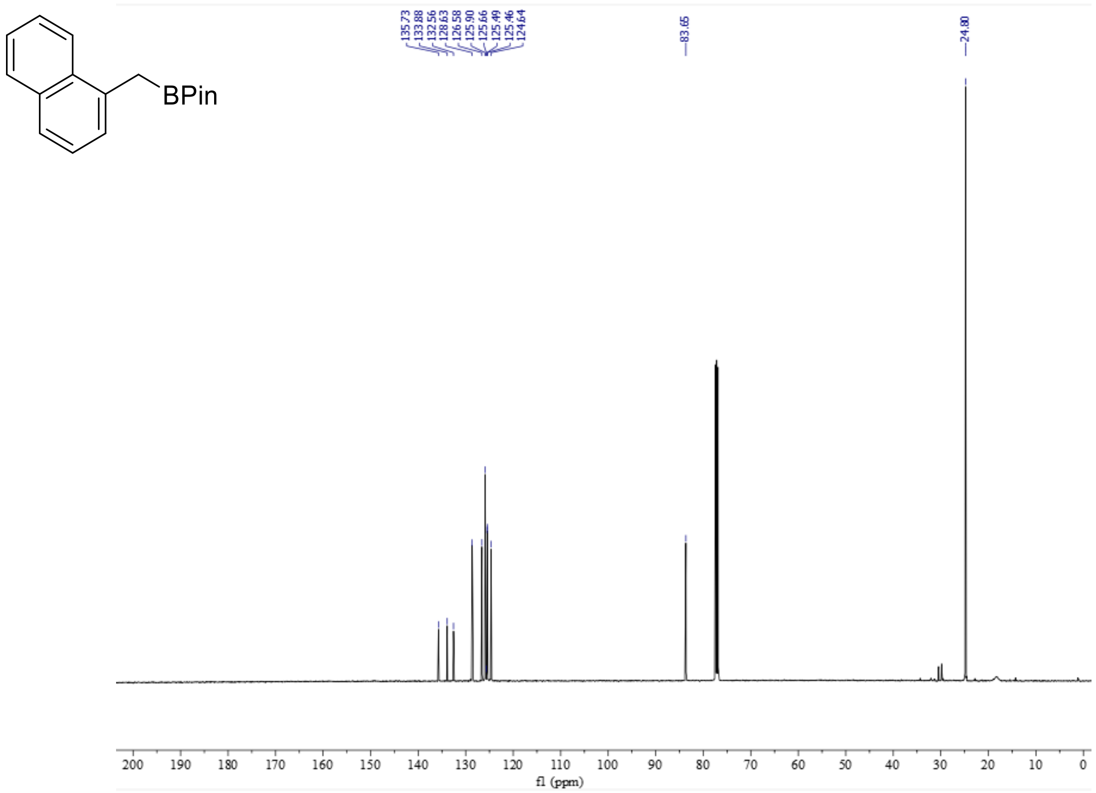

^{11}B NMR (96 MHz, $CDCl_3$)

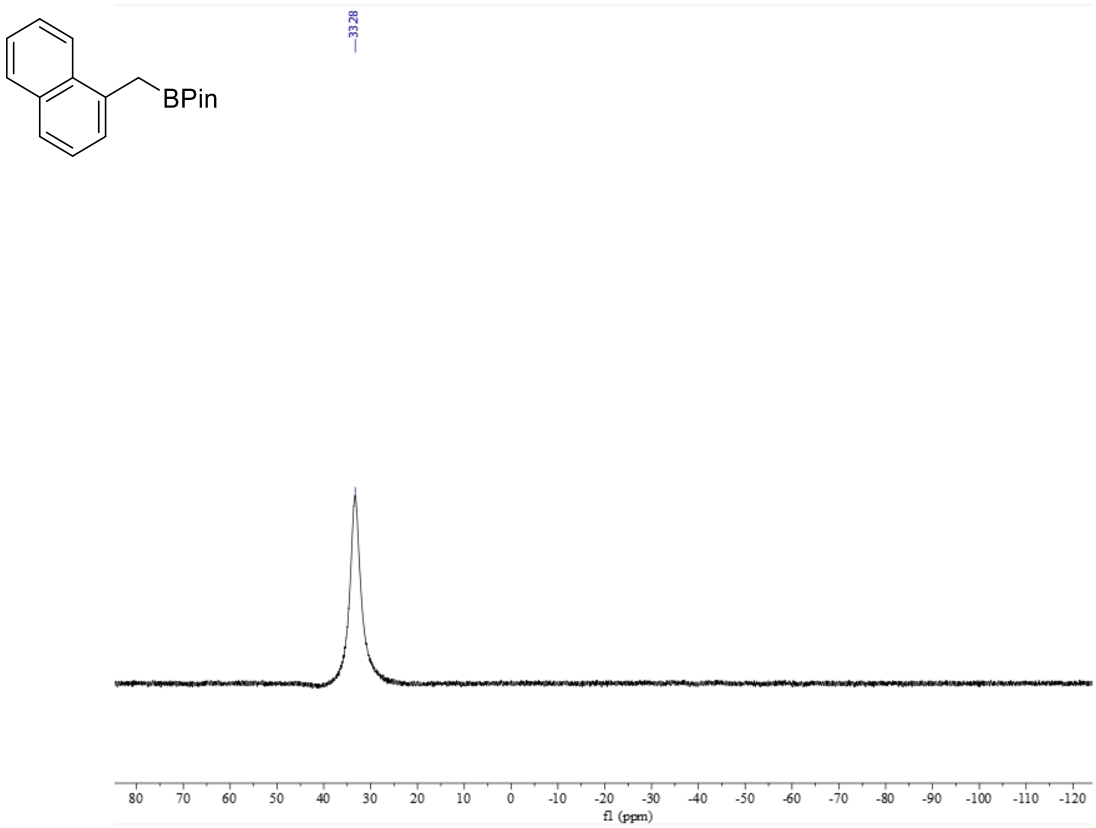

2-(4-methoxybenzyl)-4,4,5,5-tetramethyl-1,3,2-dioxaborolane (18)

^{1}H NMR (500 MHz, CDCl₃)

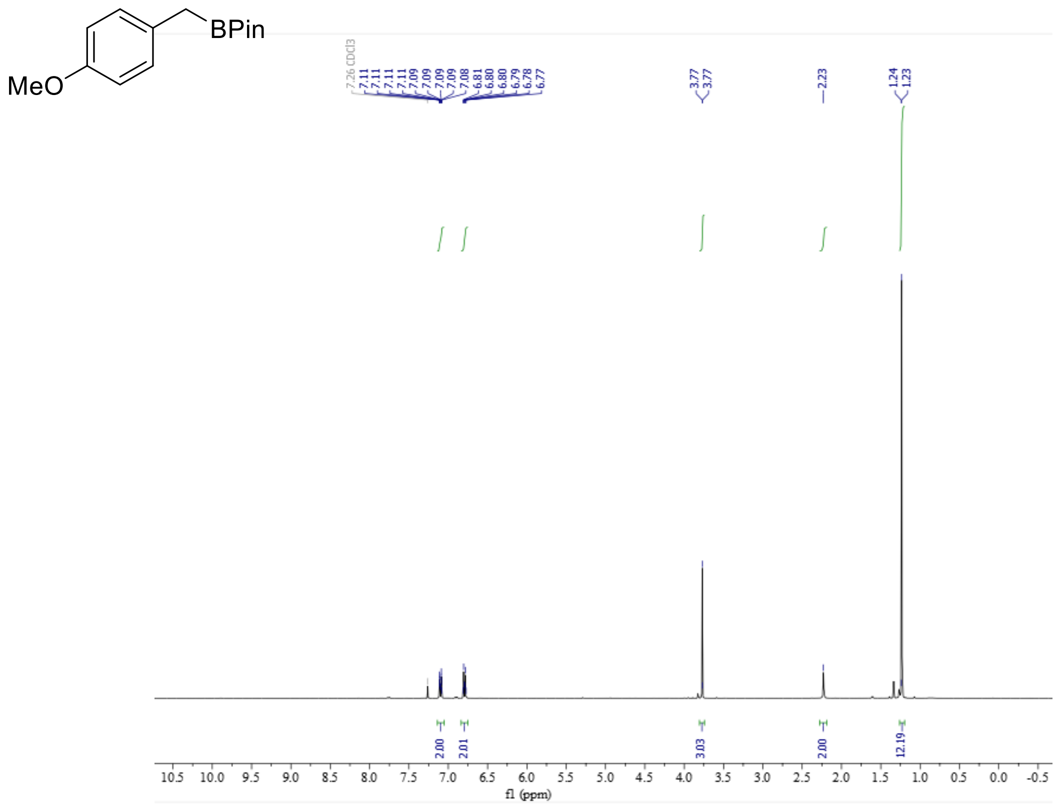

^{13}C NMR (126 MHz, CDCl₃)

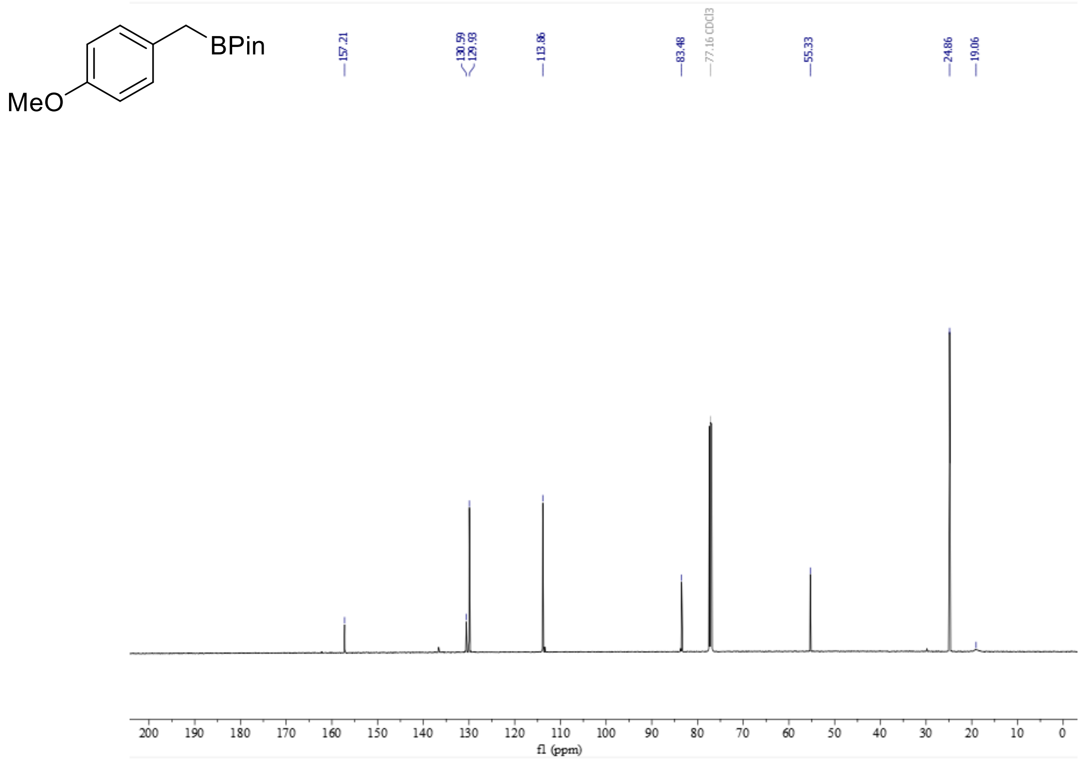

^{1}H-^{13}C-HSQC NMR – *α-boryl carbon atom highlighted*

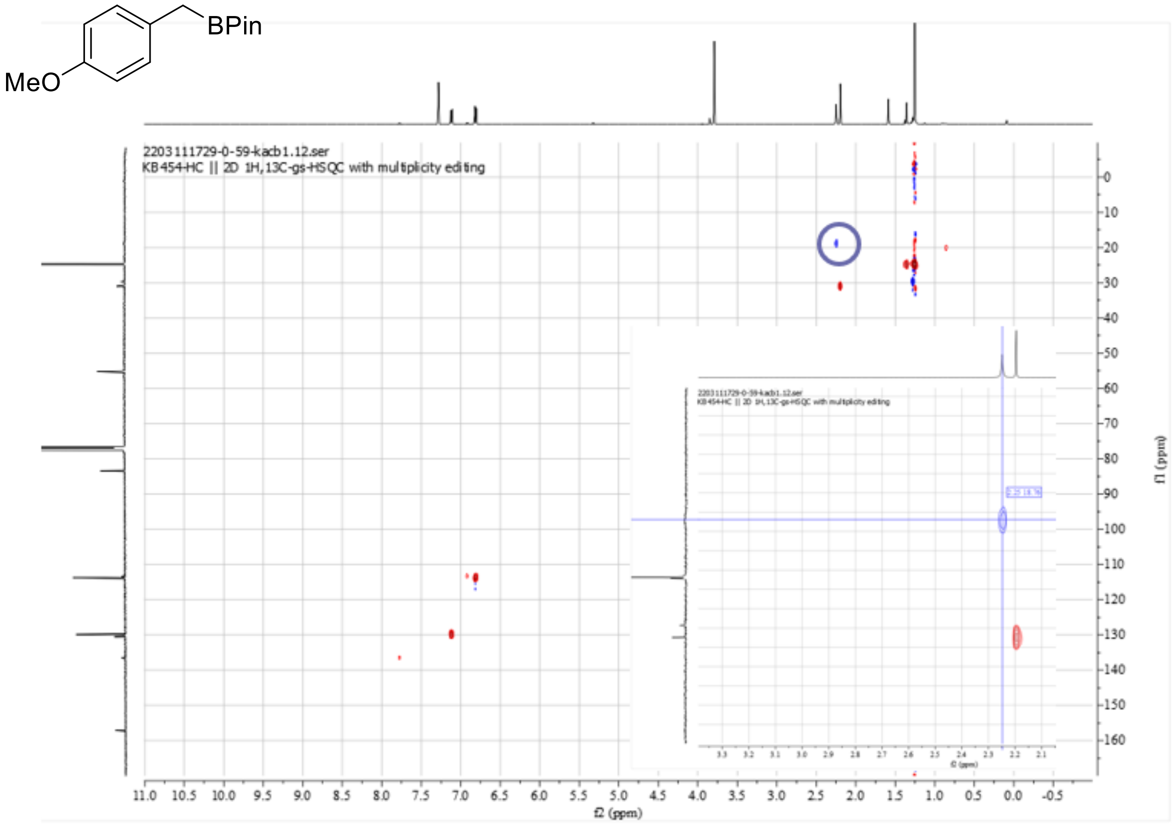

^{11}B NMR (96 MHz, $CDCl_3$)

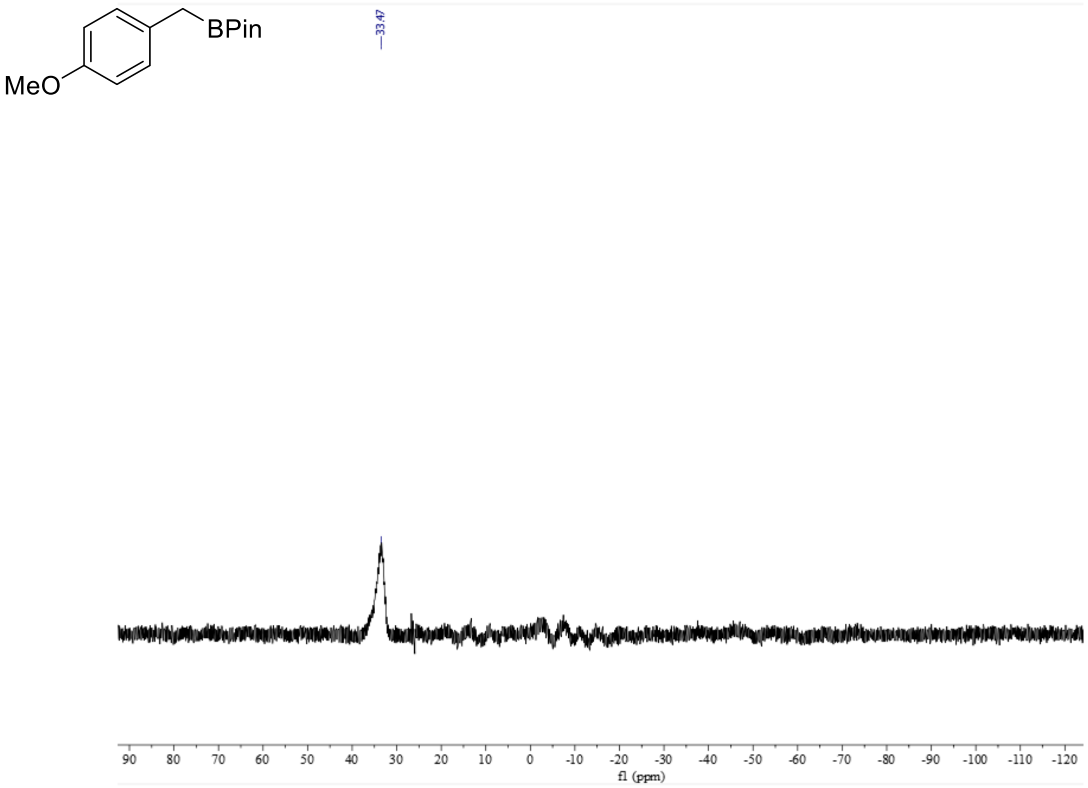

2-(3-methoxybenzyl)-4,4,5,5-tetramethyl-1,3,2-dioxaborolane (19)

^{1}H NMR (500 MHz, $CDCl_3$)

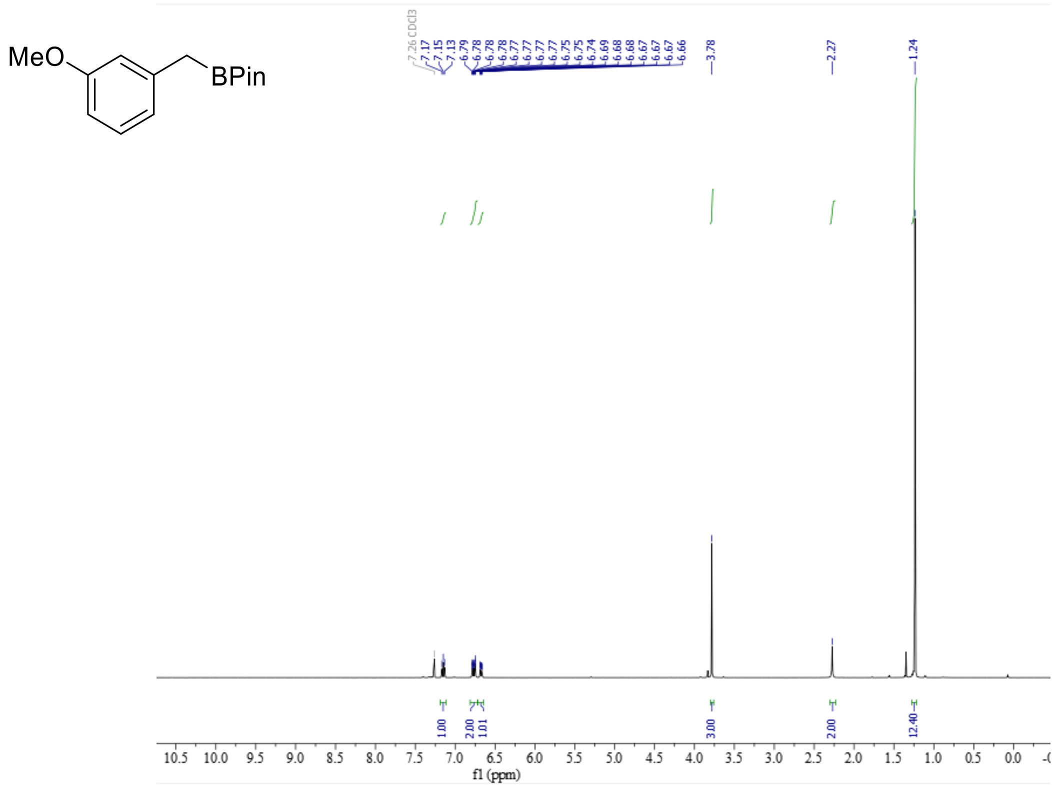

^{13}C NMR (126 MHz, $CDCl_3$)

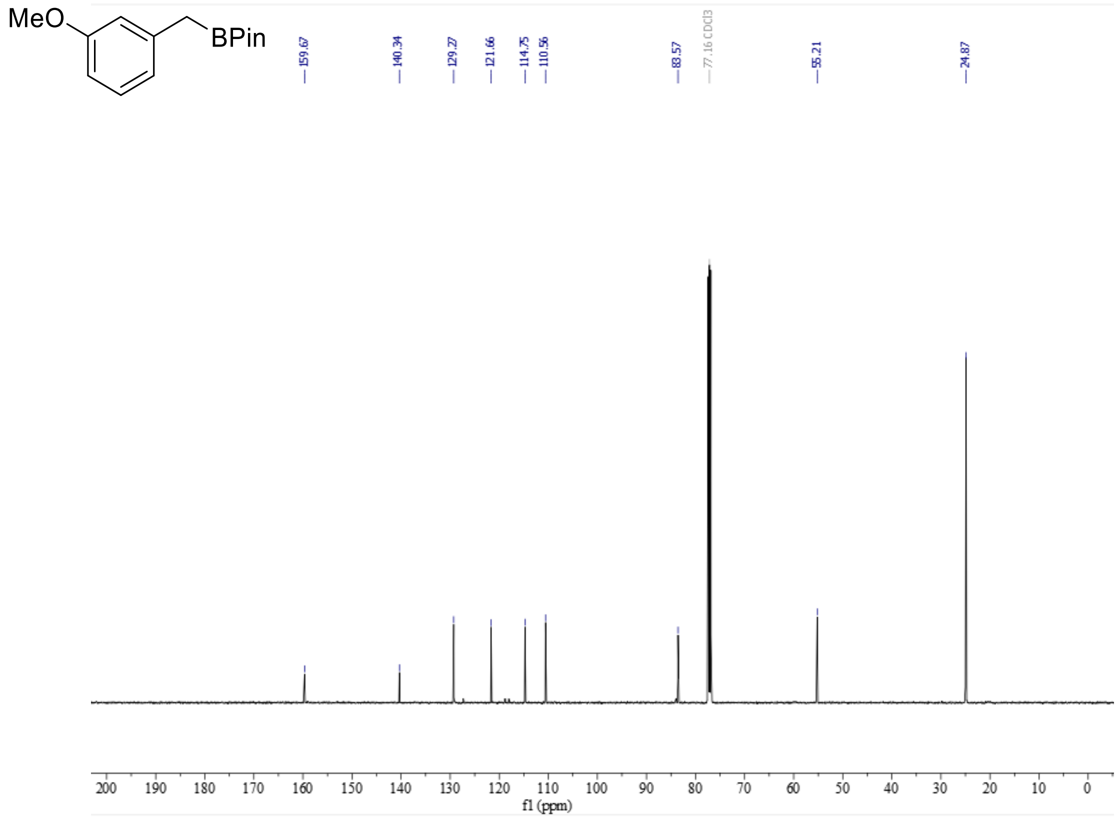

^{1}H-^{13}C-HSQC NMR – *α-boryl carbon atom highlighted*

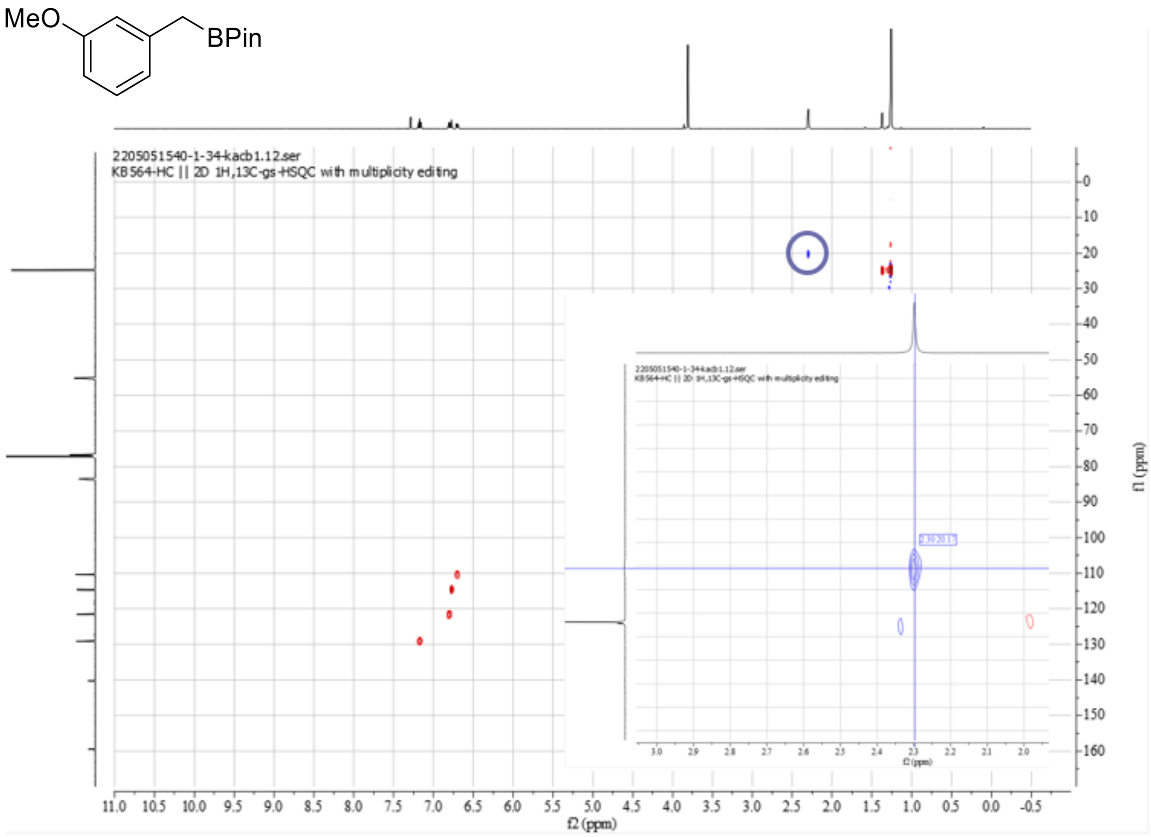

^{11}B NMR (96 MHz, $CDCl_3$)

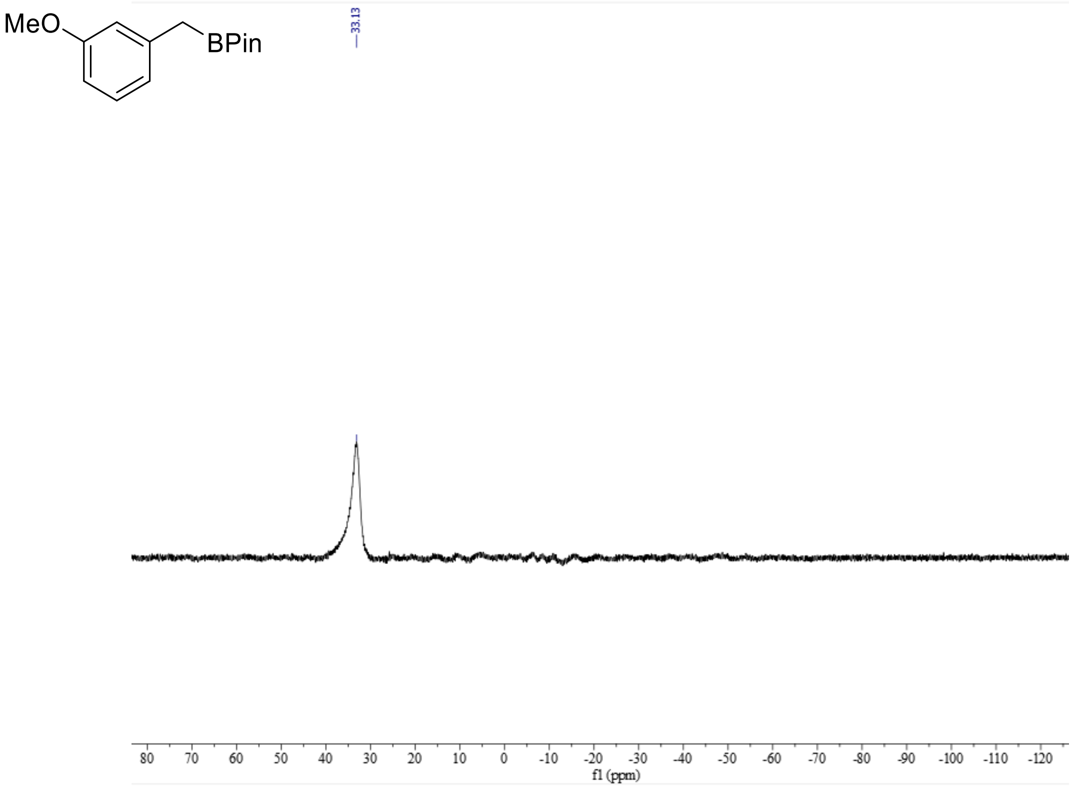

2-(2-methoxybenzyl)-4,4,5,5-tetramethyl-1,3,2-dioxaborolane (20)

^{1}H NMR (500 MHz, $CDCl_3$)

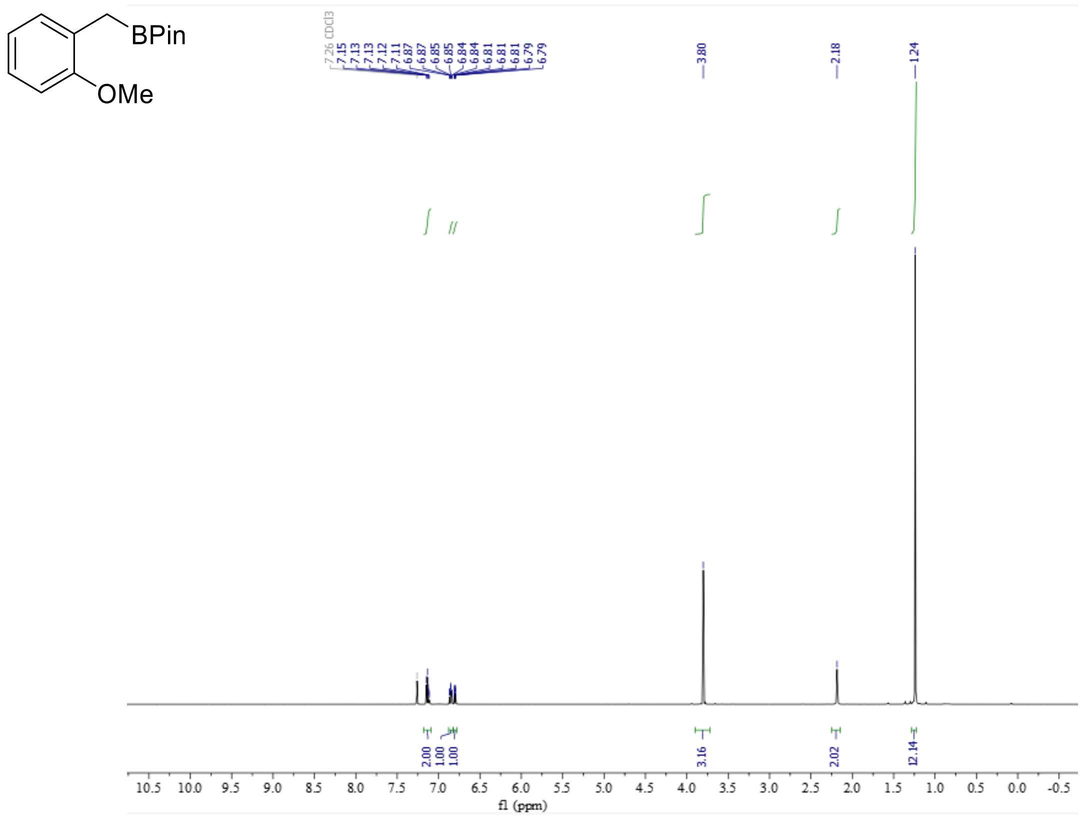

^{13}C NMR (126 MHz, $CDCl_3$)

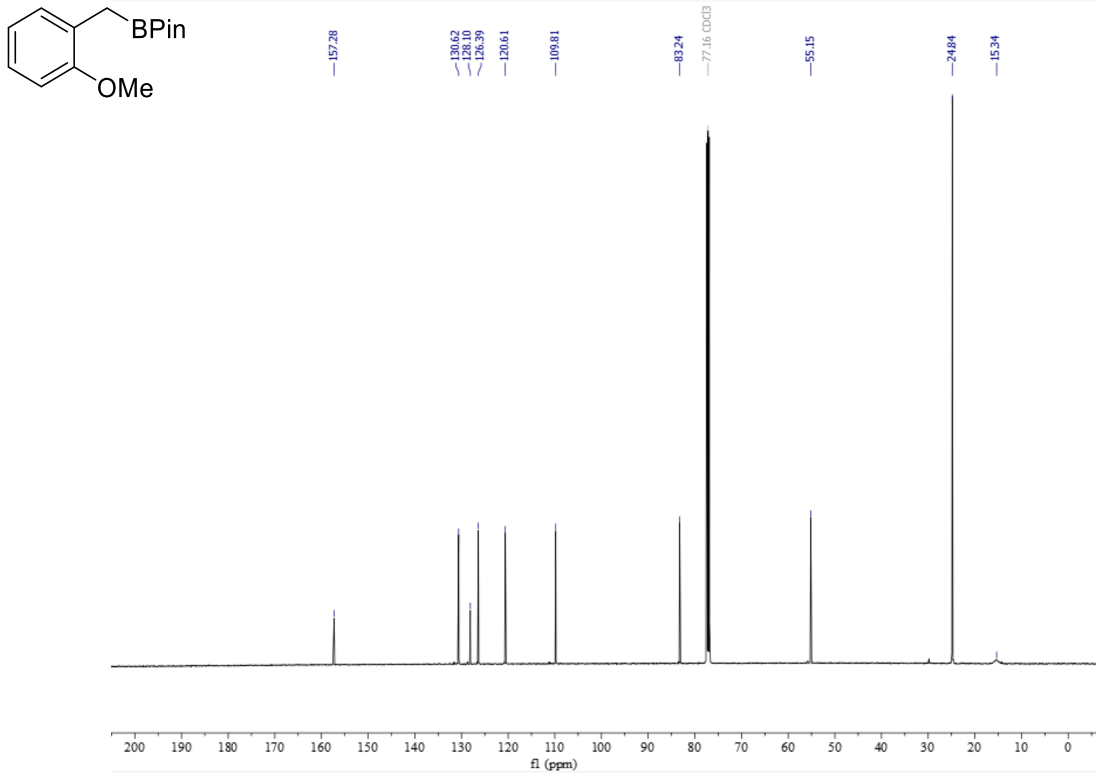

^{1}H-^{13}C-HSQC NMR – *α-boryl carbon atom highlighted*

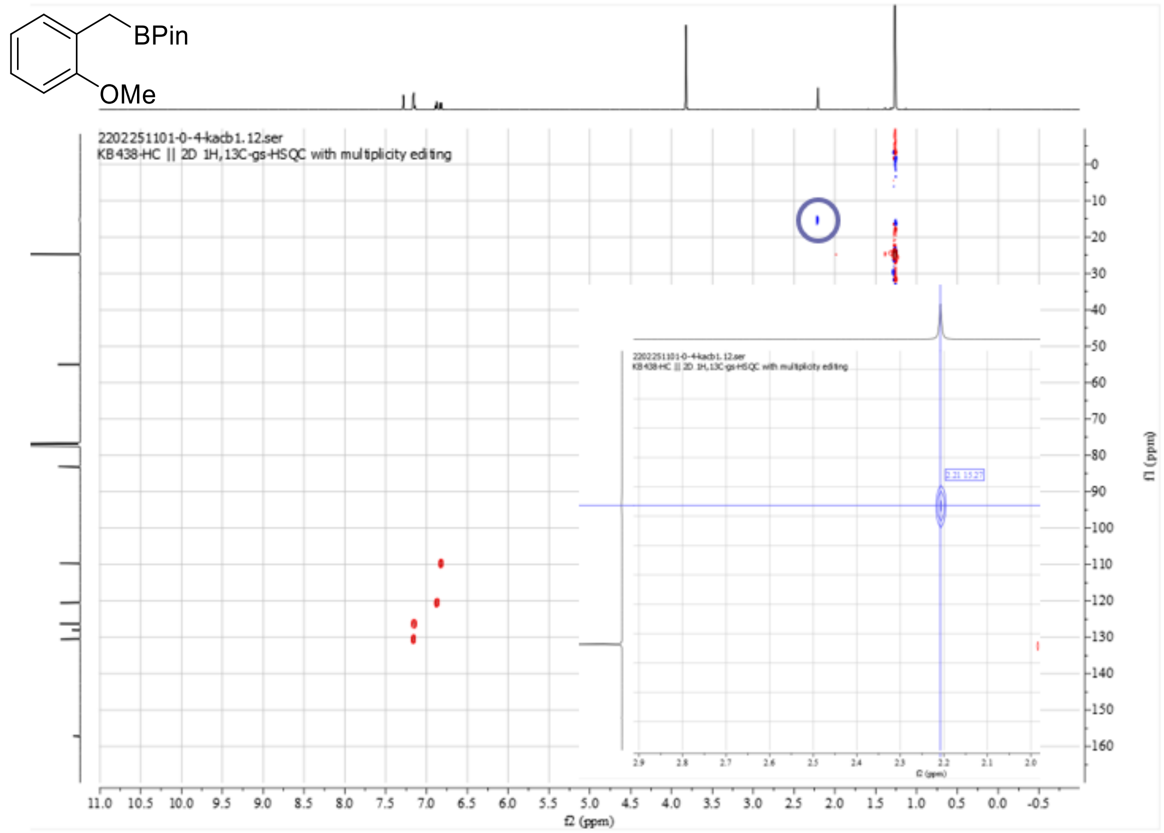

^{11}B NMR (96 MHz, $CDCl_3$)

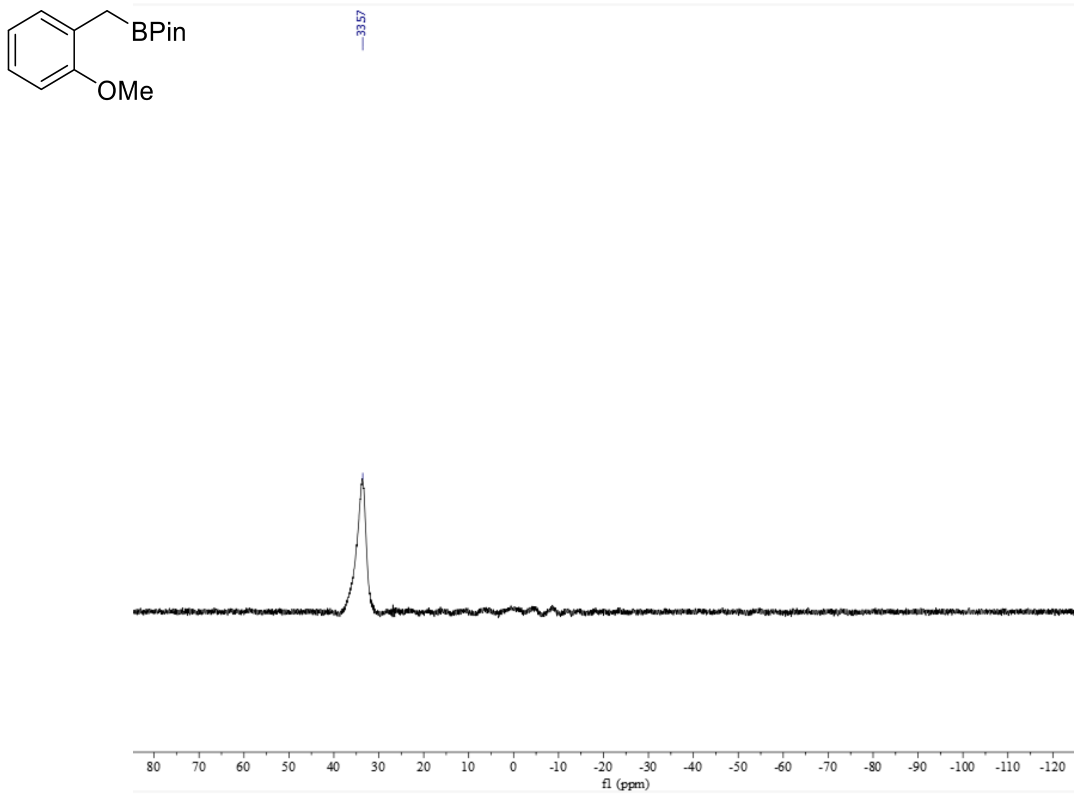

2-(2,4-dimethoxybenzyl)-4,4,5,5-tetramethyl-1,3,2-dioxaborolane (21)

^{1}H NMR (500 MHz, $CDCl_3$)

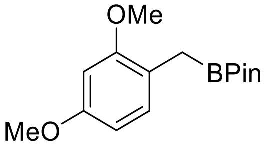

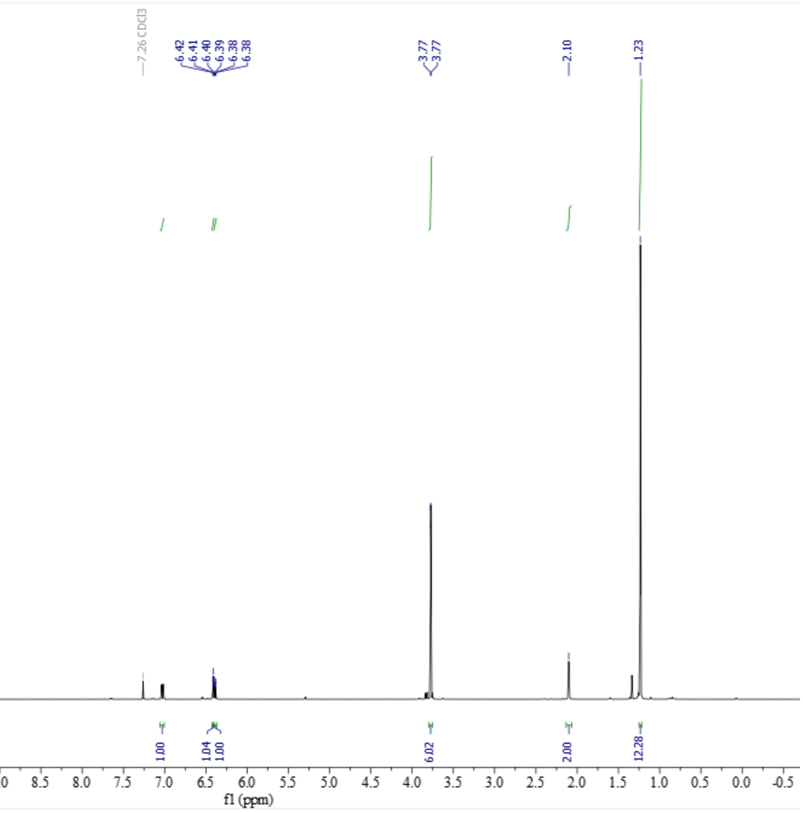

^{13}C NMR (126 MHz, $CDCl_3$)

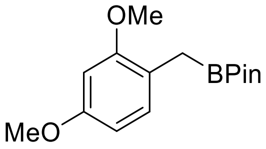

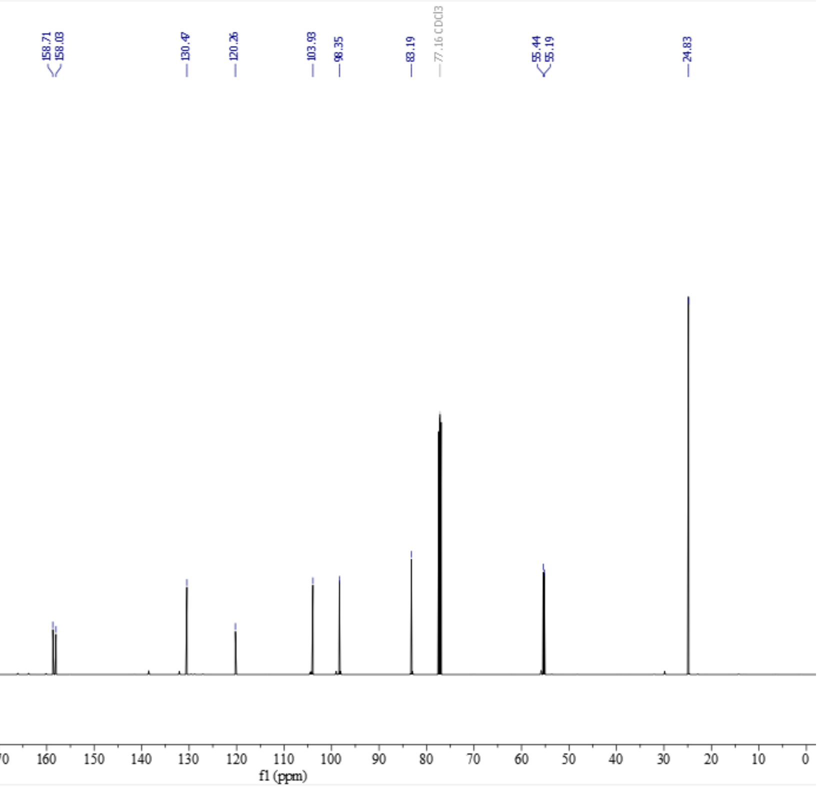

^{1}H-^{13}C-HSQC NMR – *α-boryl carbon atom highlighted*

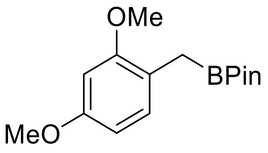

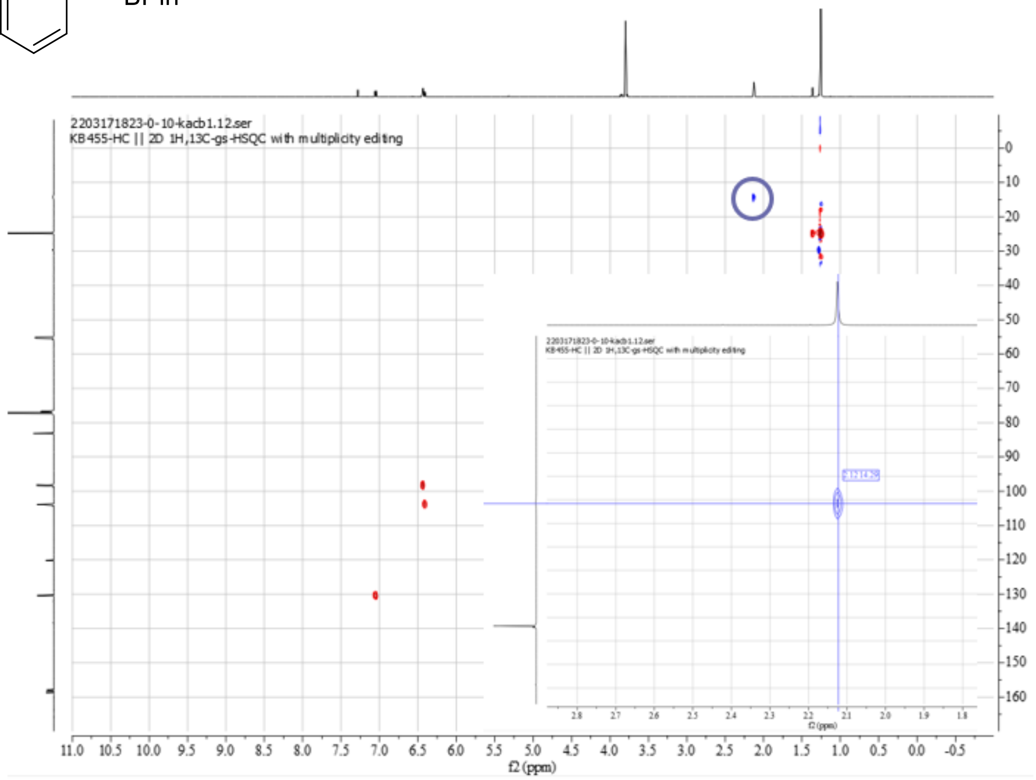

^{11}B NMR (96 MHz, CDCl$_3$)

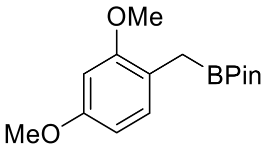

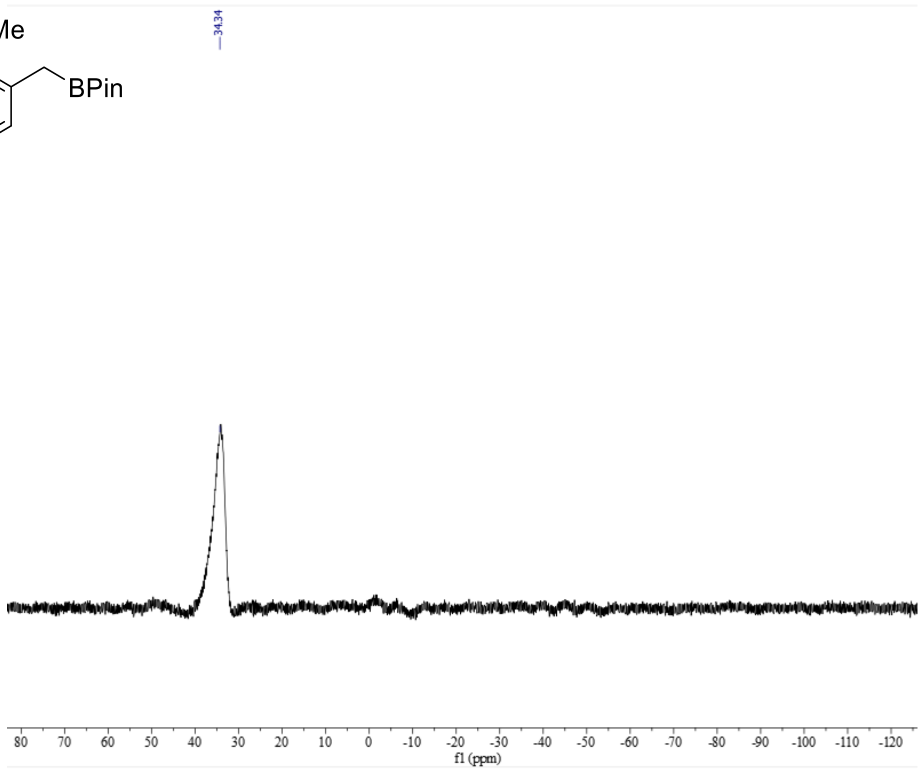

4,4,5,5-tetramethyl-2-(3,4,5-trimethoxybenzyl)-1,3,2-dioxaborolane (22)

^{1}H NMR (500 MHz, $CDCl_3$)

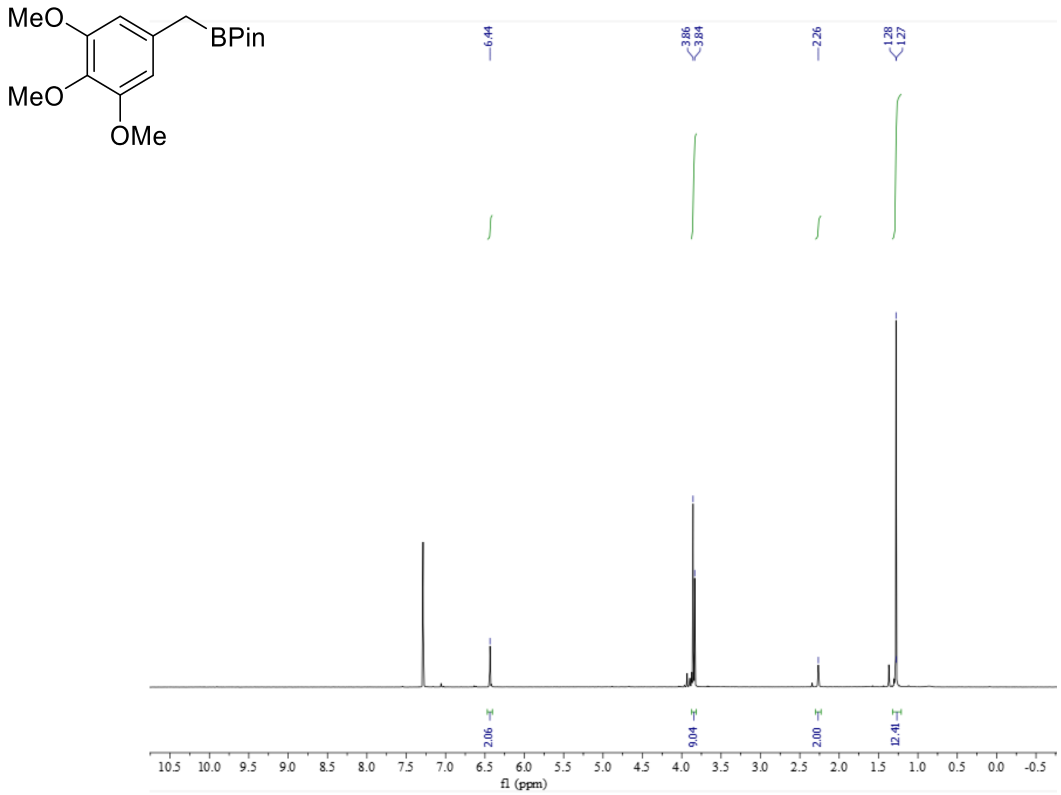

^{13}C NMR (126 MHz, $CDCl_3$)

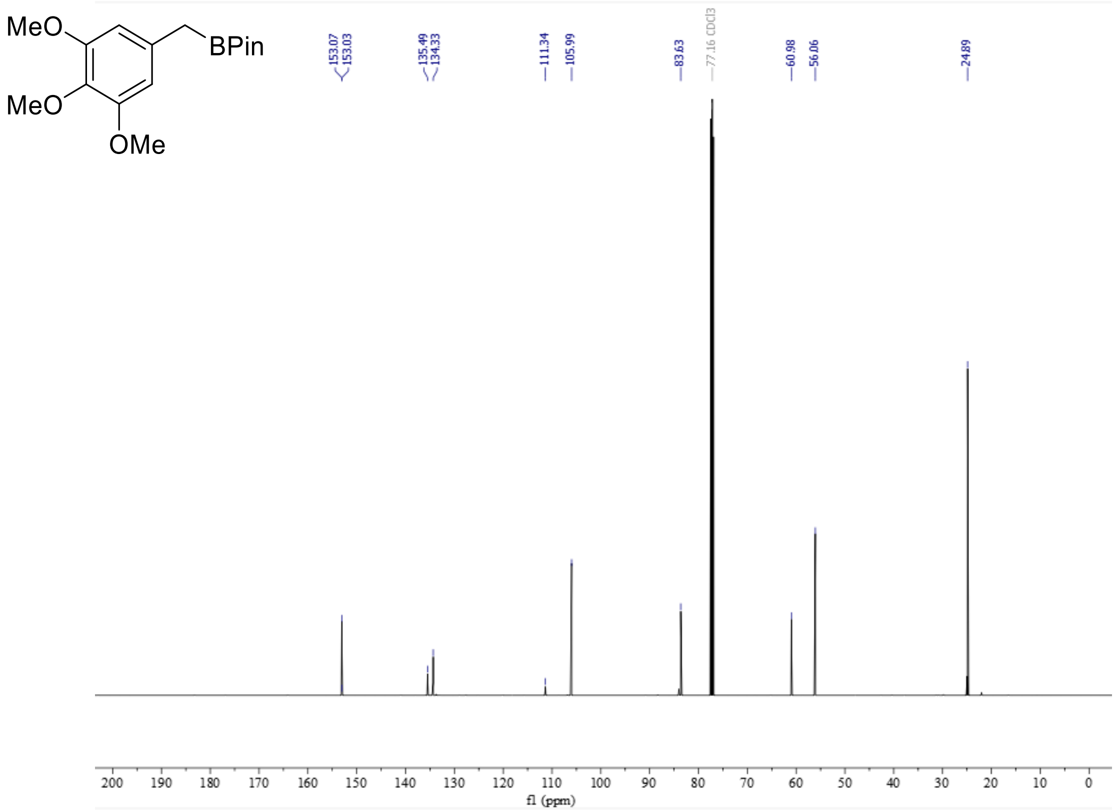

H-^{13}C-HSQC NMR – *α-boryl carbon atom highlighted*

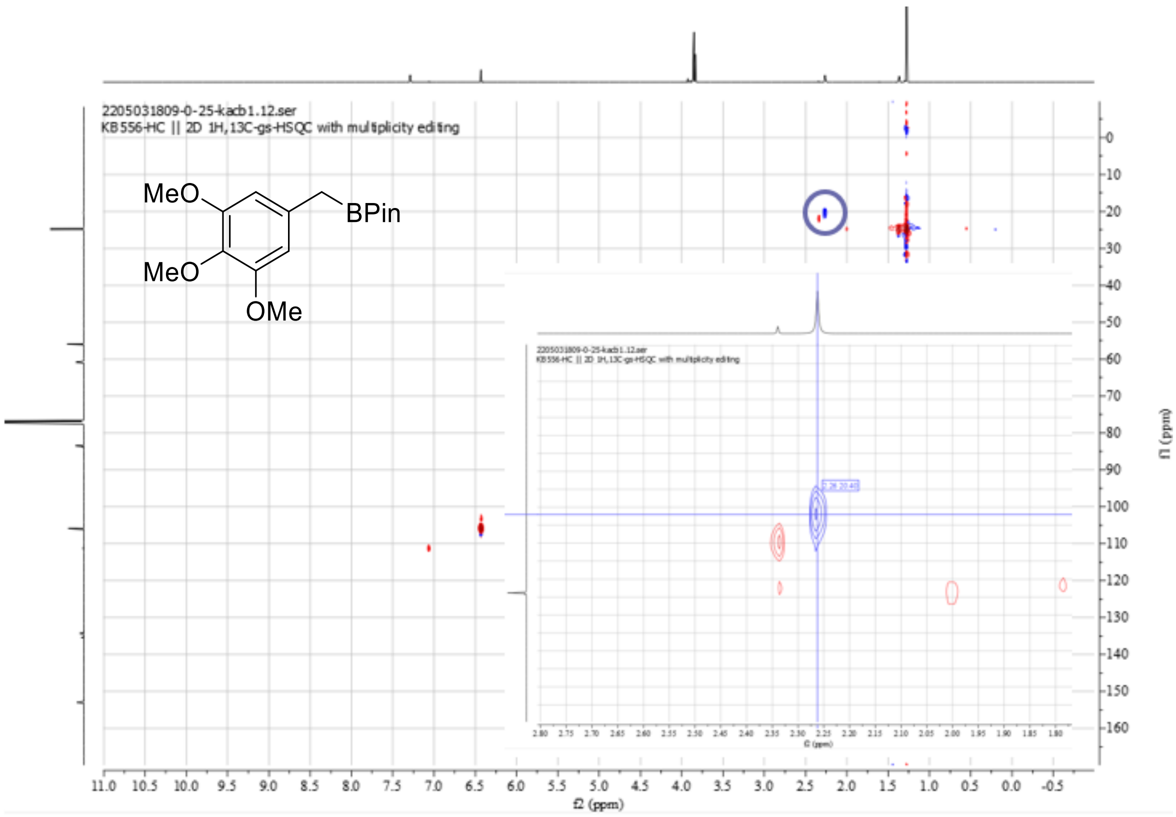

^{11}B NMR (96 MHz, $CDCl_3$)

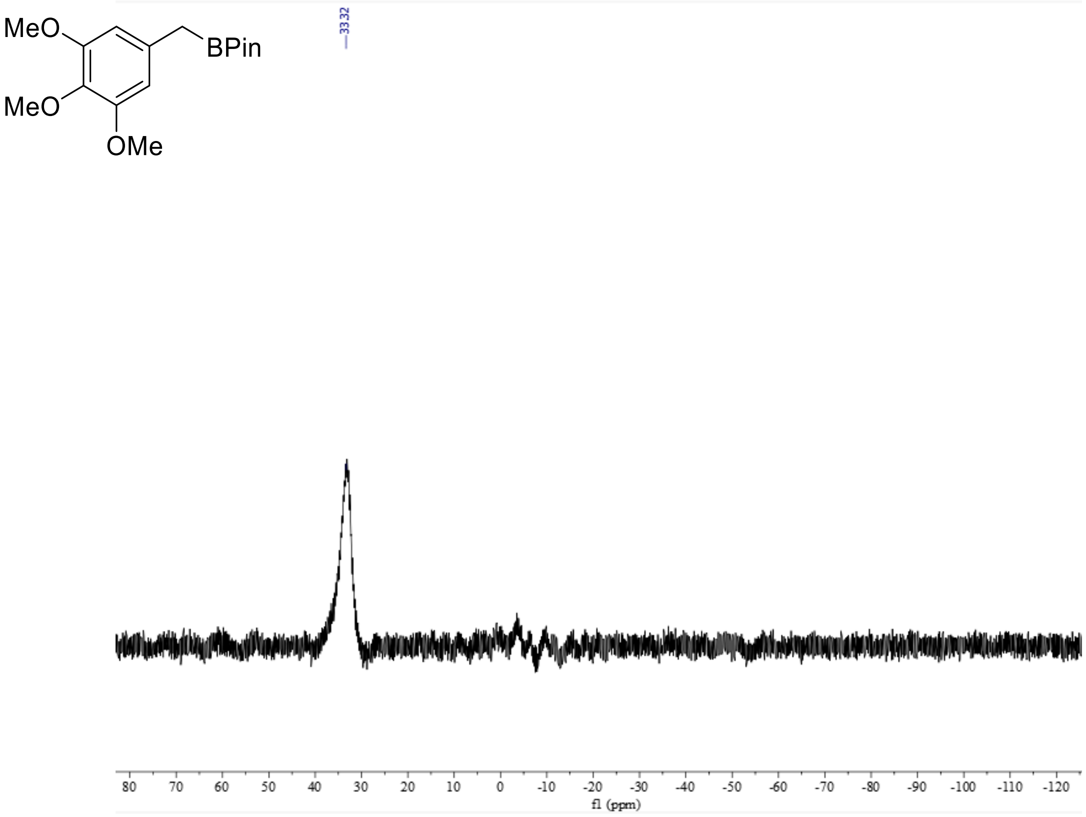

4,4,5,5-tetramethyl-2-(4-(methylthio)benzyl)-1,3,2-dioxaborolane (23)

^{1}H NMR (500 MHz, CDCl$_3$)

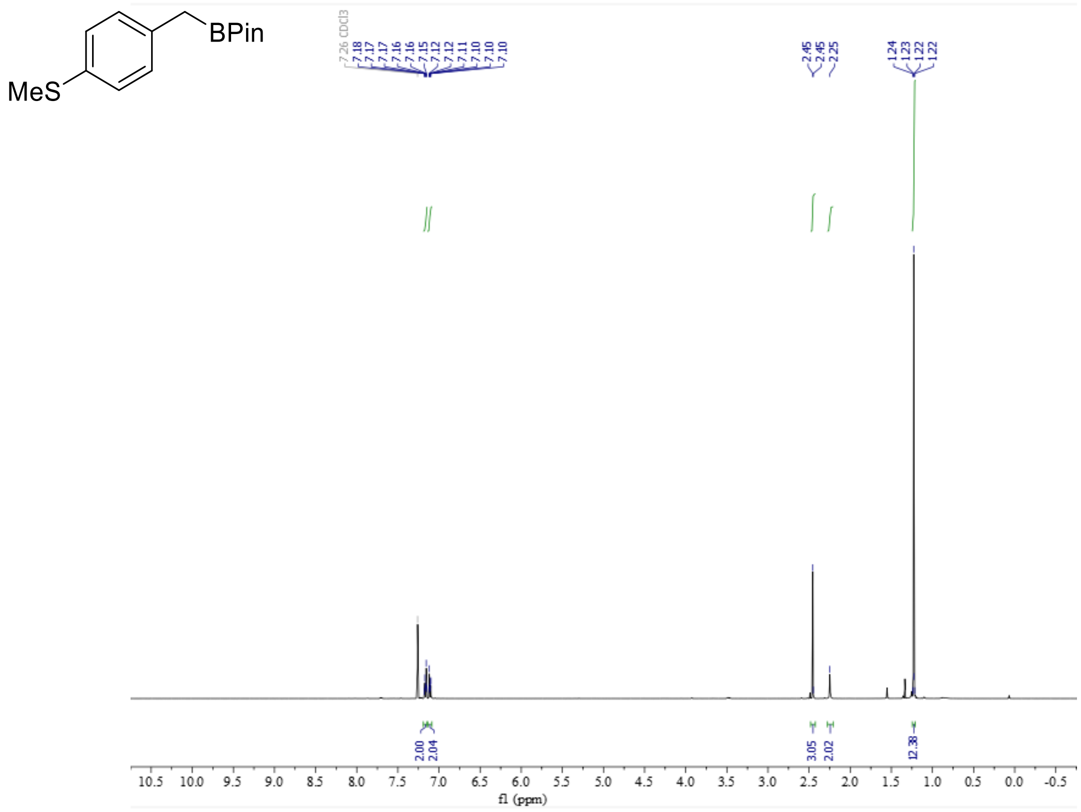

^{13}C NMR (500 MHz, CDCl$_3$)

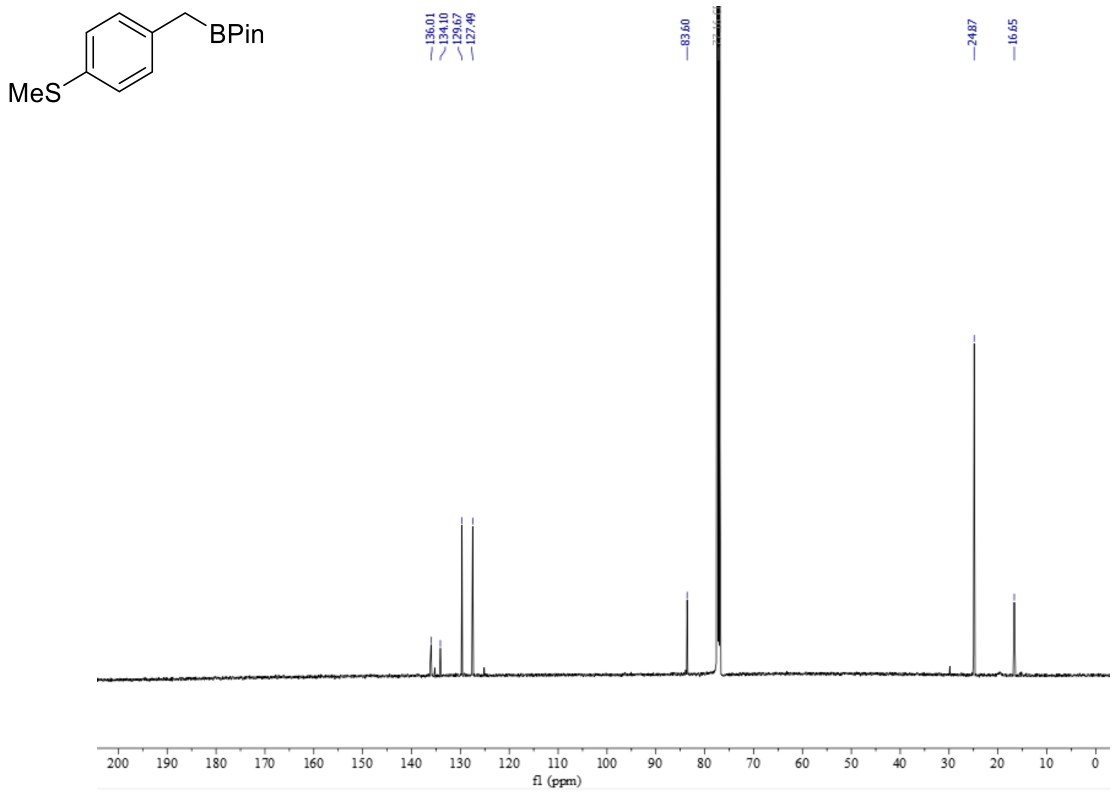

^{1}H-^{13}C-HSQC NMR – *α-boryl carbon atom highlighted*

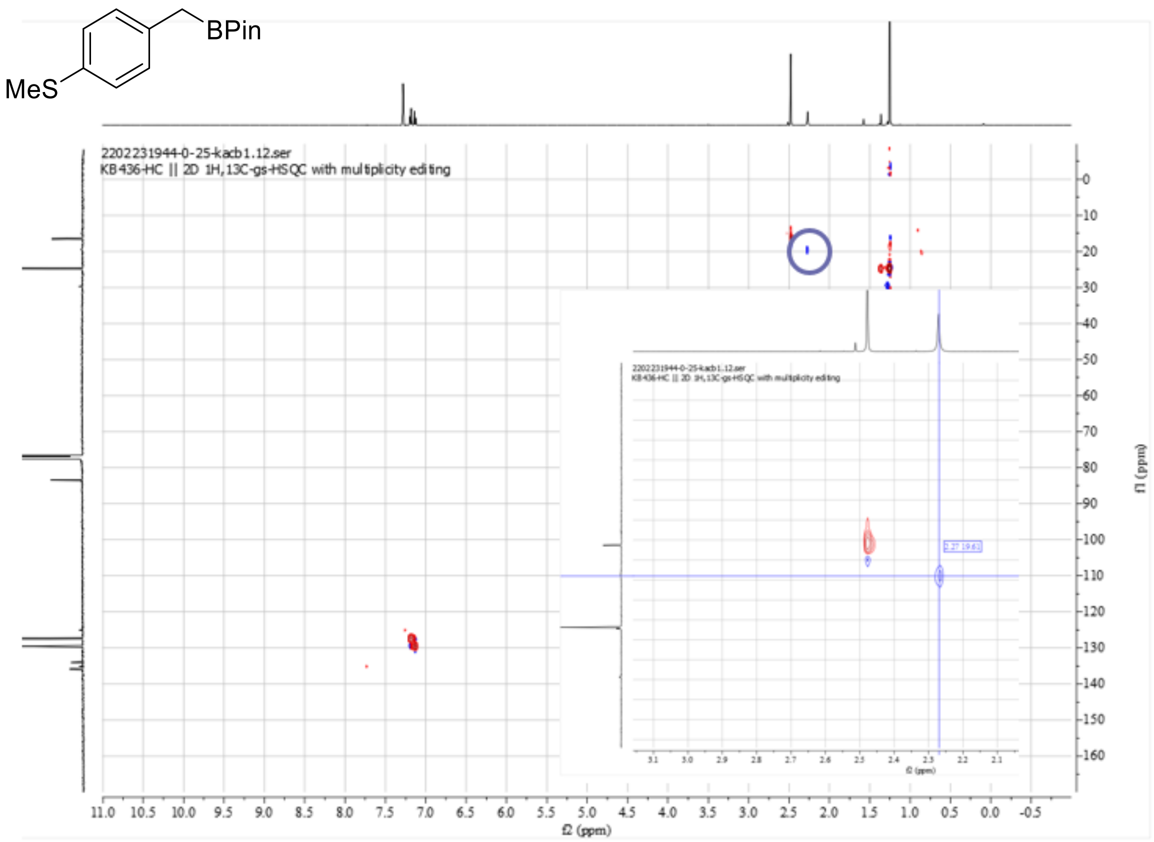

^{11}B NMR (96 MHz, $CDCl_3$)

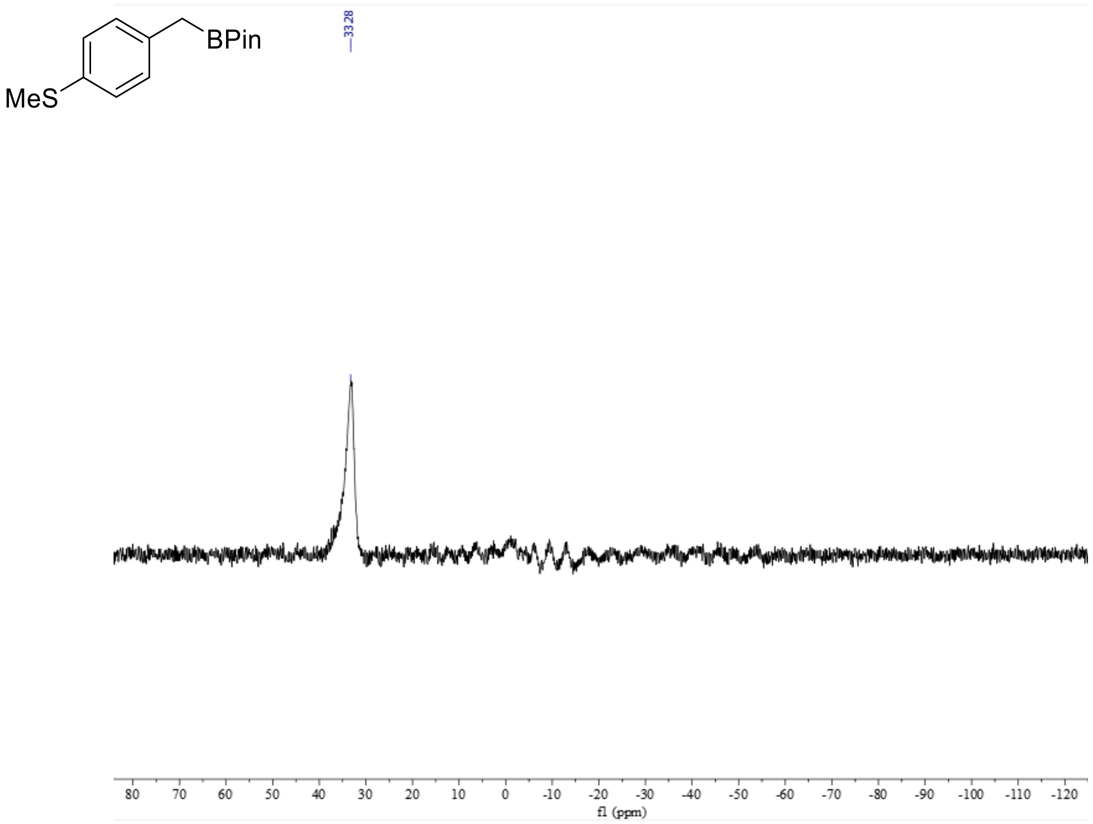

trimethyl(4-((4,4,5,5-tetramethyl-1,3,2-dioxaborolan-2-yl)methyl)phenyl)silane (24)

^{1}H NMR (500 MHz, CDCl$_3$)

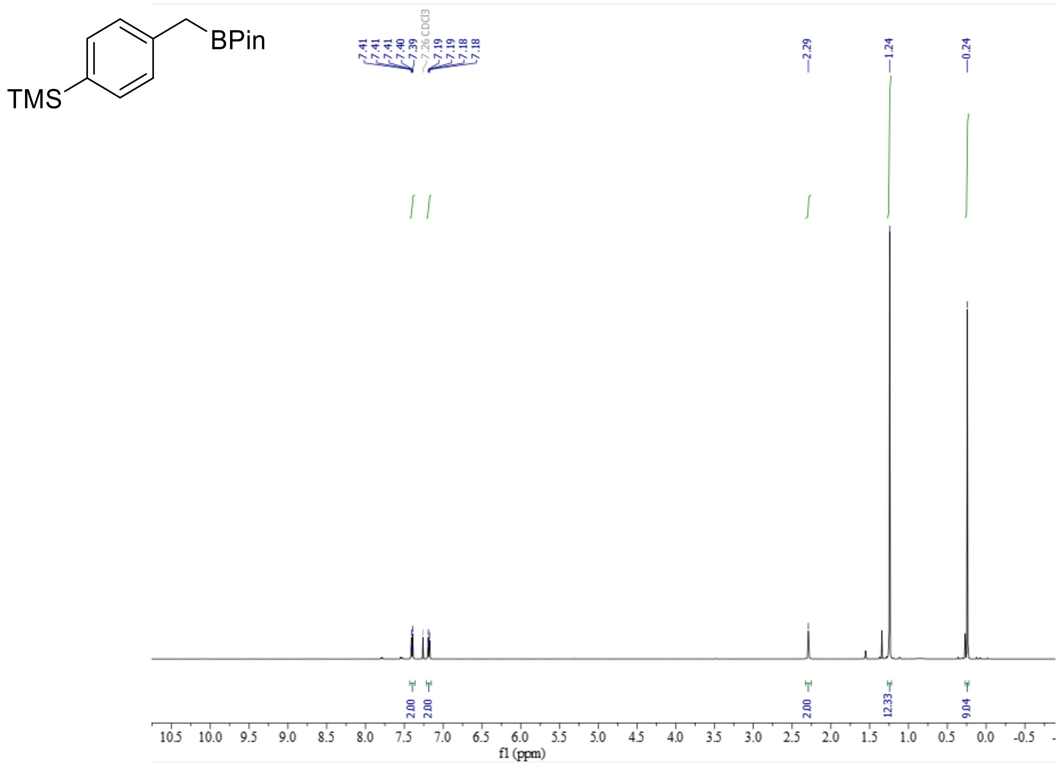

^{13}C NMR (126 MHz, CDCl$_3$)

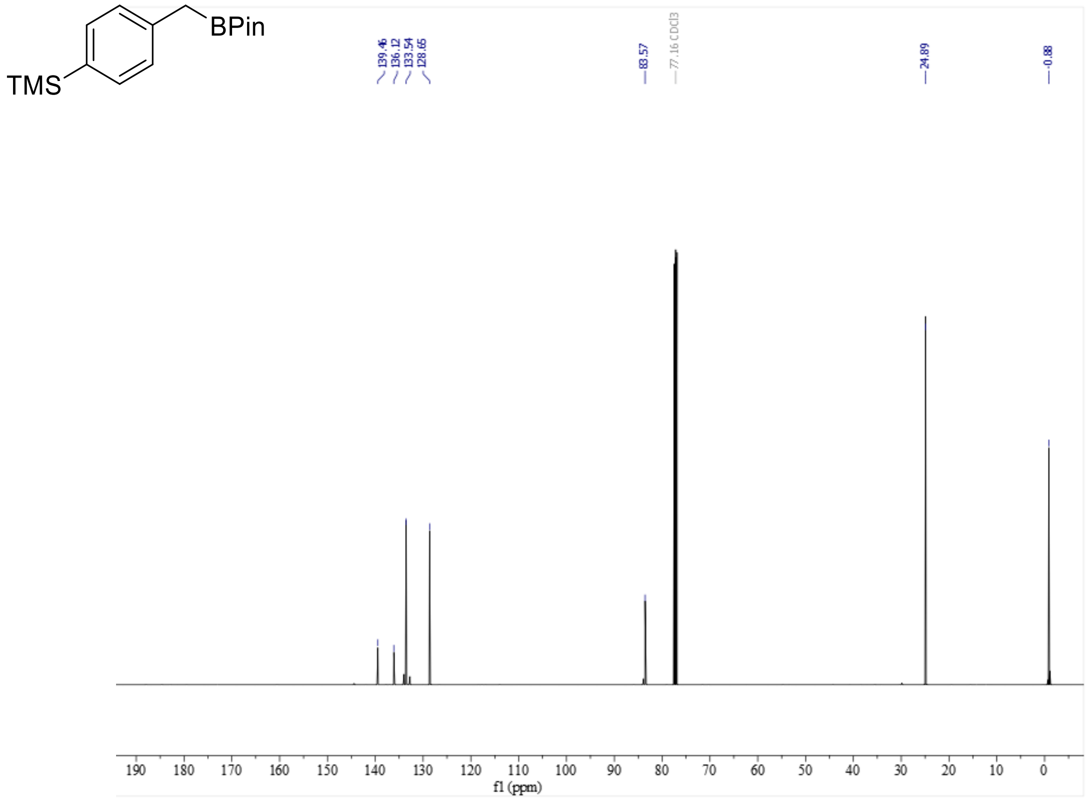

^{1}H-^{13}C-HSQC NMR – *α-boryl carbon atom highlighted*

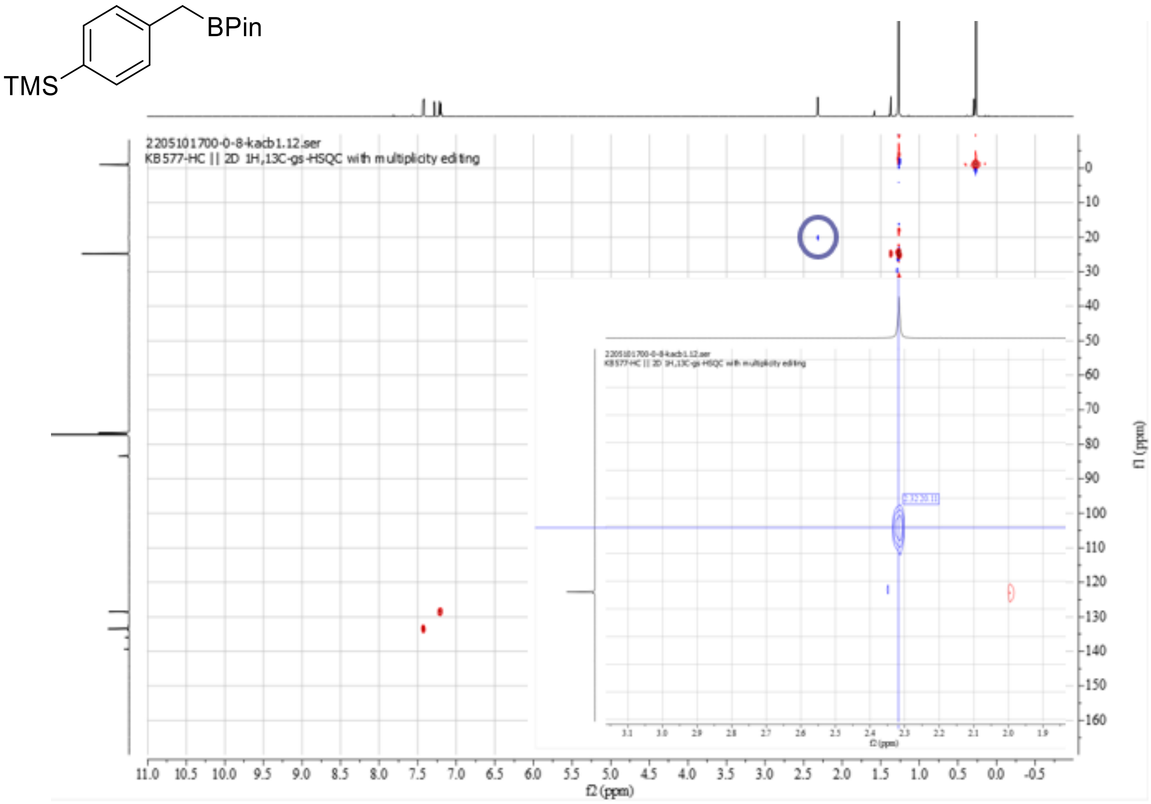

^{11}B NMR (96 MHz, $CDCl_3$)

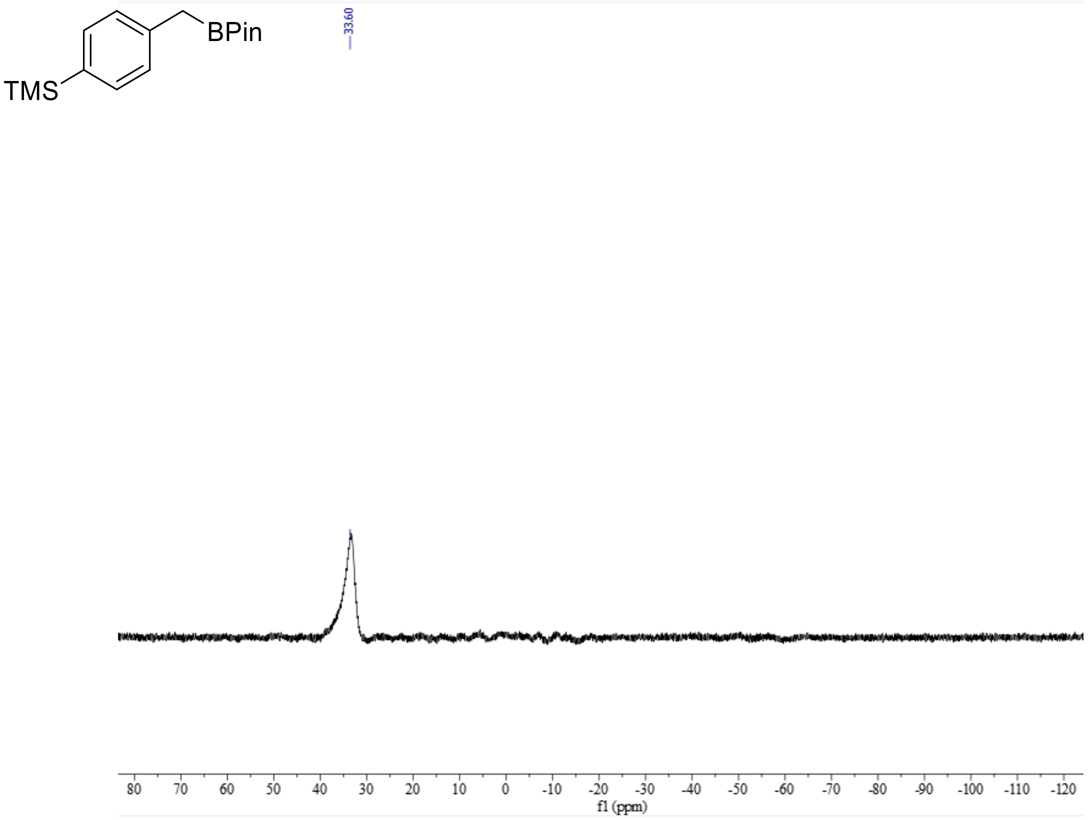

4,4,5,5-tetramethyl-2-(2-(methylthio)benzyl)-1,3,2-dioxaborolane (25)

^{1}H NMR (500 MHz, $CDCl_3$)

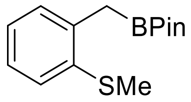

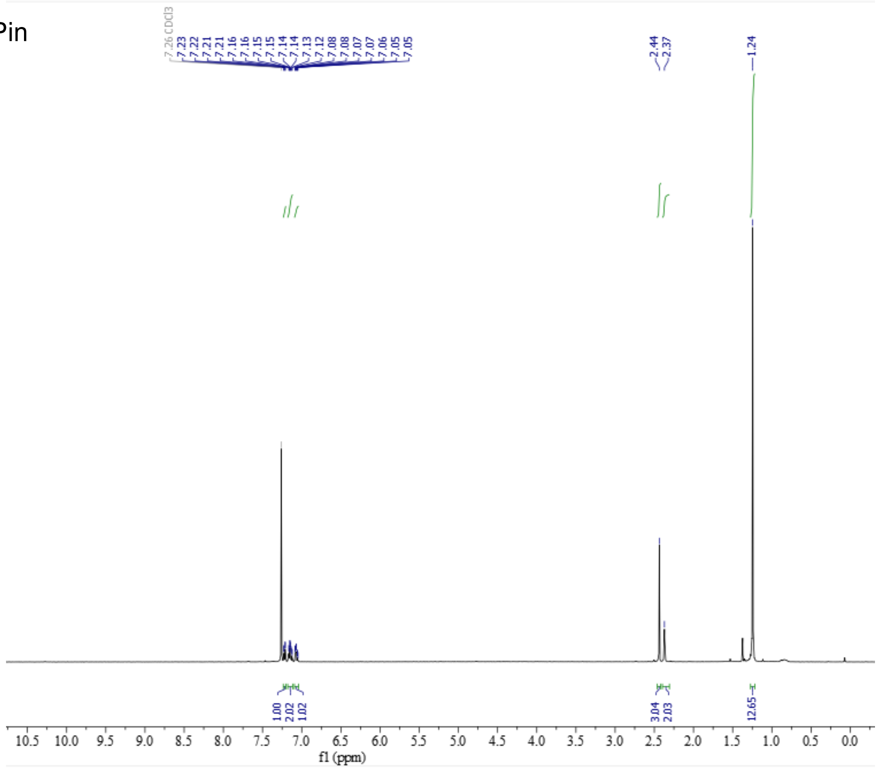

^{13}C NMR (126 MHz, $CDCl_3$)

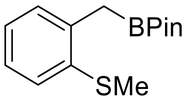

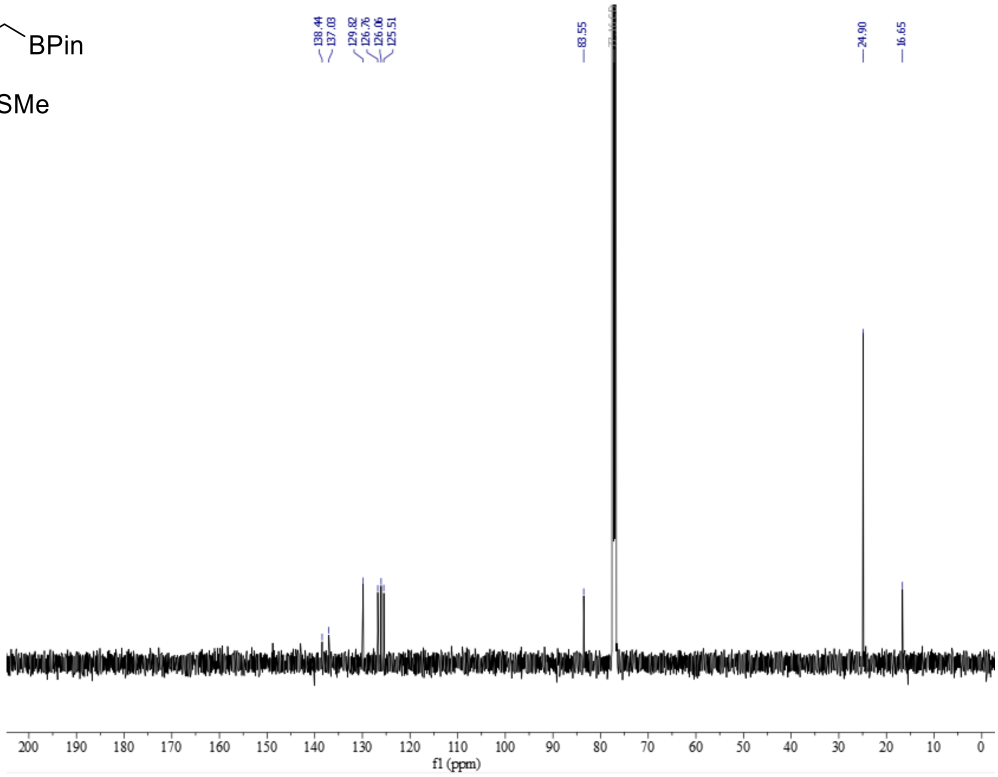

^{11}B NMR (96 MHz, $CDCl_3$)

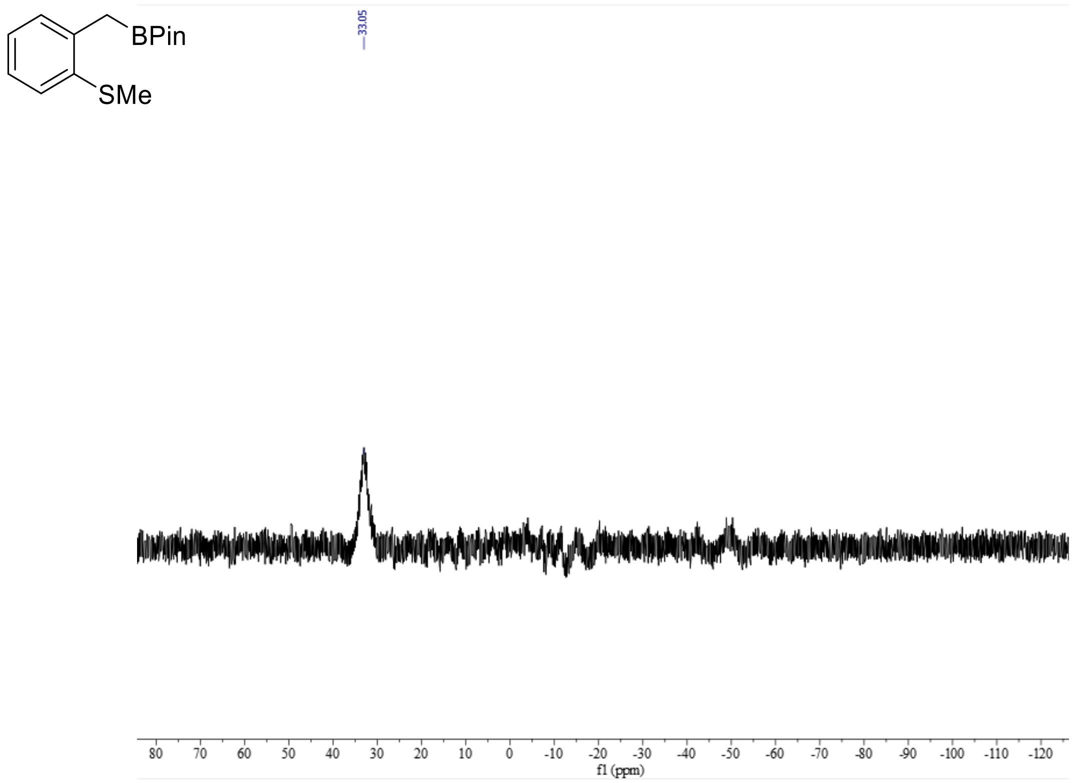

2-(3-fluoro-4-methoxybenzyl)-4,4,5,5-tetramethyl-1,3,2-dioxaborolane (26)

^{1}H NMR (500 MHz, $CDCl_3$)

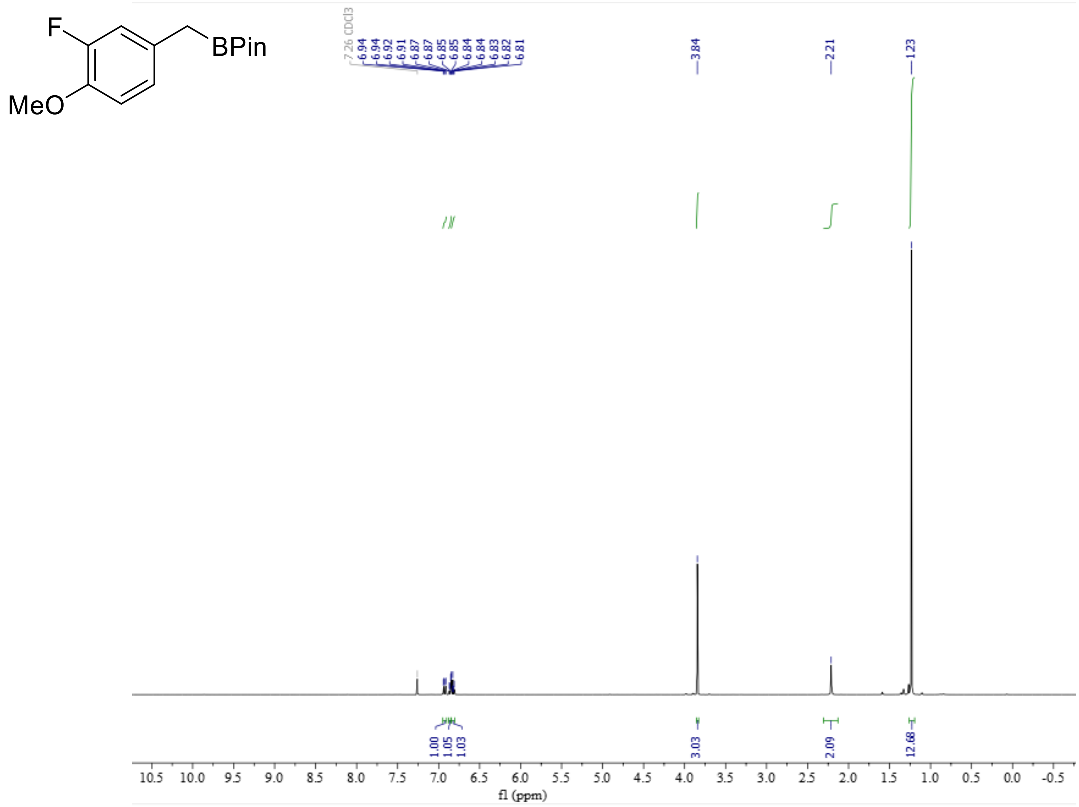

^{13}C NMR (126 MHz, CDCl$_3$)

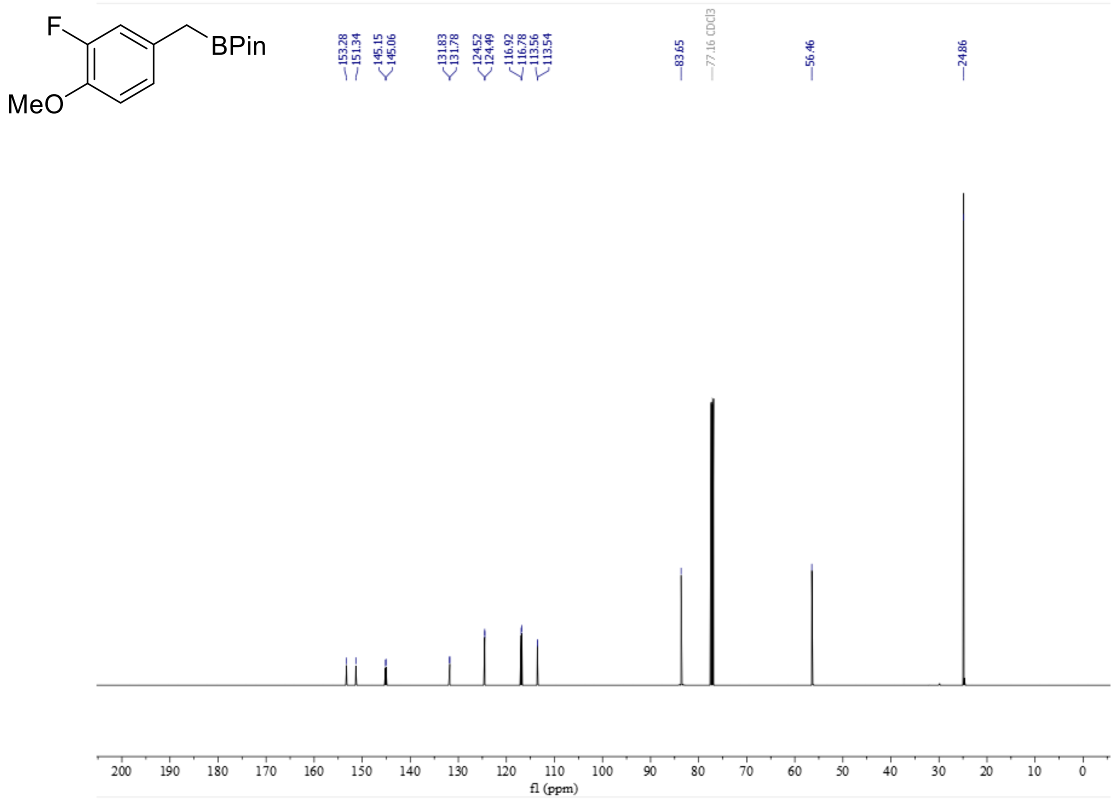

H-^{13}C-HSQC NMR – *α-boryl carbon atom highlighted*

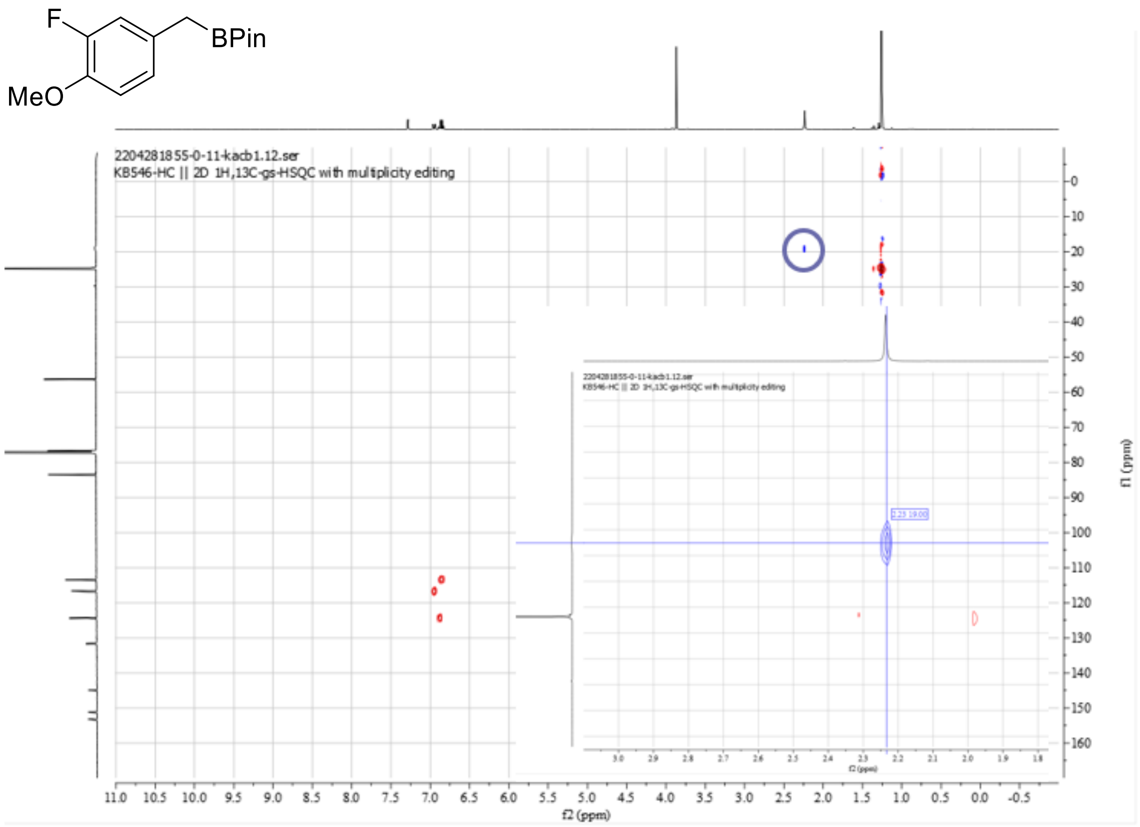

^{11}B NMR (96 MHz, $CDCl_3$)

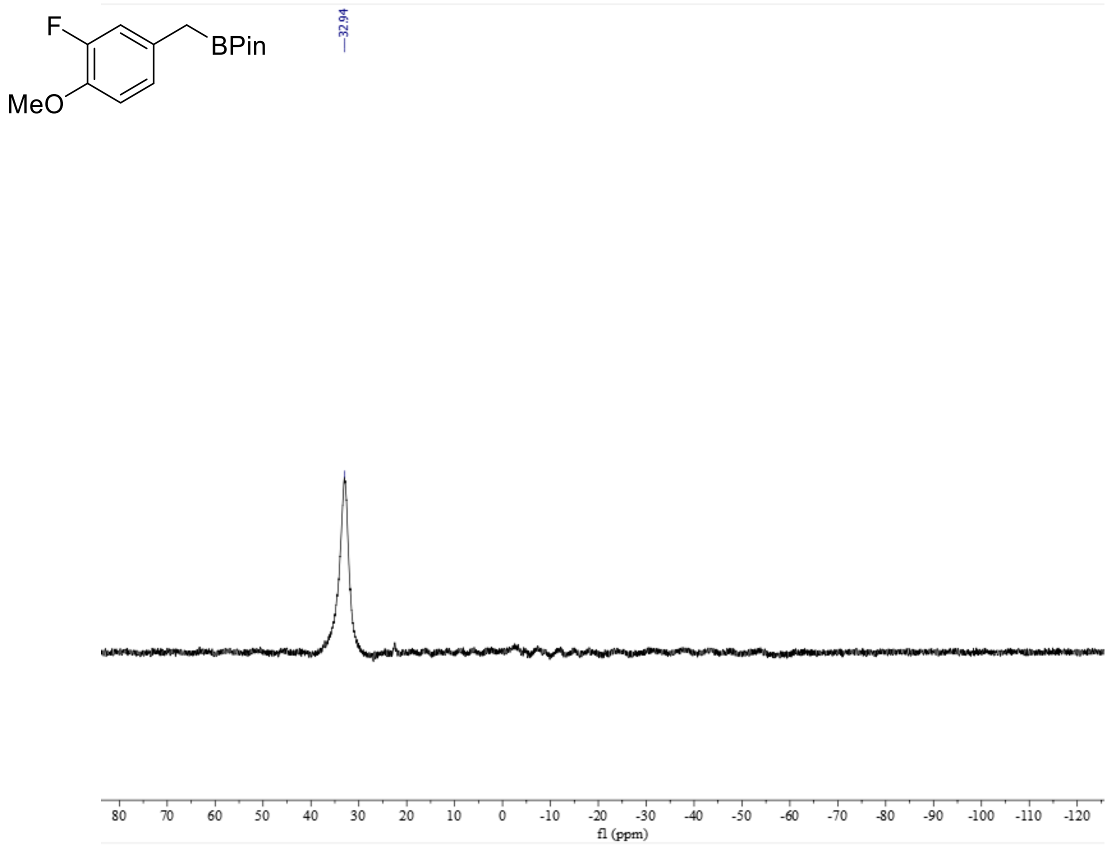

^{19}F NMR (470 MHz, $CDCl_3$)

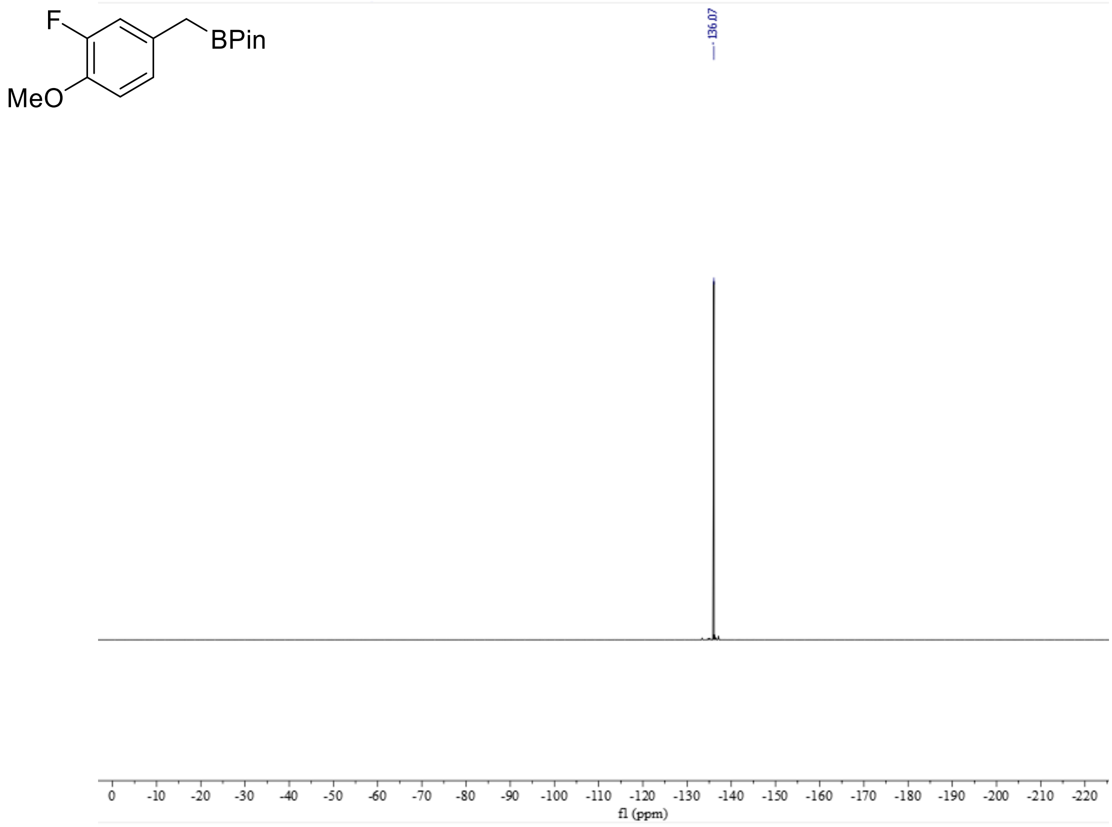

2-(4-fluoro-3-methoxybenzyl)-4,4,5,5-tetramethyl-1,3,2-dioxaborolane (27)

^{1}H NMR (500 MHz, $CDCl_3$)

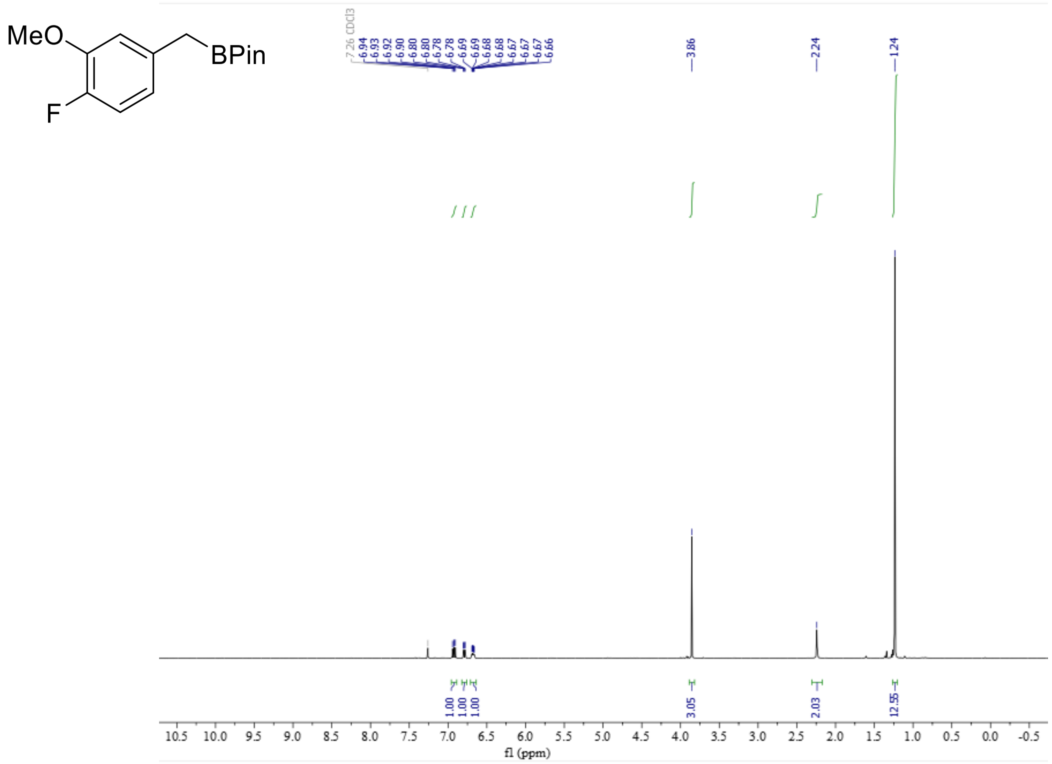

^{13}C NMR (126 MHz, $CDCl_3$)

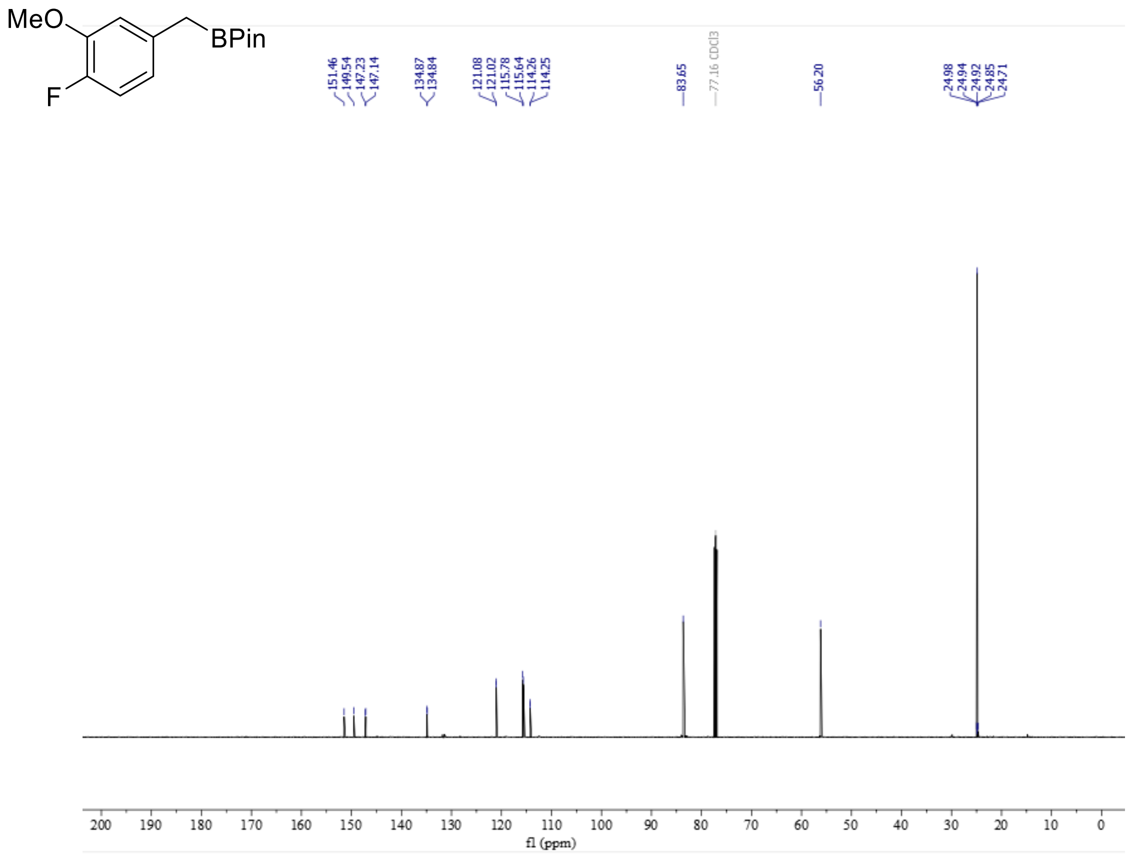

H-^{13}C-HSQC NMR – *α-boryl carbon atom highlighted*

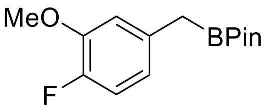

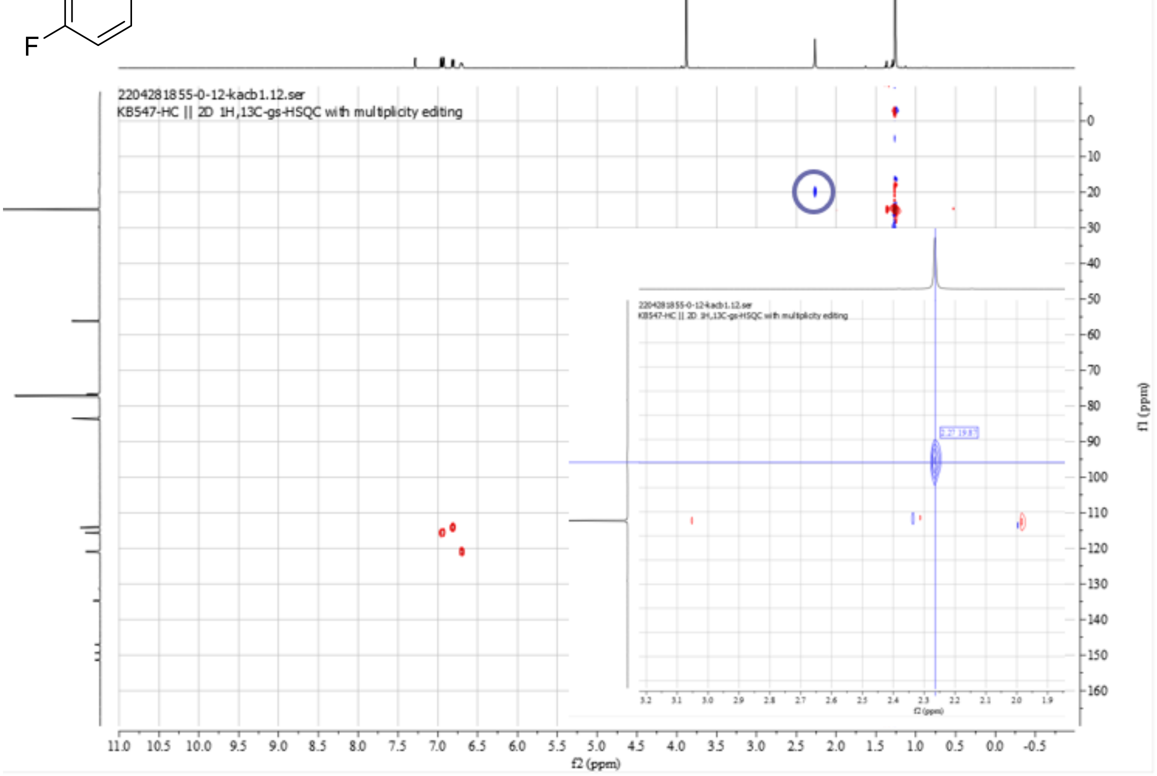

^{11}B NMR (96 MHz, $CDCl_3$)

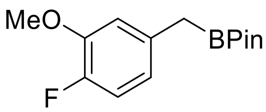

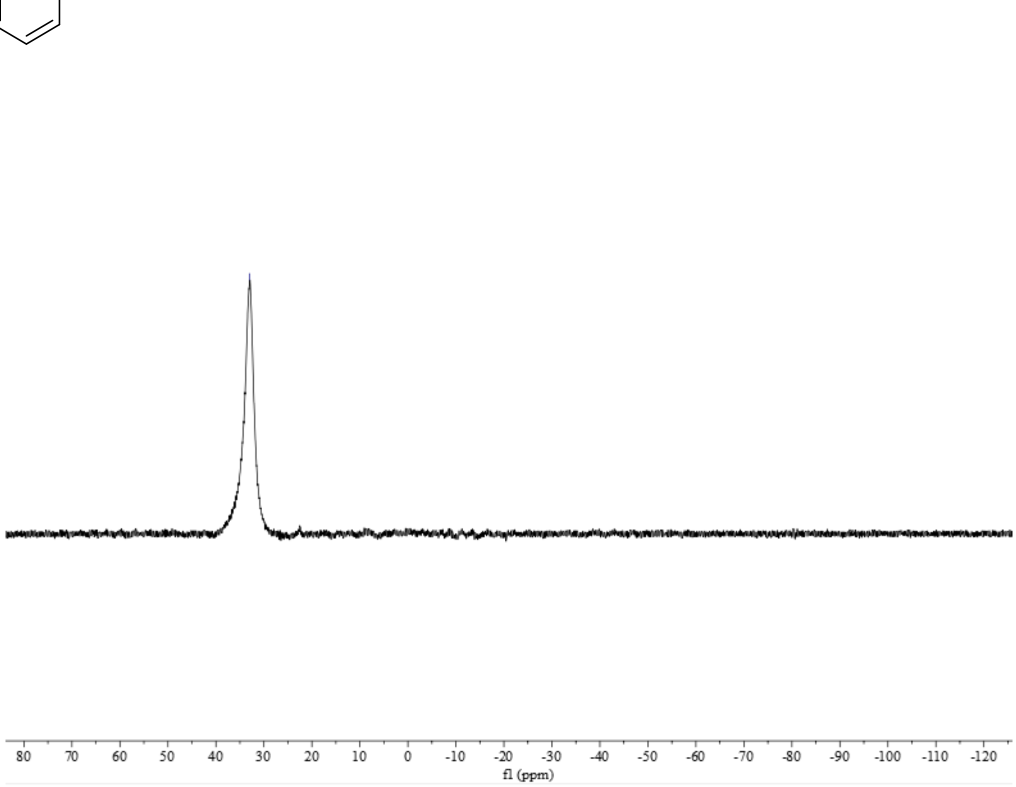

^{19}F NMR (470 MHz, CDCl$_3$)

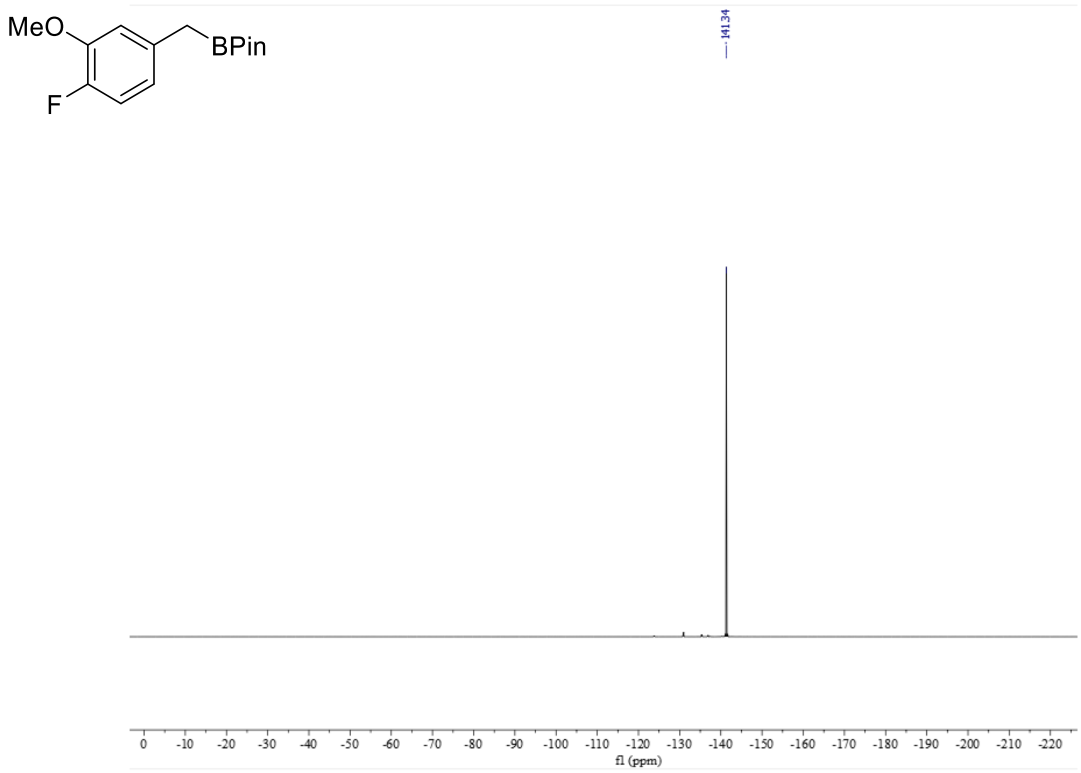

4,4,5,5-tetramethyl-2-(4-(trifluoromethoxy)benzyl)-1,3,2-dioxaborolane (28)

^{1}H NMR (500 MHz, CDCl$_3$)

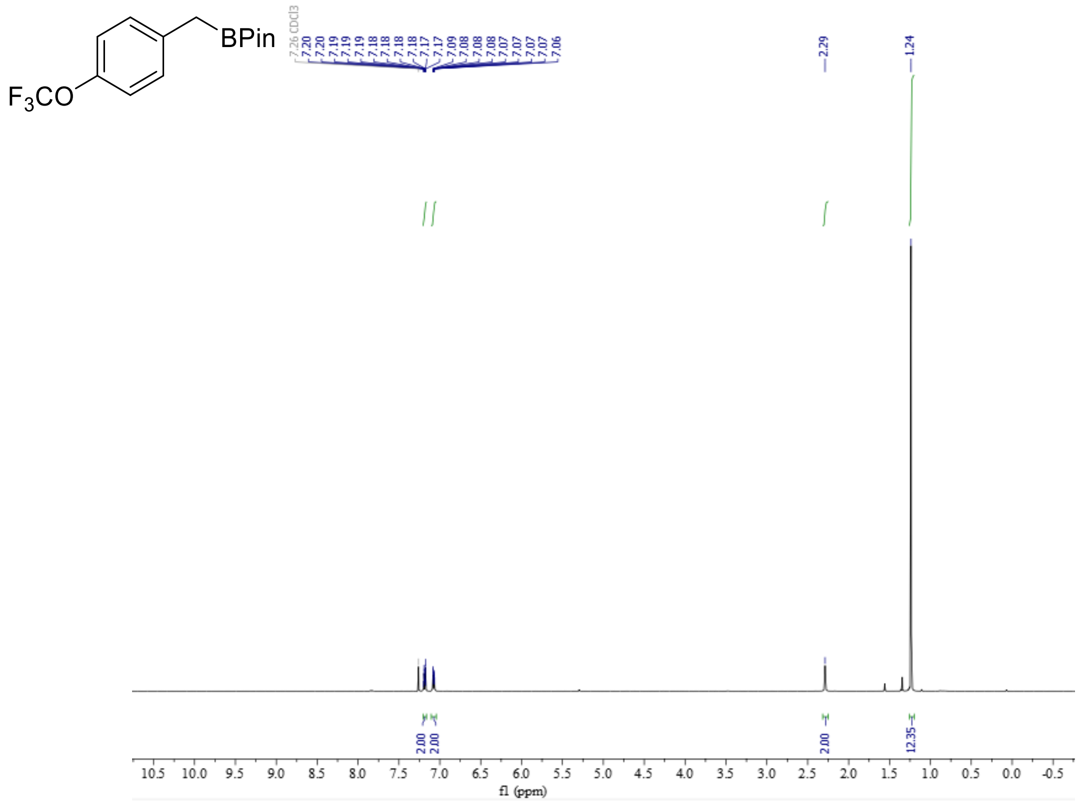

^{13}C NMR (126 MHz, CDCl$_3$)

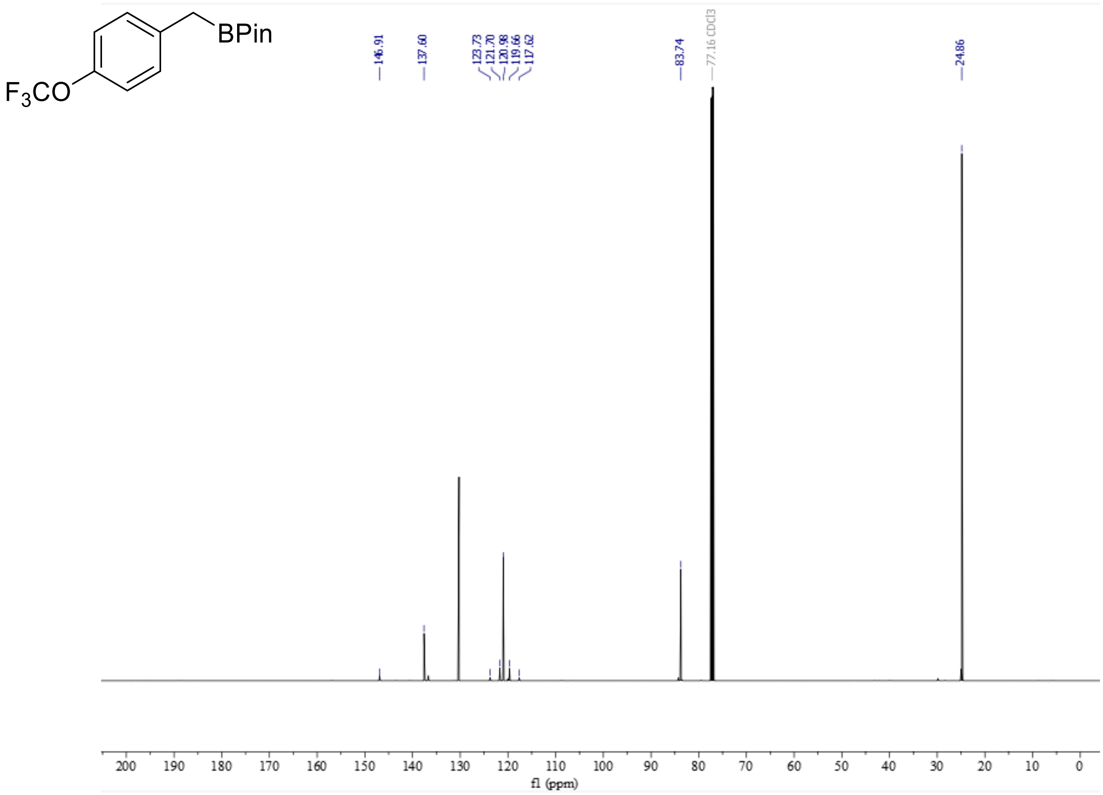

^{1}H-^{13}C-HSQC NMR – *α-boryl carbon atom highlighted*

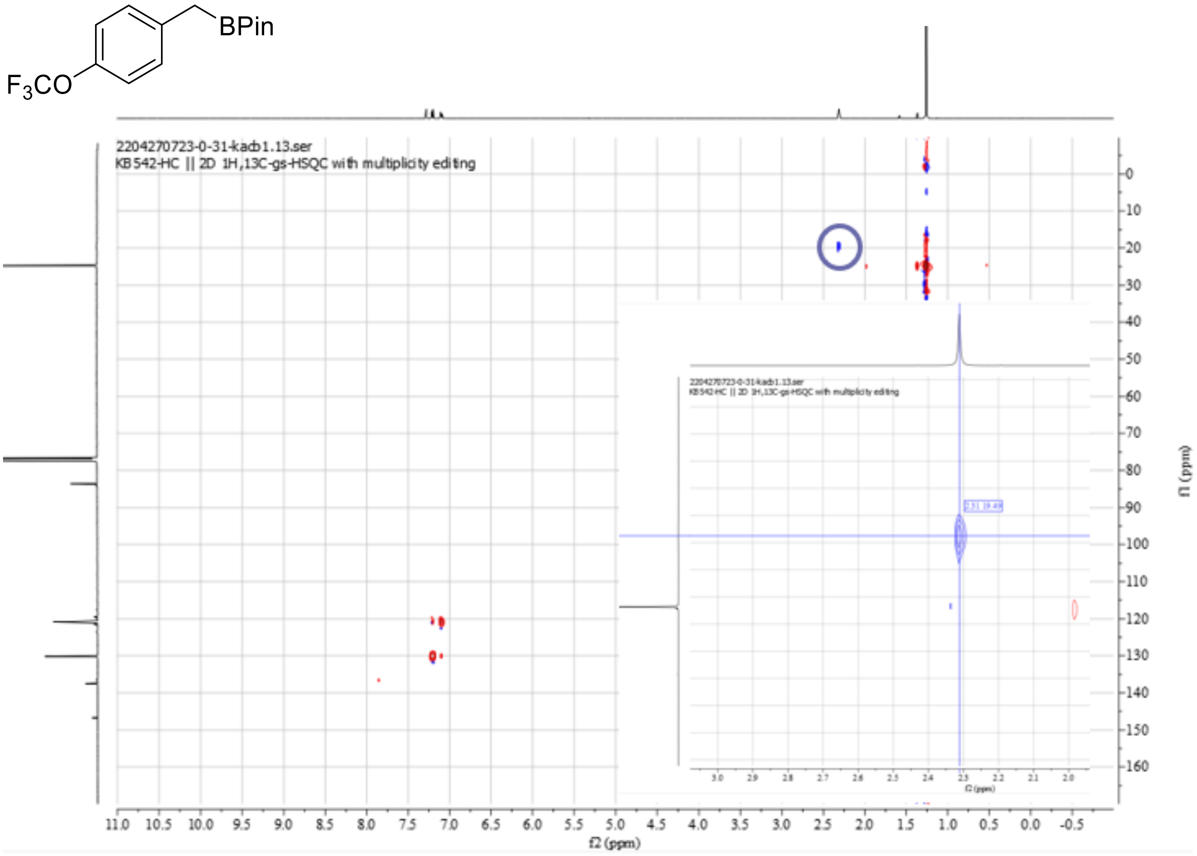

^{11}B NMR (96 MHz, $CDCl_3$)

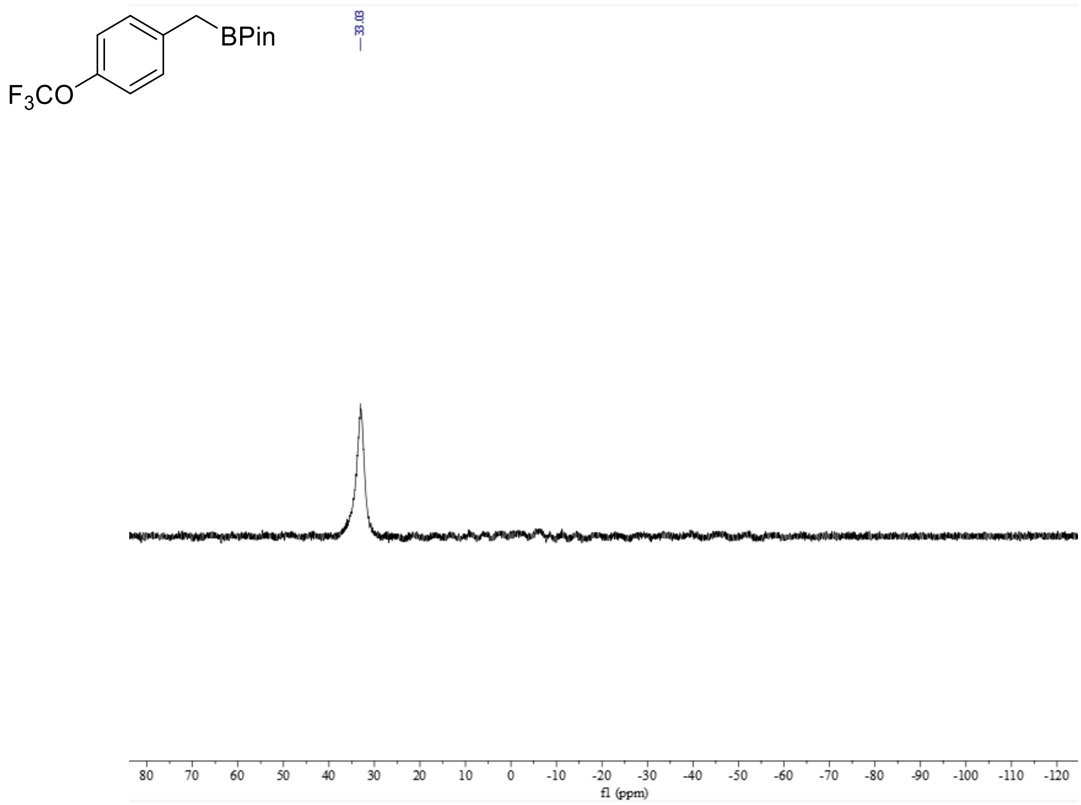

^{19}F NMR (470 MHz, $CDCl_3$)

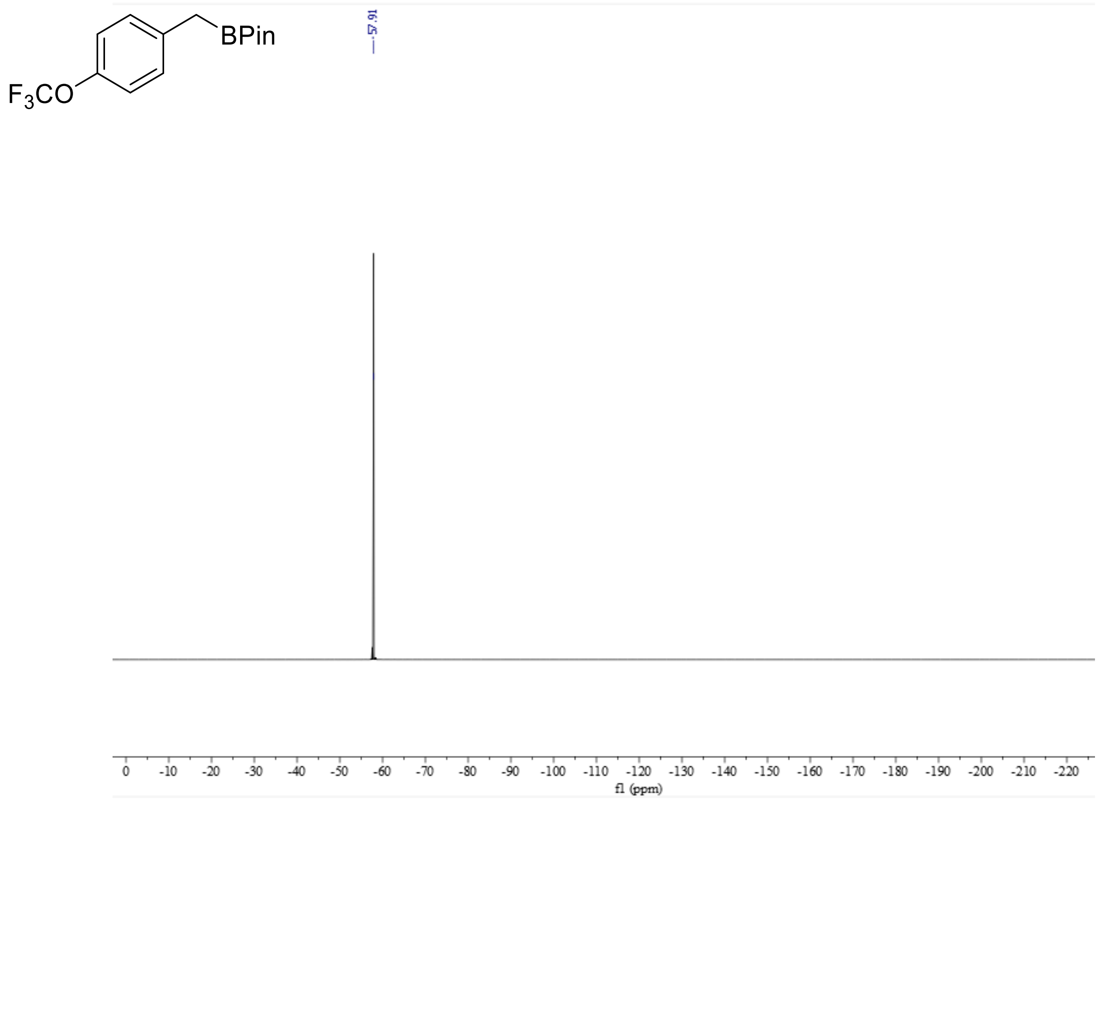

2-(4-fluorobenzyl)-4,4,5,5-tetramethyl-1,3,2-dioxaborolane (29)

^{1}H NMR (500 MHz, $CDCl_3$)

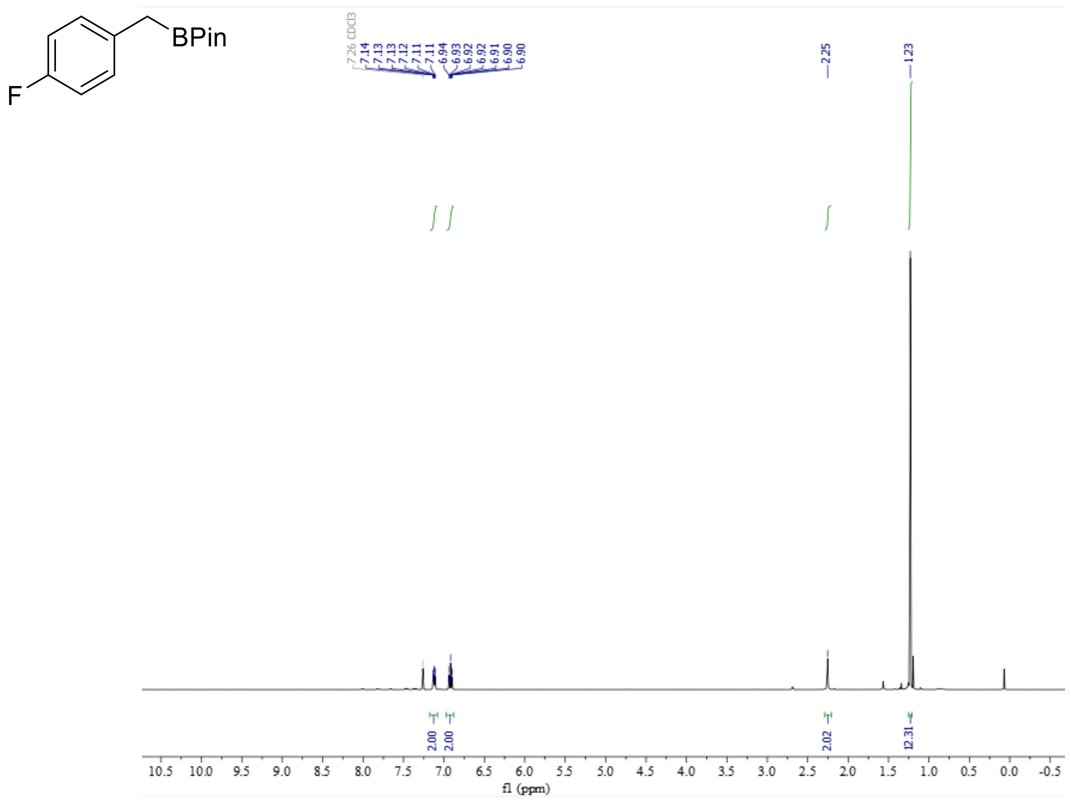

^{13}C NMR (126 MHz, $CDCl_3$)

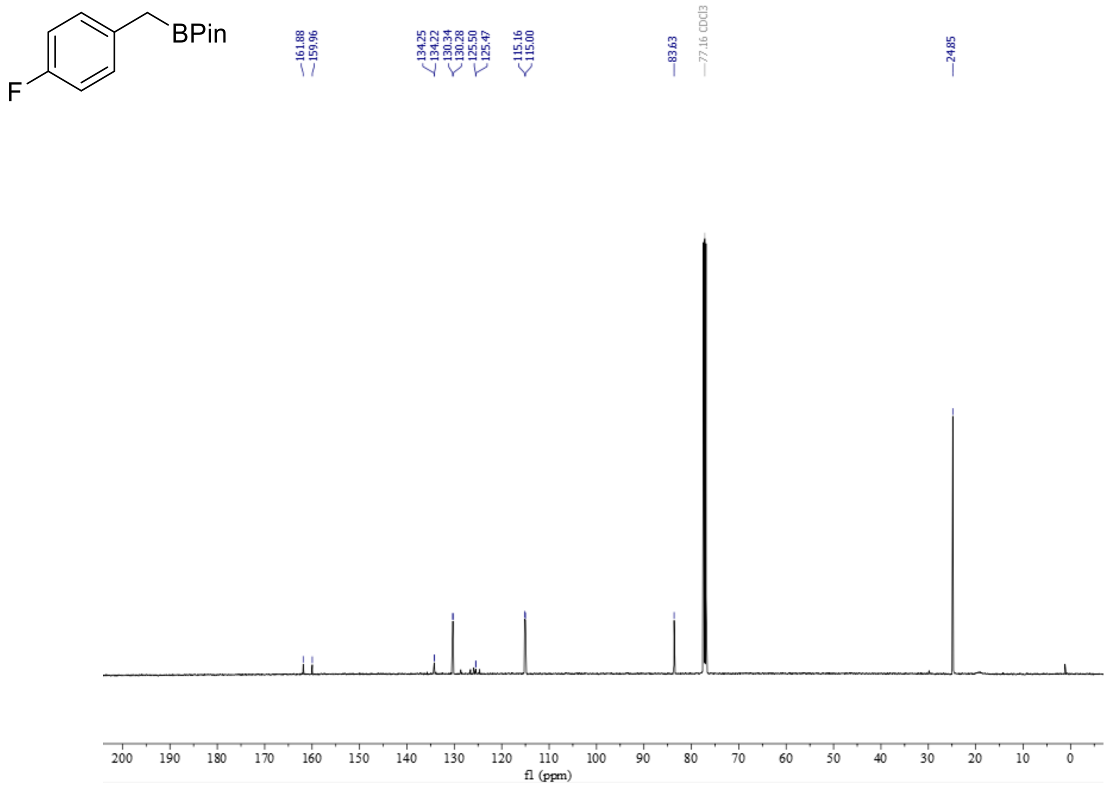

^{11}B NMR (96 MHz, $CDCl_3$)

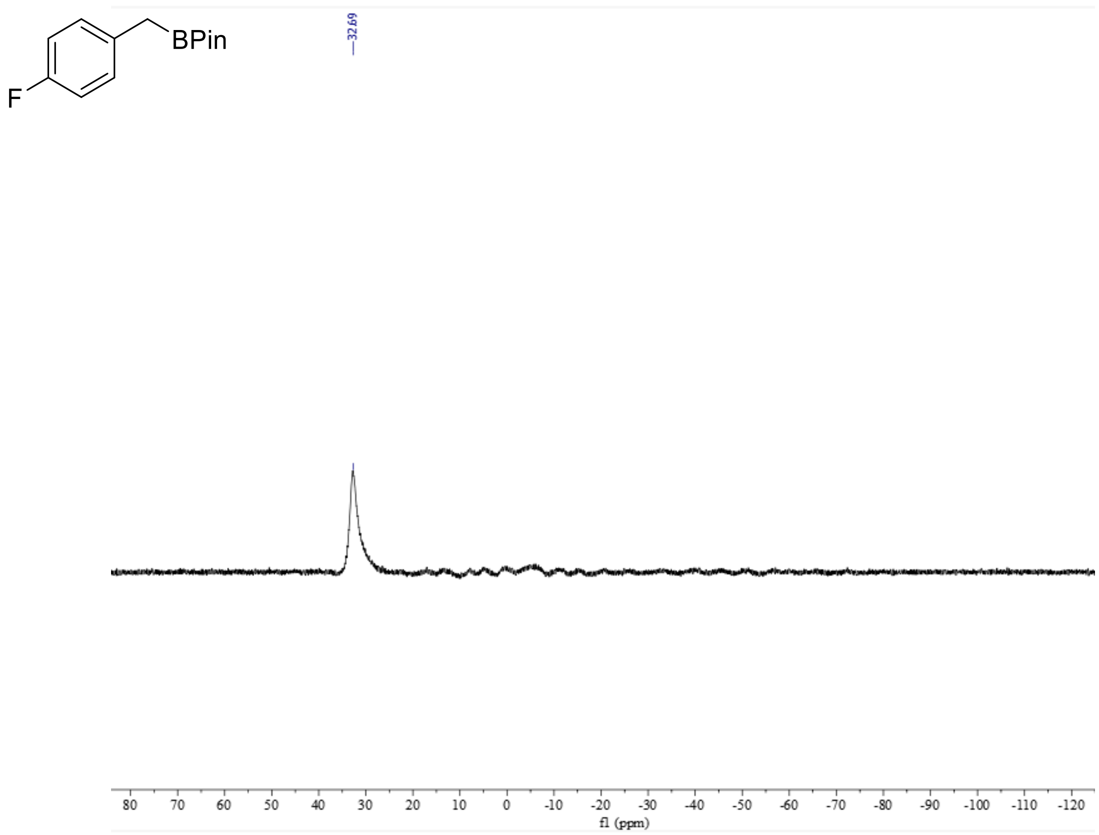

^{19}F NMR (376 MHz, $CDCl_3$)

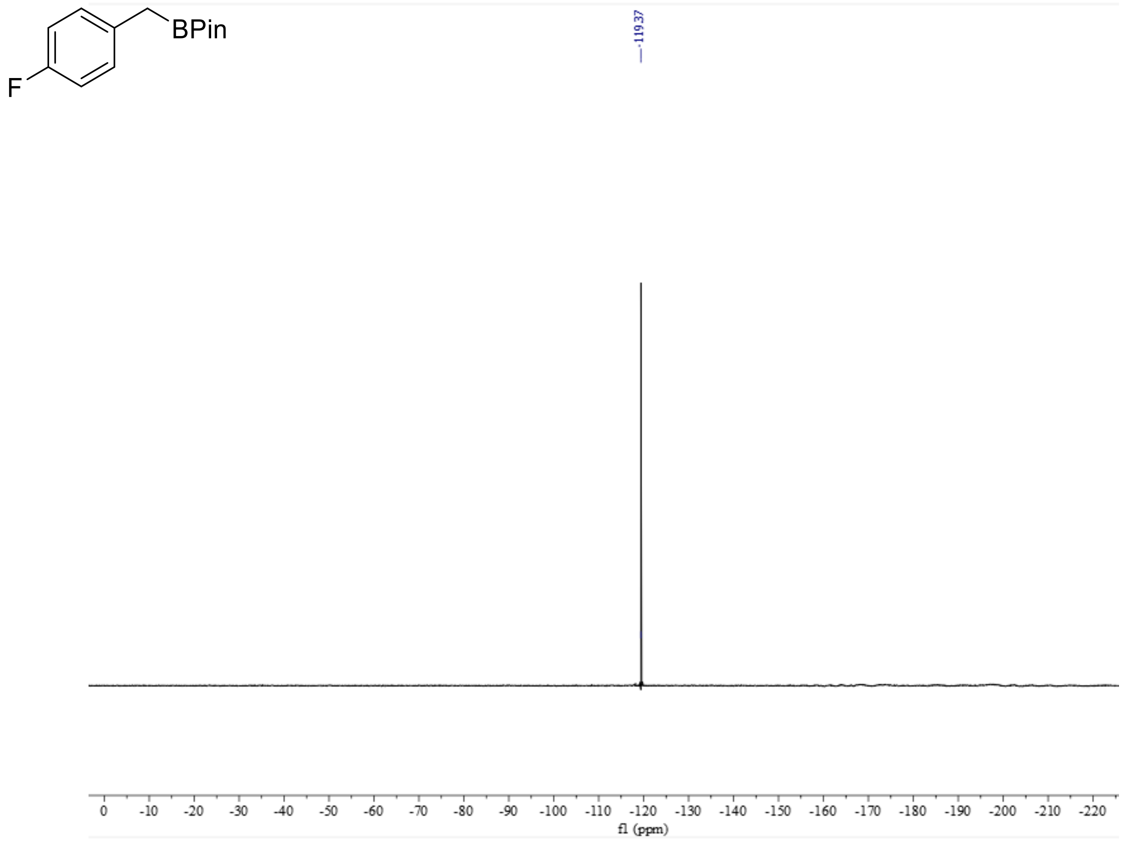

2-(3-fluorobenzyl)-4,4,5,5-tetramethyl-1,3,2-dioxaborolane (30)

^{1}H NMR (500 MHz, $CDCl_3$)

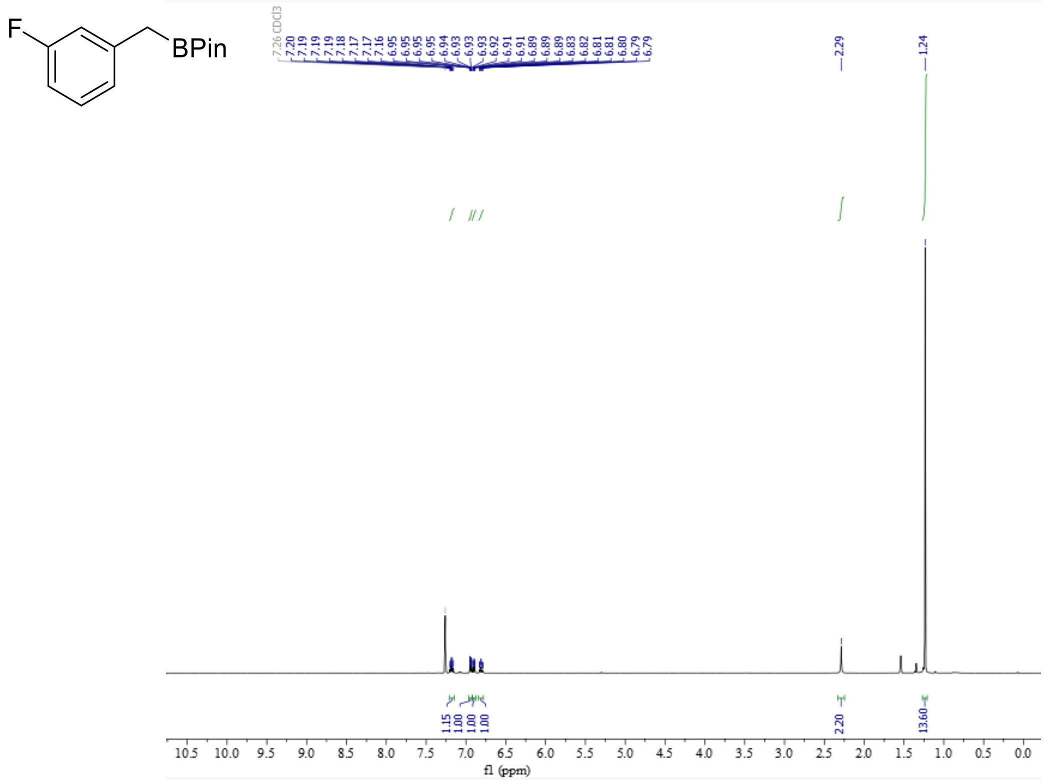

^{13}C NMR (126 MHz, $CDCl_3$)

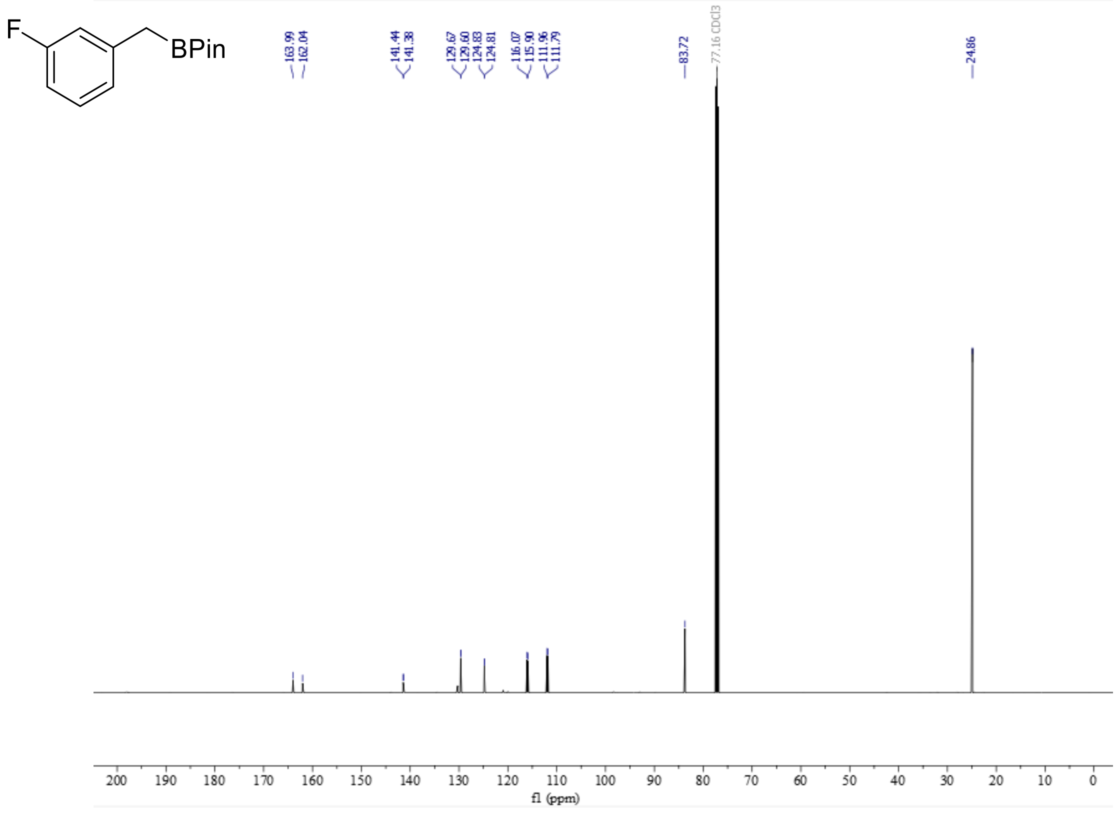

^{1}H-^{13}C-**HSQC NMR** – *α-boryl carbon atom highlighted*

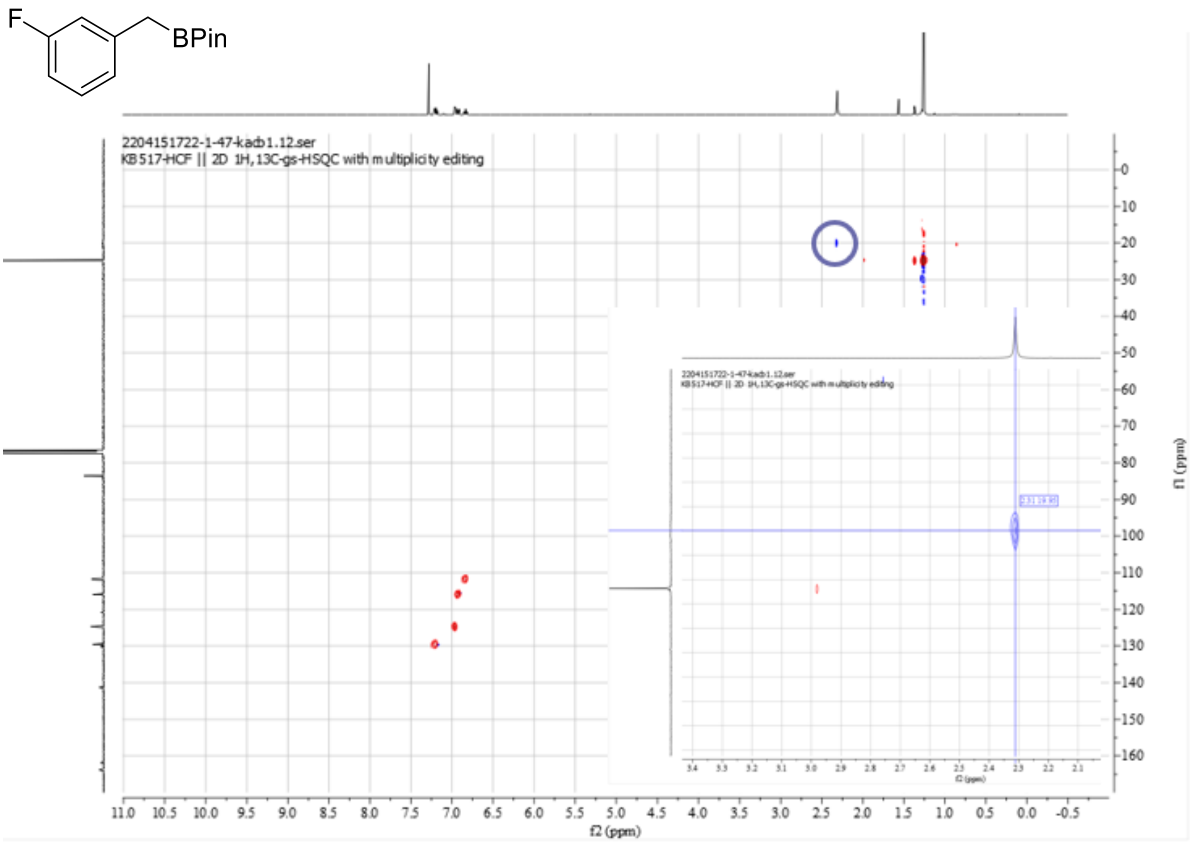

^{11}B **NMR (96 MHz,** $CDCl_3$**)**

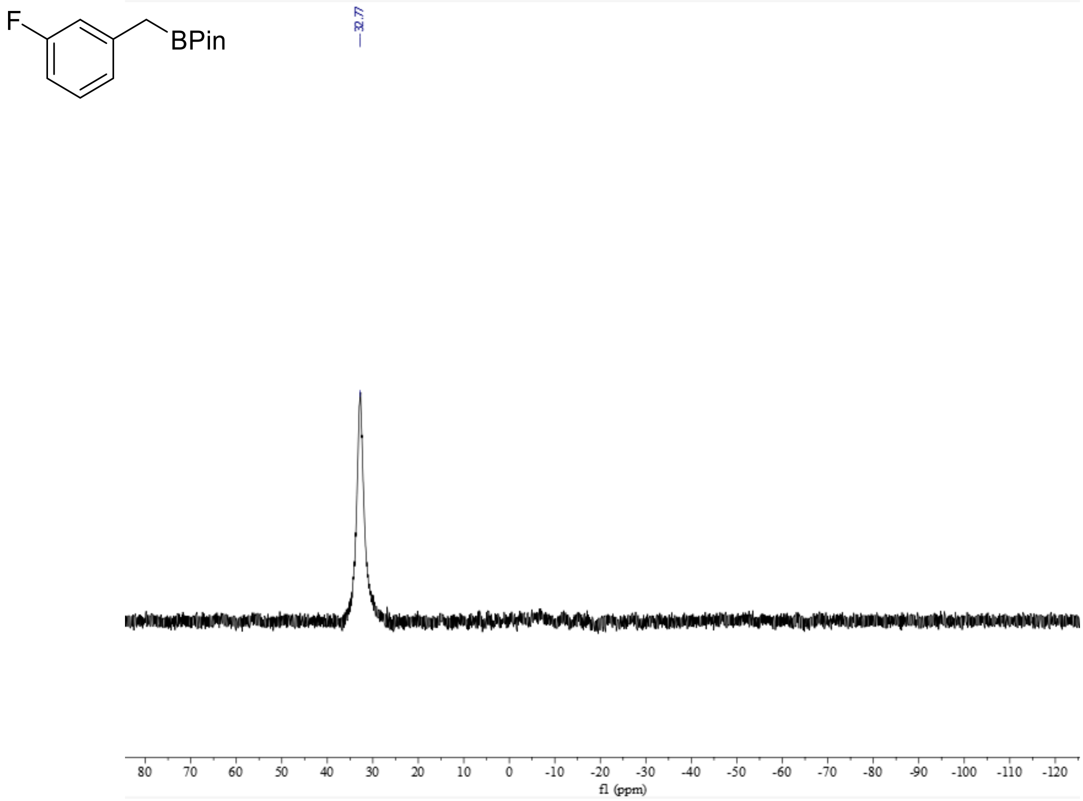

^{19}F NMR (376 MHz, $CDCl_3$)

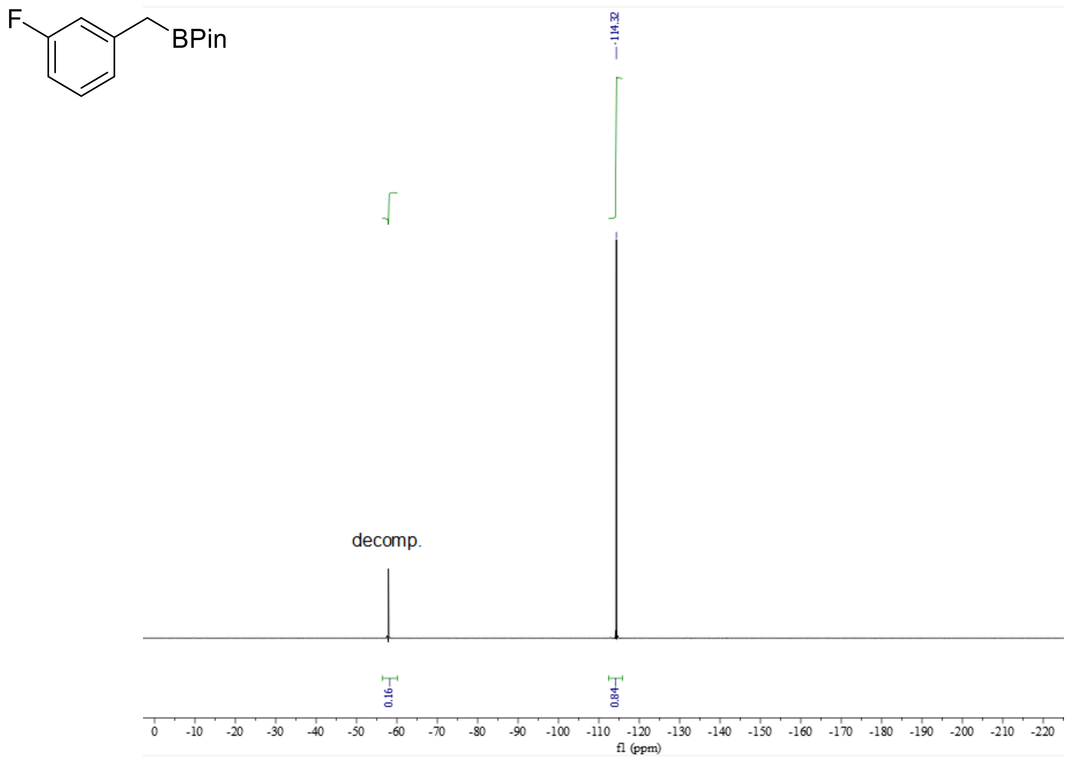

2-(2-chloro-6-propoxybenzyl)-4,4,5,5-tetramethyl-1,3,2-dioxaborolane (31)

^{1}H NMR (500 MHz, $CDCl_3$)

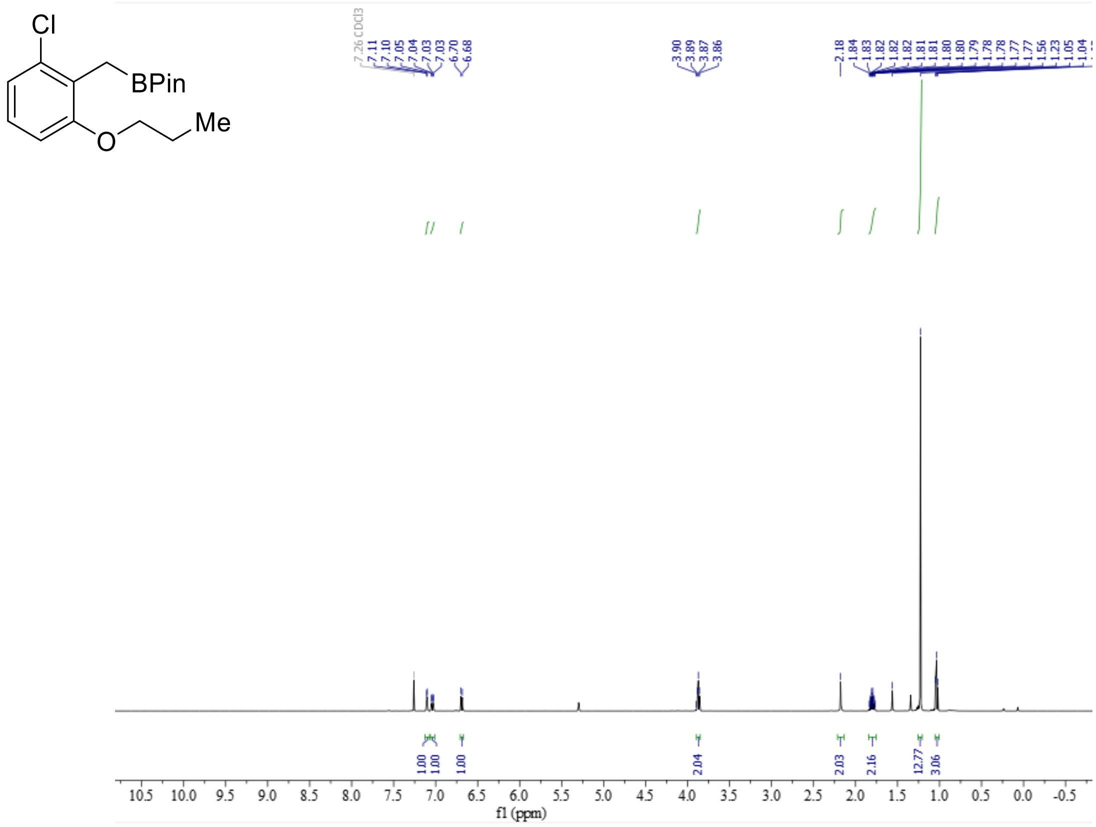

^{13}C NMR (126 MHz, CDCl$_3$)

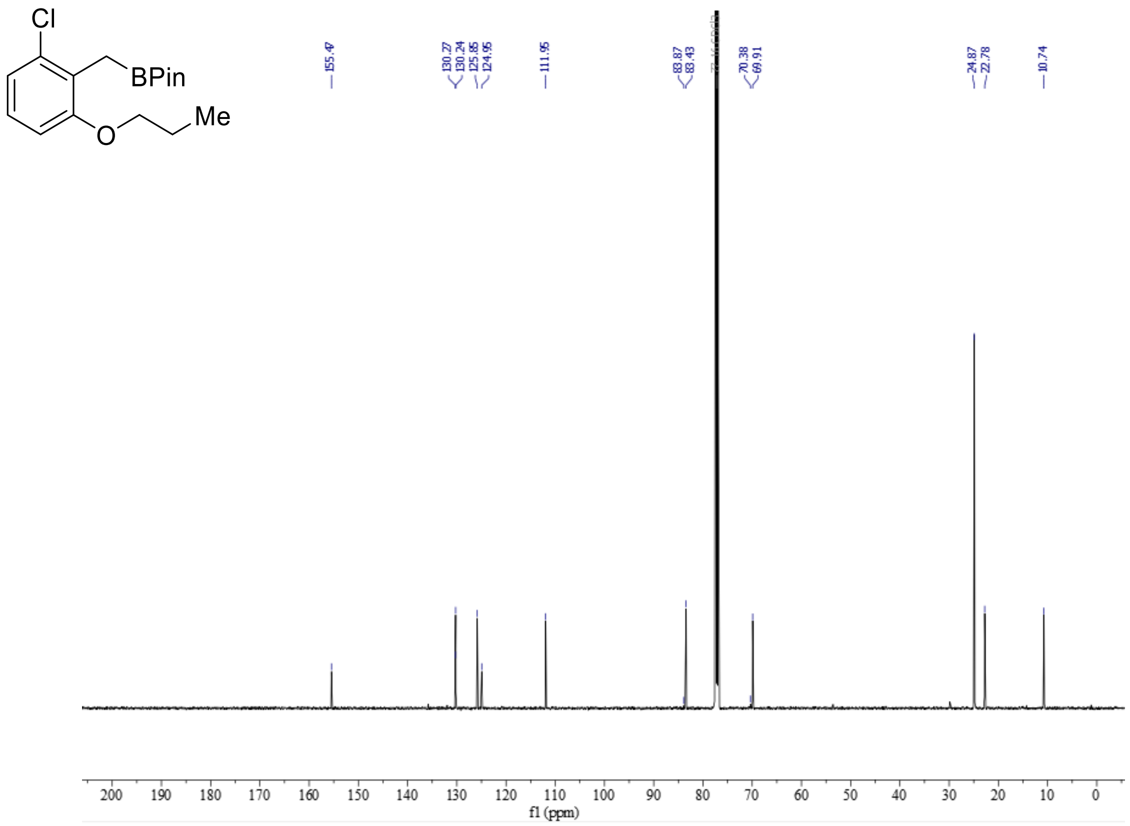

^{1}H-^{13}C-HSQC NMR – *α-boryl carbon atom highlighted*

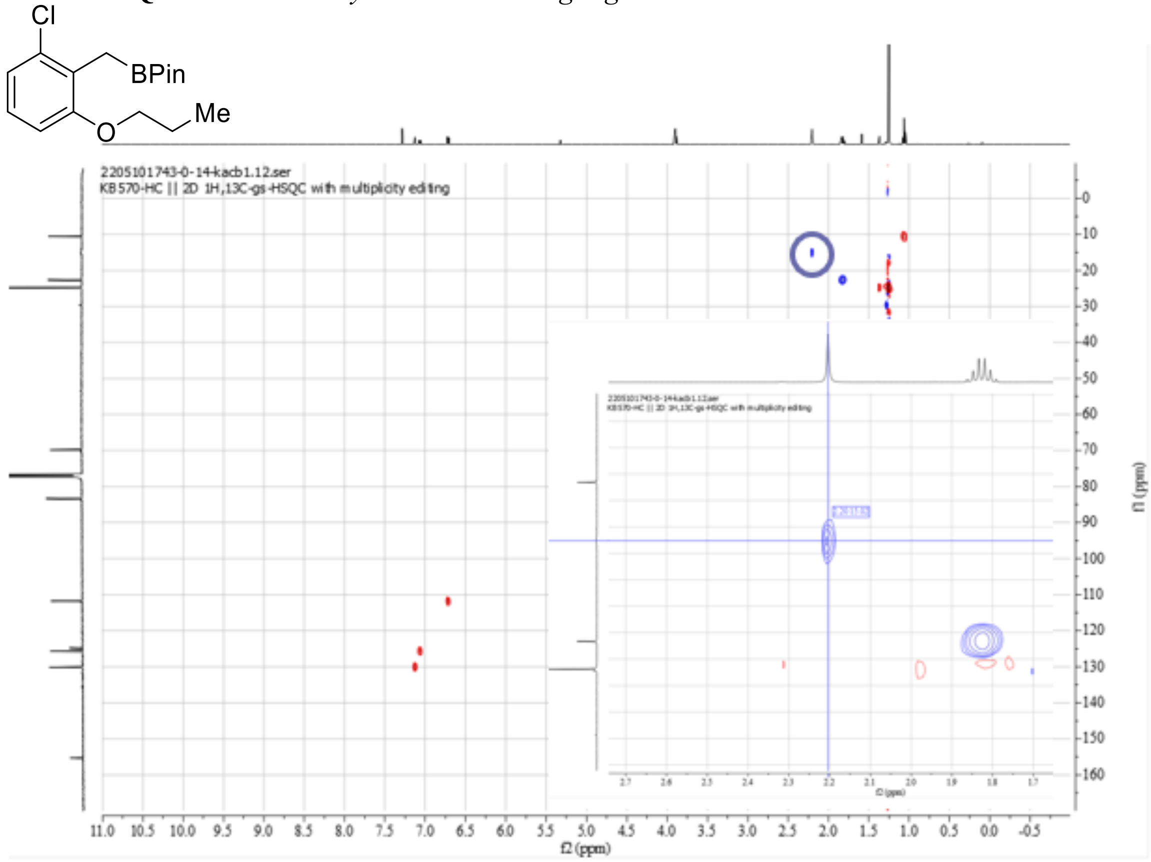

^{11}B NMR (96 MHz, CDCl$_3$)

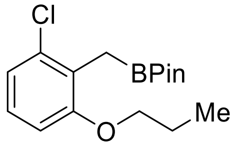

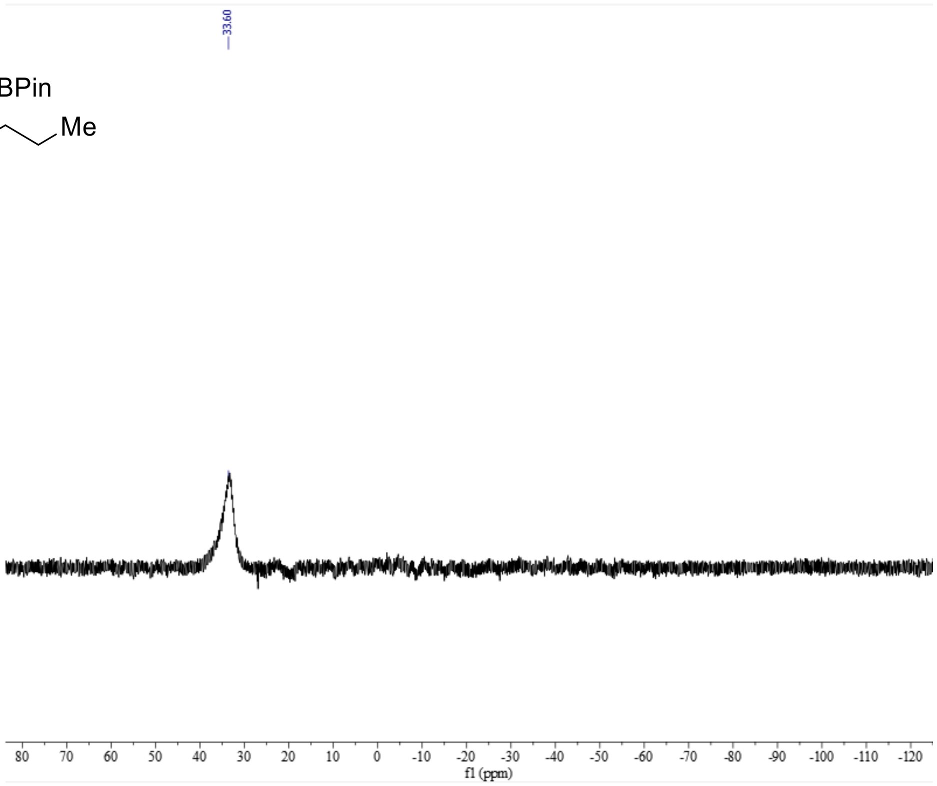

2-(2-fluorobenzyl)-4,4,5,5-tetramethyl-1,3,2-dioxaborolane (32)

^{1}H NMR (500 MHz, CDCl$_3$)

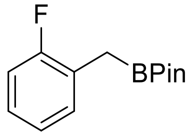

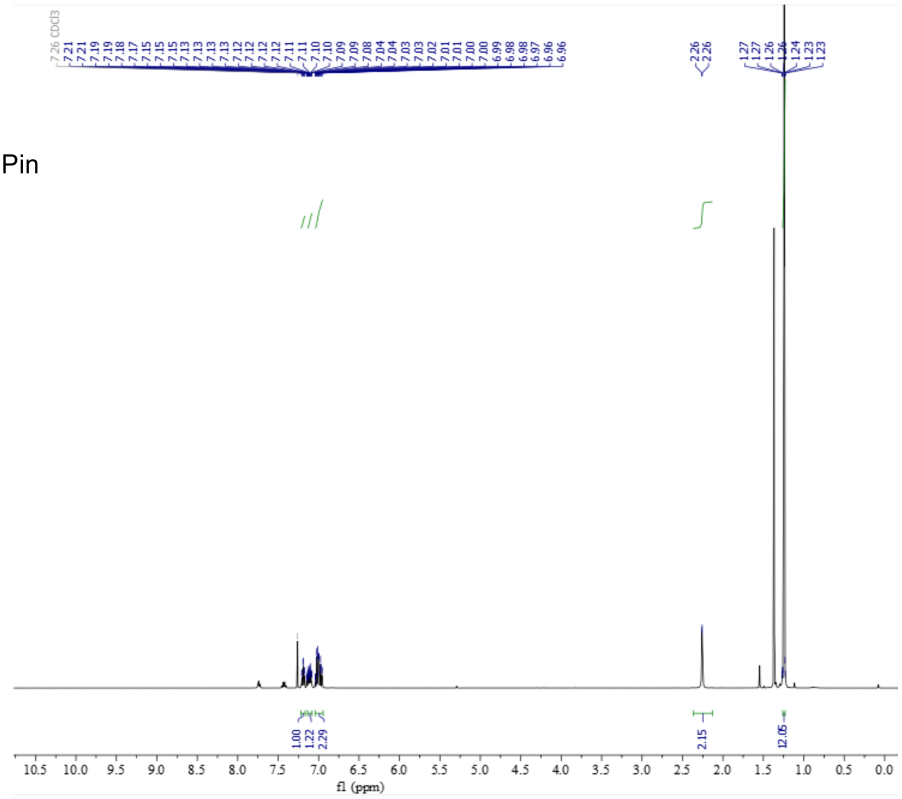

^{13}C NMR (126 MHz, CDCl$_3$)

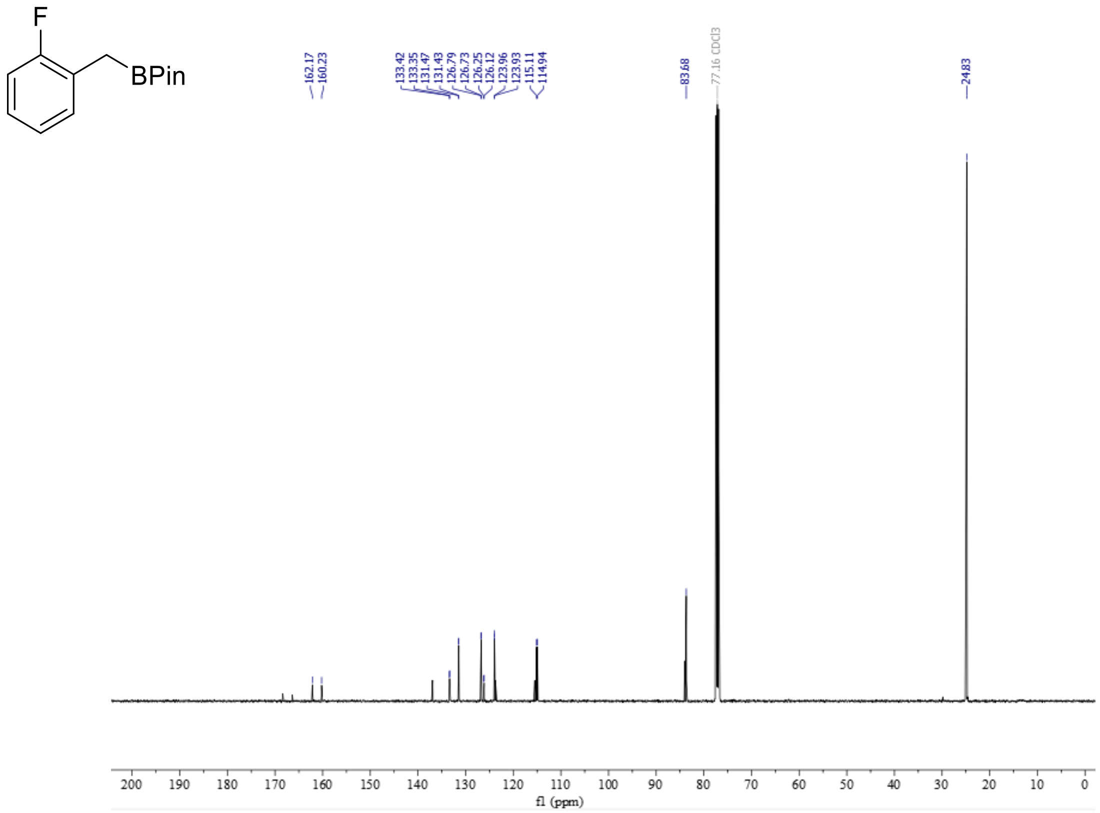

^{1}H-^{13}C-HSQC NMR – *α-boryl carbon atom highlighted*

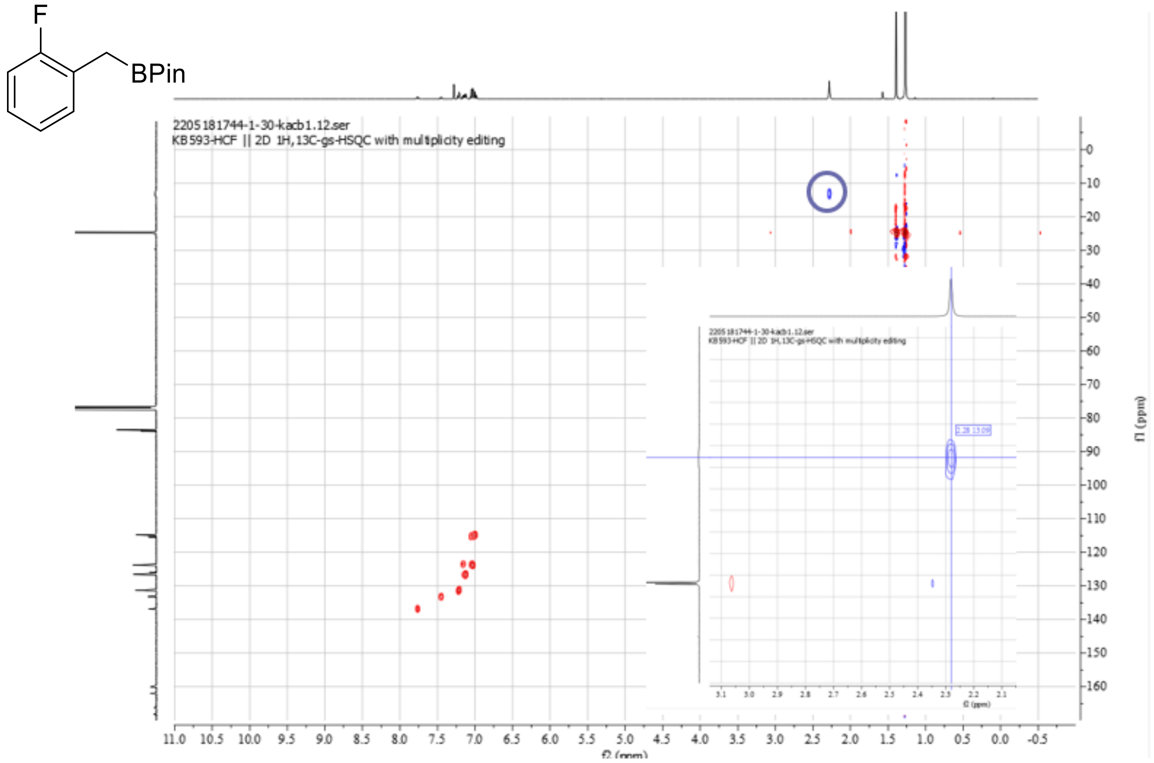

^{11}B NMR (96 MHz, $CDCl_3$)

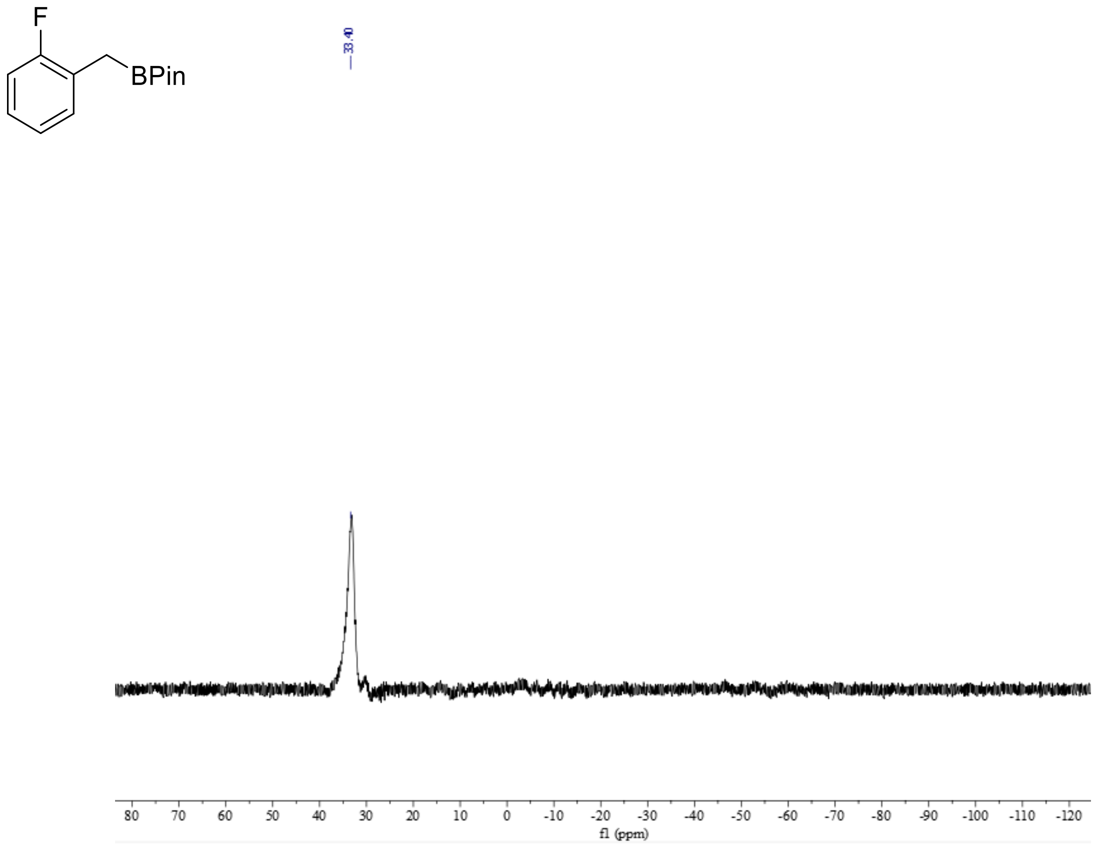

^{19}F NMR (376 MHz, $CDCl_3$)

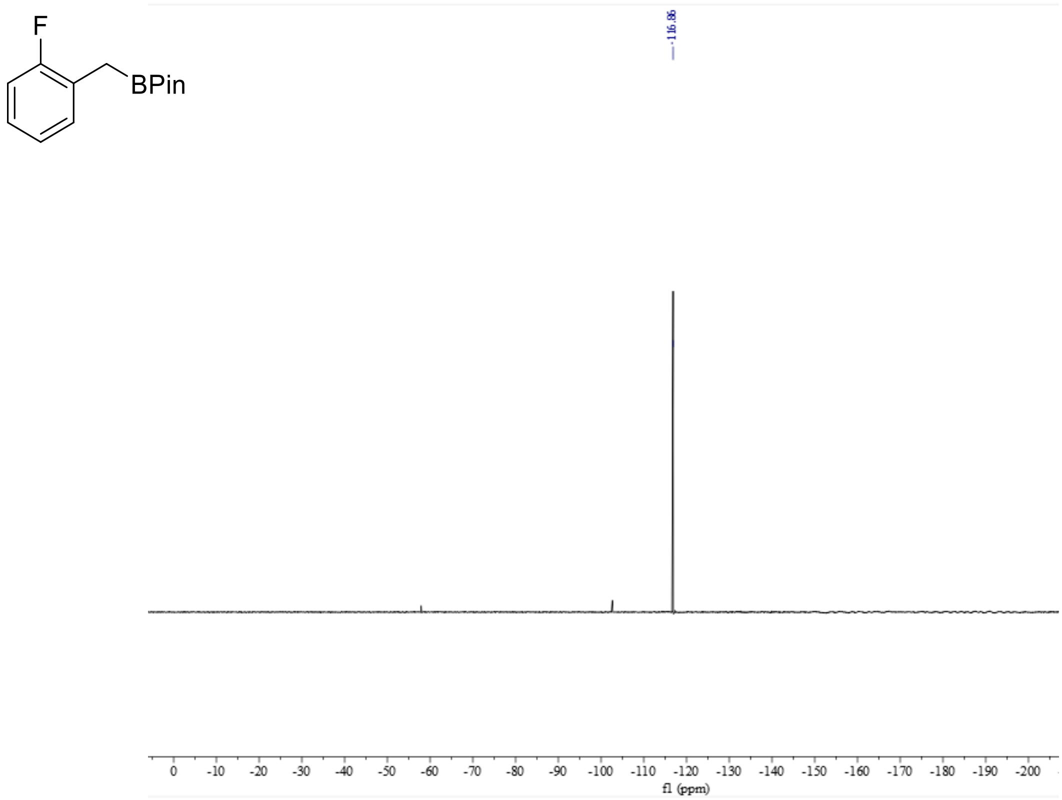

2-(4-chlorobenzyl)-4,4,5,5-tetramethyl-1,3,2-dioxaborolane (33)

^{1}H NMR (500 MHz, $CDCl_3$)

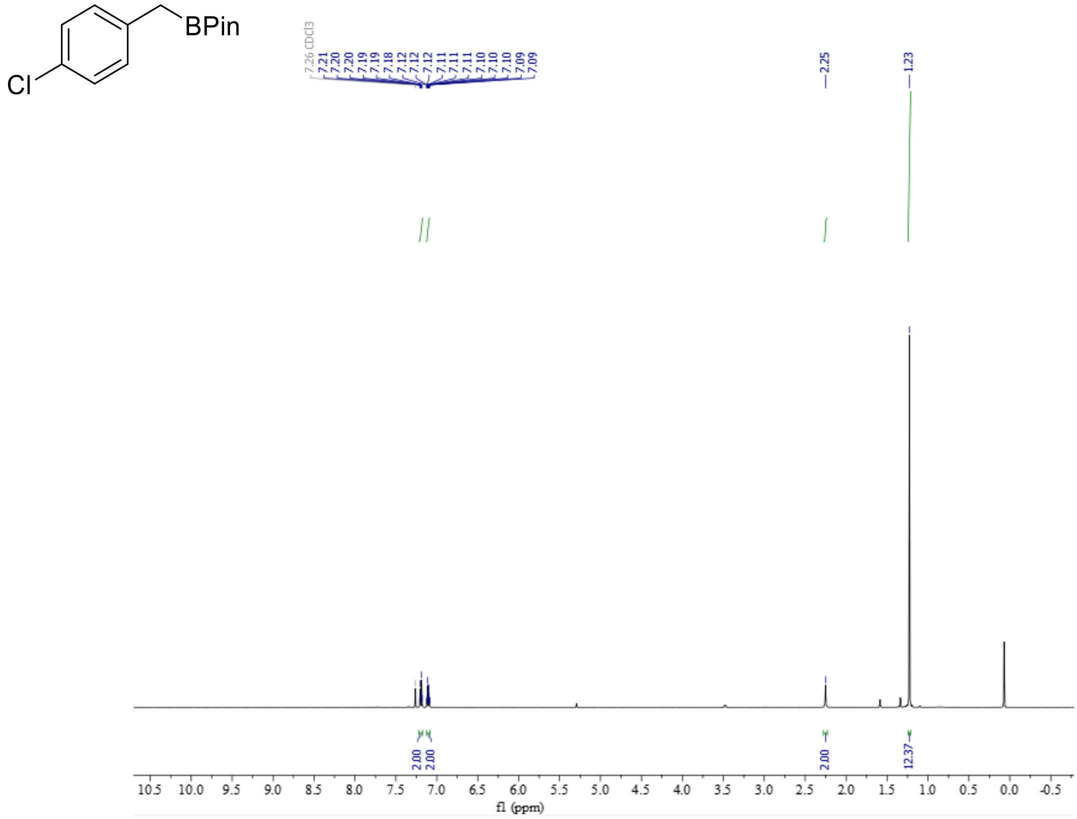

^{13}C NMR (126 MHz, $CDCl_3$)

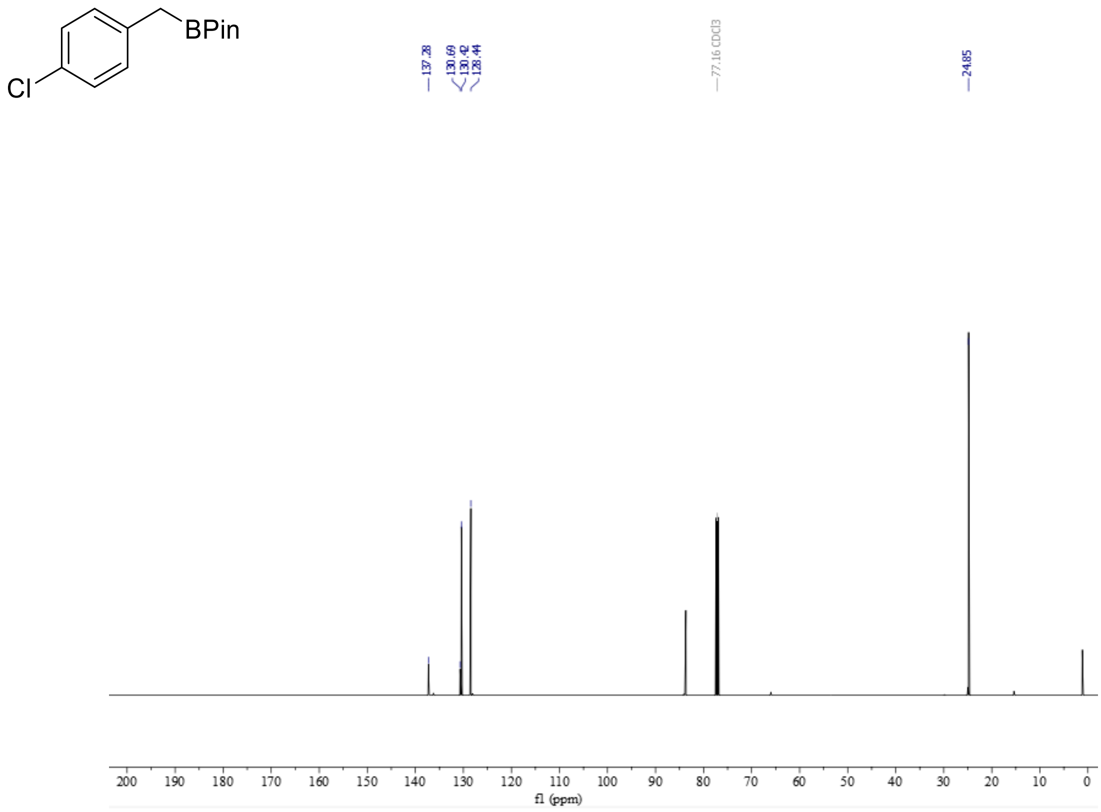

^{1}H-^{13}C-HSQC NMR – *α-boryl carbon atom highlighted*

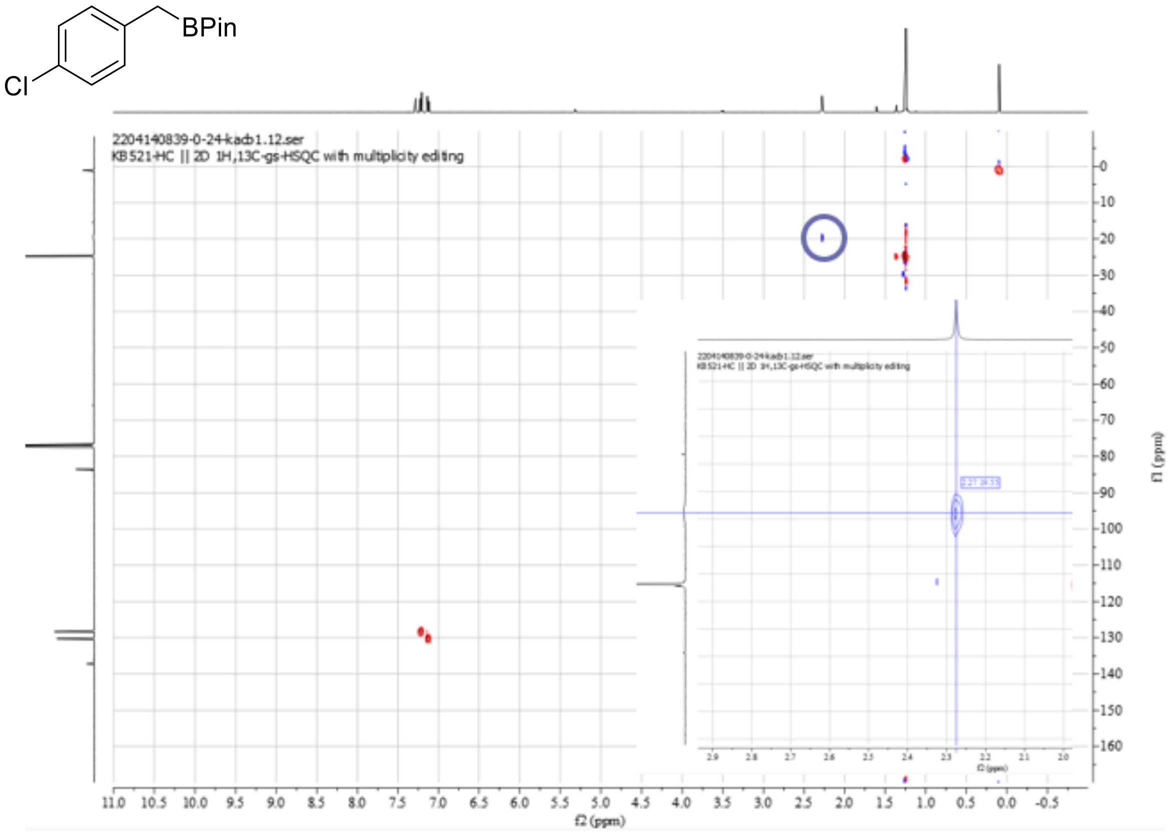

^{11}B NMR (96 MHz, $CDCl_3$)

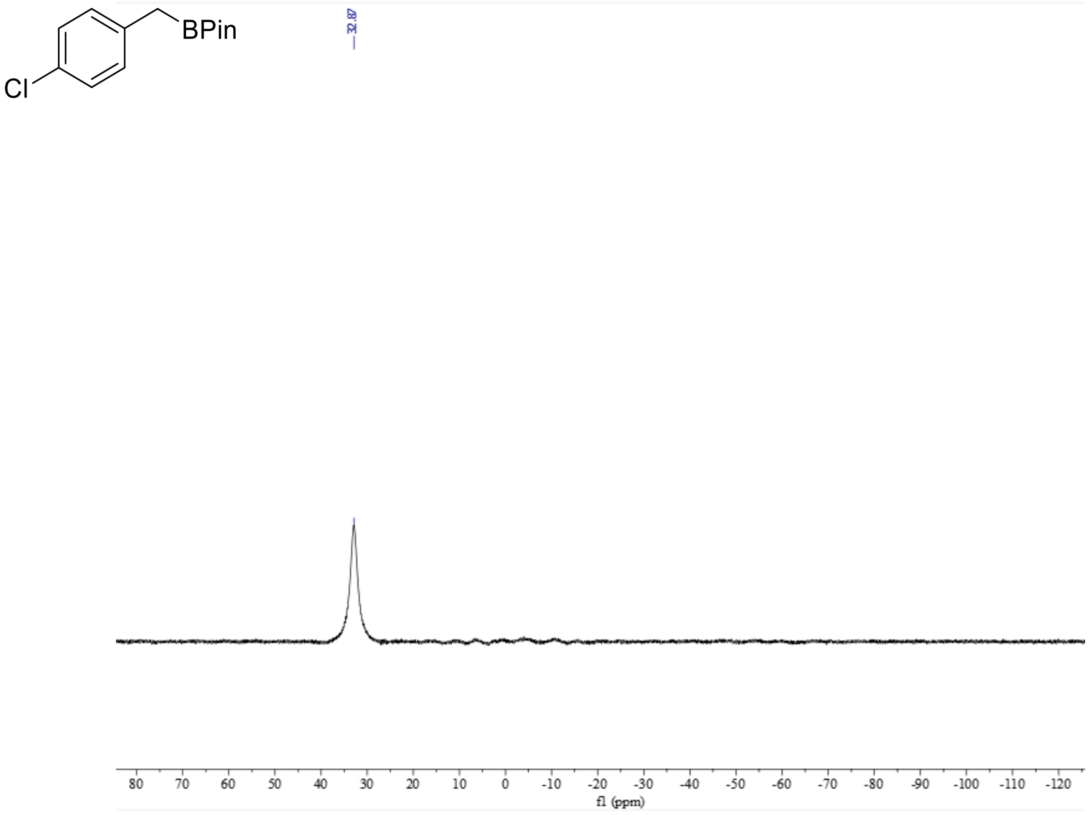

2-(2-chlorobenzyl)-4,4,5,5-tetramethyl-1,3,2-dioxaborolane (34)

^{1}H NMR (500 MHz, $CDCl_3$)

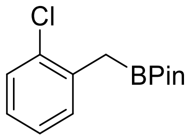

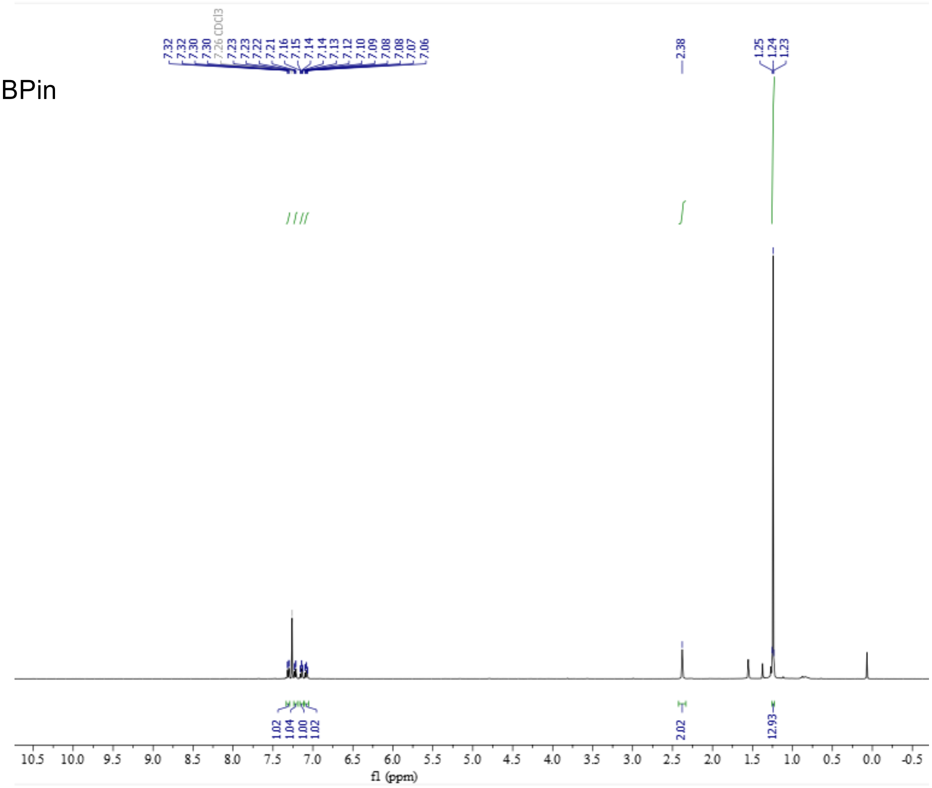

^{13}C NMR (126 MHz, $CDCl_3$)

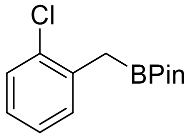

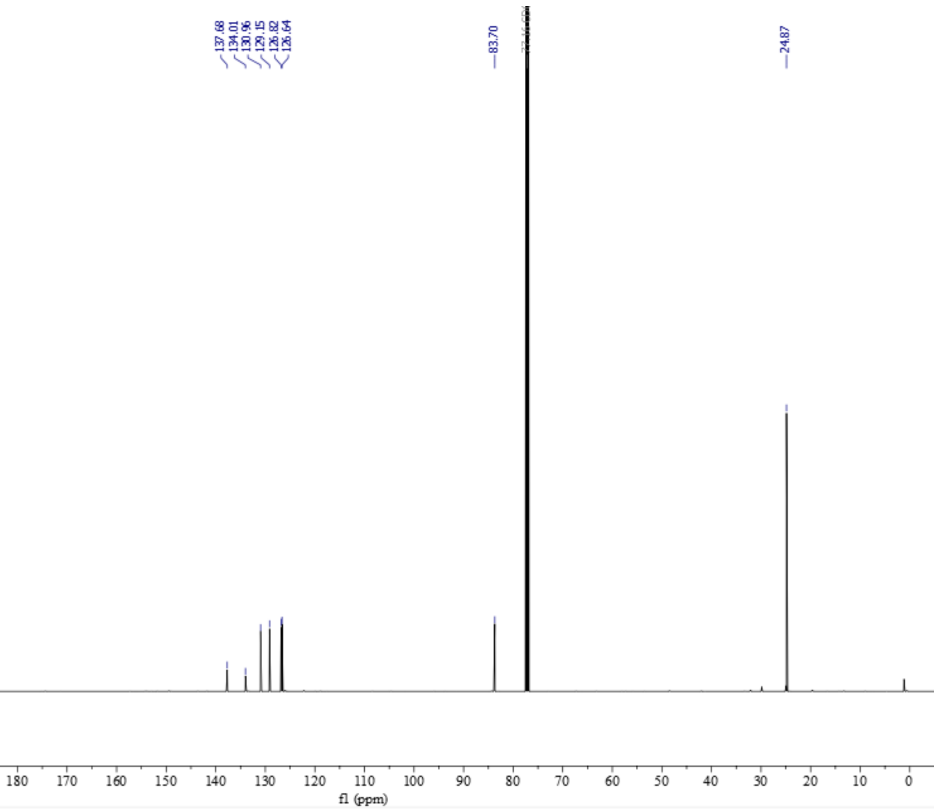

H-^{13}C-HSQC NMR – *α-boryl carbon atom highlighted*

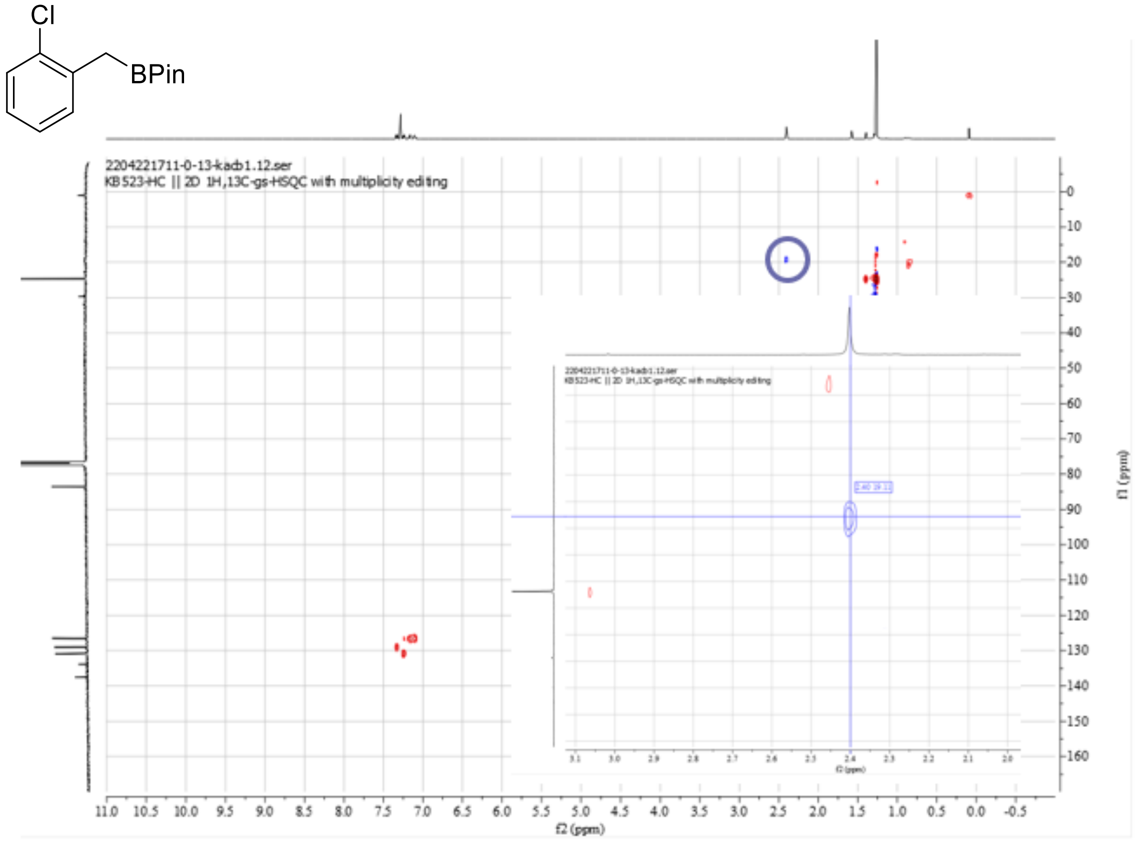

^{11}B NMR (96 MHz, $CDCl_3$)

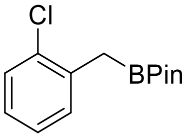

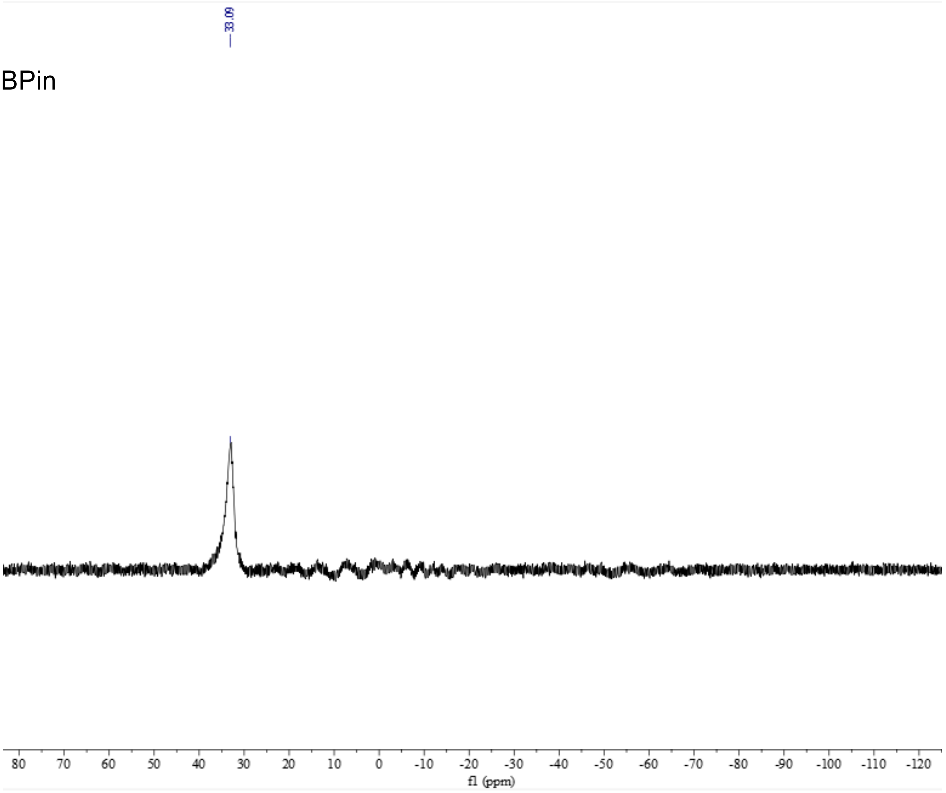

2-(3-chlorobenzyl)-4,4,5,5-tetramethyl-1,3,2-dioxaborolane (35)

^{1}H NMR (500 MHz, CDCl$_3$)

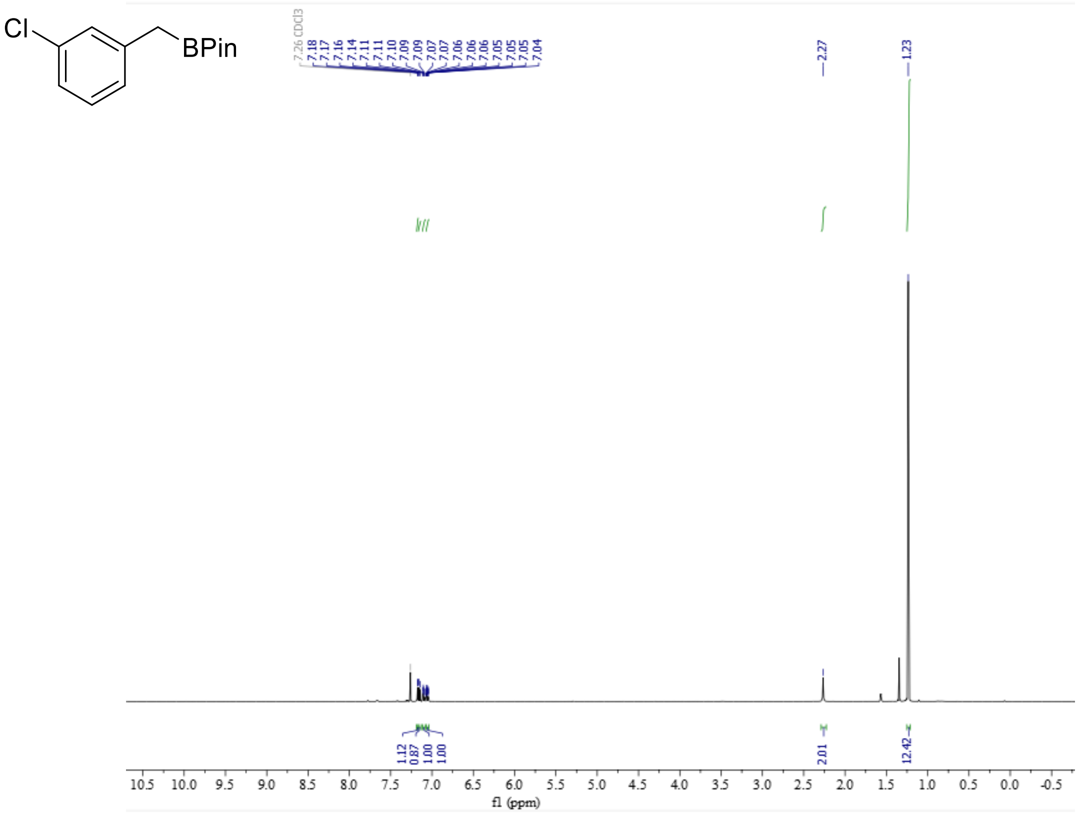

^{13}C NMR (126 MHz, CDCl$_3$)

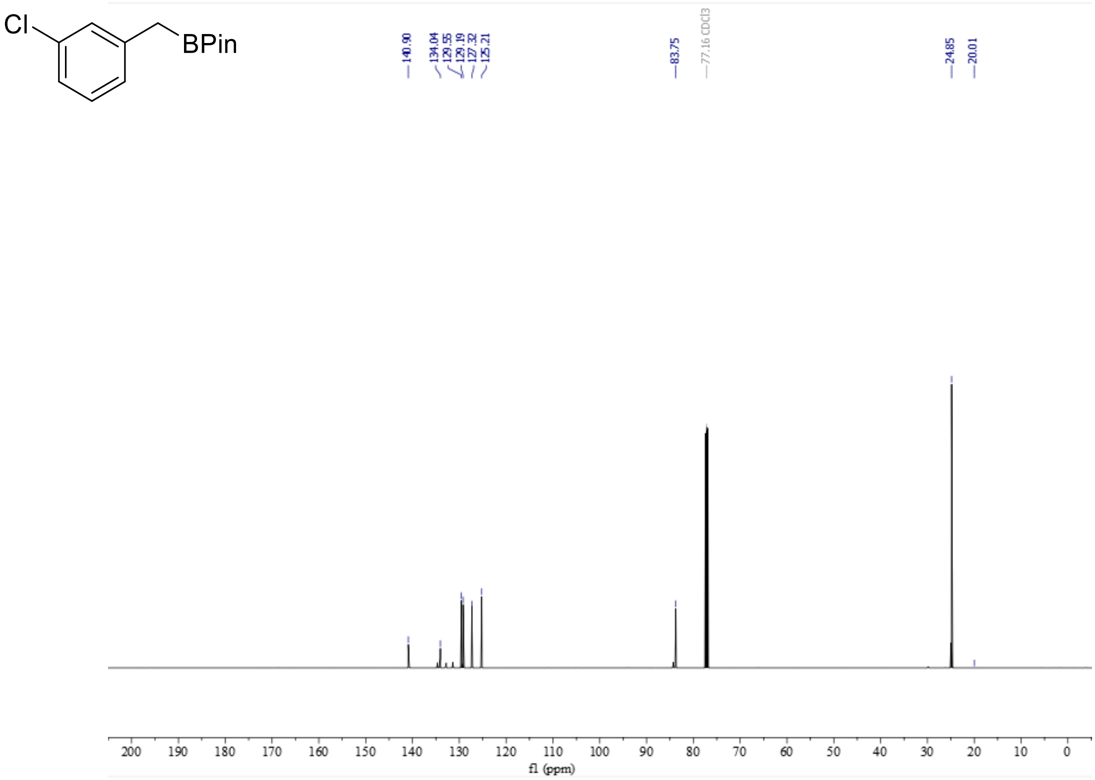

H-^{13}C-HSQC NMR – *α-boryl carbon atom highlighted*

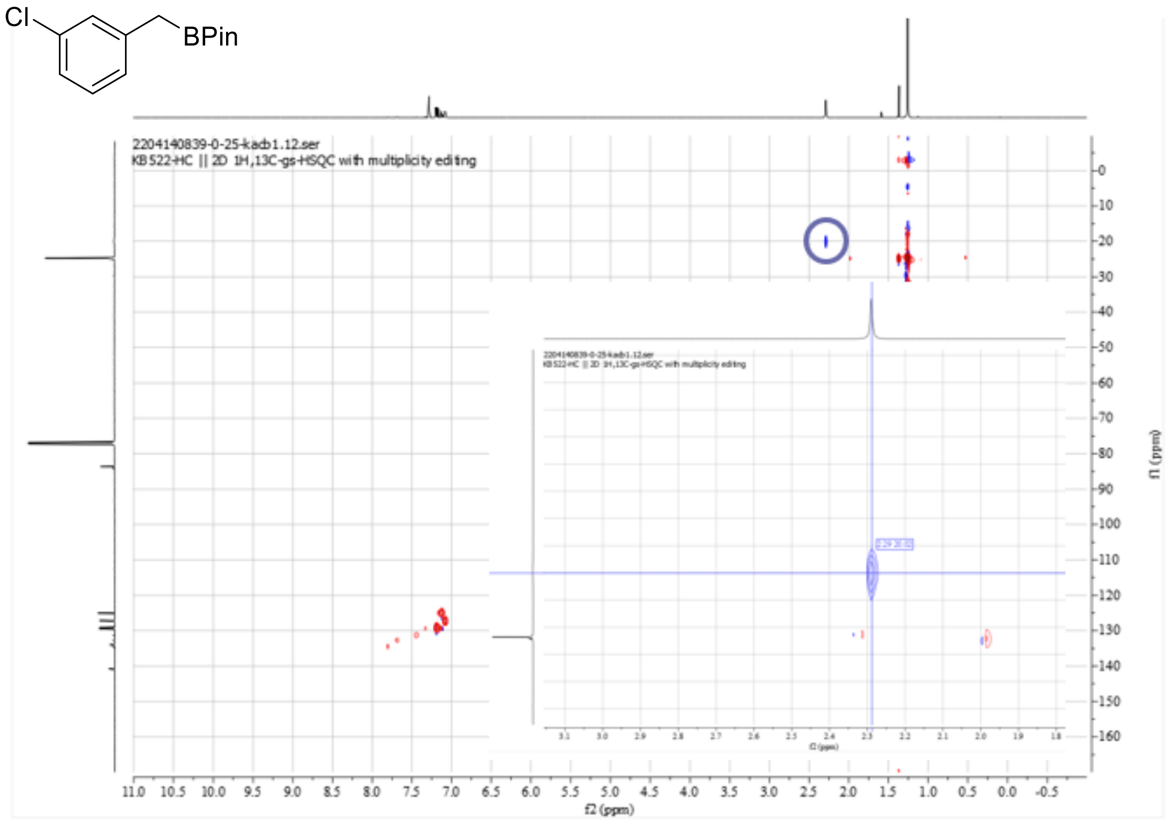

^{11}B NMR (96 MHz, $CDCl_3$)

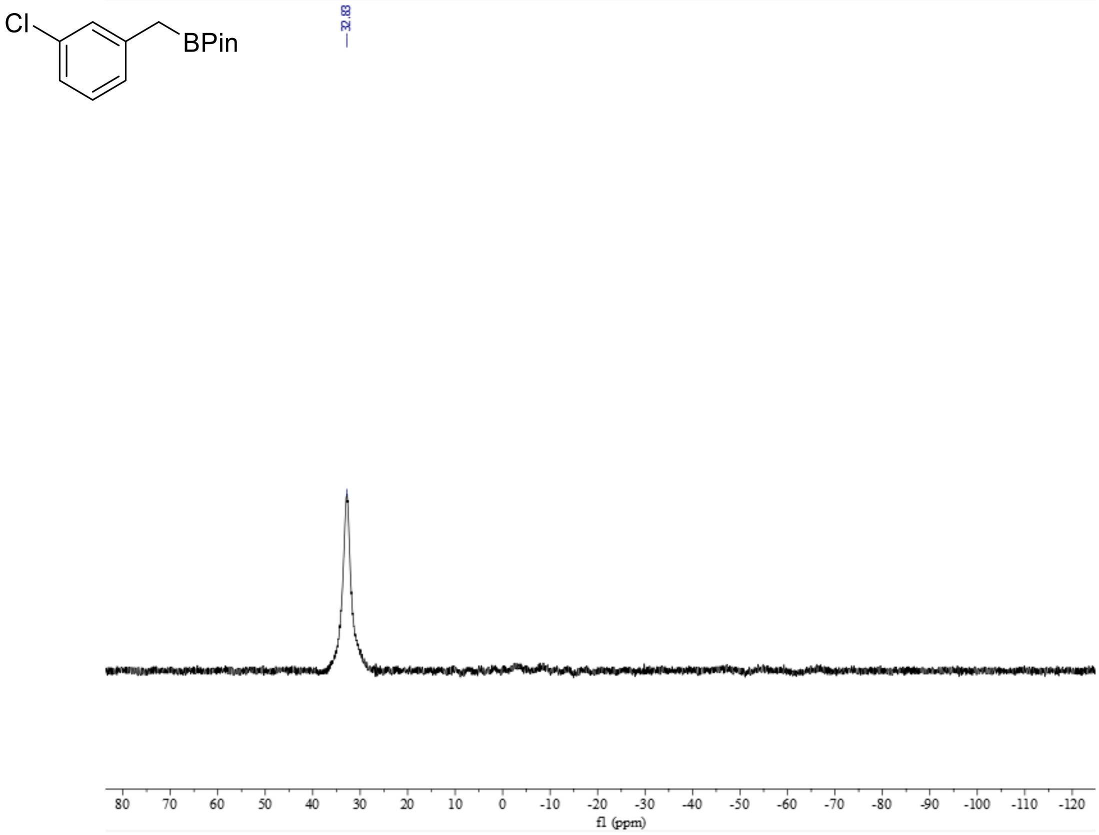

2-(2-bromo-6-methoxybenzyl)-4,4,5,5-tetramethyl-1,3,2-dioxaborolane (36)

^{1}H NMR (500 MHz, $CDCl_3$)

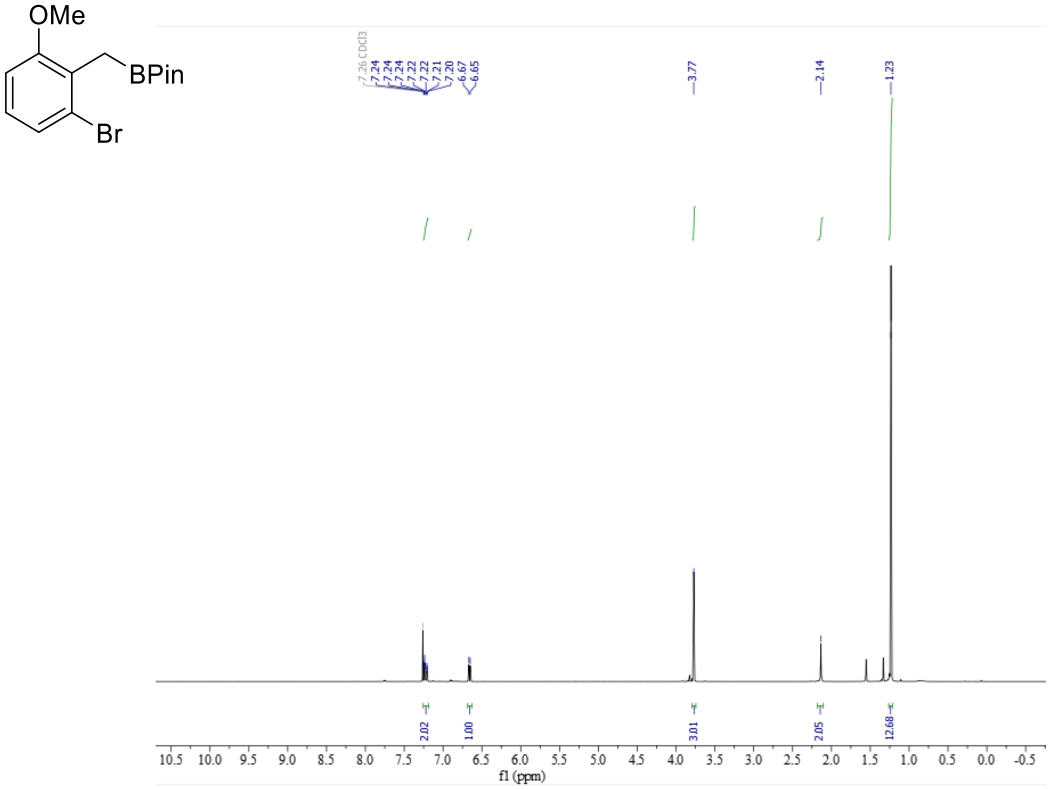

^{13}C NMR (126 MHz, $CDCl_3$)

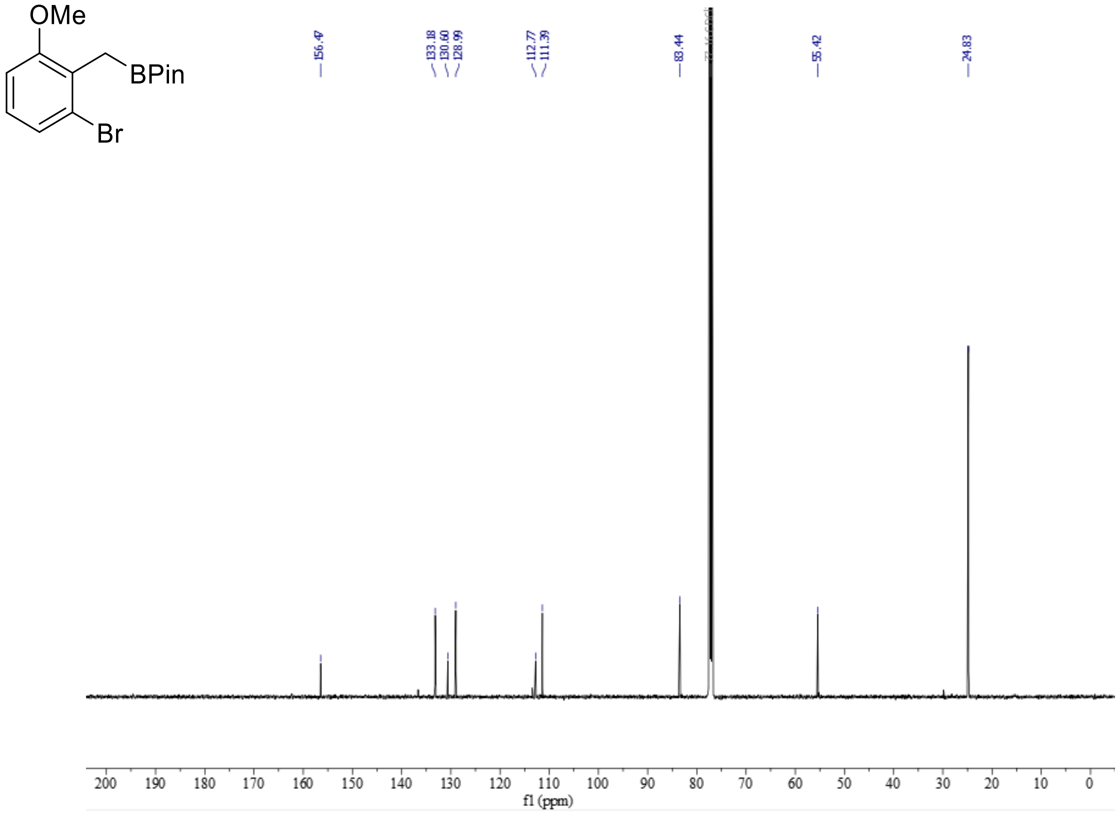

^{1}H-^{13}C-HSQC NMR – *α-boryl carbon atom highlighted*

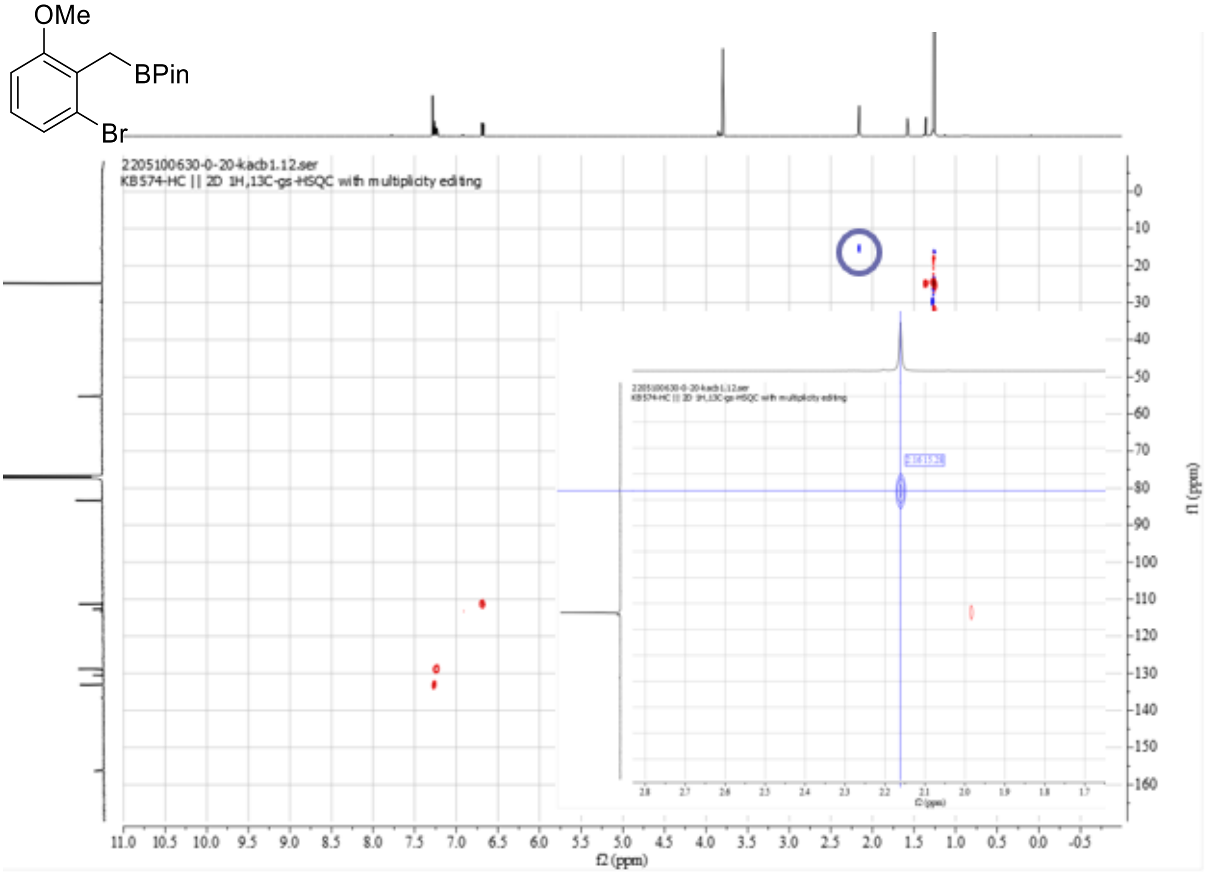

^{11}B NMR (96 MHz, $CDCl_3$)

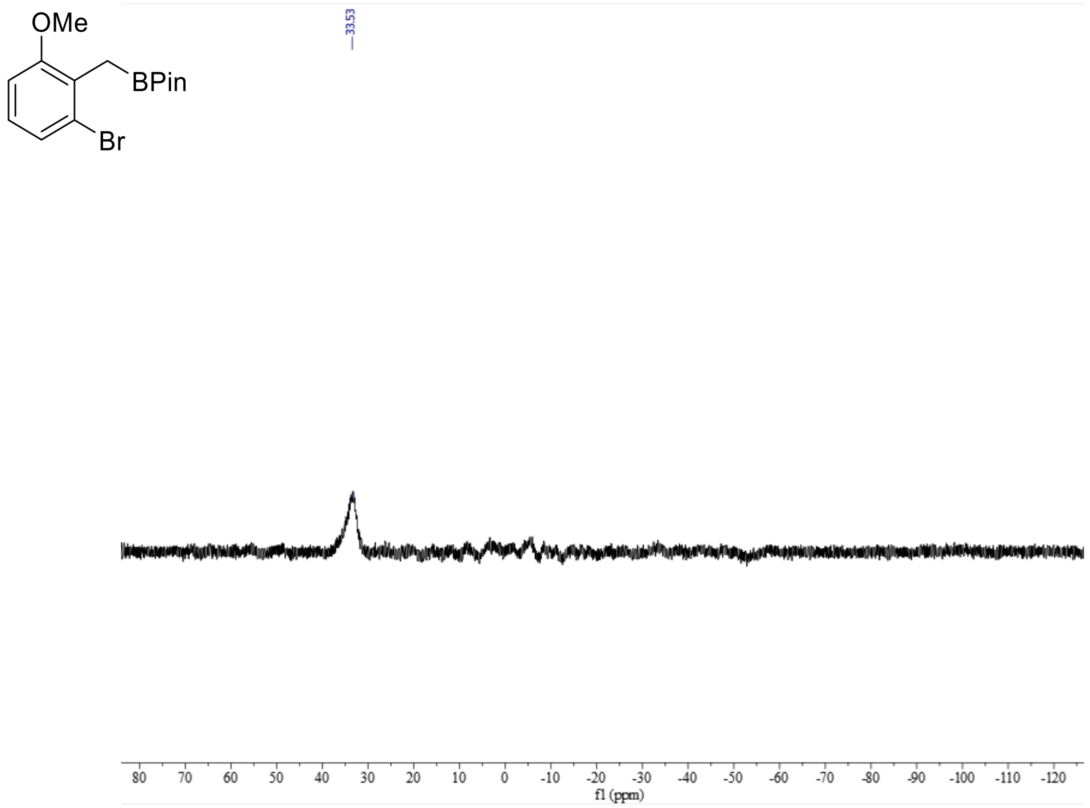

methyl 4-((4,4,5,5-tetramethyl-1,3,2-dioxaborolan-2-yl)methyl)benzoate (37)

^{1}H NMR (500 MHz, CDCl$_3$)

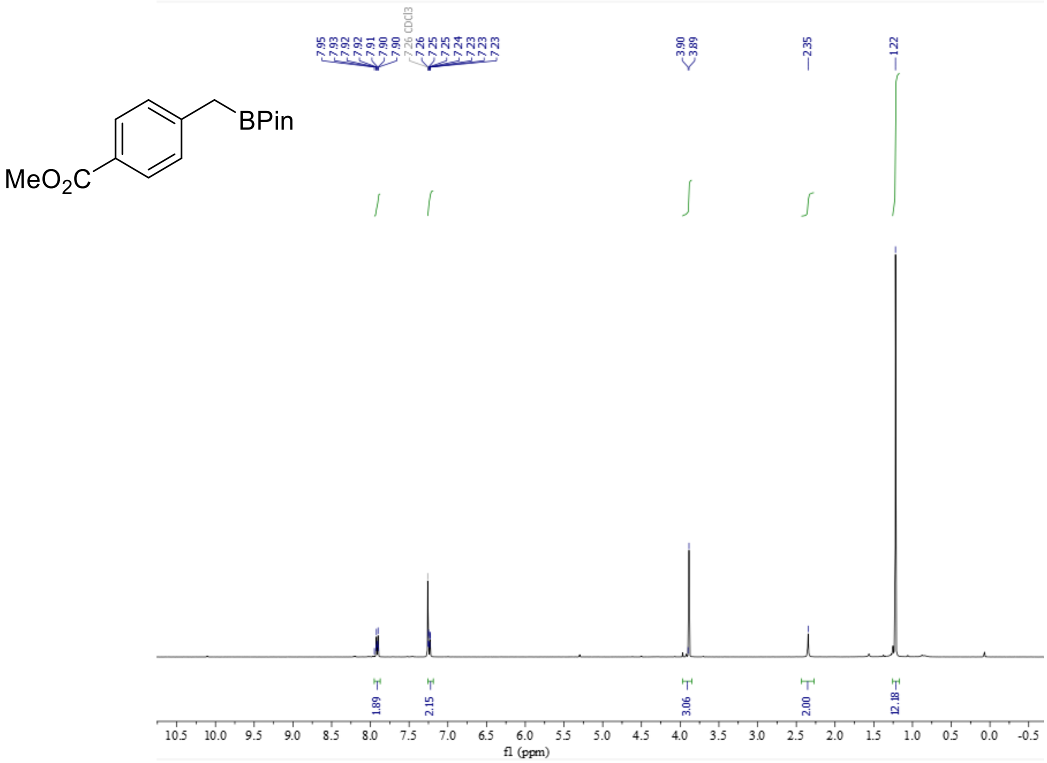

^{13}C NMR (126 MHz, CDCl$_3$)

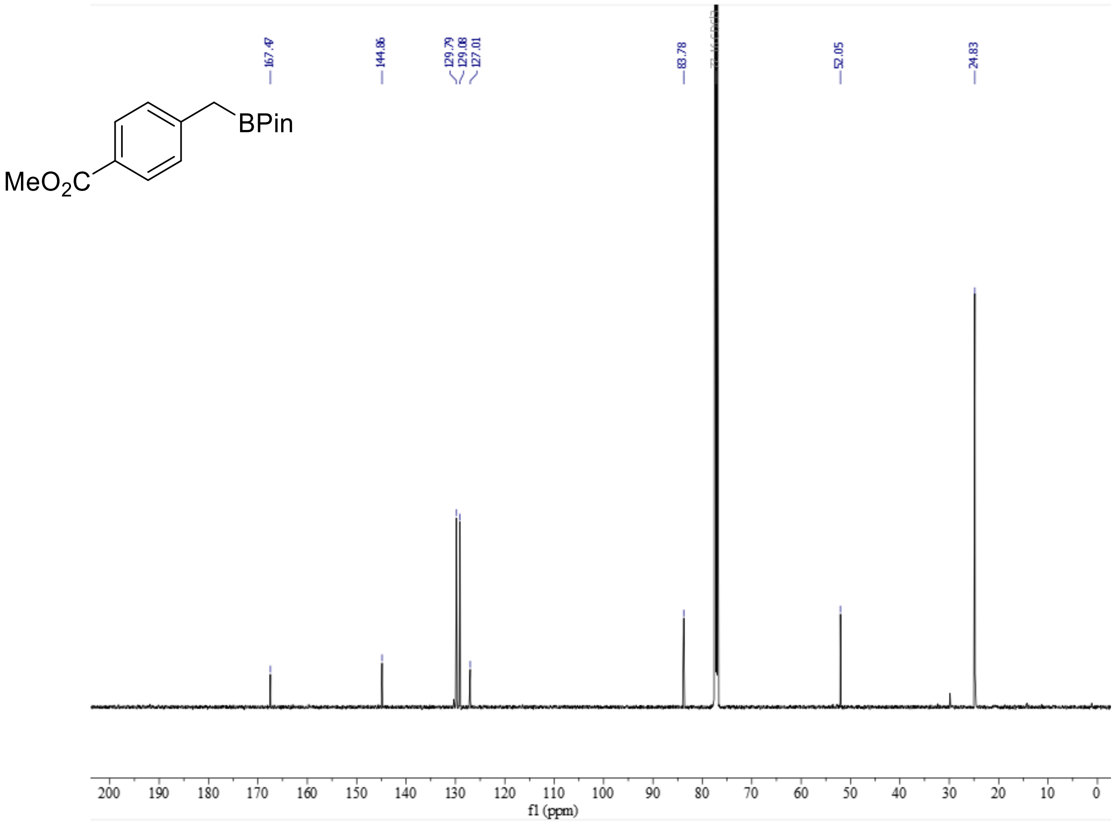

^{11}B NMR (96 MHz, $CDCl_3$)

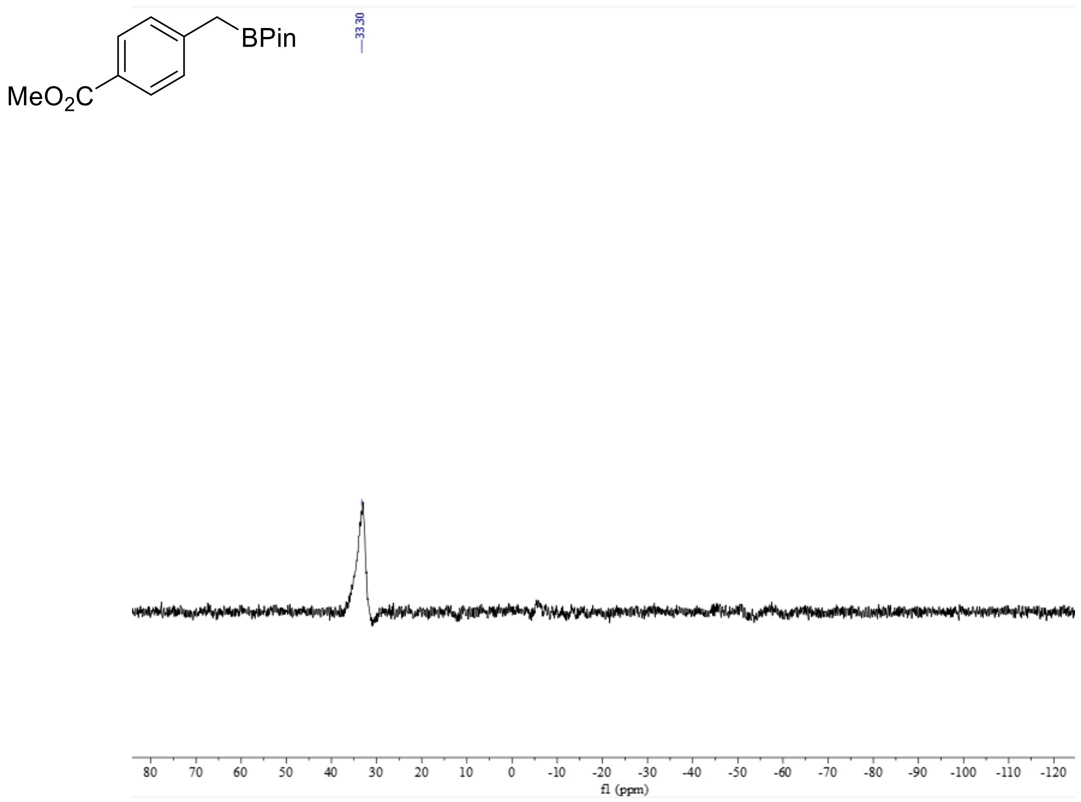

4,4,5,5-tetramethyl-2-((phenyl-d_5)methyl)-1,3,2-dioxaborolane (38)

^{1}H NMR (500 MHz, $CDCl_3$)

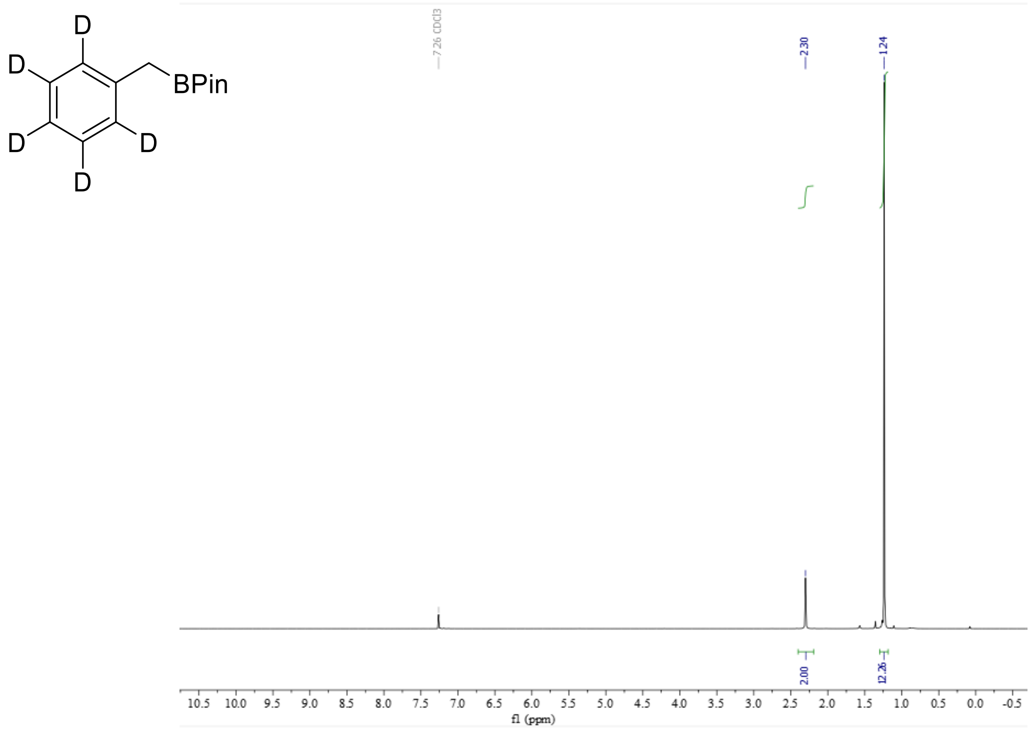

^{2}H NMR (77 MHz, $CHCl_3$)

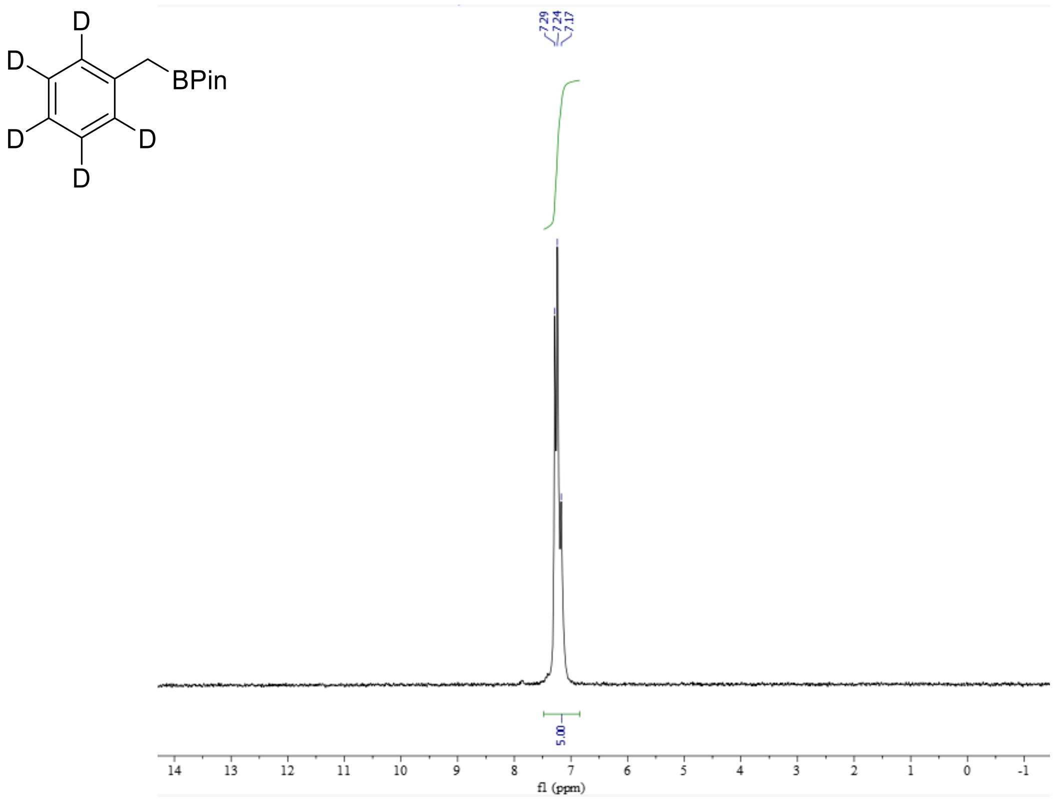

^{13}C NMR (126 MHz, $CDCl_3$)

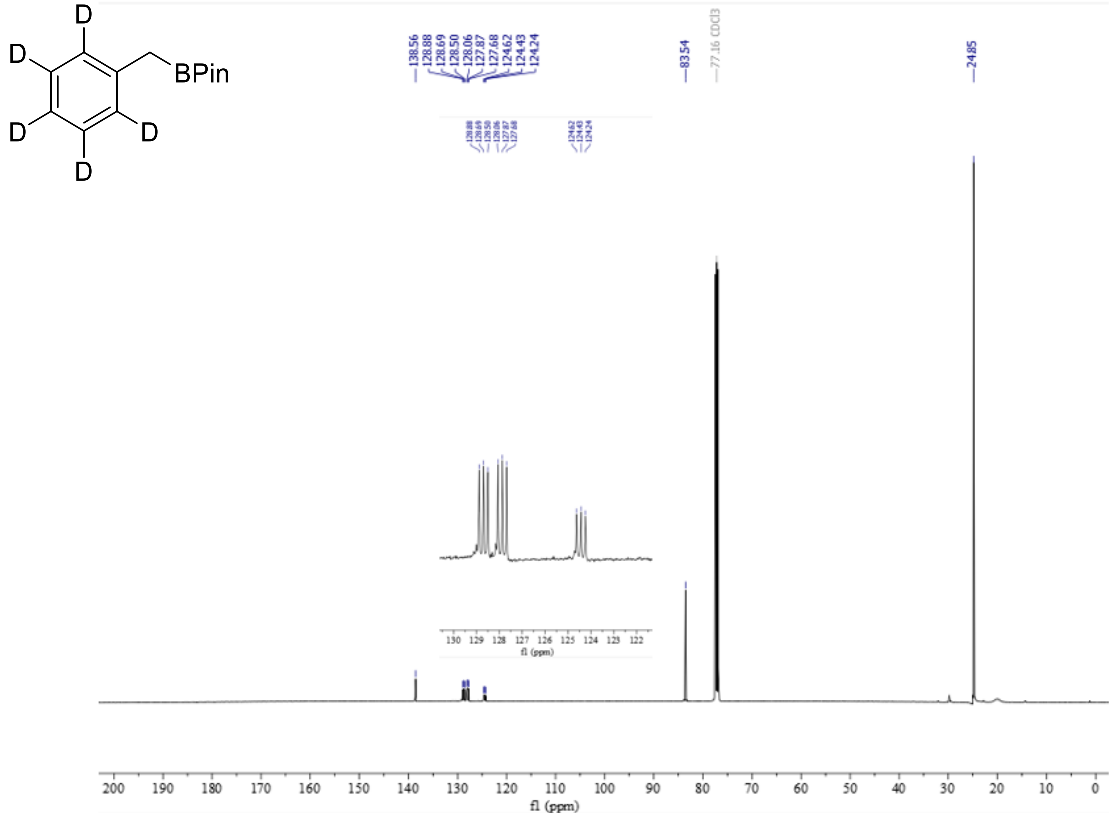

^{11}**B NMR (96 MHz, CDCl$_3$)**

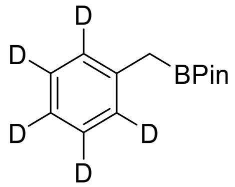

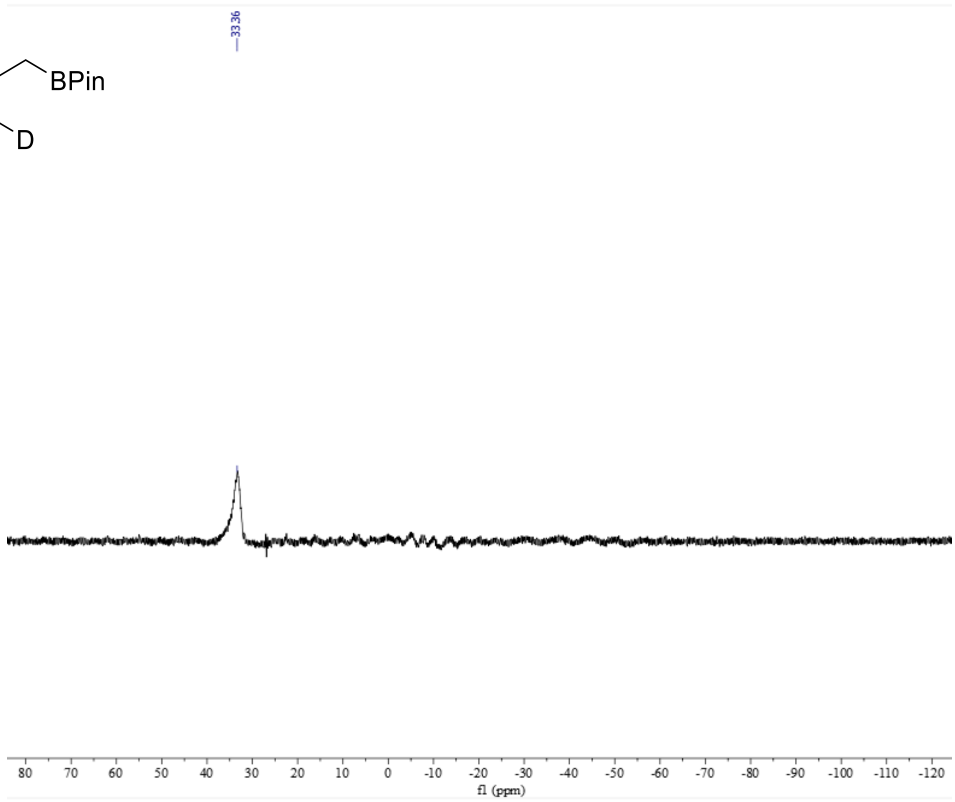

4,4,5,5-tetramethyl-2-(thiophen-3-ylmethyl)-1,3,2-dioxaborolane (39)

^{1}**H NMR (500 MHz, CDCl$_3$)**

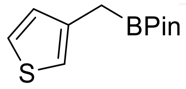

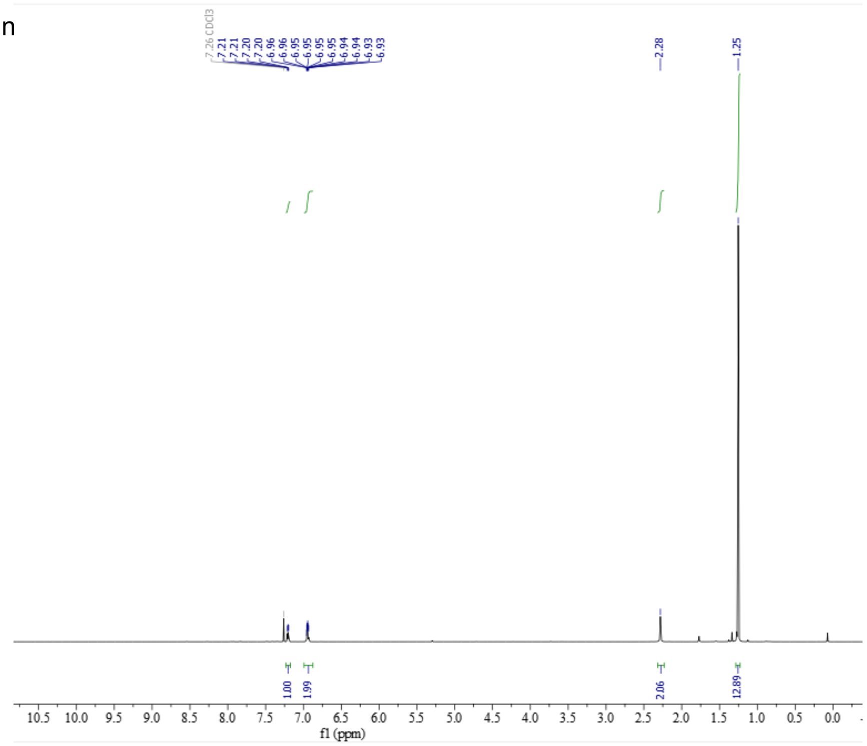

^{13}C NMR (126 MHz, CDCl$_3$)

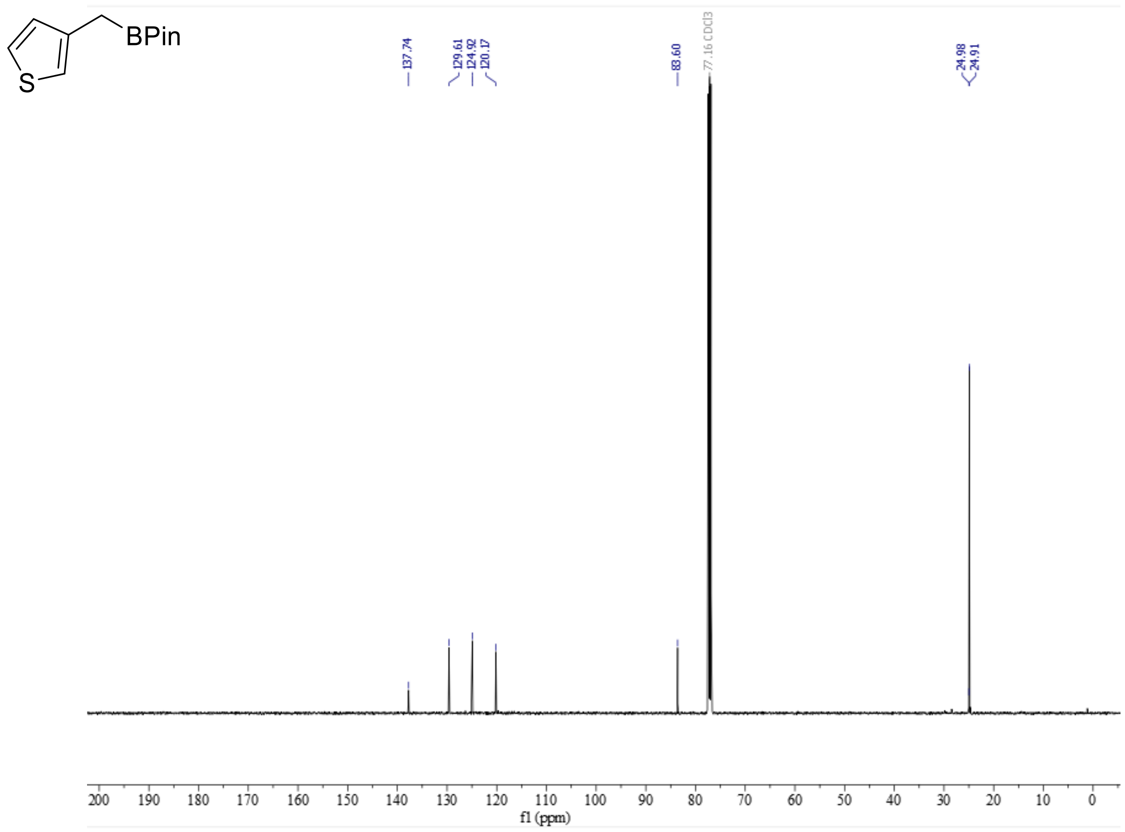

^{1}H-^{13}C-HSQC NMR – *α-boryl carbon atom highlighted*

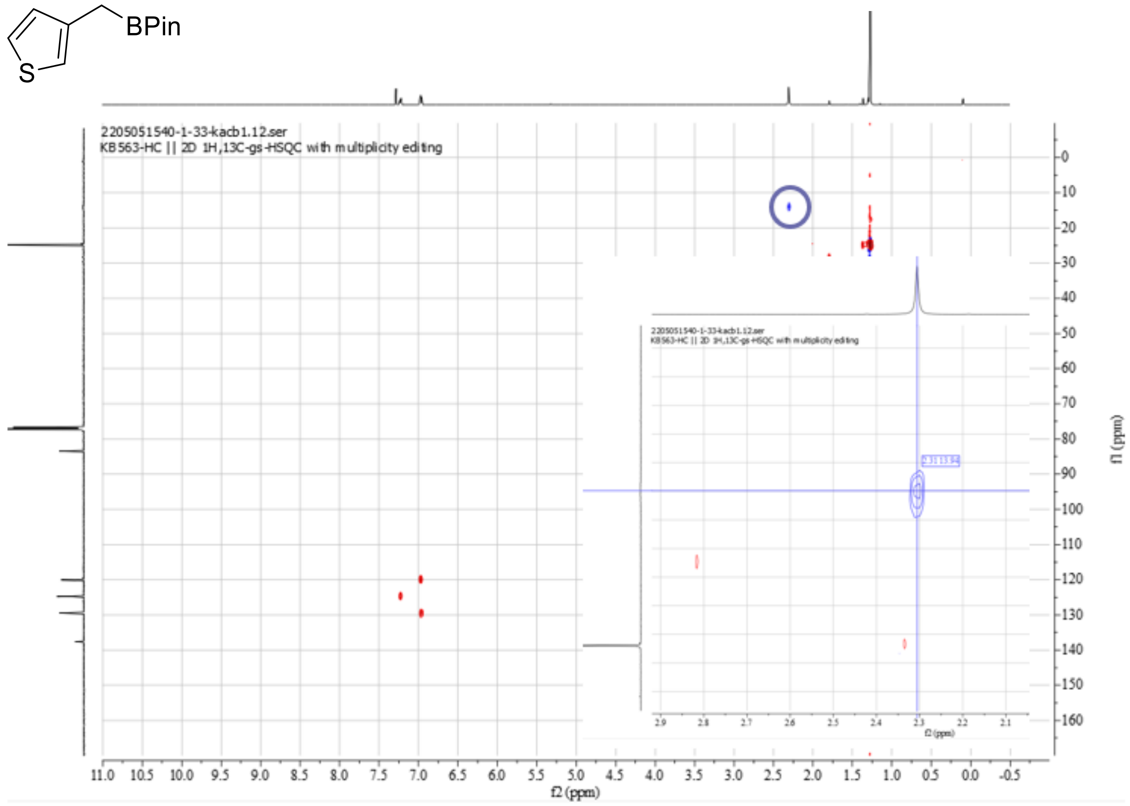

^{11}B NMR (96 MHz, CDCl$_3$)

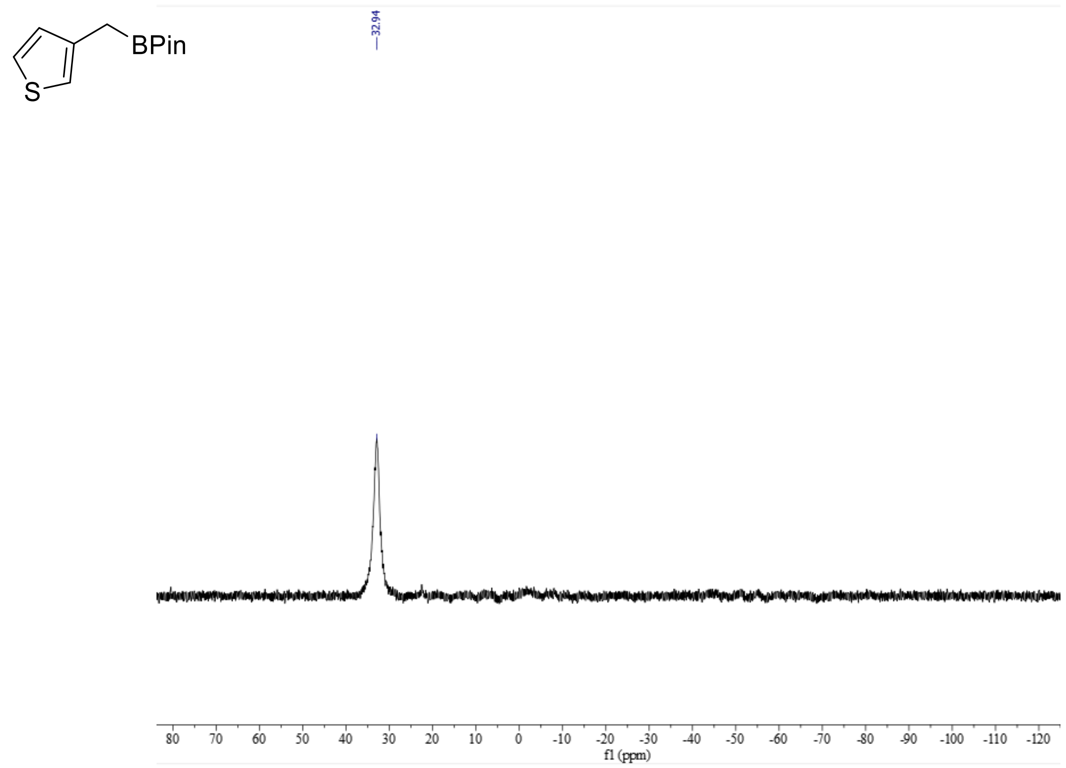

4,4,5,5-tetramethyl-2-((3-methylthiophen-2-yl)methyl)-1,3,2-dioxaborolane (40)

^{1}H NMR (500 MHz, CDCl$_3$)

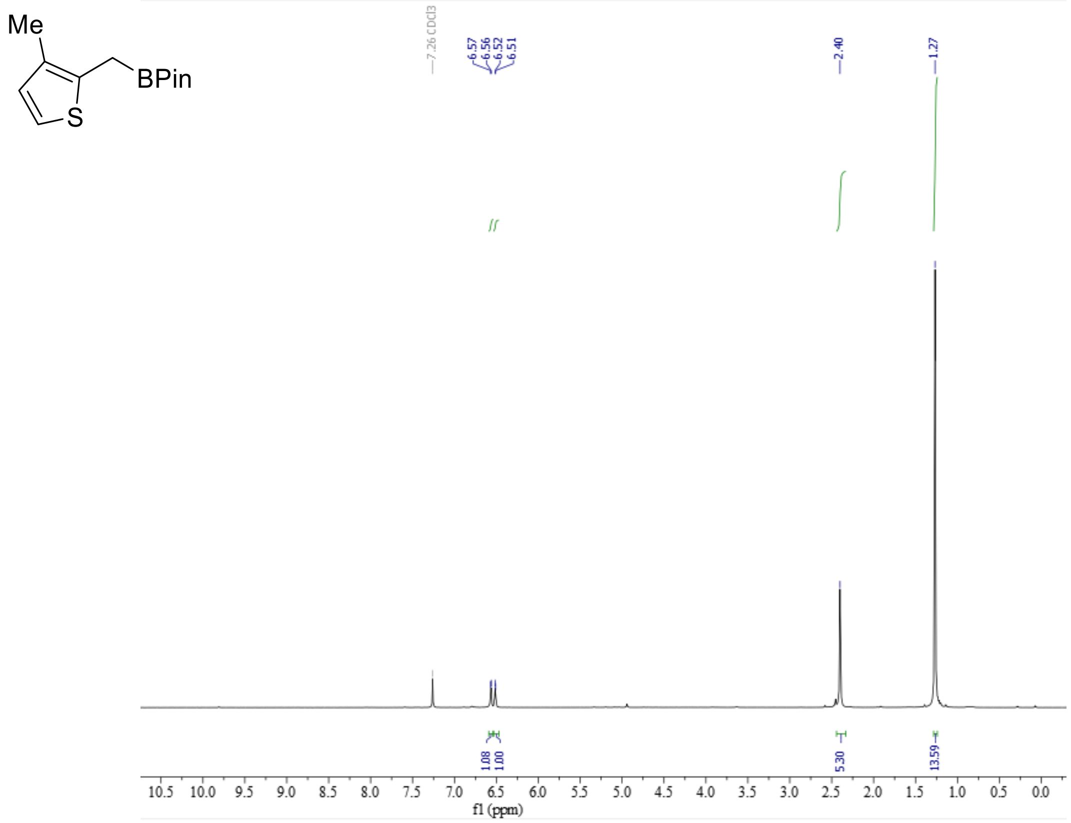

^{13}C NMR (126 MHz, $CDCl_3$)

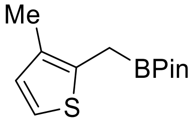

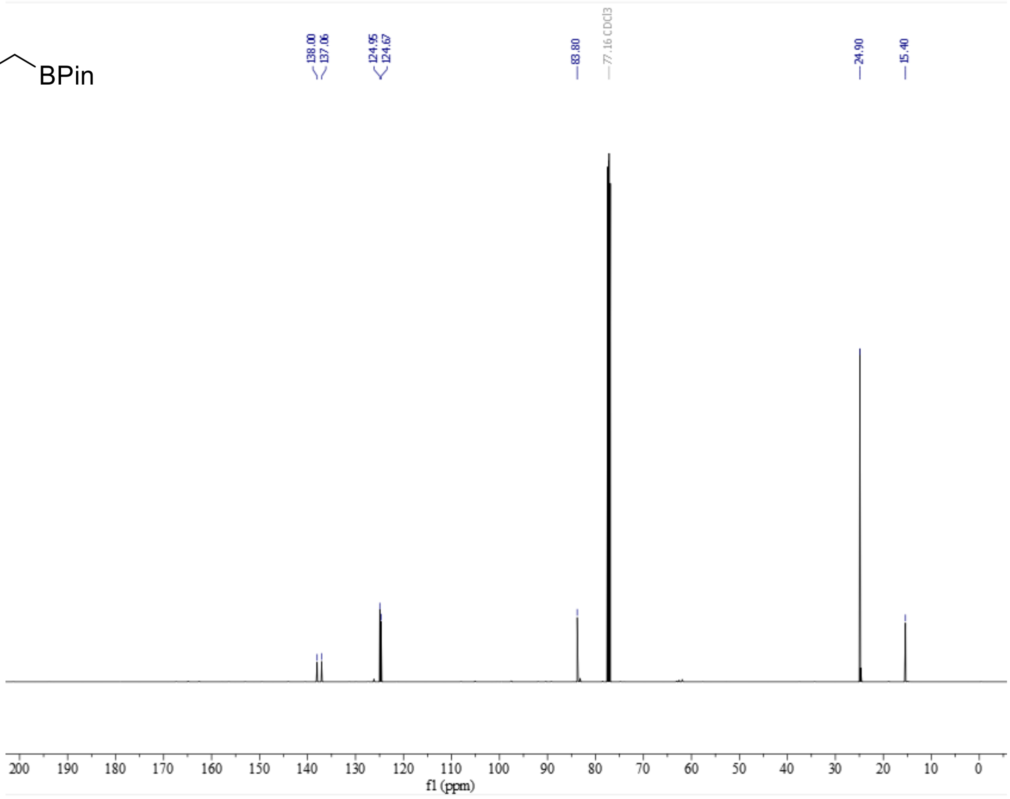

^{11}B NMR (96 MHz, $CDCl_3$)

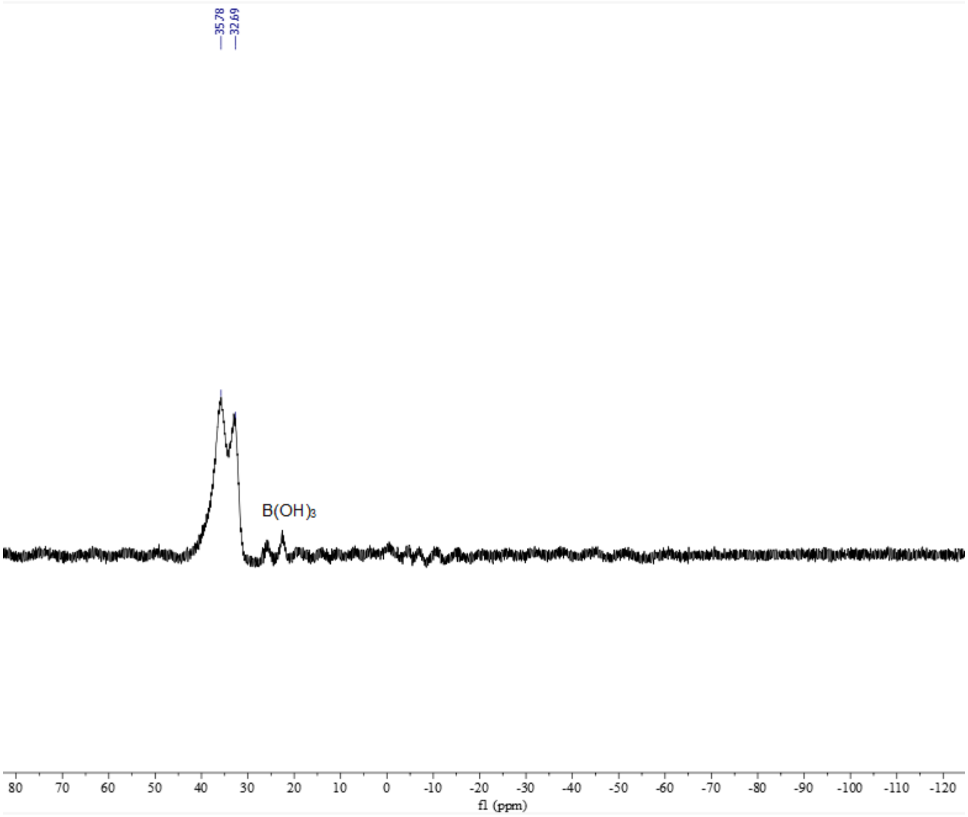

2-(benzo[b]thiophen-3-ylmethyl)-4,4,5,5-tetramethyl-1,3,2-dioxaborolane (41)

^{1}H NMR (500 MHz, $CDCl_3$)

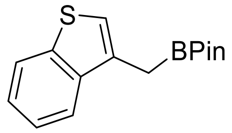

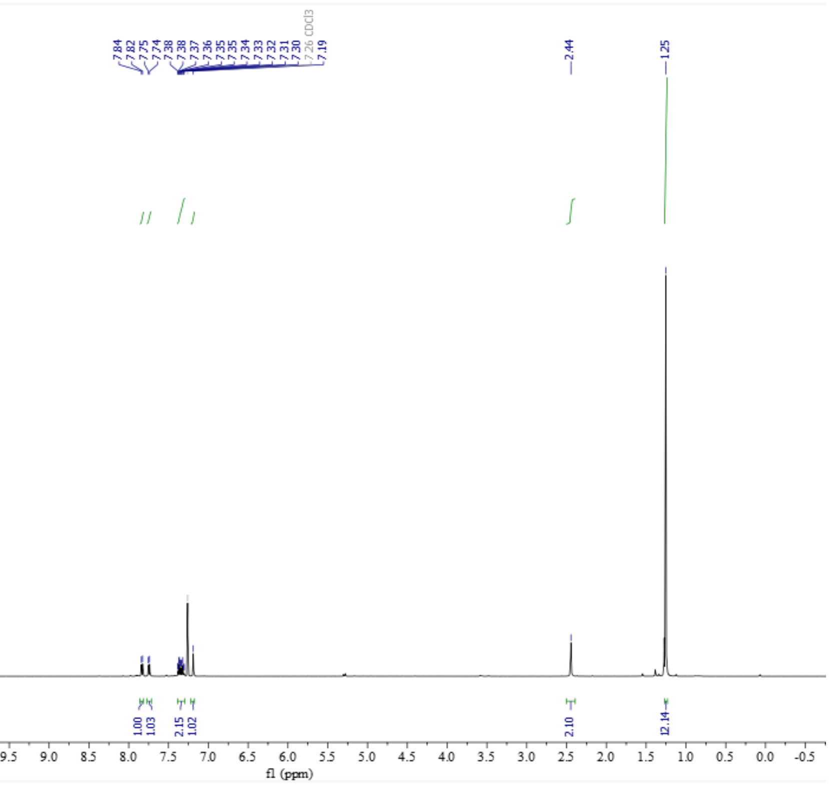

^{13}C NMR (126 MHz, $CDCl_3$)

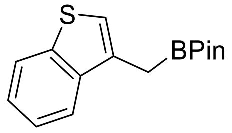

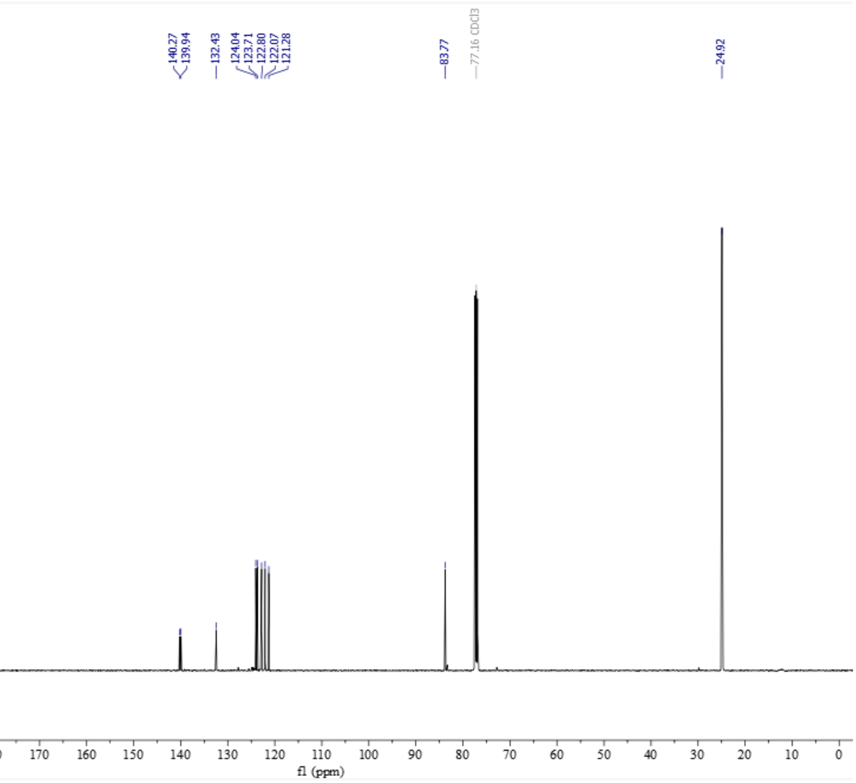

^{1}H-^{13}C-HSQC NMR – *α-boryl carbon atom highlighted*

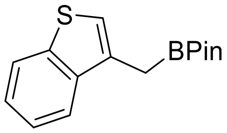

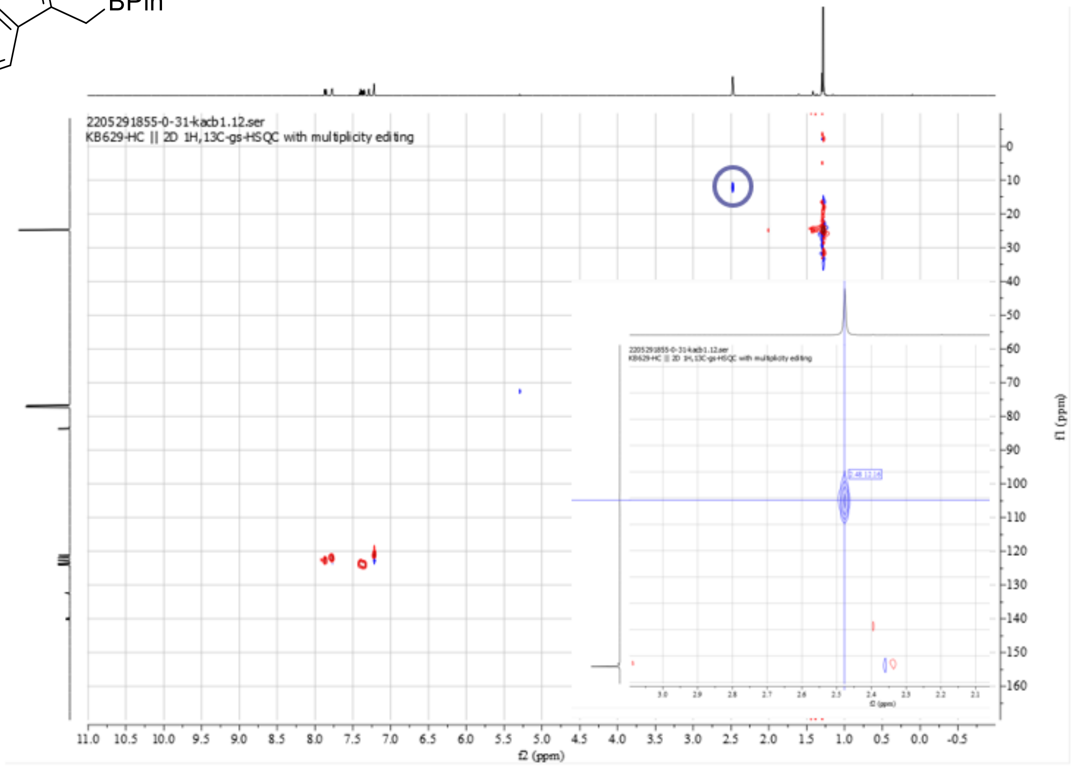

^{11}B NMR (96 MHz, $CDCl_3$)

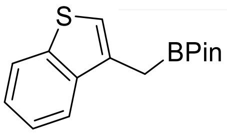

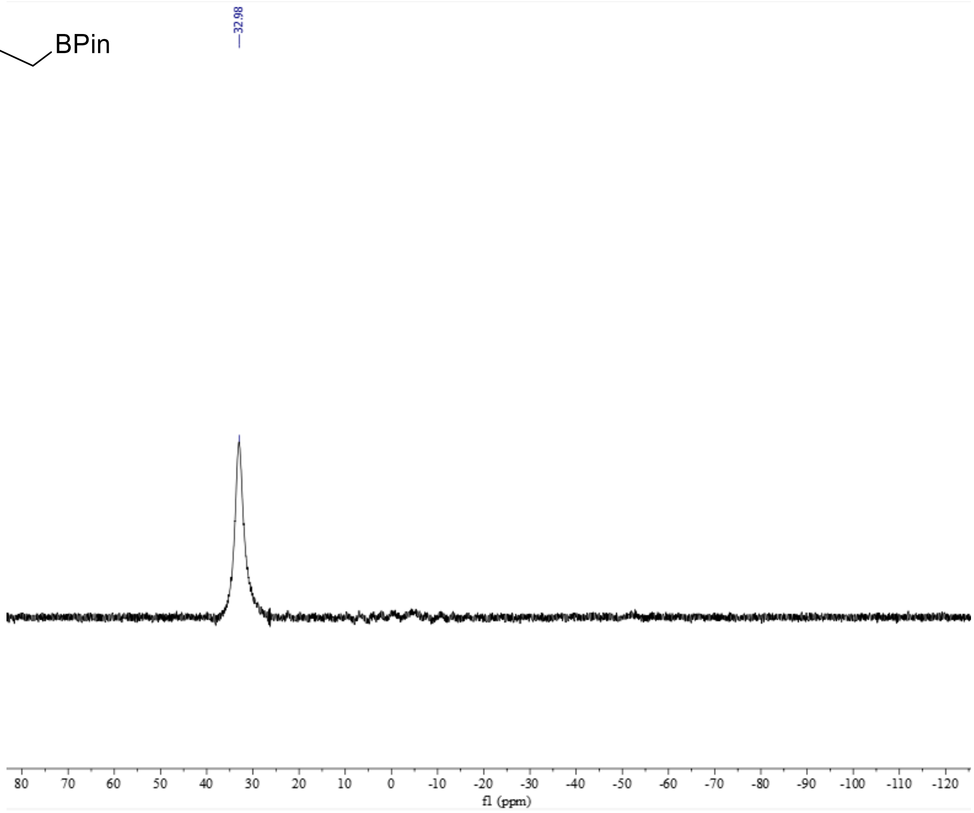

2-methoxy-3-((4,4,5,5-tetramethyl-1,3,2-dioxaborolan-2-yl)methyl)pyridine (42)

^{1}H NMR (500 MHz, $CDCl_3$)

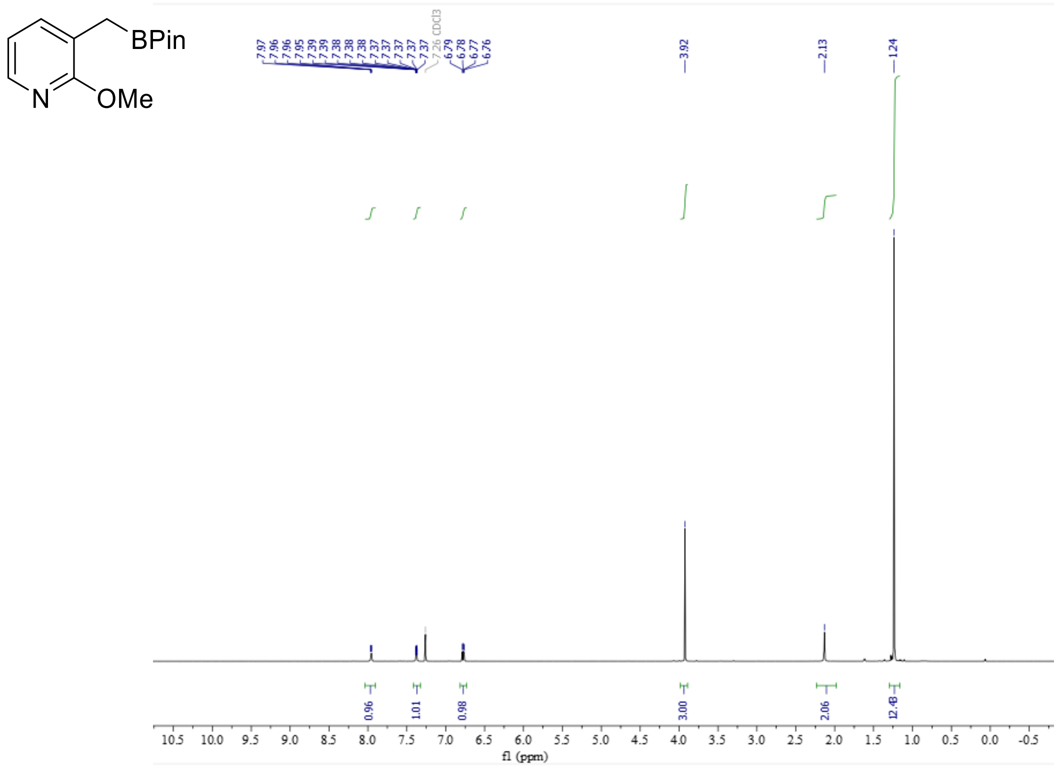

^{13}C NMR (126 MHz, $CDCl_3$)

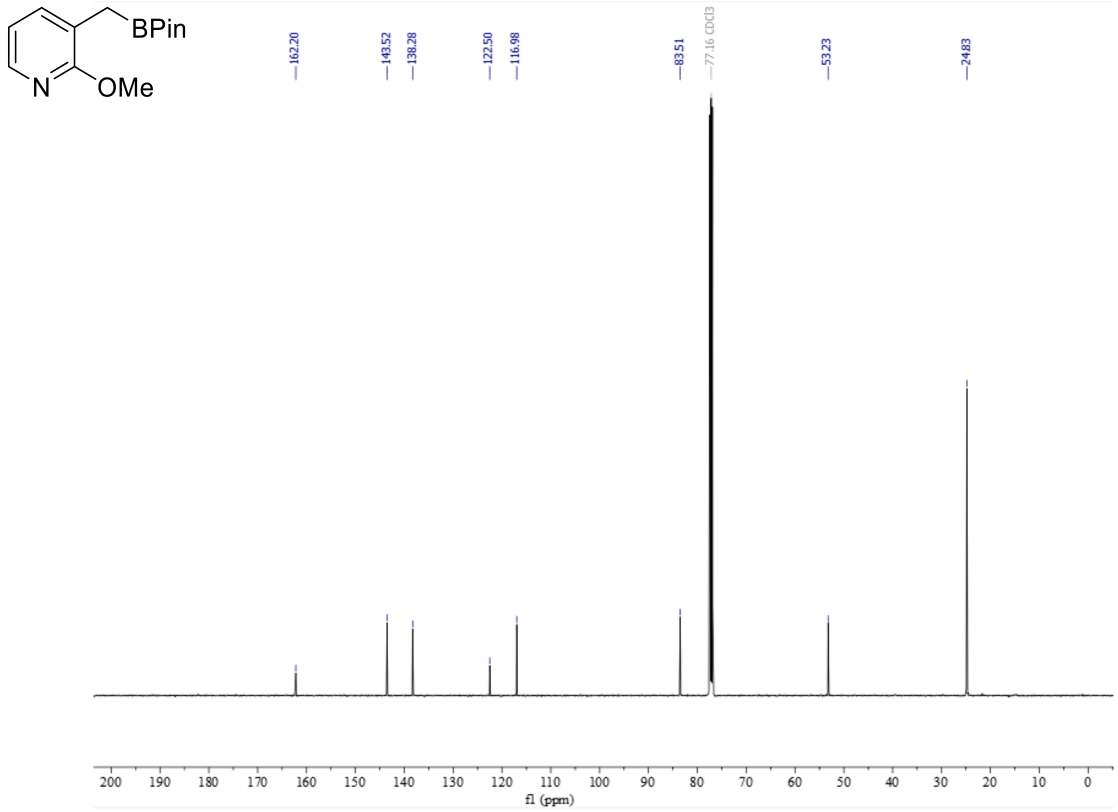

H-^{13}C-HSQC NMR – *α-boryl carbon atom highlighted*

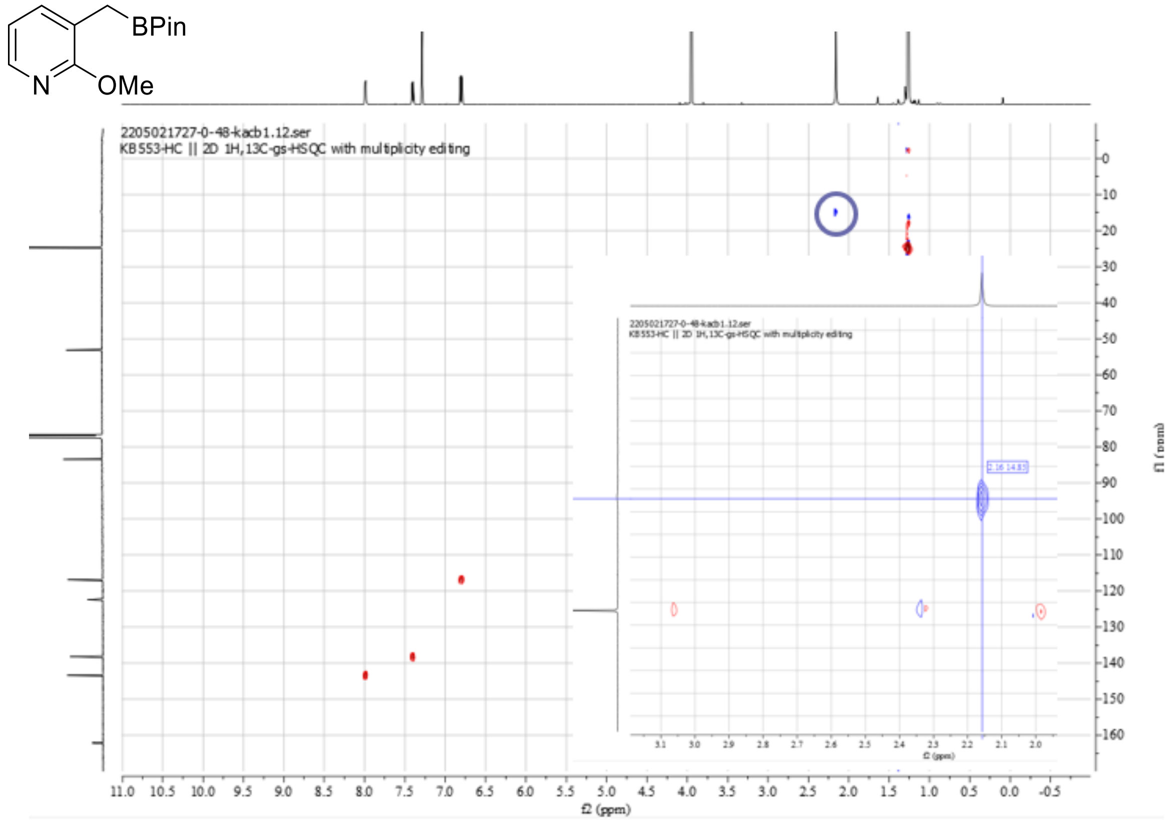

^{11}B NMR (96 MHz, $CDCl_3$)

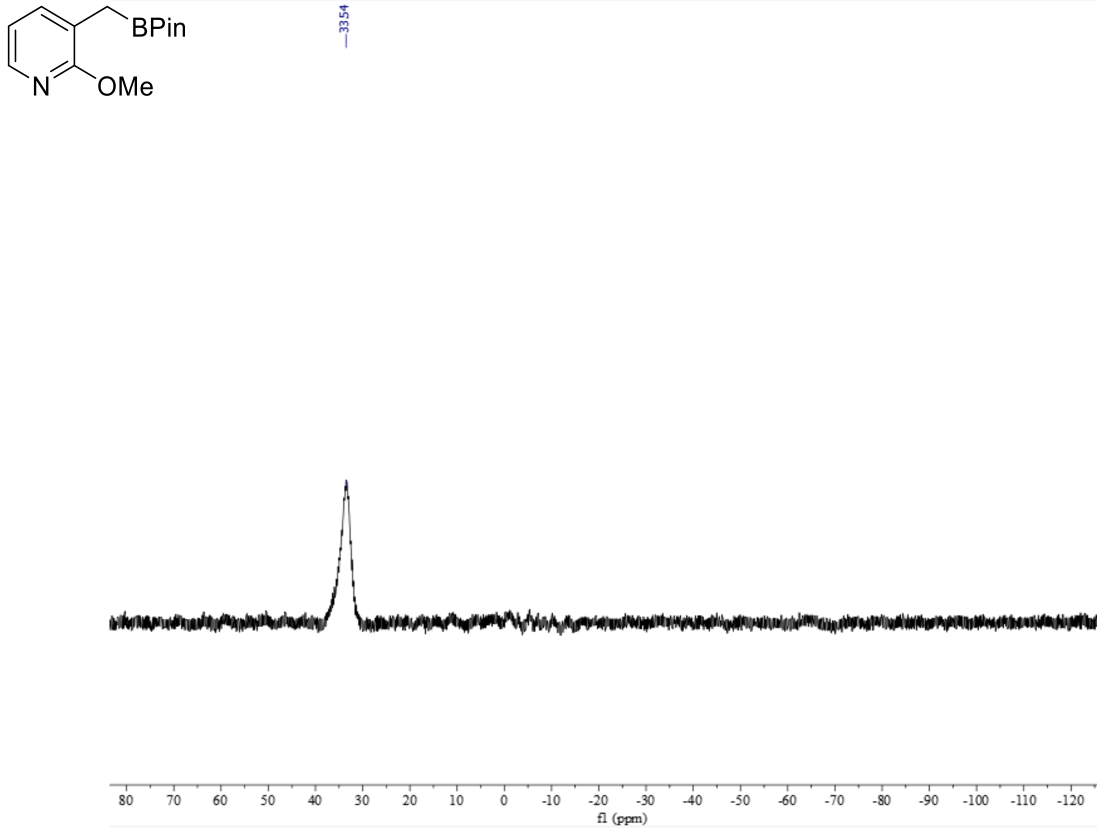

4,4,5,5-tetramethyl-2-((3-methylfuran-2-yl)methyl)-1,3,2-dioxaborolane (43)

^{1}H NMR (500 MHz, $CDCl_3$)

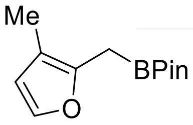

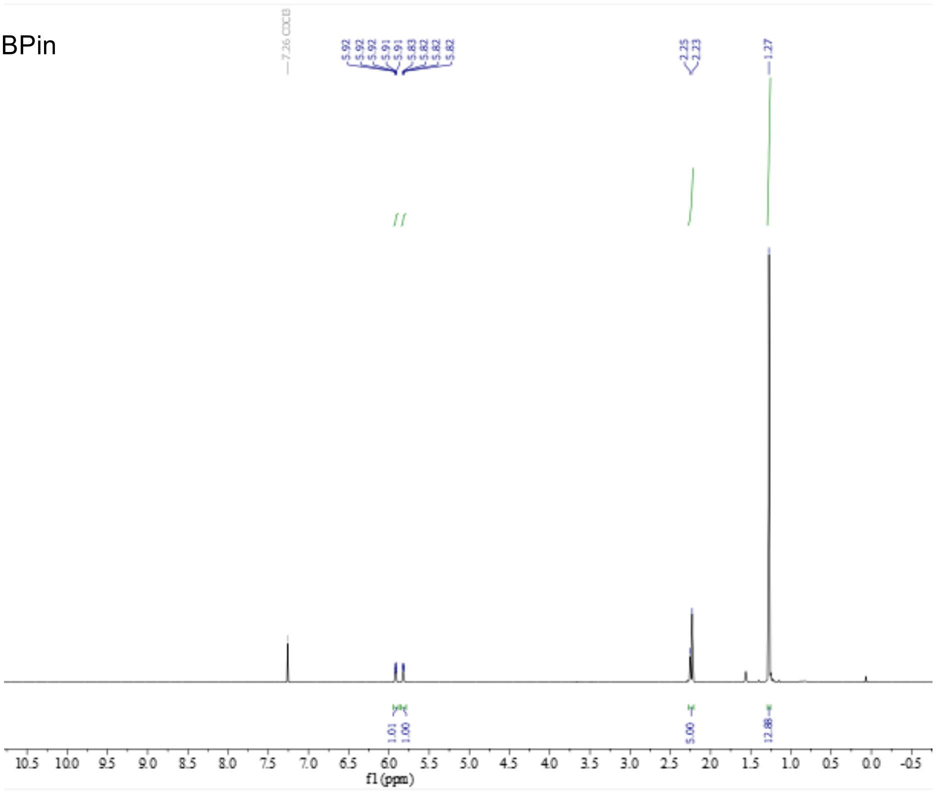

^{13}C NMR (126 MHz, $CDCl_3$)

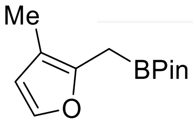

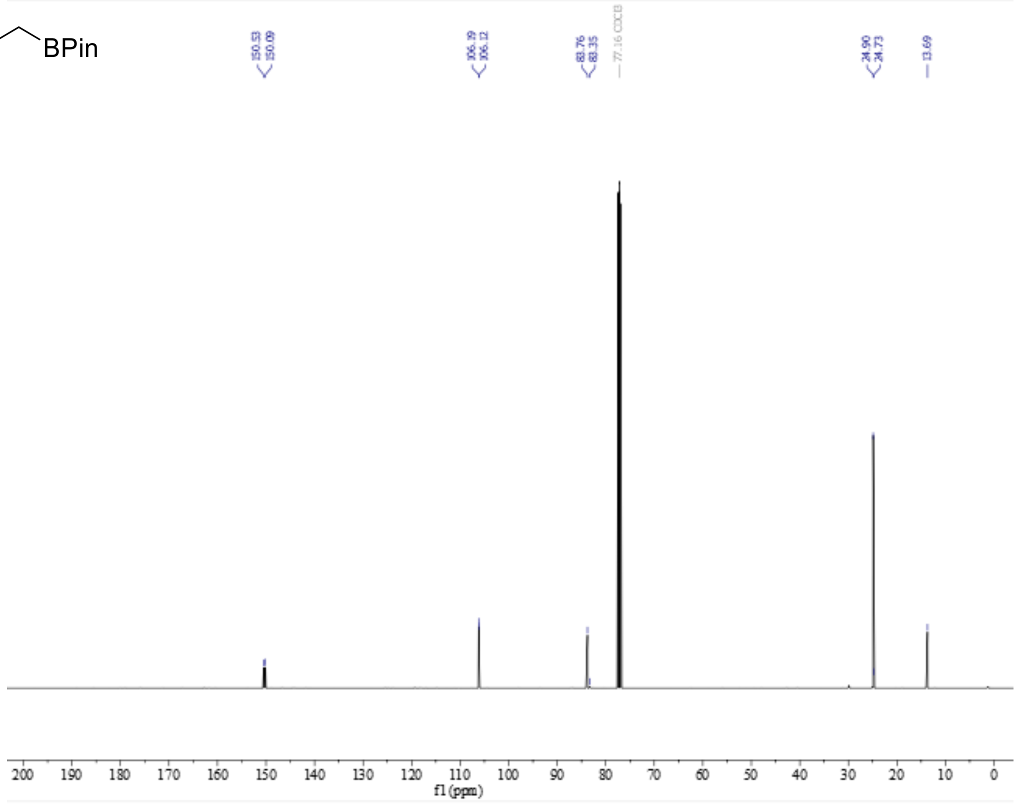

H-^{13}C-HSQC NMR – *α-boryl carbon atom highlighted*

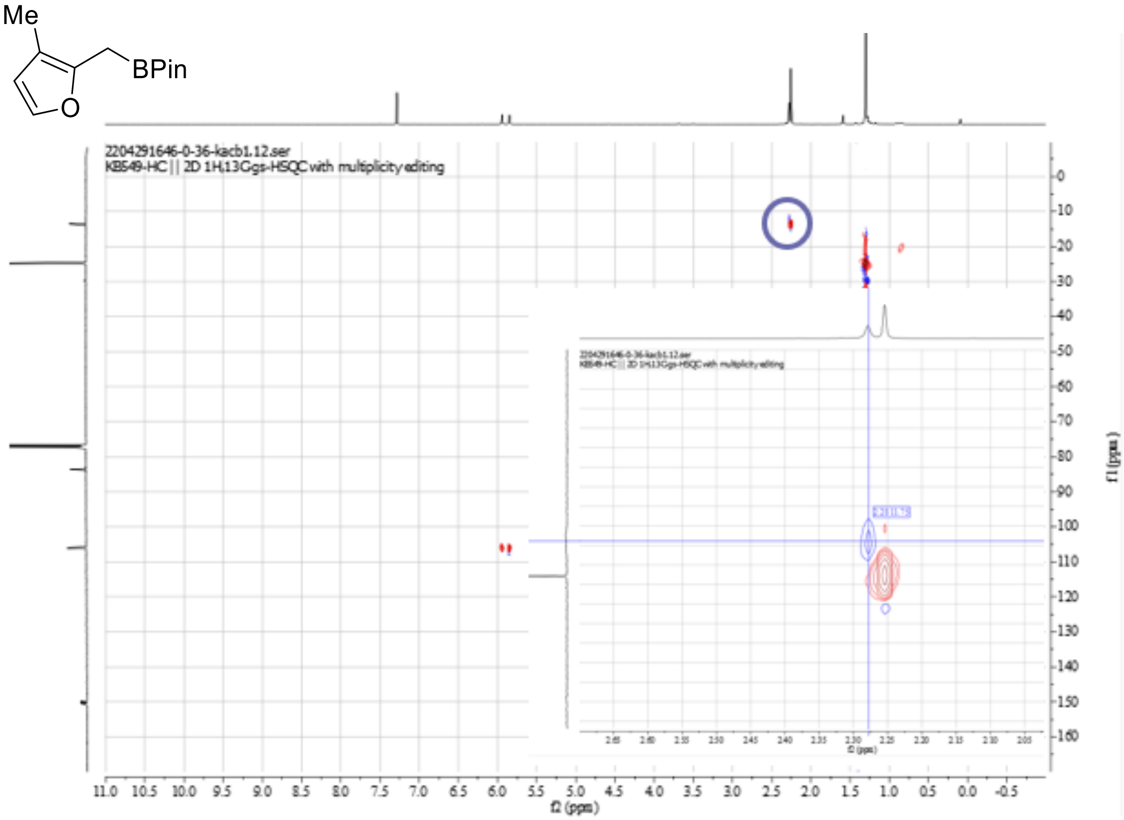

^{11}B NMR (96 MHz, $CDCl_3$)

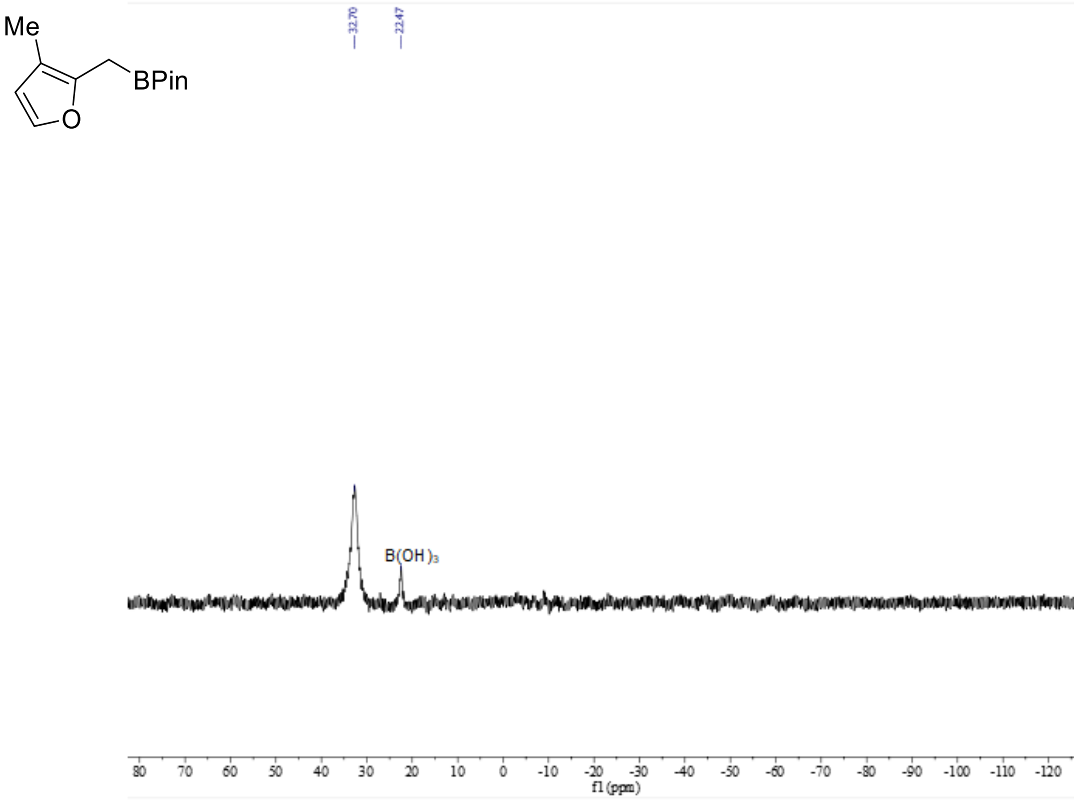

2-(furan-3-ylmethyl)-4,4,5,5-tetramethyl-1,3,2-dioxaborolane (44)

^{1}H NMR (500 MHz, $CDCl_3$)

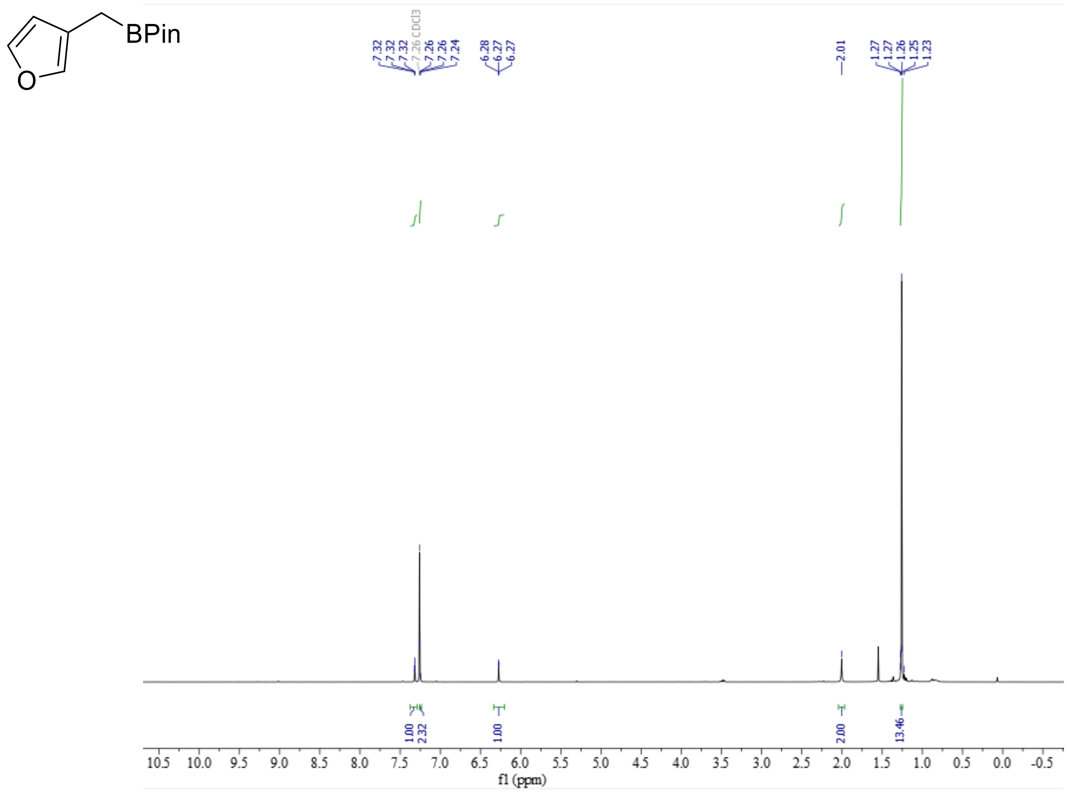

^{13}C NMR (126 MHz, $CDCl_3$)

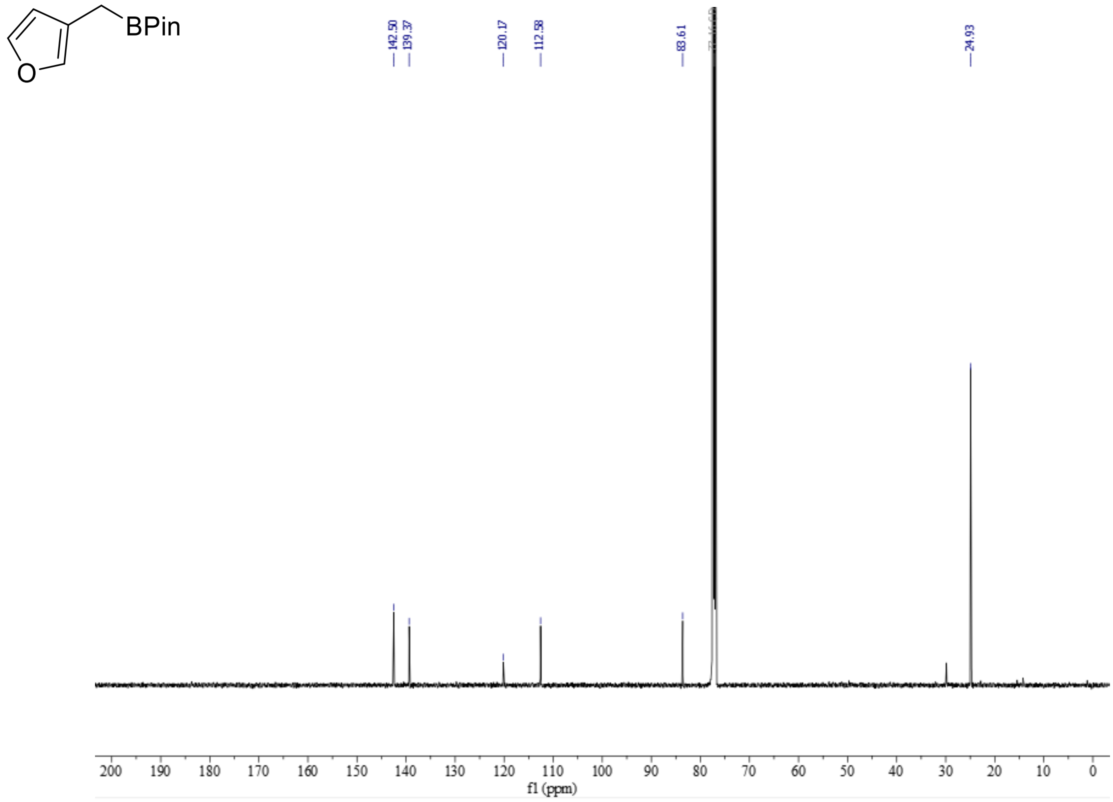

H-^{13}C-HSQC NMR – *^{1}H m masked by CDCl3 highlighted*

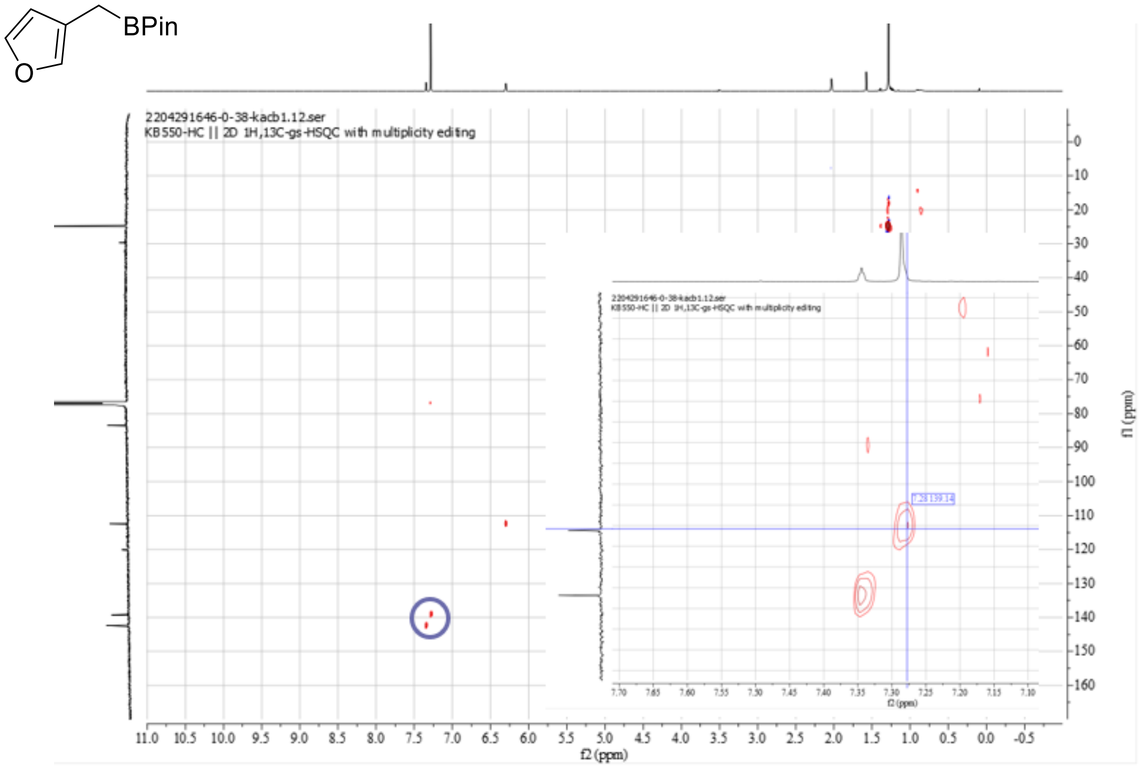

^{11}B NMR (96 MHz, $CDCl_3$)

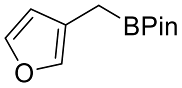

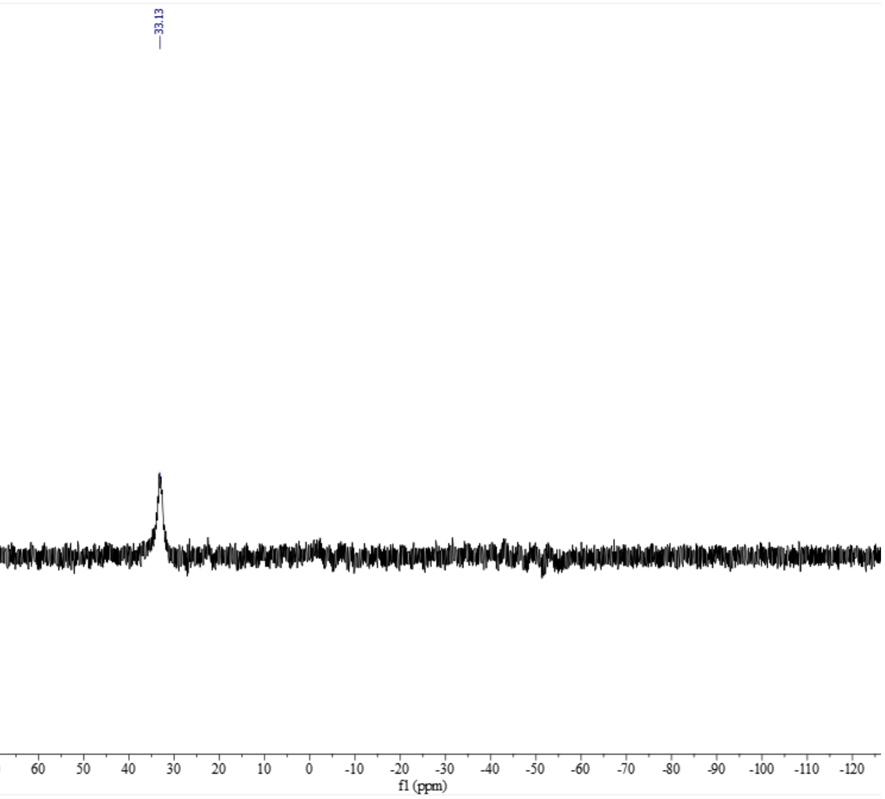

3,5-dimethyl-4-((4,4,5,5-tetramethyl-1,3,2-dioxaborolan-2-yl)methyl)isoxazole (45)

^{1}H NMR (500 MHz, $CDCl_3$)

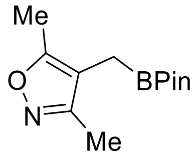

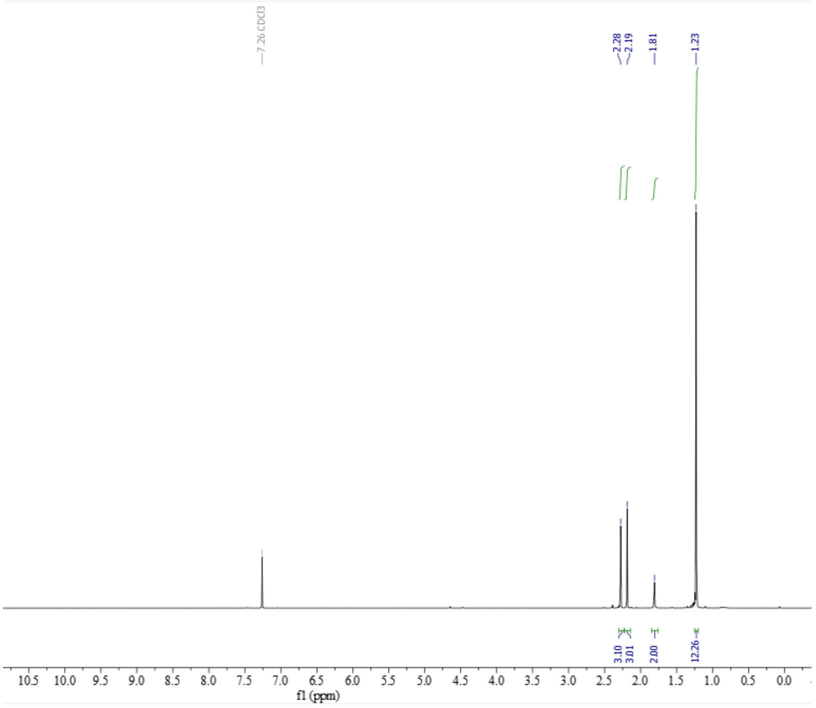

^{13}C NMR (126 MHz, $CDCl_3$)

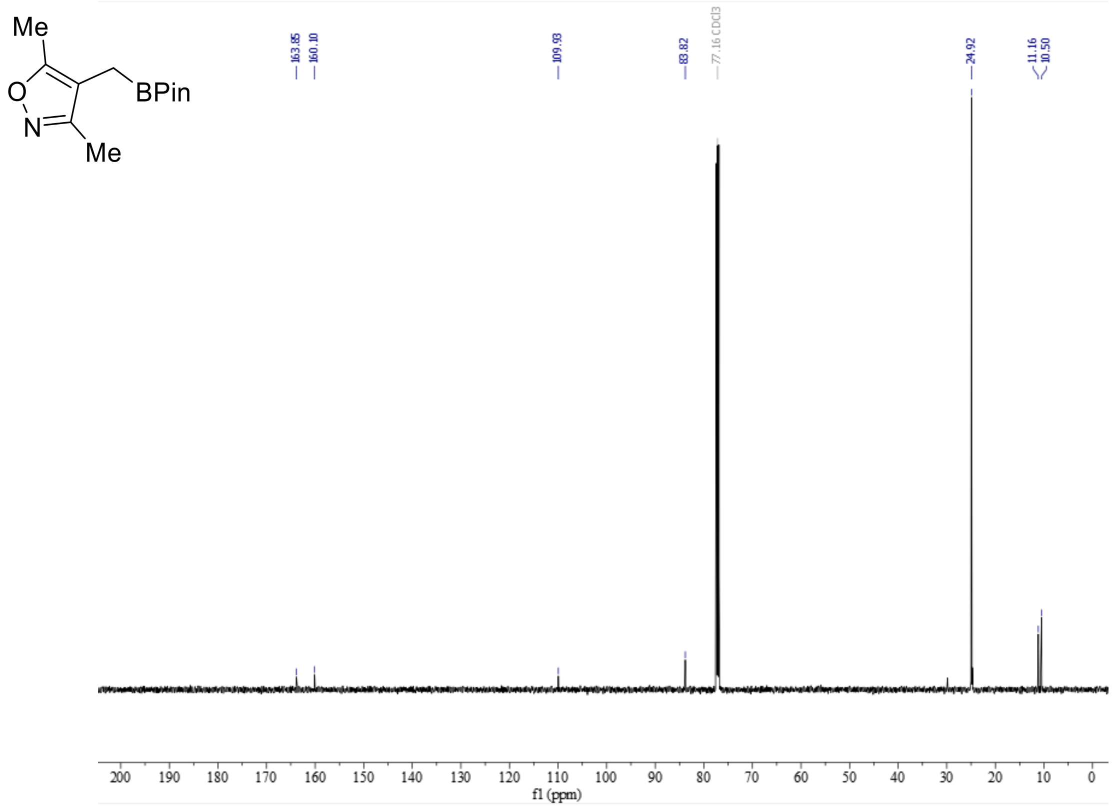

^{11}B NMR (96 MHz, CDCl$_3$)

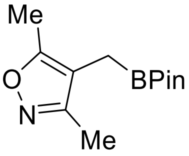

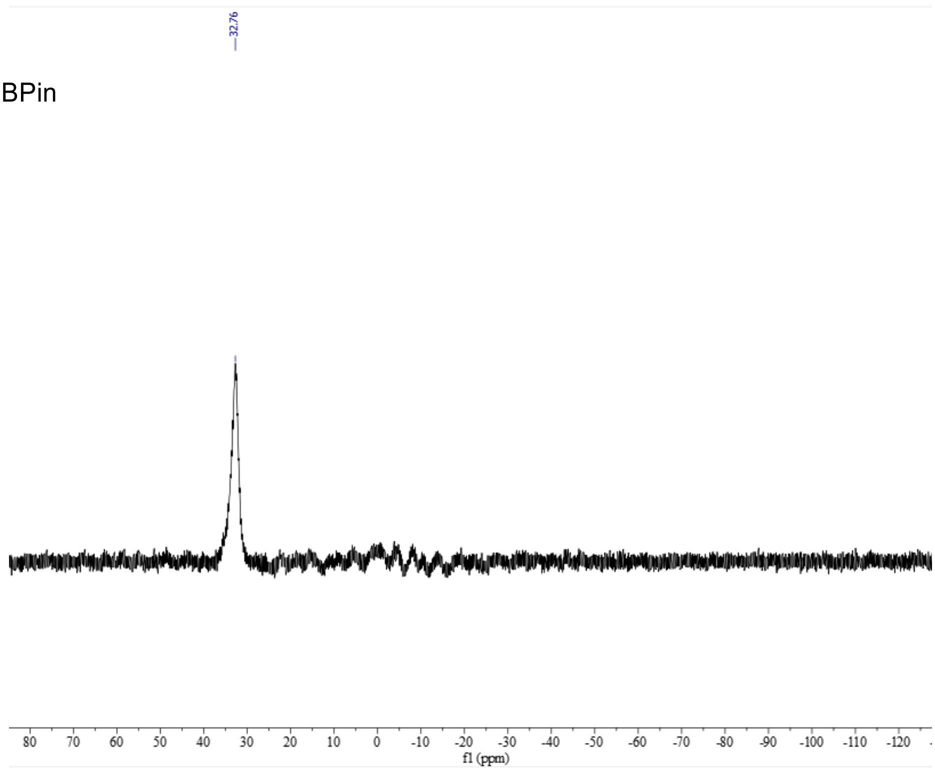

diphenylmethane (52)

^{1}H NMR (500 MHz, CDCl$_3$)

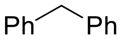

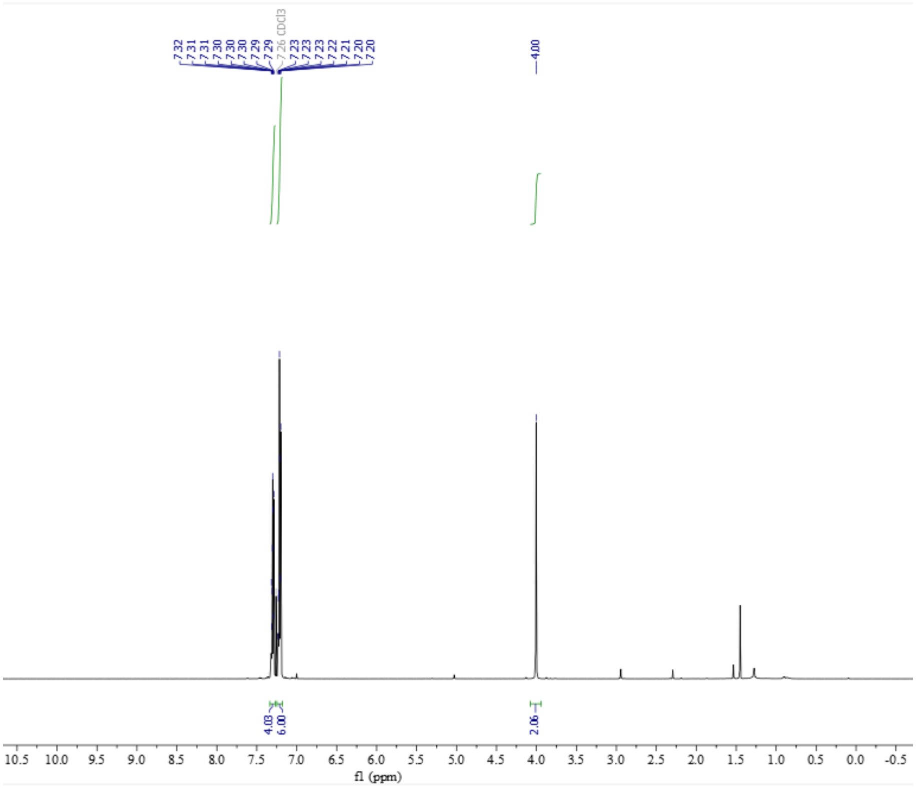

^{13}C NMR (126 MHz, CDCl3)

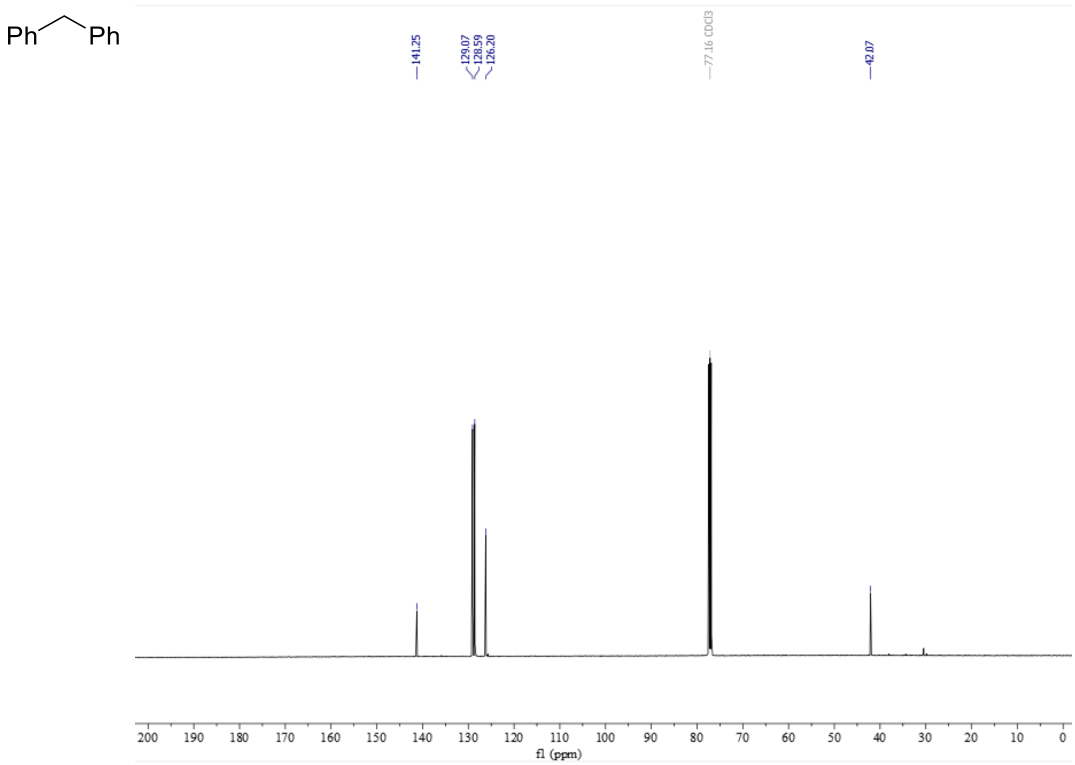

4-benzyl-1,1'-biphenyl (53)

^{1}H NMR (500 MHz, CDCl3)

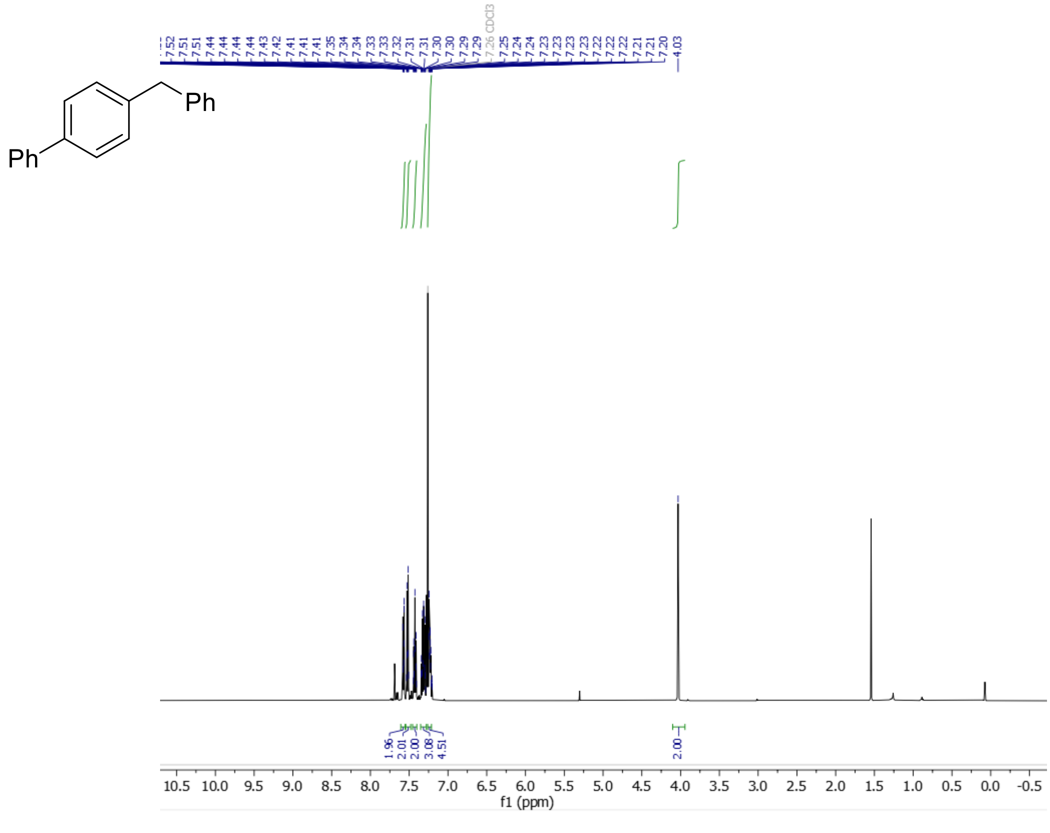

^{13}C NMR (126 MHz, CDCl$_3$)

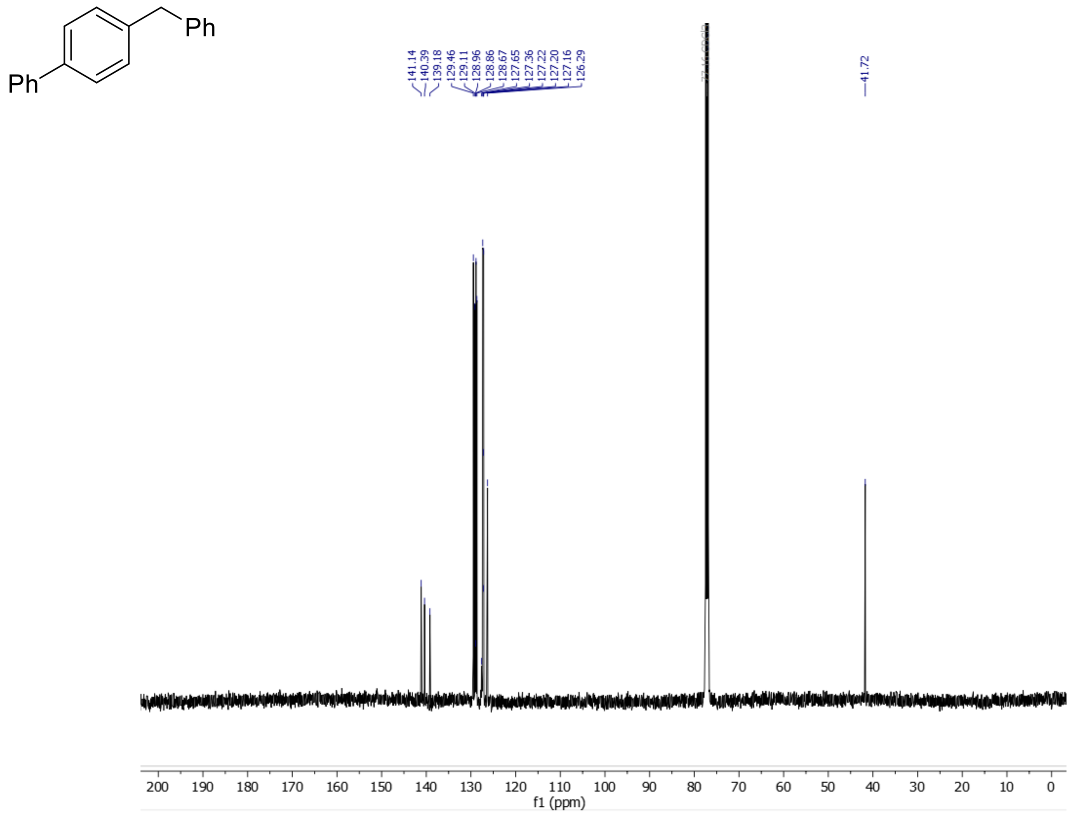

bifonazole (54)

^{1}H NMR (500 MHz, CDCl$_3$)

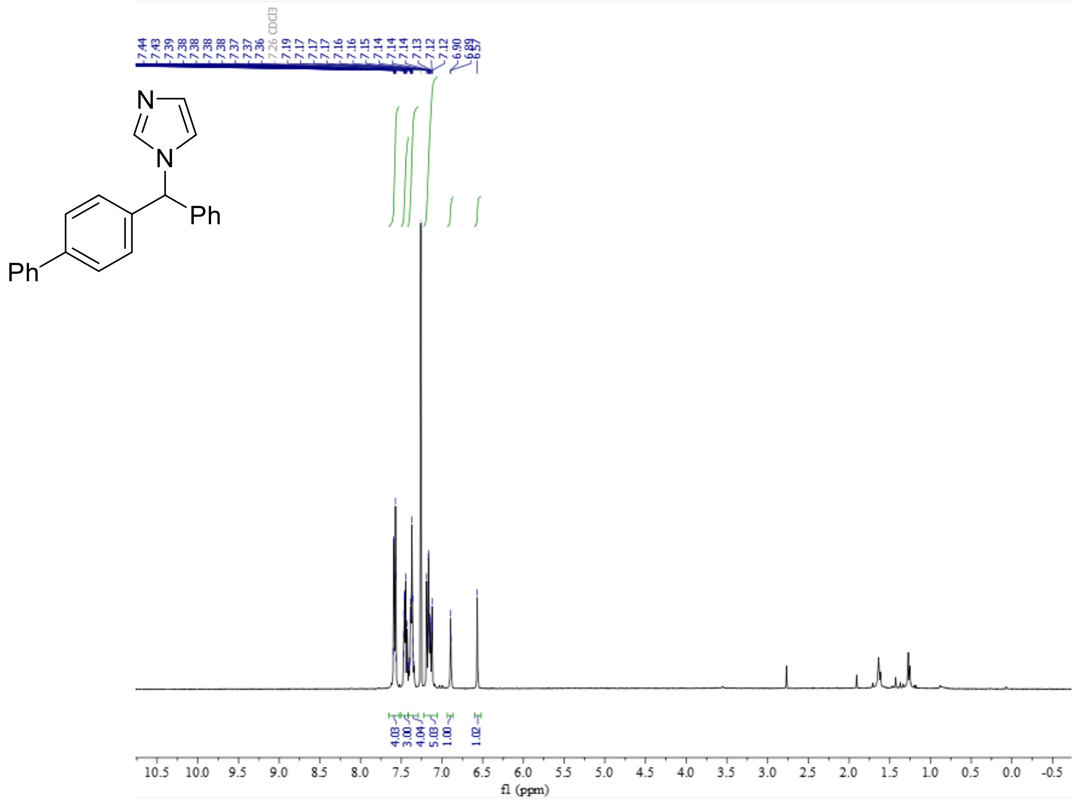

^{13}C NMR (126 MHz, CDCl$_3$)

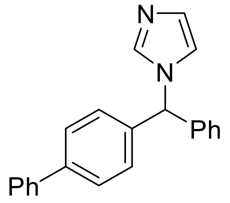

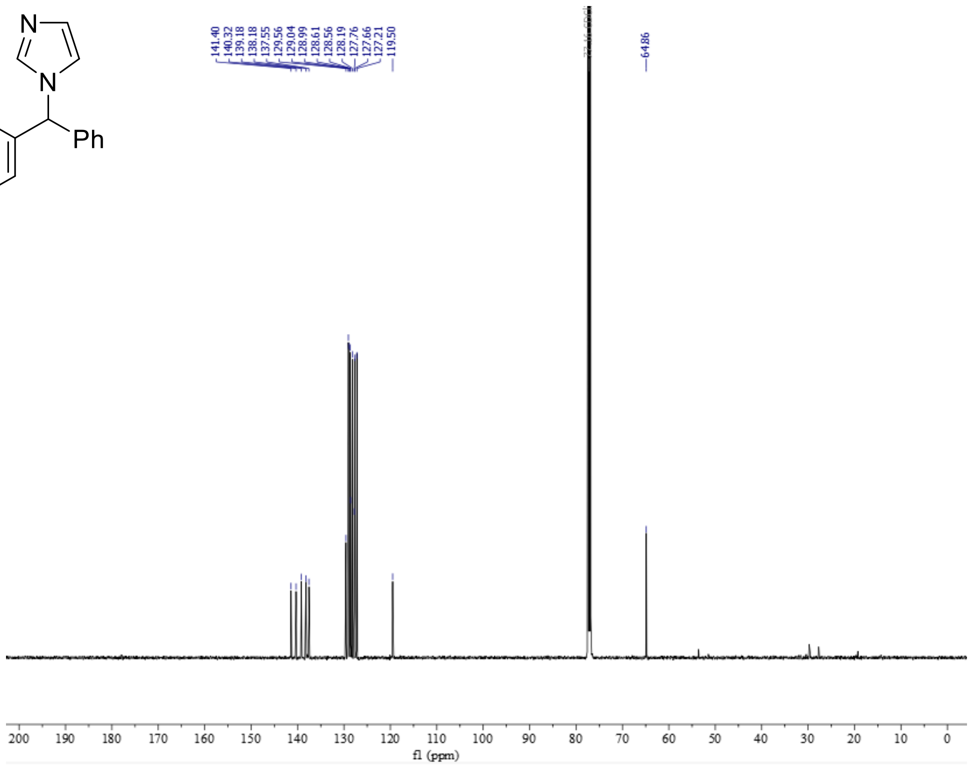

cyclizine (55)

^{1}H NMR (500 MHz, CDCl$_3$)

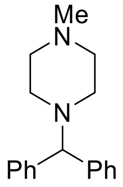

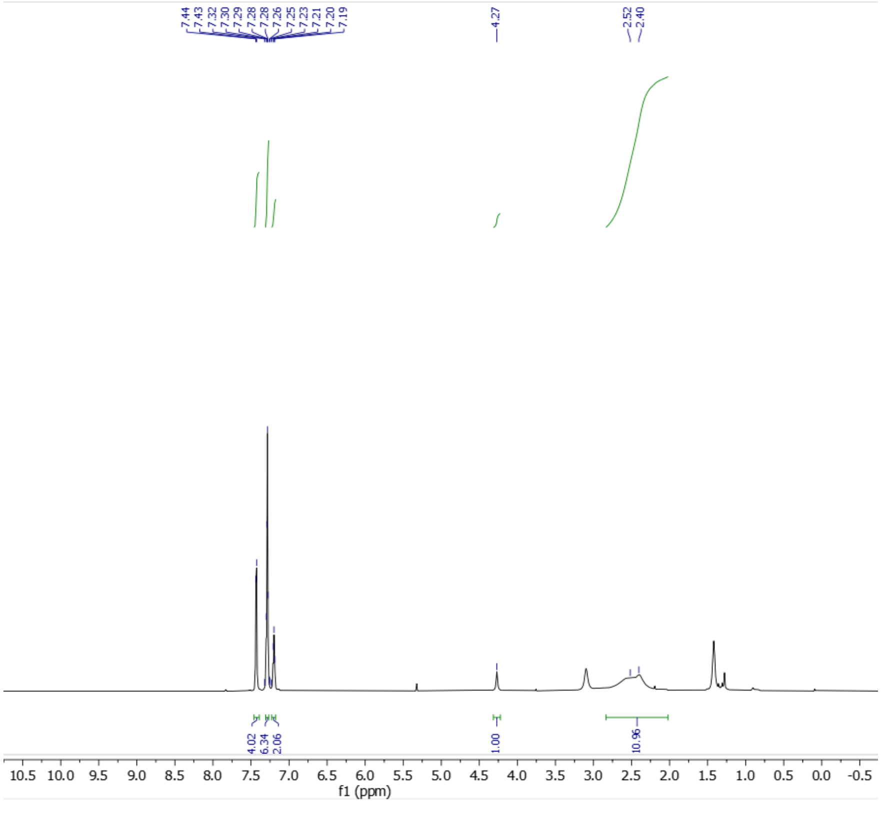

^{13}C NMR (126 MHz, CDCl$_3$)

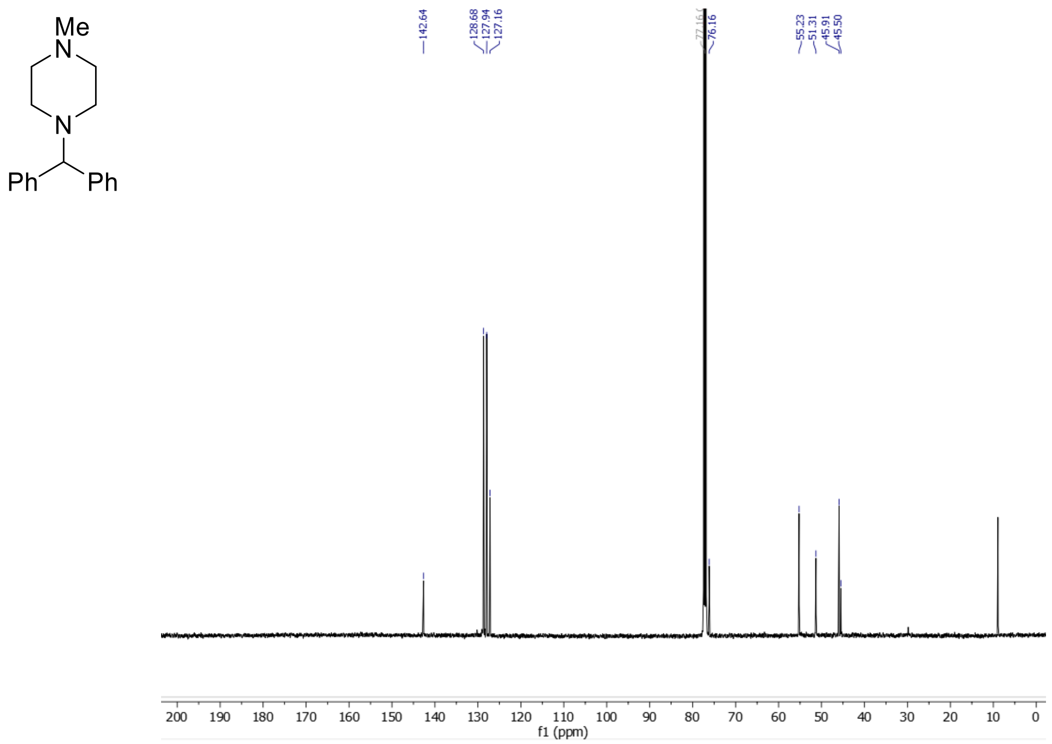

4,4,5,5-tetramethyl-2-((4'-(trifluoromethoxy)-[1,1'-biphenyl]-4-yl)methyl)-1,3,2-dioxaborolane (56)

^{1}H NMR (500 MHz, CDCl$_3$)

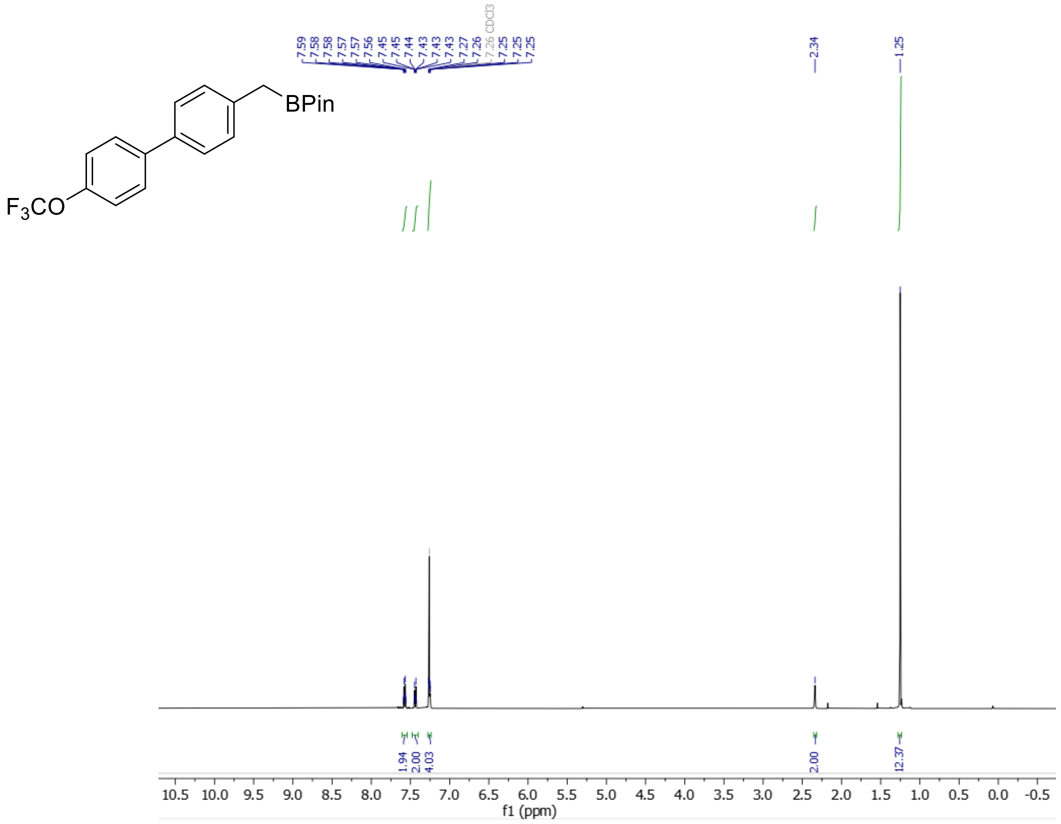

^{13}C NMR (126 MHz, CDCl$_3$)

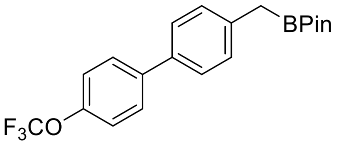

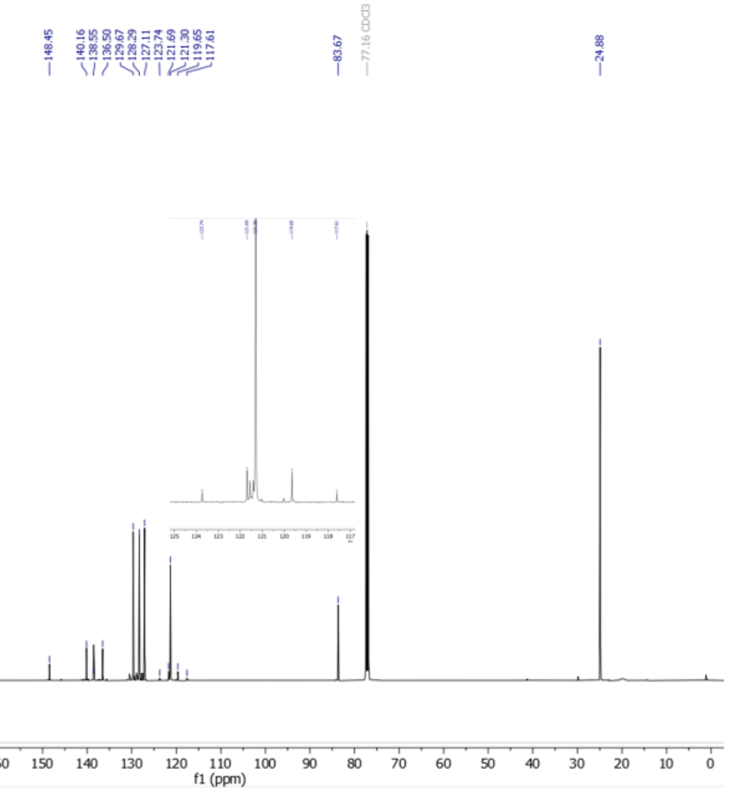

^{11}B NMR (96 MHz, CDCl$_3$)

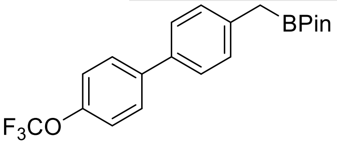

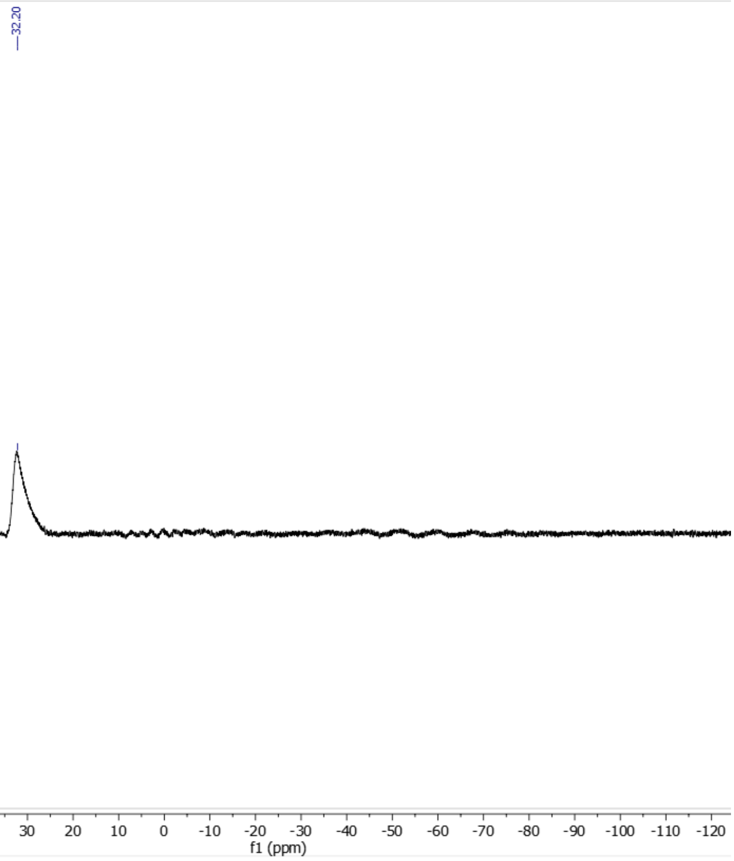

^{19}F NMR (470 MHz, $CDCl_3$)

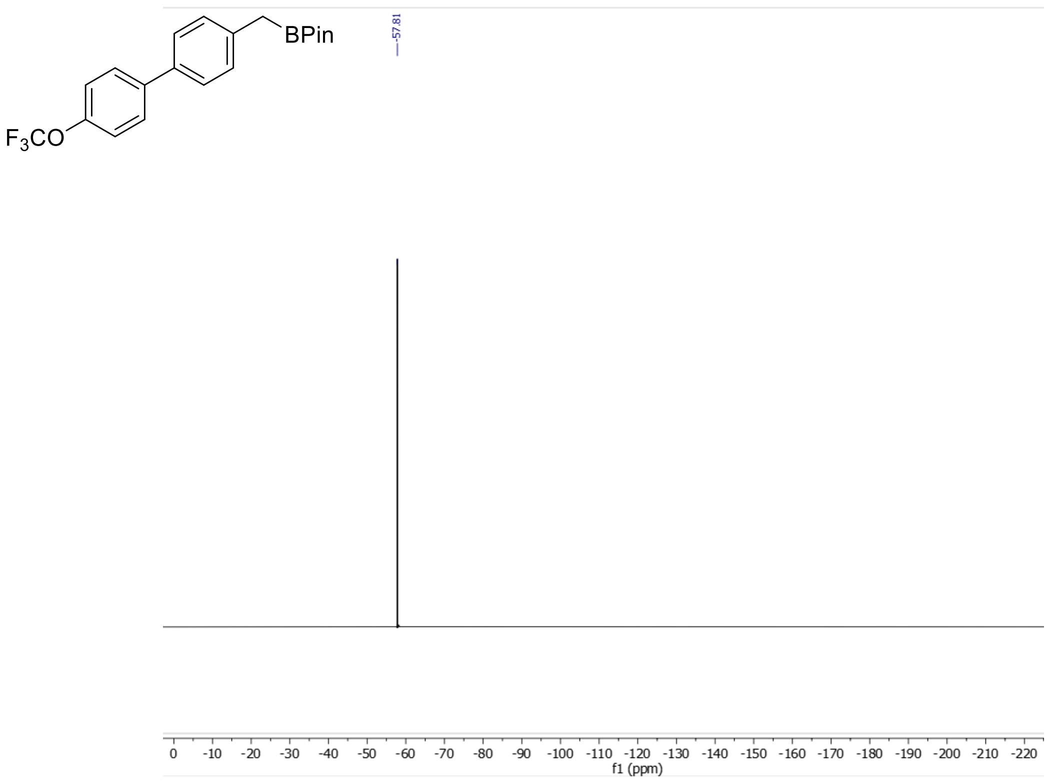

1-chloro-4-(4-methoxybenzyl)benzene (57)

^{1}H NMR (500 MHz, $CDCl_3$)

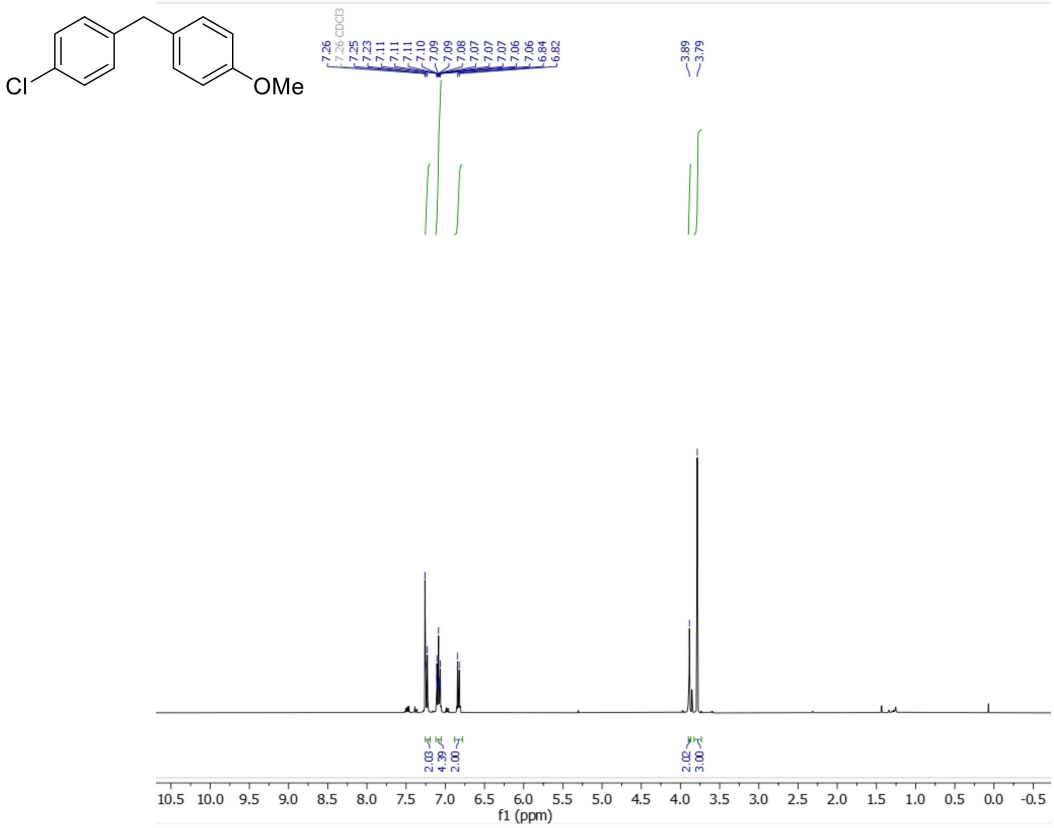

^{13}C NMR (126 MHz, CDCl$_3$)

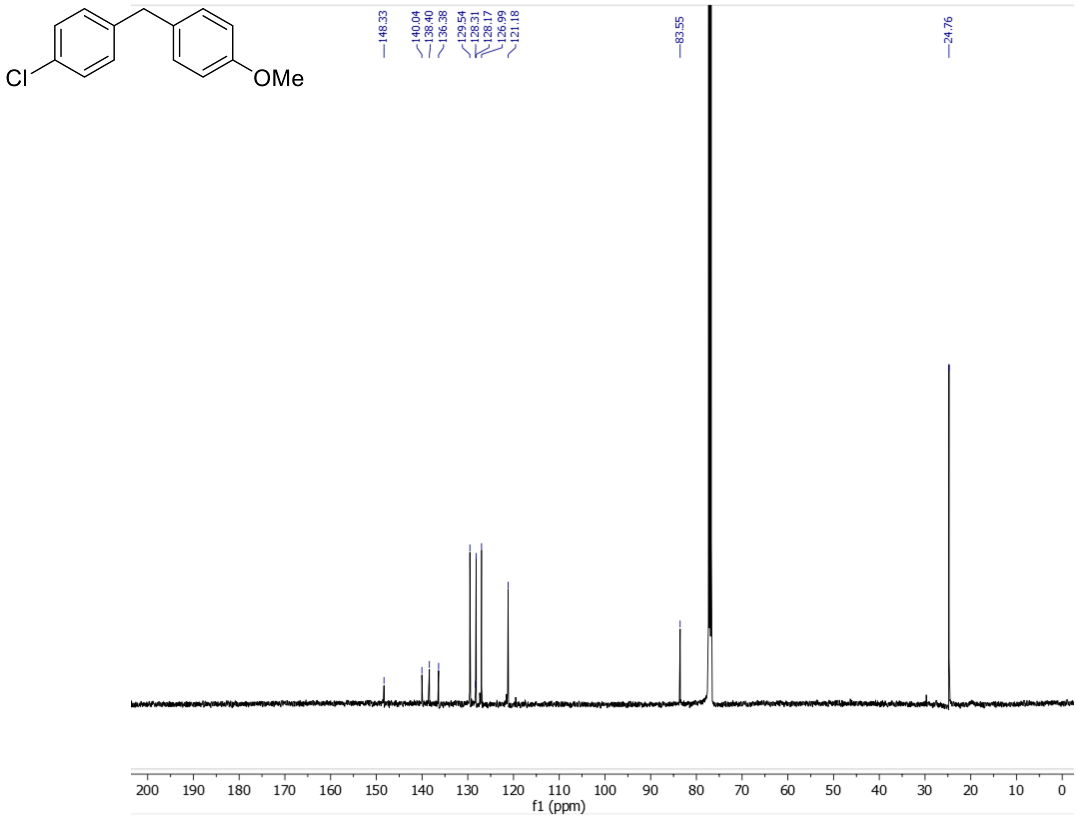

potassium bromomethyltetrafluoroborate (64)

^{1}H NMR (500 MHz, $(CD_3)_2CO$)

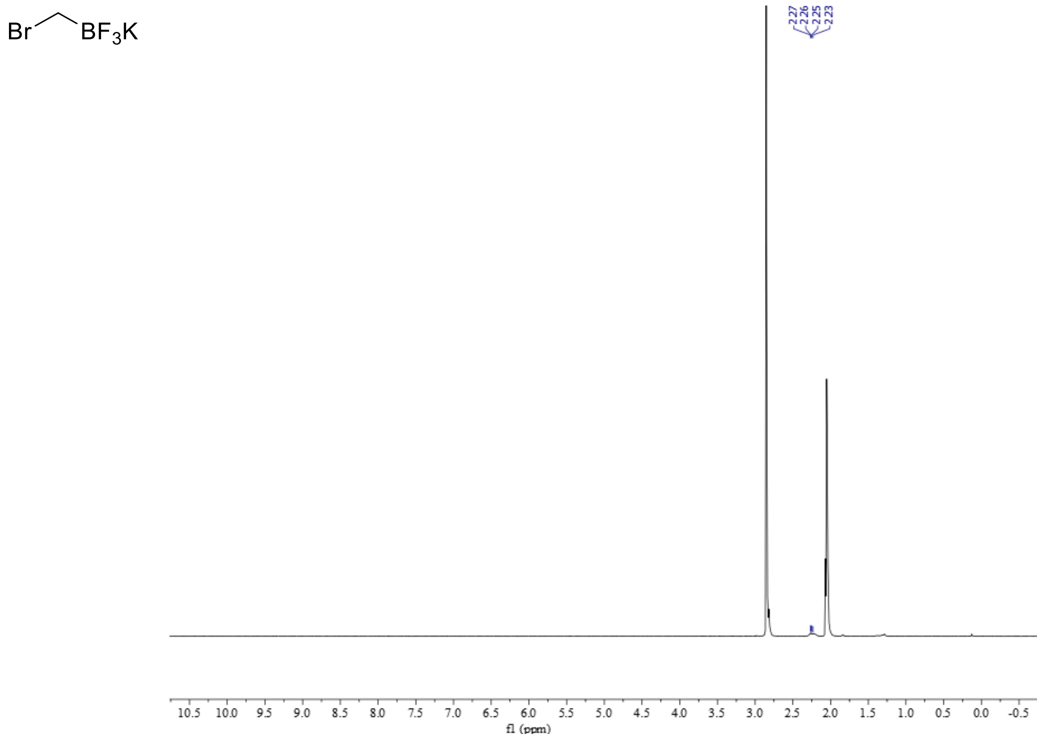

^{13}C NMR (126 MHz, $(CD_3)_2CO$)

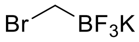

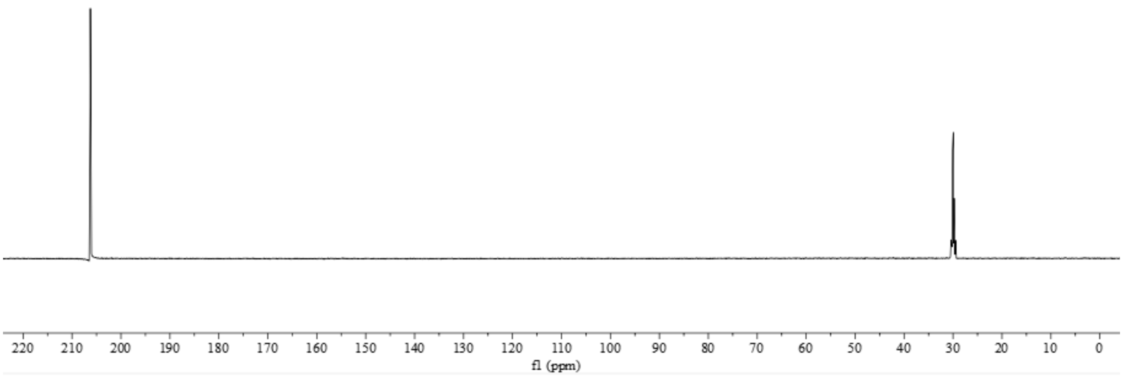

^{11}B NMR (96 MHz, $(CD_3)_2CO$)

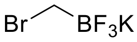

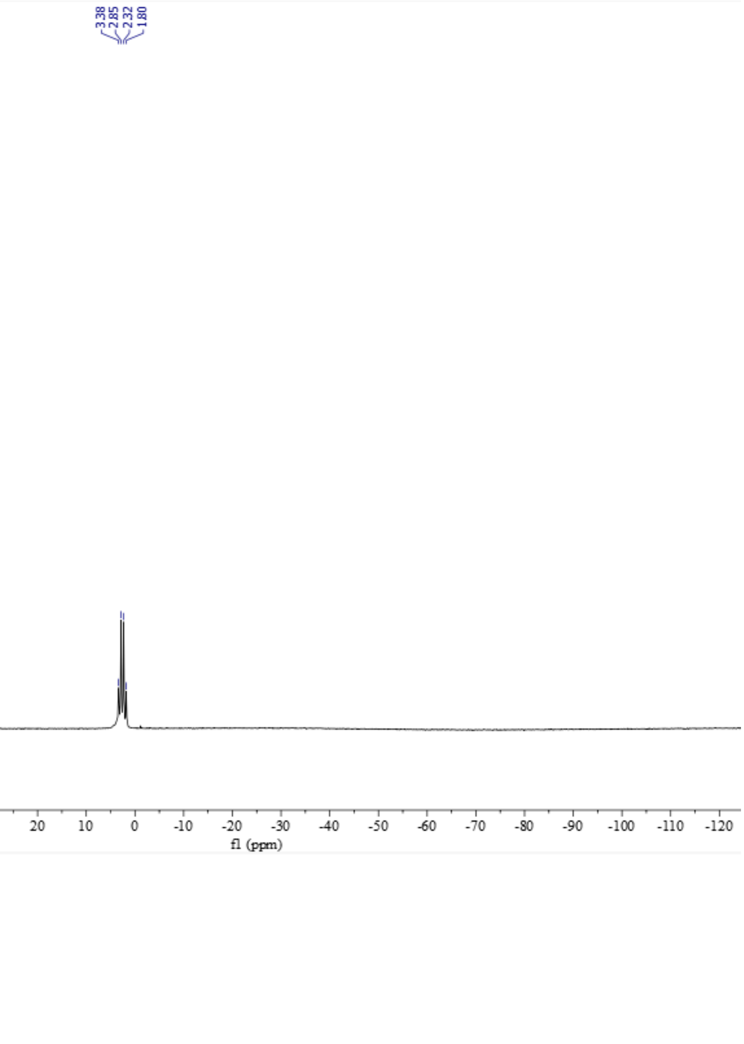

^{19}F NMR (470 MHz, $(CD_3)_2CO$)

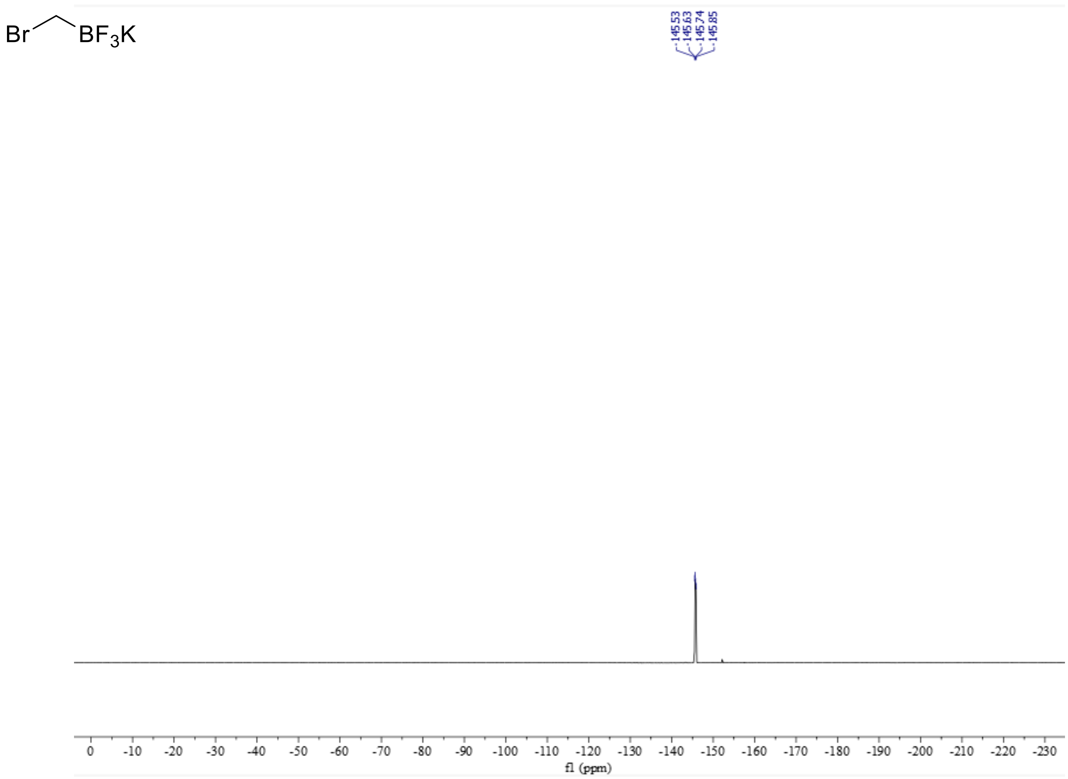

2-(bromomethyl)-1,3,2-dioxaborolane (65)

^{1}H NMR (500 MHz, $CDCl_3$)

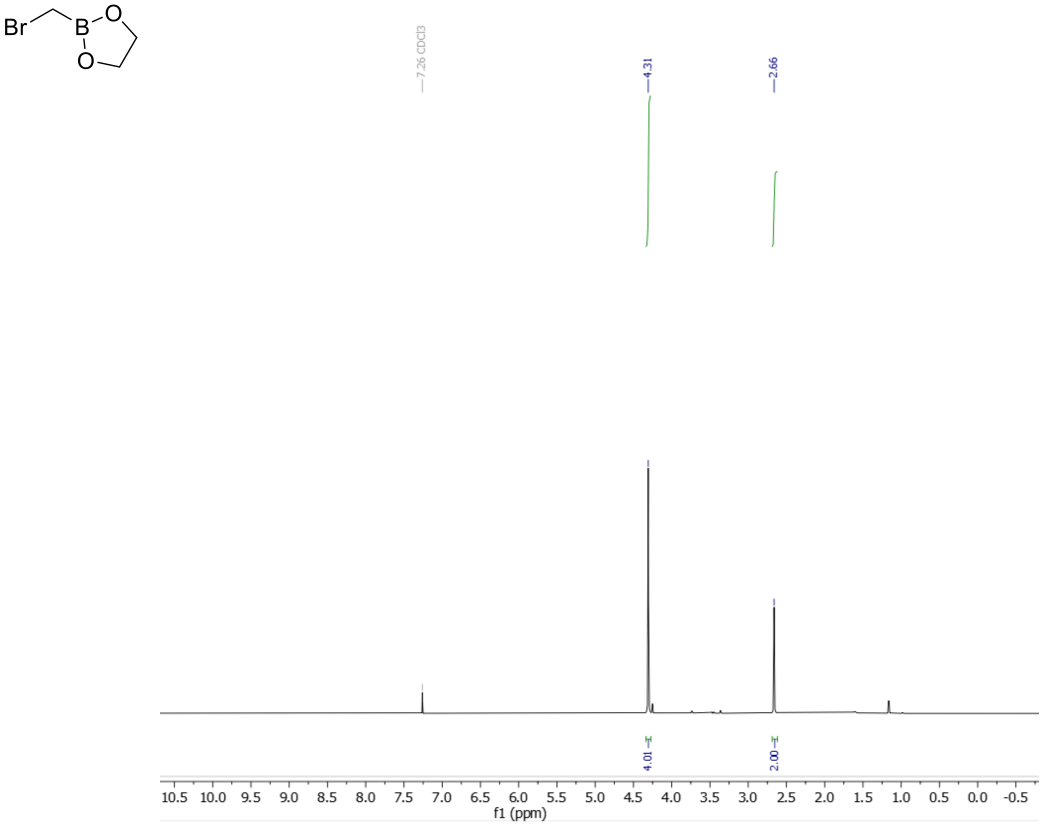

^{13}C NMR (126 MHz, $CDCl_3$)

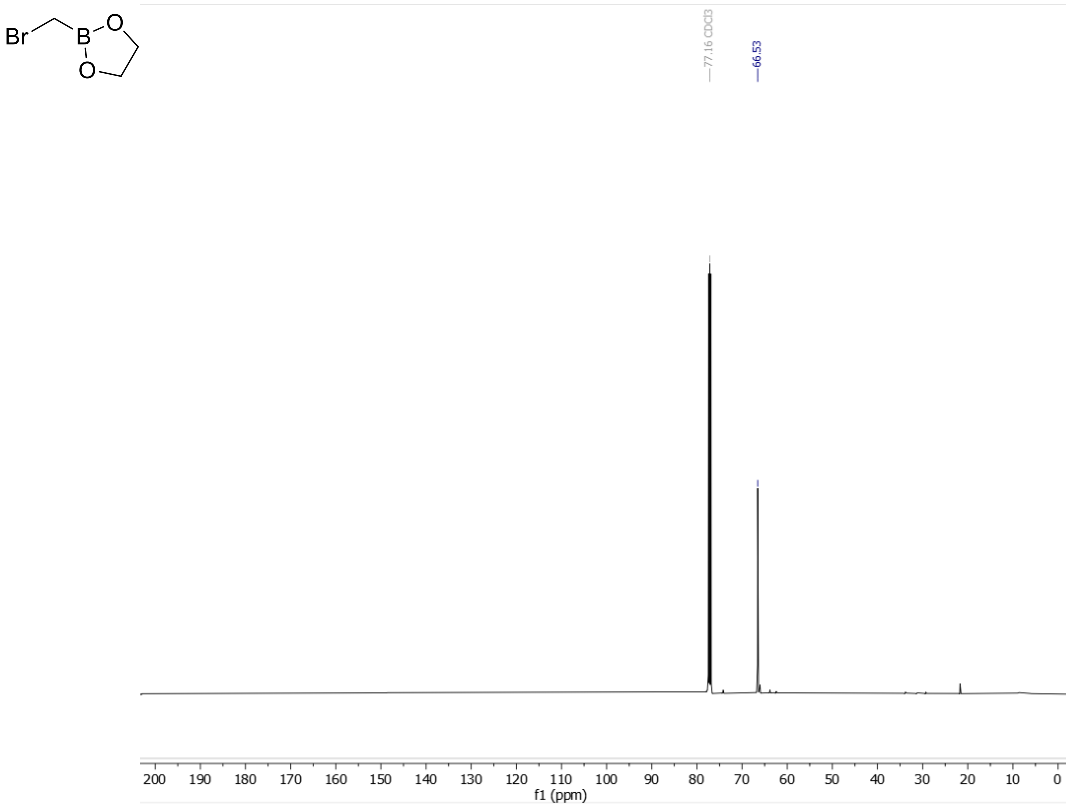

^{11}B NMR (96 MHz, $CDCl_3$)

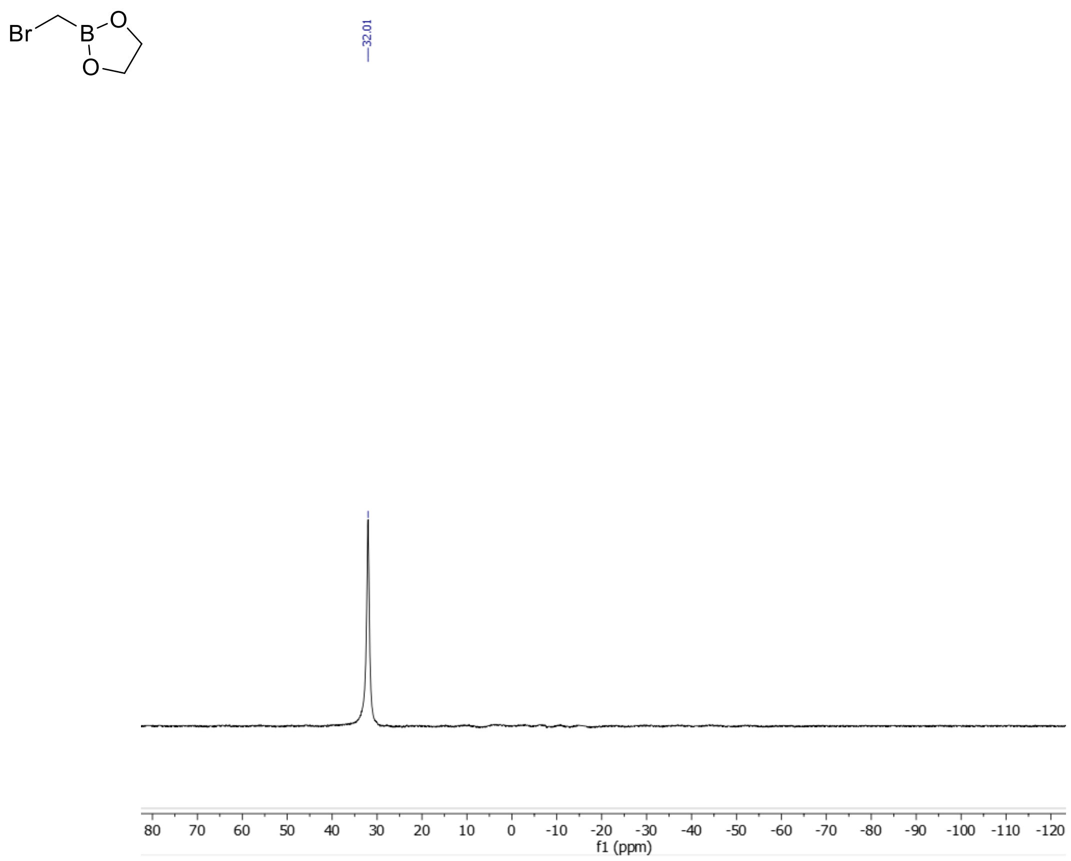

diisopropyl (4S,5S)-2-(bromomethyl)-1,3,2-dioxaborolane-4,5-dicarboxylate (66)

^{1}H NMR (500 MHz, $CDCl_3$)

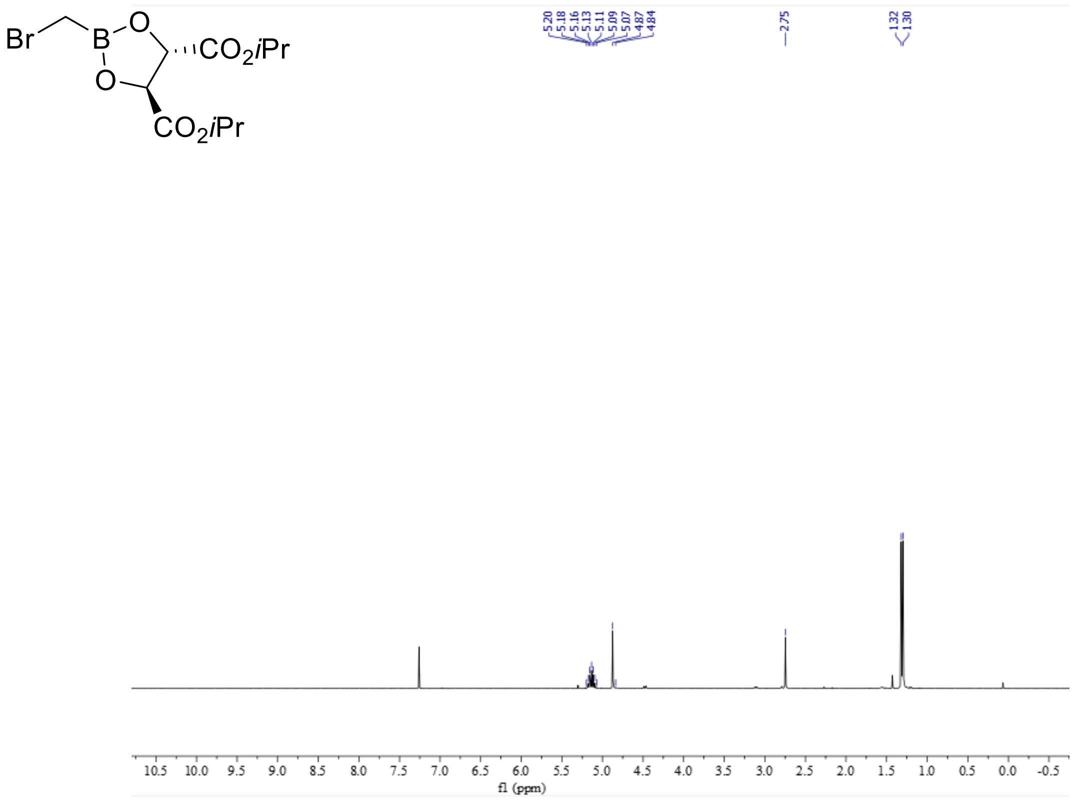

^{13}C NMR (126 MHz, $CDCl_3$)

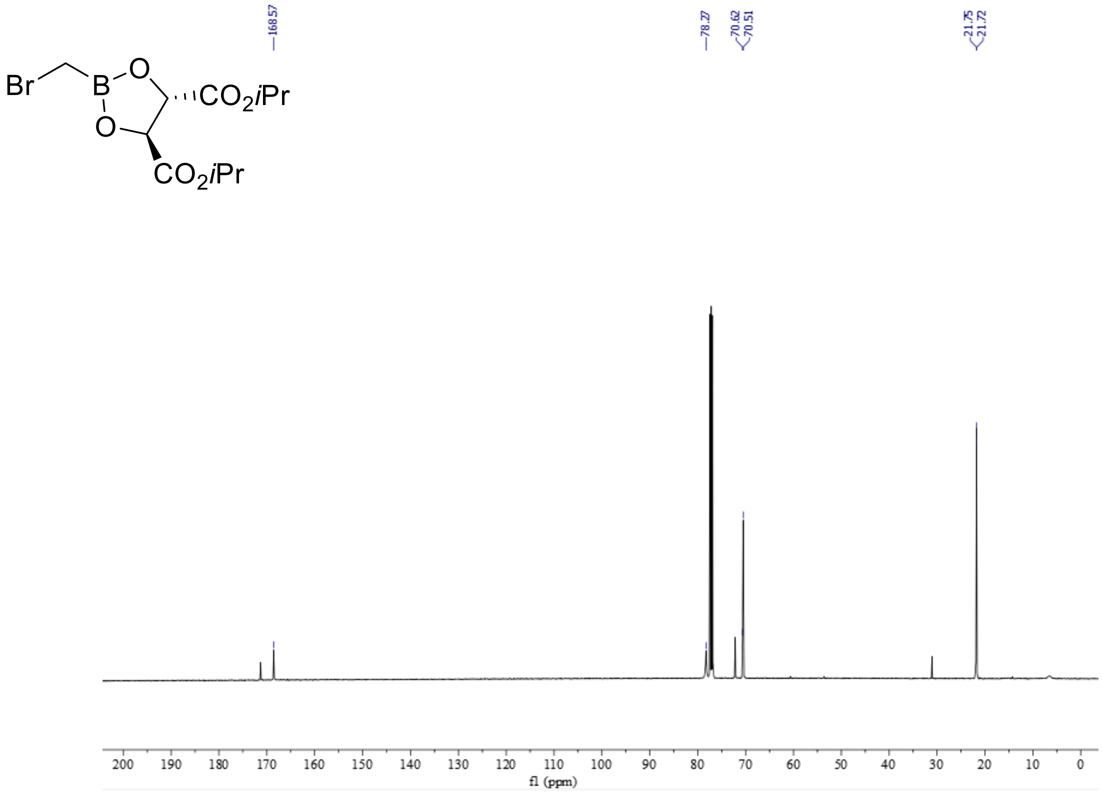

^{11}B NMR (96 MHz, $CDCl_3$)

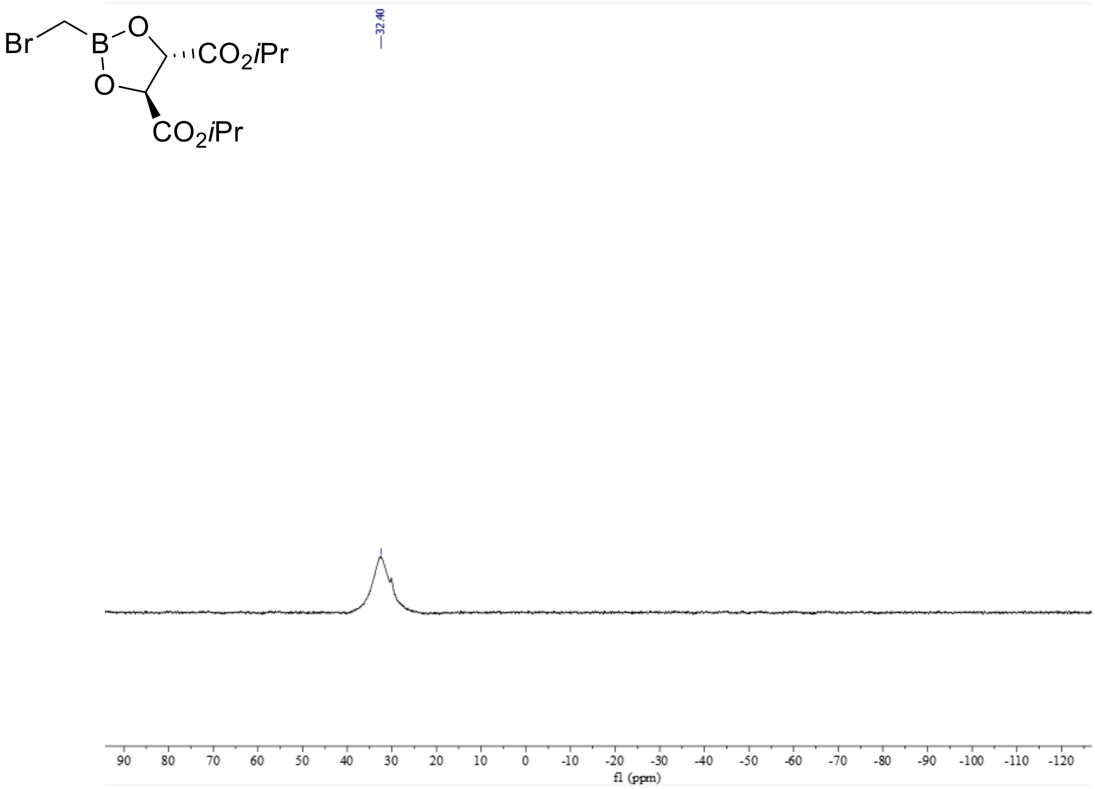

2-(bromomethyl)-1,3,2-dioxaborinane (67)

^{1}H NMR (500 MHz, $CDCl_3$)

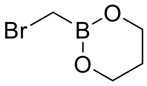

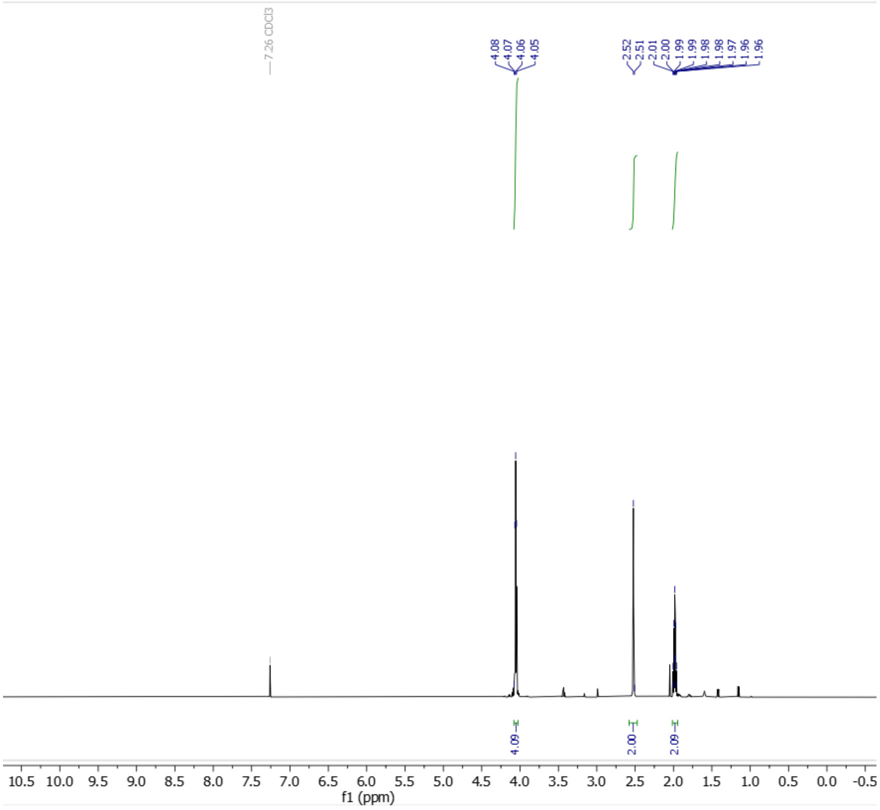

^{13}C NMR (126 MHz, $CDCl_3$)

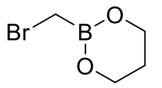

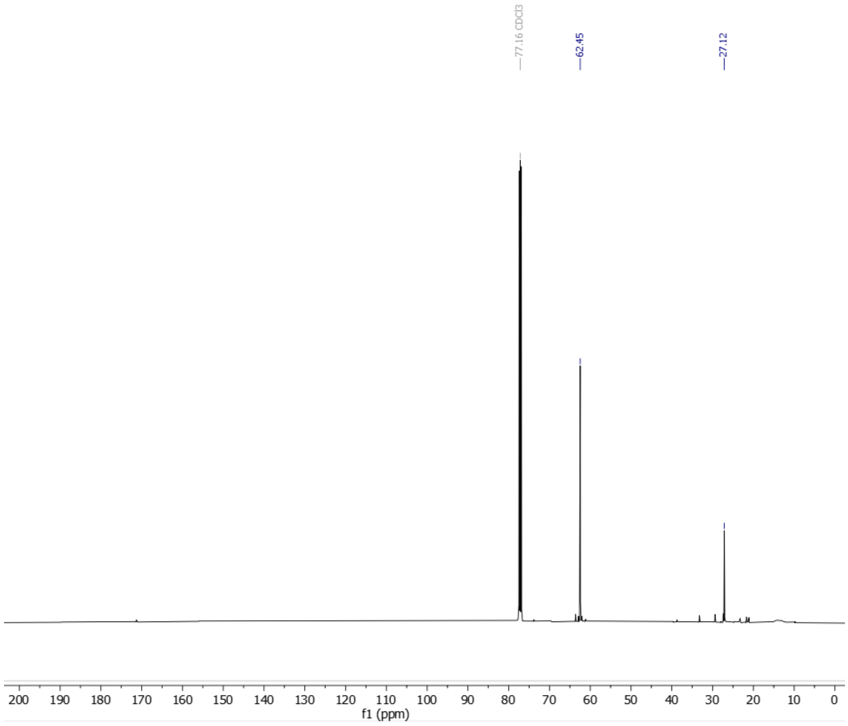

^{11}B NMR (96 MHz, $CDCl_3$)

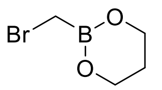

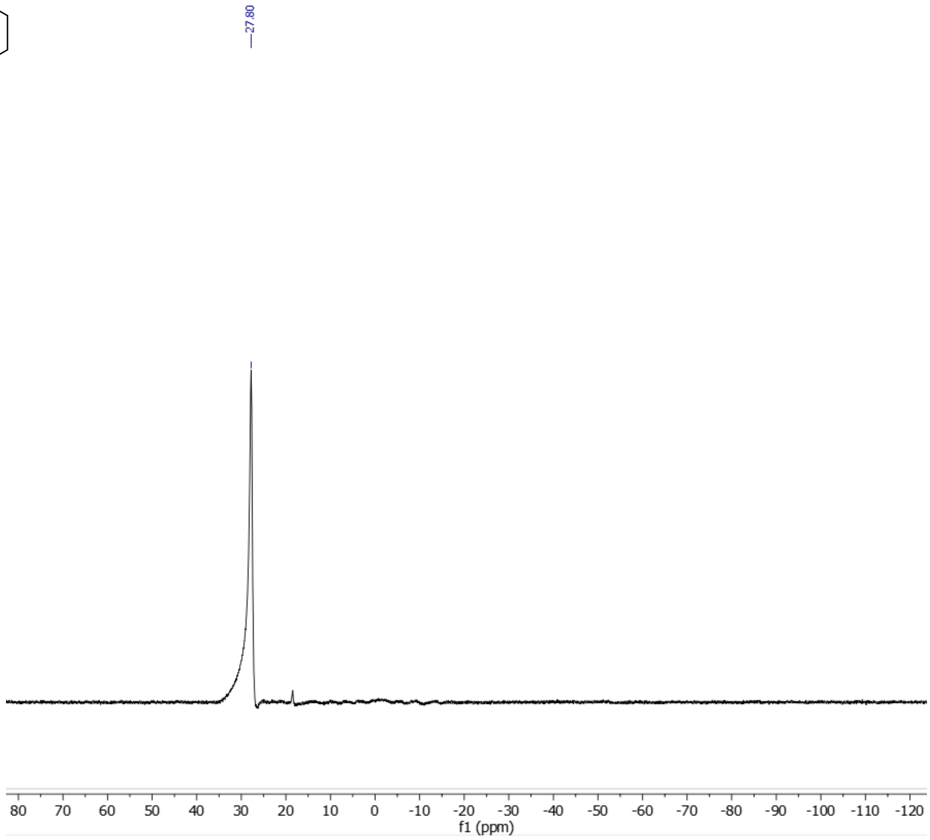

2-(bromomethyl)-5,5-dimethyl-1,3,2-dioxaborinane (68)

^{1}H NMR (500 MHz, $CDCl_3$)

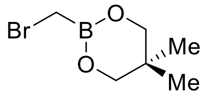

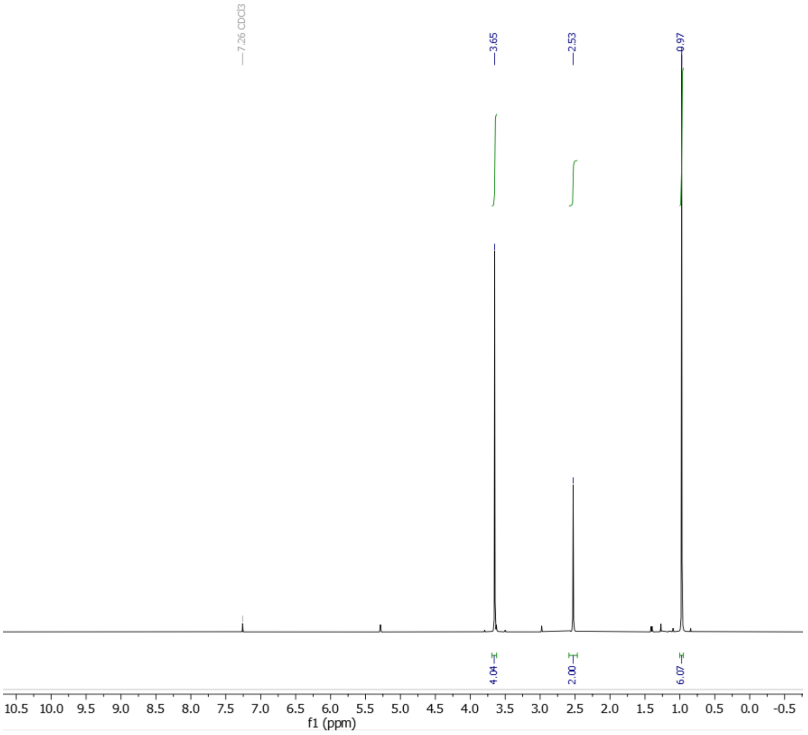

^{13}C NMR (126 MHz, $CDCl_3$)

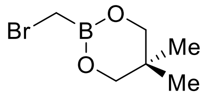

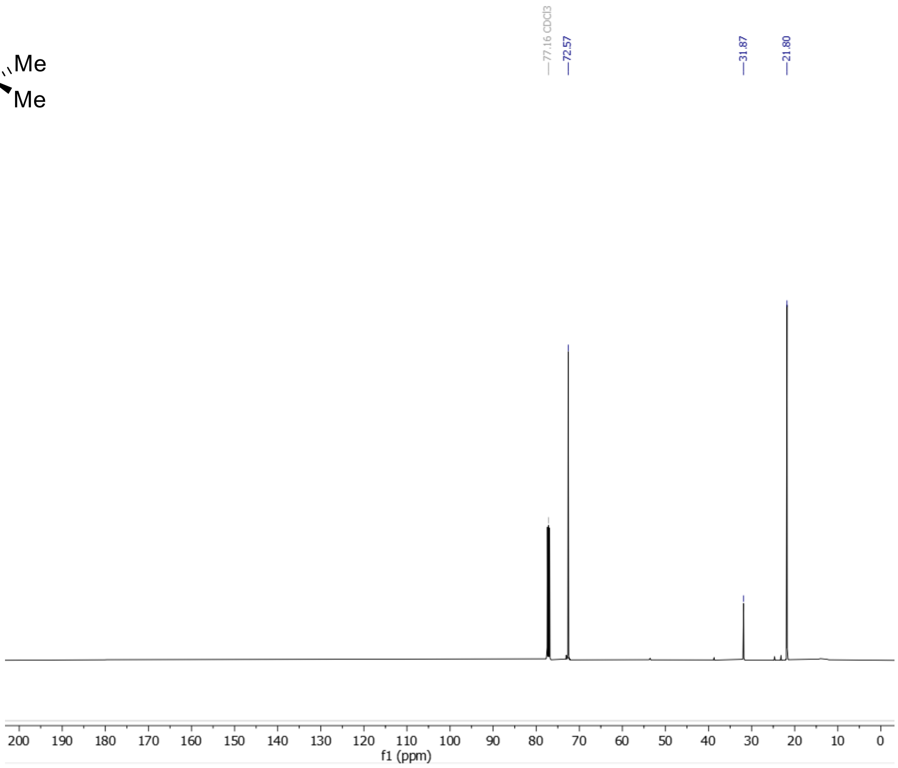

^{11}B NMR (96 MHz, $CDCl_3$)

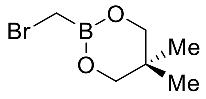

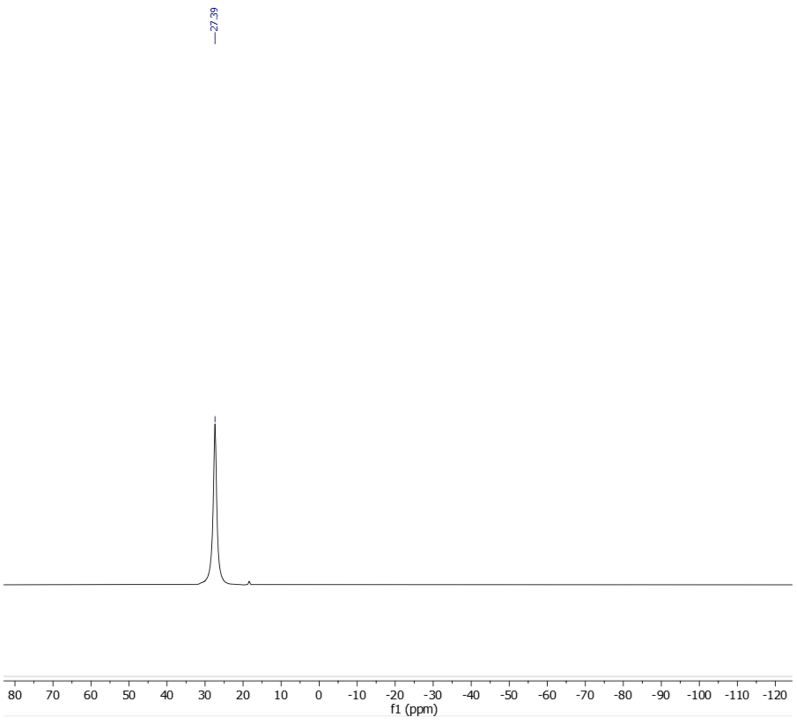

2-(bromomethyl)-4,6-dimethyl-1,3,2-dioxaborinane (69)

^{1}H NMR (500 MHz, $CDCl_3$)

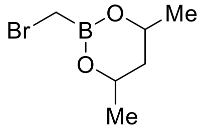

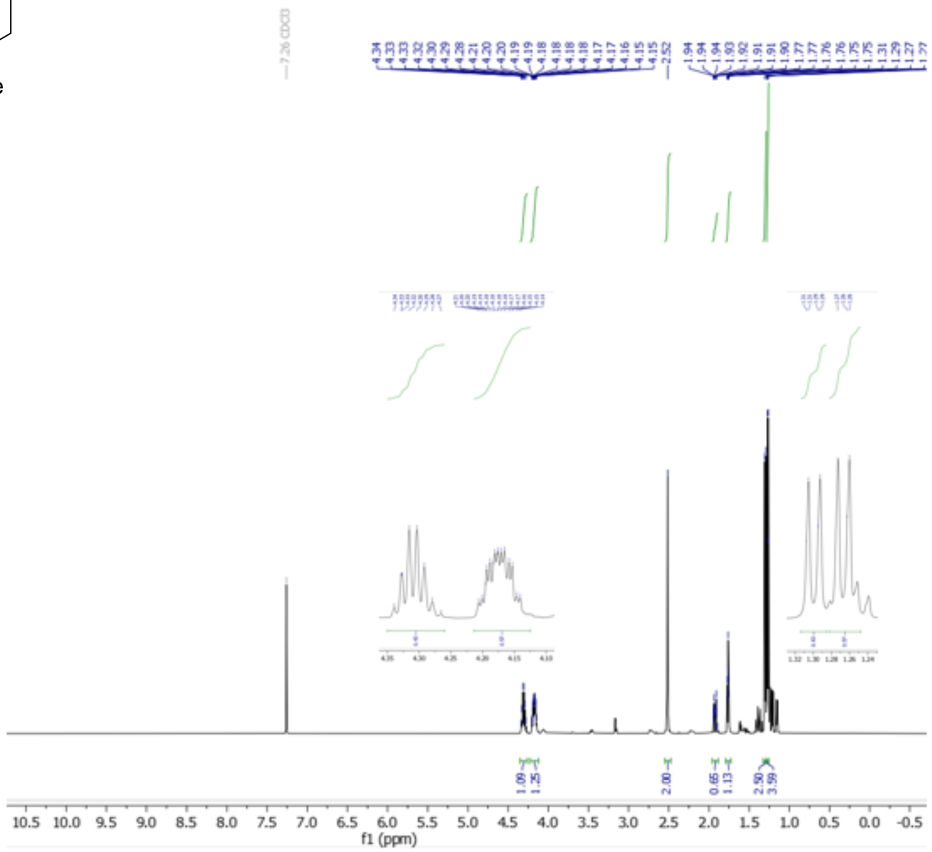

^{13}C NMR (126 MHz, $CDCl_3$)

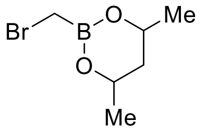

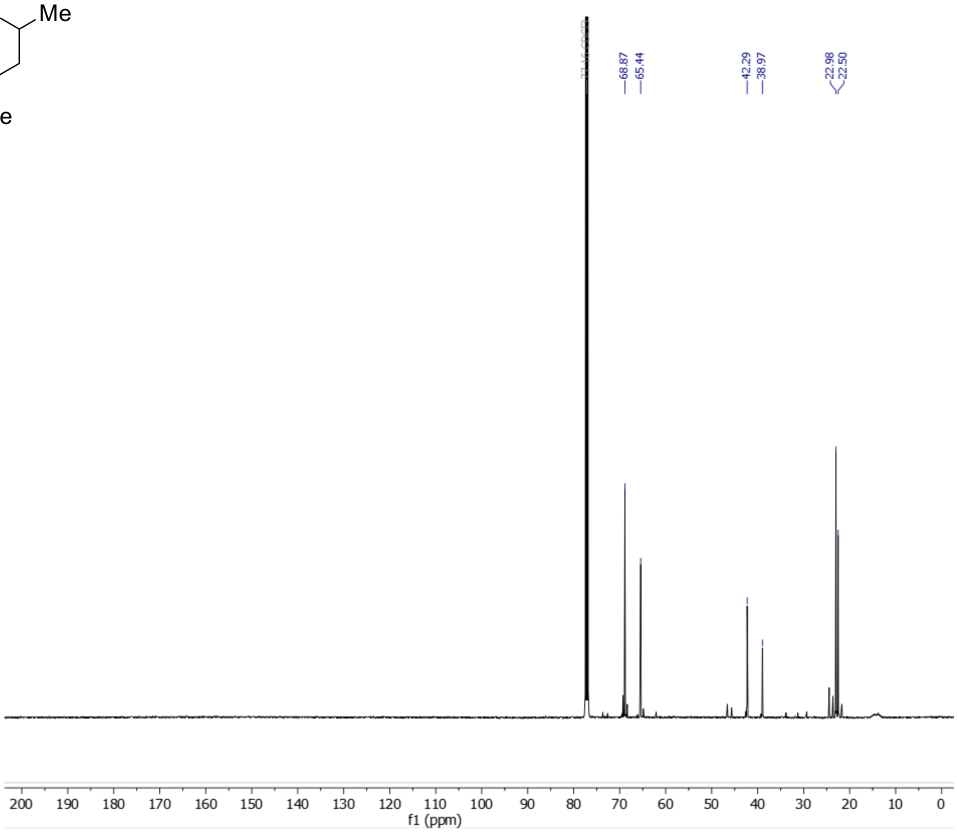

^{11}B NMR (96 MHz, $CDCl_3$)

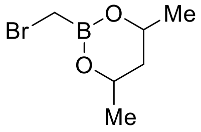

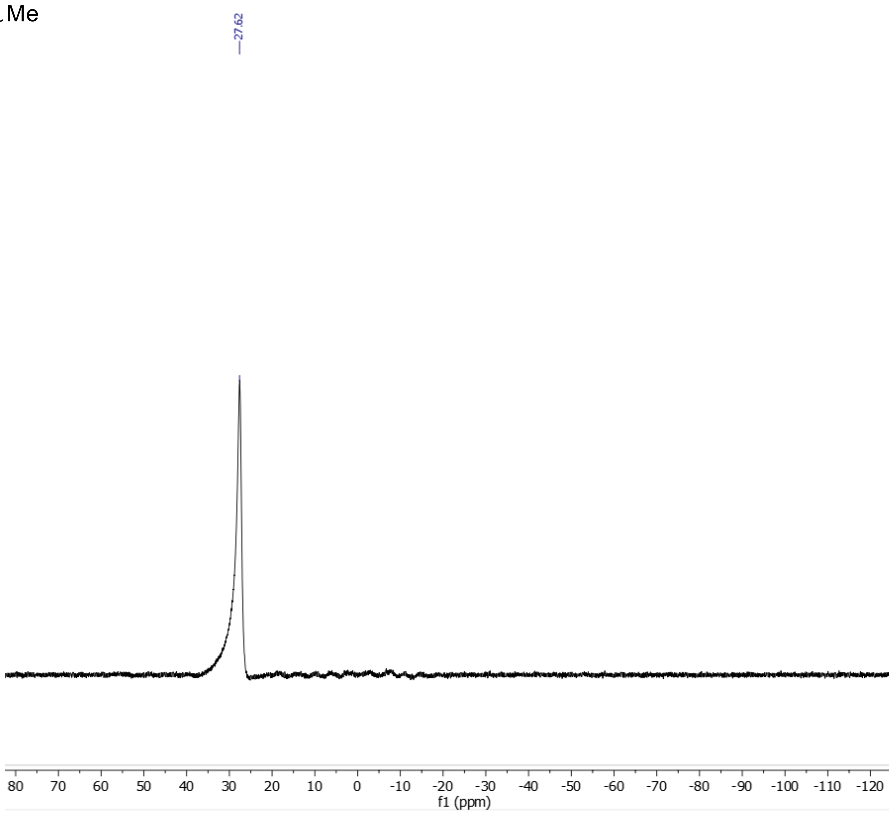

***N*-methyliminodiacetyl (bromomethyl)boronate (70)**

^{1}H NMR (500 MHz, $(CD_3)_2SO$)

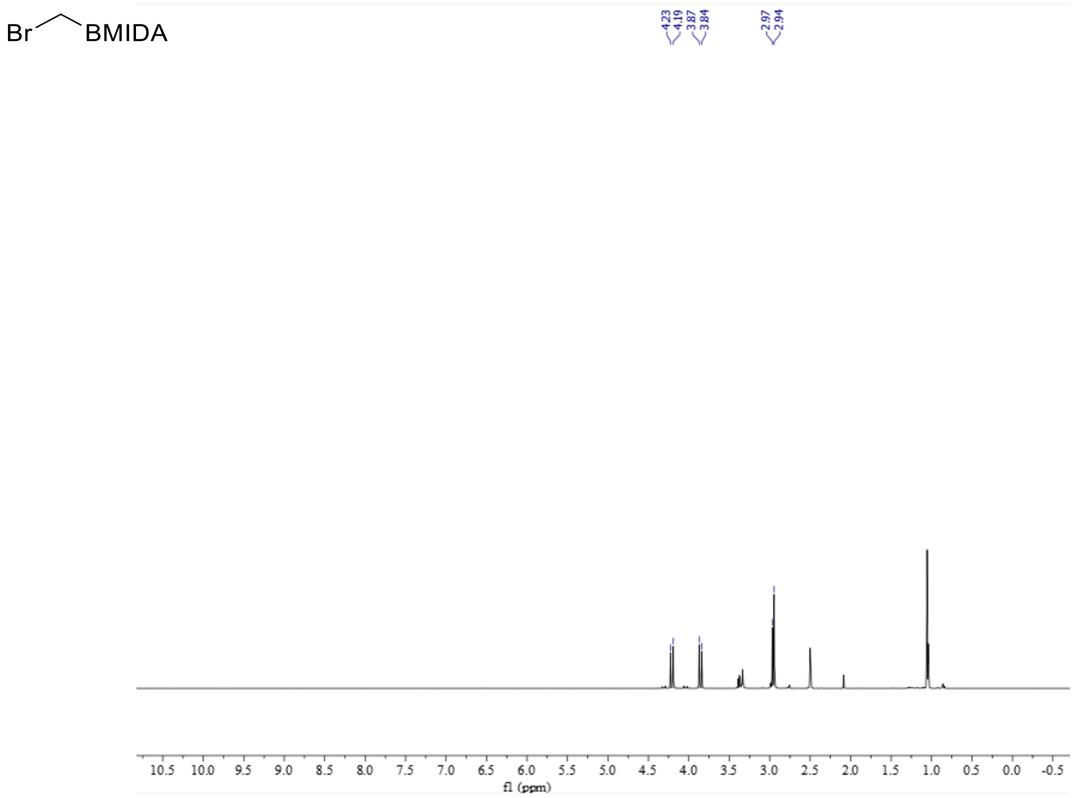

^{13}C NMR (126 MHz, $(CD_3)_2SO$)

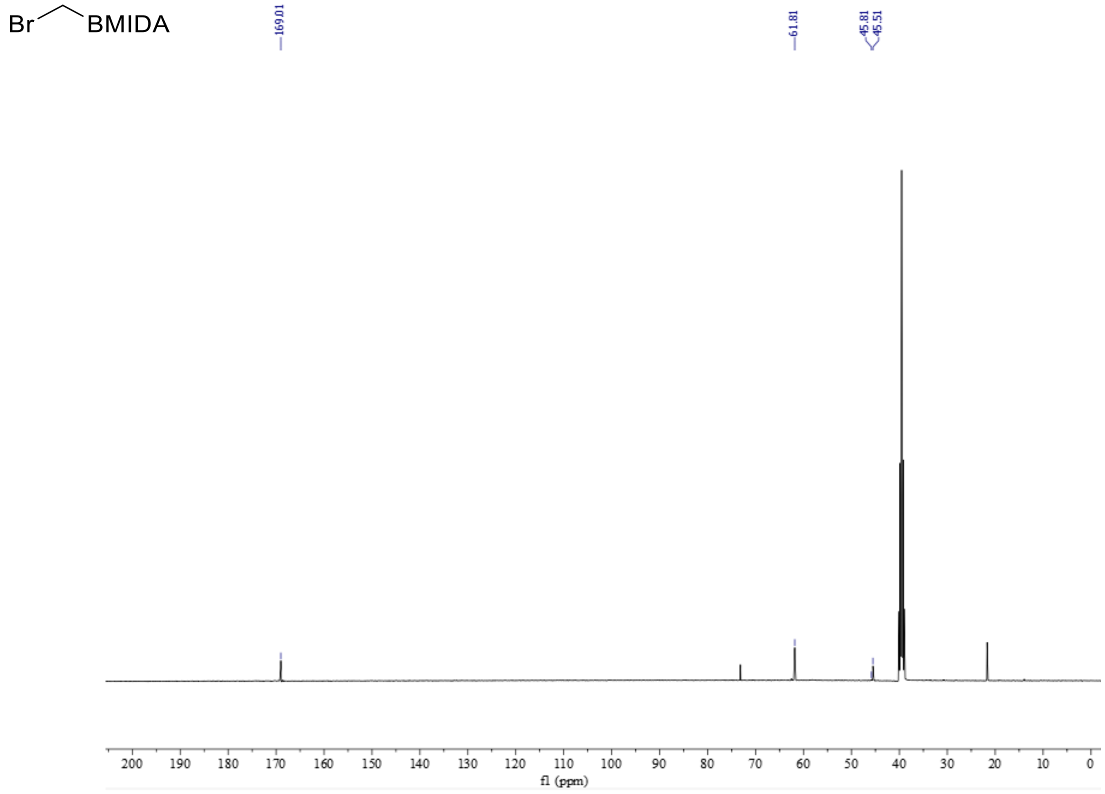

^{11}B NMR (96 MHz, $(CD_3)_2SO$)

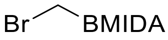

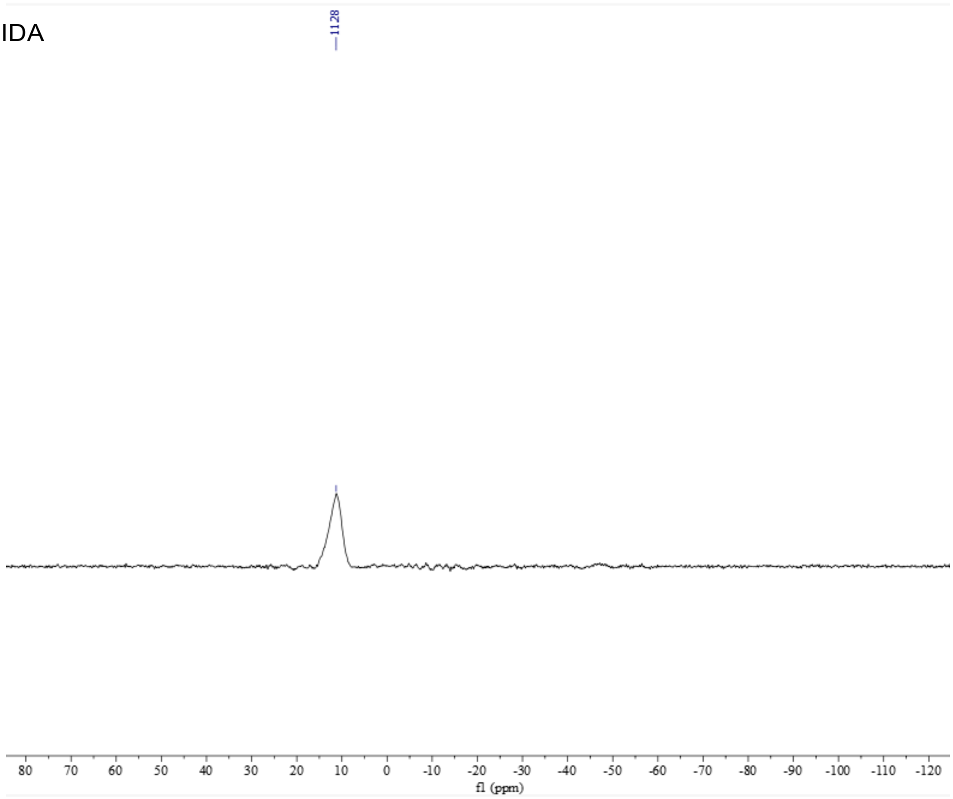

2-(1-chloroethyl)-4,4,5,5-tetramethyl-1,3,2-dioxaborolane (71)

^{1}H NMR (500 MHz, $CDCl_3$)

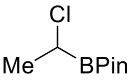

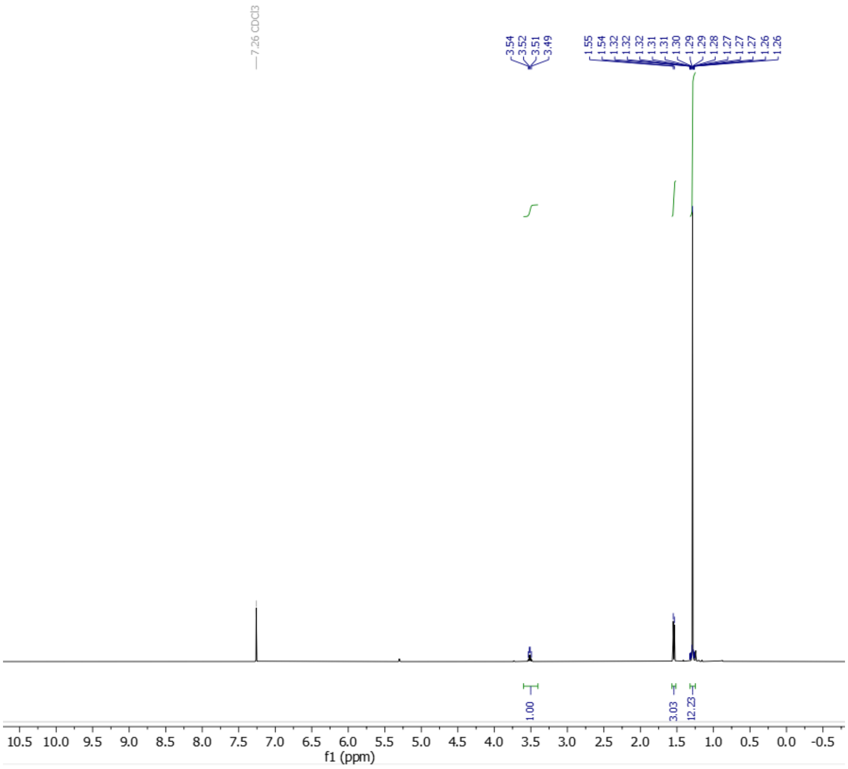

^{13}C NMR (126 MHz, CDCl$_3$)

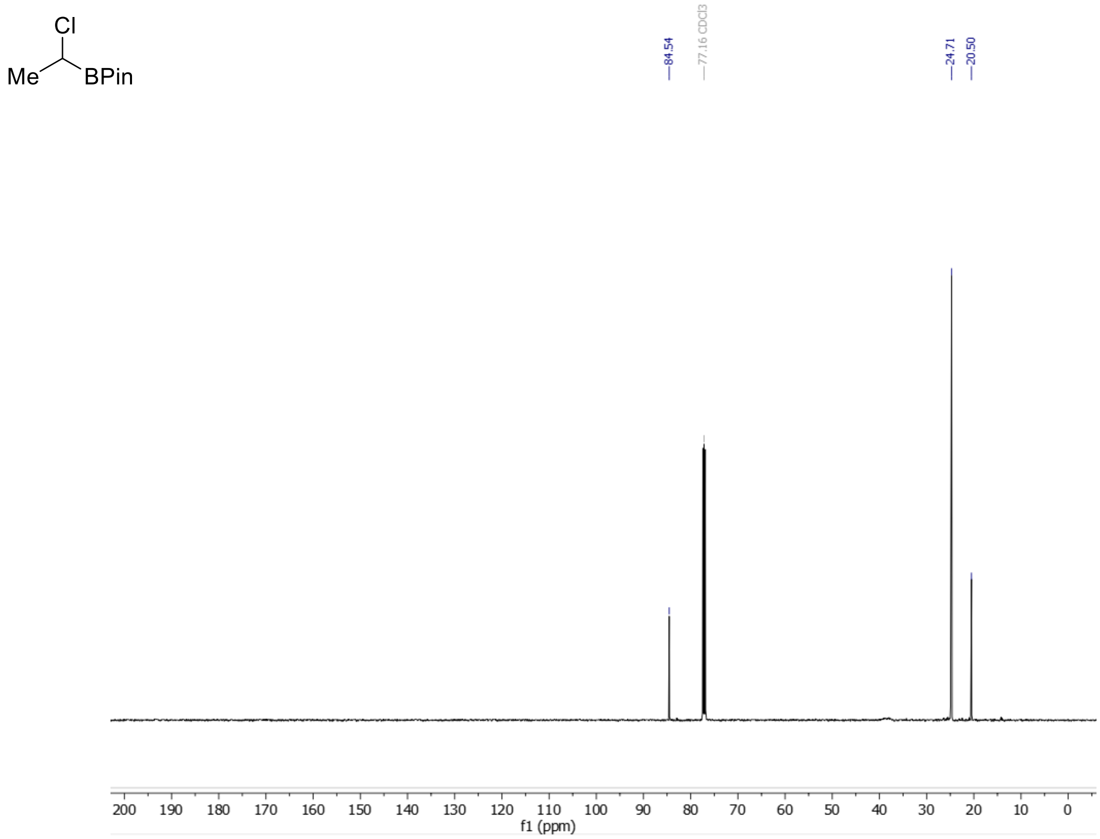

^{11}B NMR (96 MHz, CDCl$_3$)

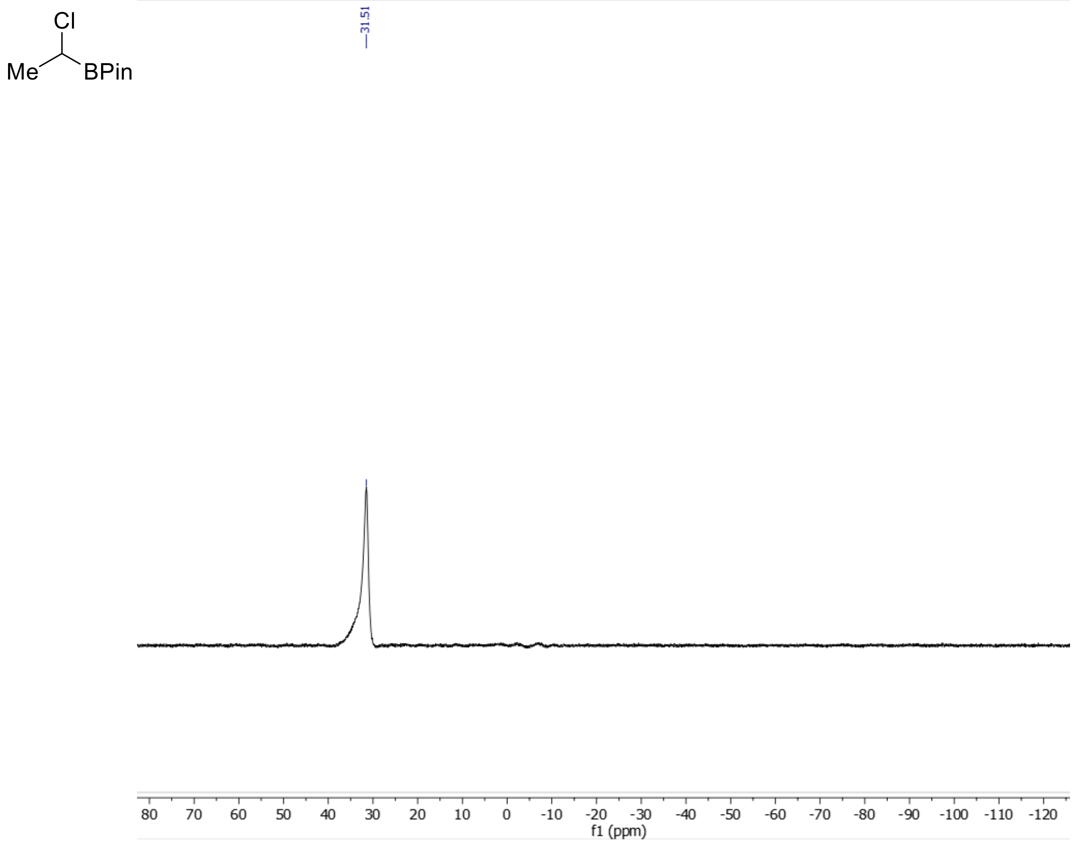

2-(1-bromoethyl)-4,4,5,5-tetramethyl-1,3,2-dioxaborolane (72)

^{1}H NMR (500 MHz, CDCl$_3$)

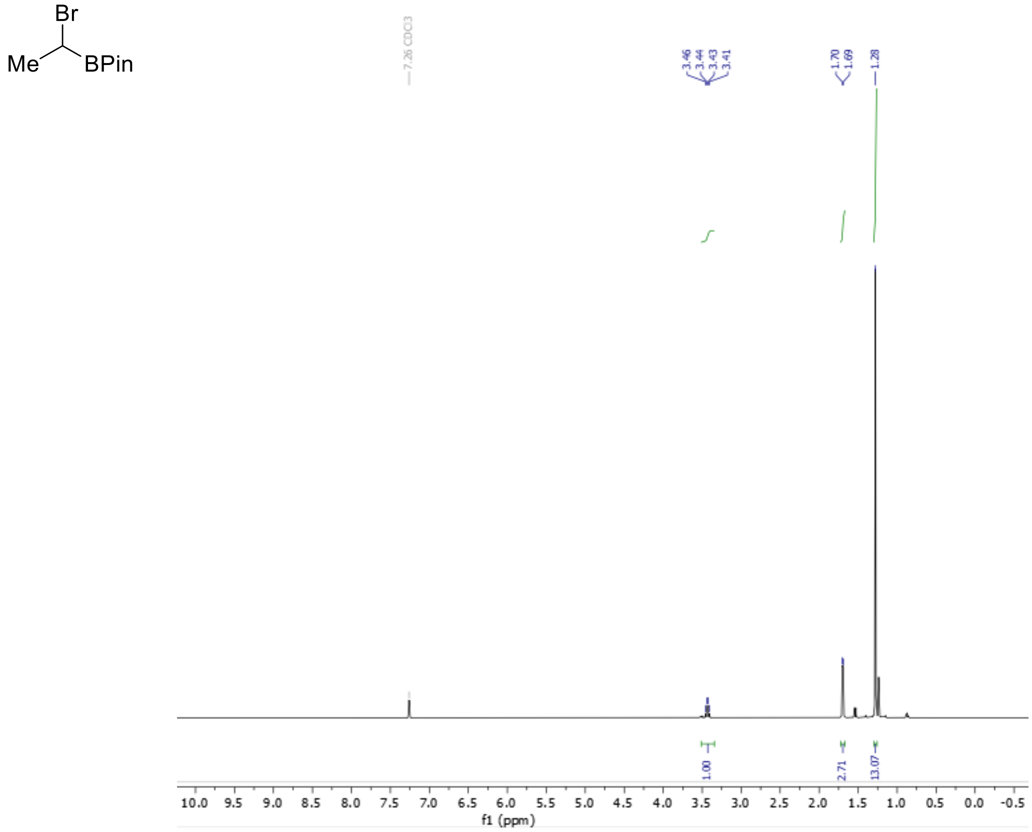

^{13}C NMR (126 MHz, CDCl$_3$)

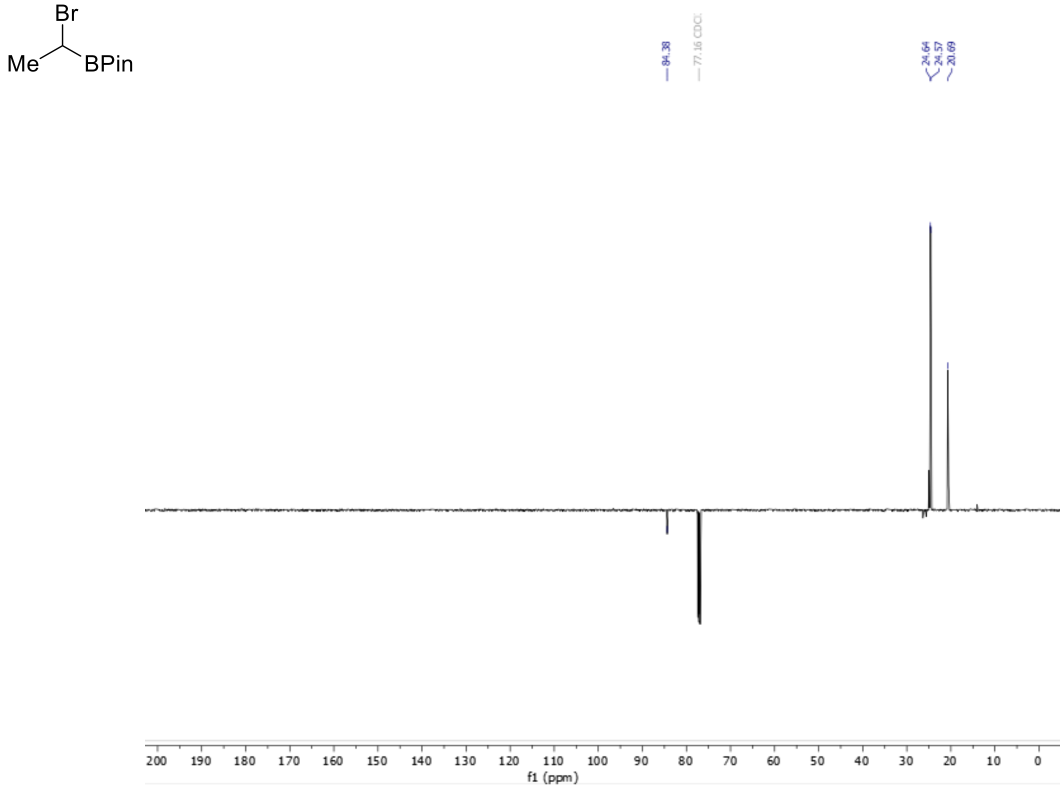

^{11}B NMR (96 MHz, CDCl$_3$)

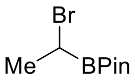

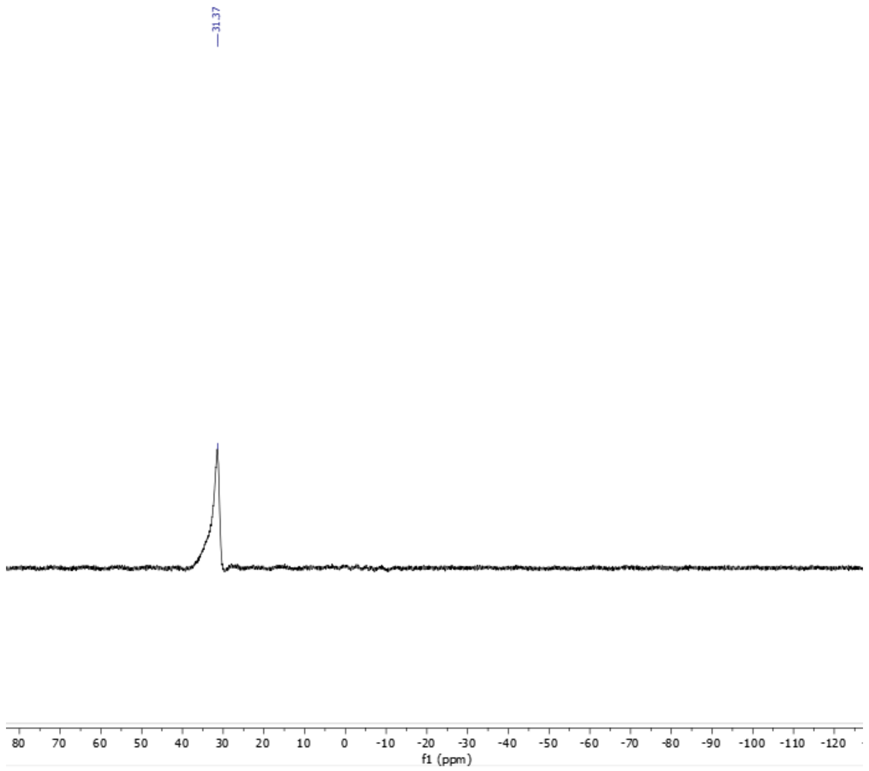

2-(1-iodoethyl)-4,4,5,5-tetramethyl-1,3,2-dioxaborolane (73)

^{1}H NMR (500 MHz, CDCl$_3$)

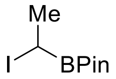

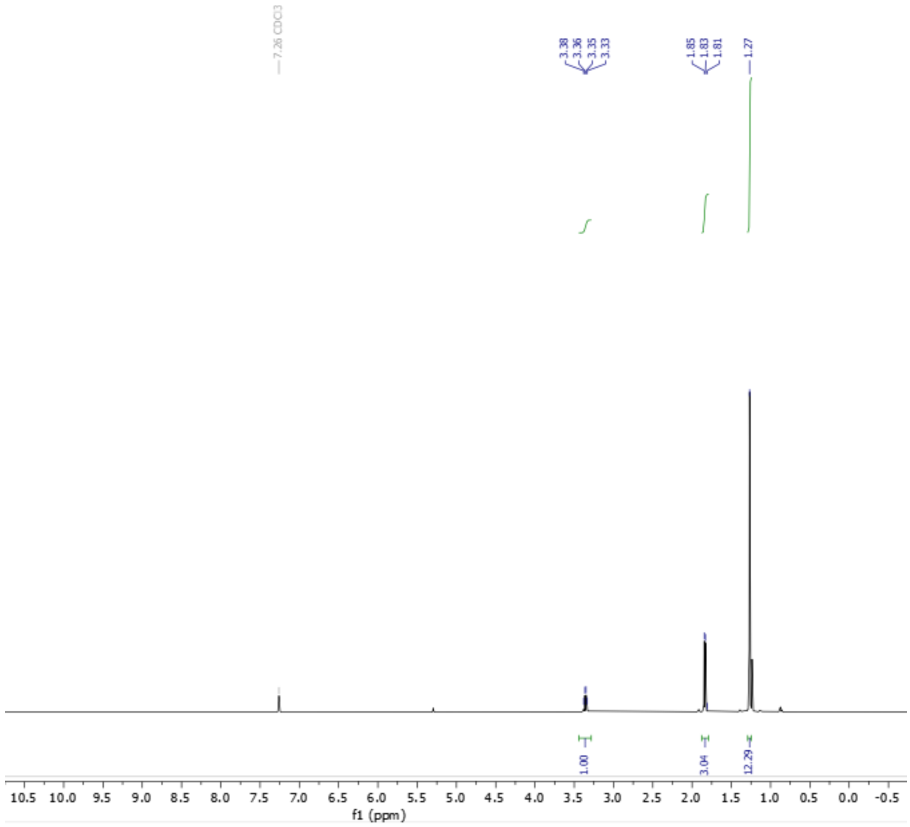

^{13}C NMR (126 MHz, CDCl$_3$)

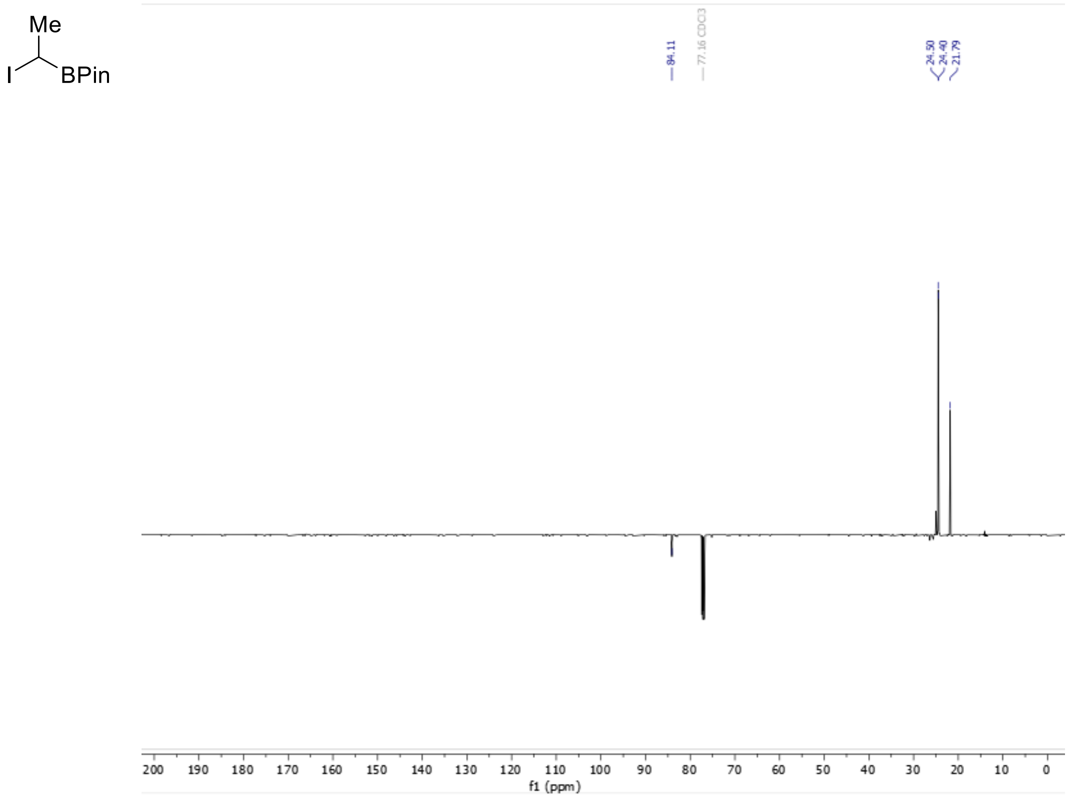

^{11}B NMR (96 MHz, CDCl$_3$)

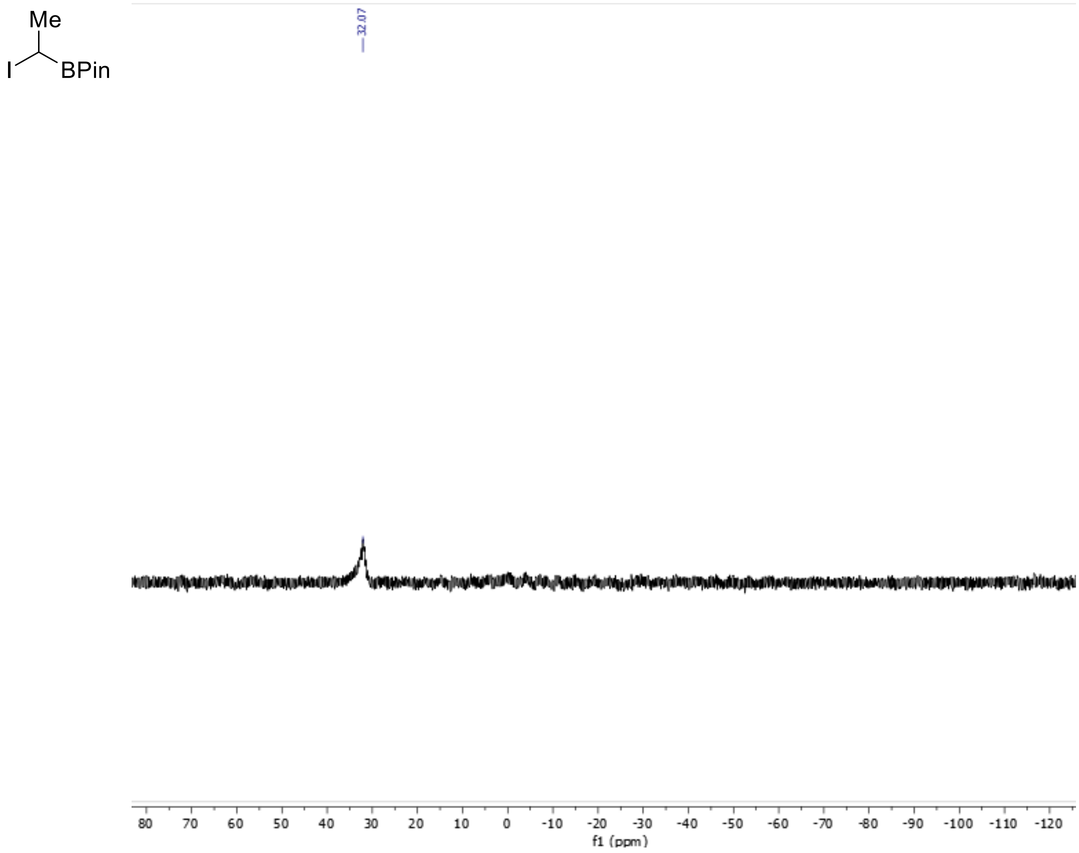

2-(2-bromopropan-2-yl)-4,4,5,5-tetramethyl-1,3,2-dioxaborolane (74)

^{1}H NMR (500 MHz, $CDCl_3$)

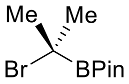

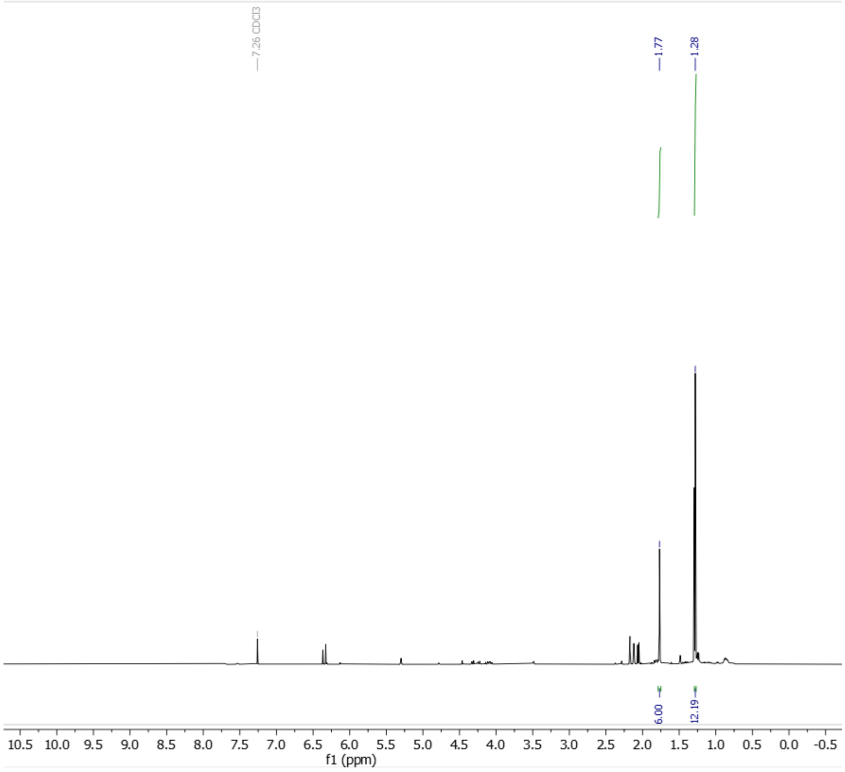

^{13}C NMR (126 MHz, $CDCl_3$)

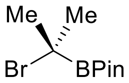

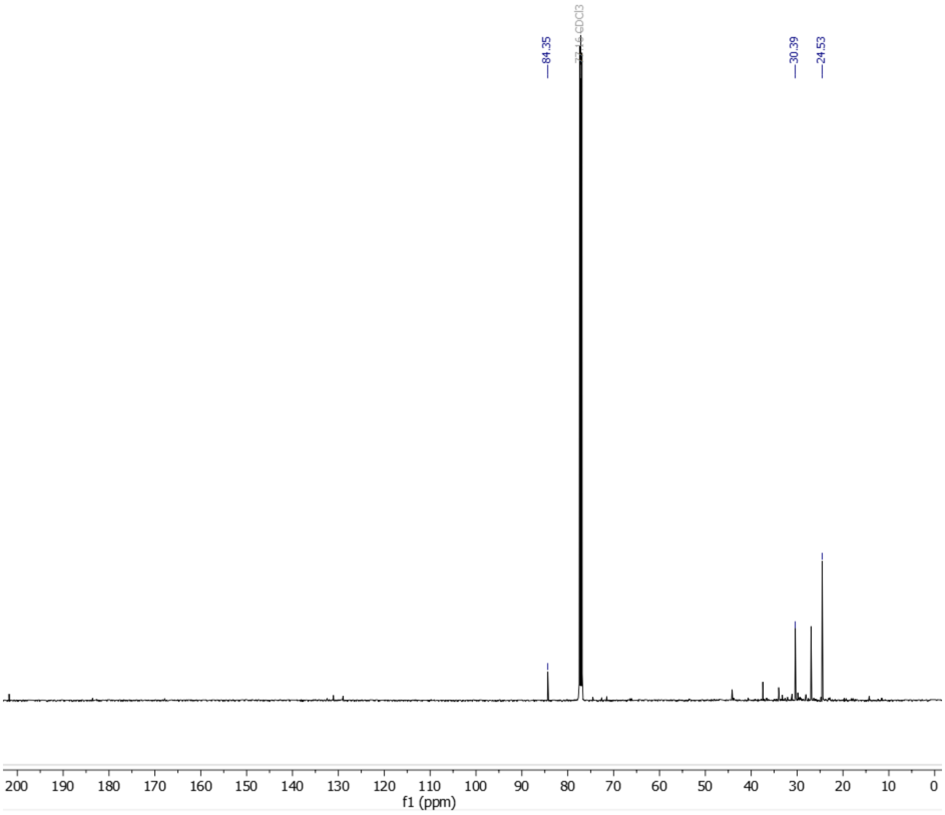

^{11}B NMR (96 MHz, $CDCl_3$)

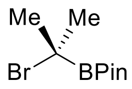

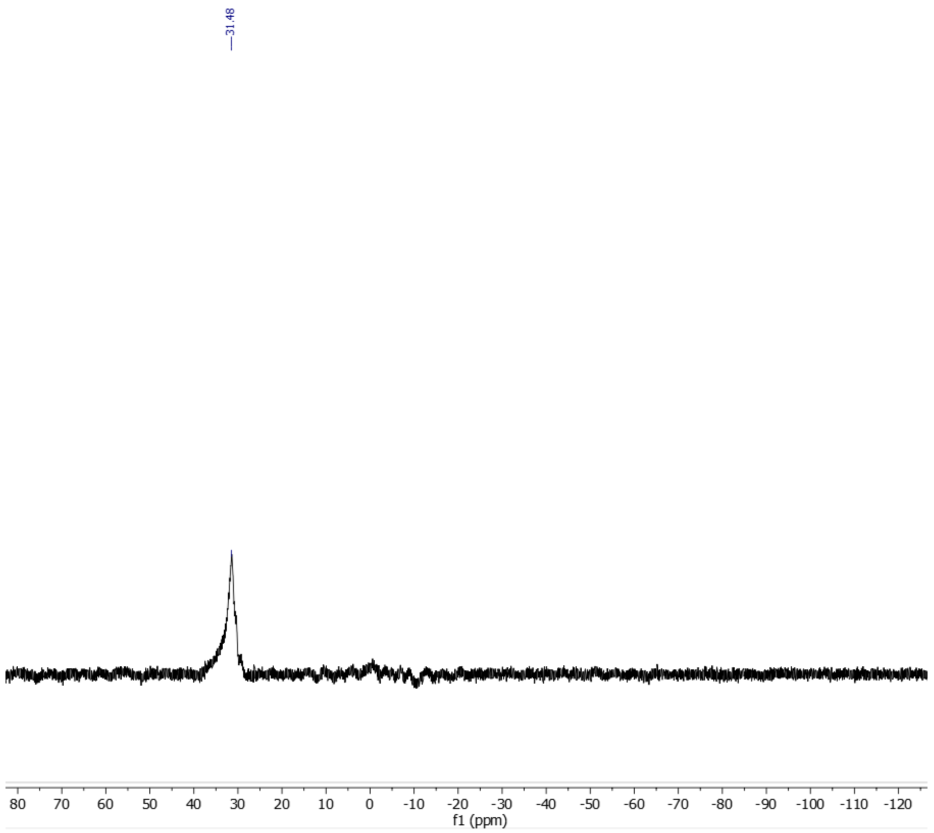

5. Representative sample of unsuccessful substrates

The following substrates were reacted according to General Procedure C using the boronic acid as the limiting reagent. In most cases, the major byproduct was the transesterfied pinacol boronic ester of the boronic acid starting material. Substrates bearing unprotected heteroatoms resulted in extremely complex mixtures which we hypothesized to be caused by facile alkylation by $BrCH_2BPin$.[28]

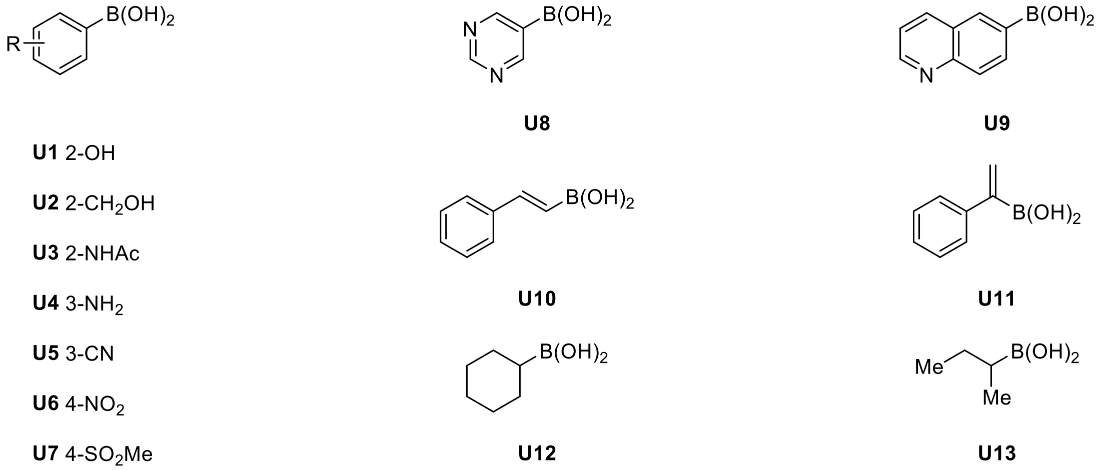

Figure S3: Unsuccessful substrates.

6. Hammett parameter analysis of successful products

Hammett analysis was plotted according to equation 1 for 2-substituted (σ_p) and 4-substituted (σ_m) arylboronic acid substrates:

$$k = \log \frac{\%\ yield\ (R)}{\%\ yield\ (H)} \quad \mathbf{(1)}$$

σ values were obtained from Taft and coworkers.[29]

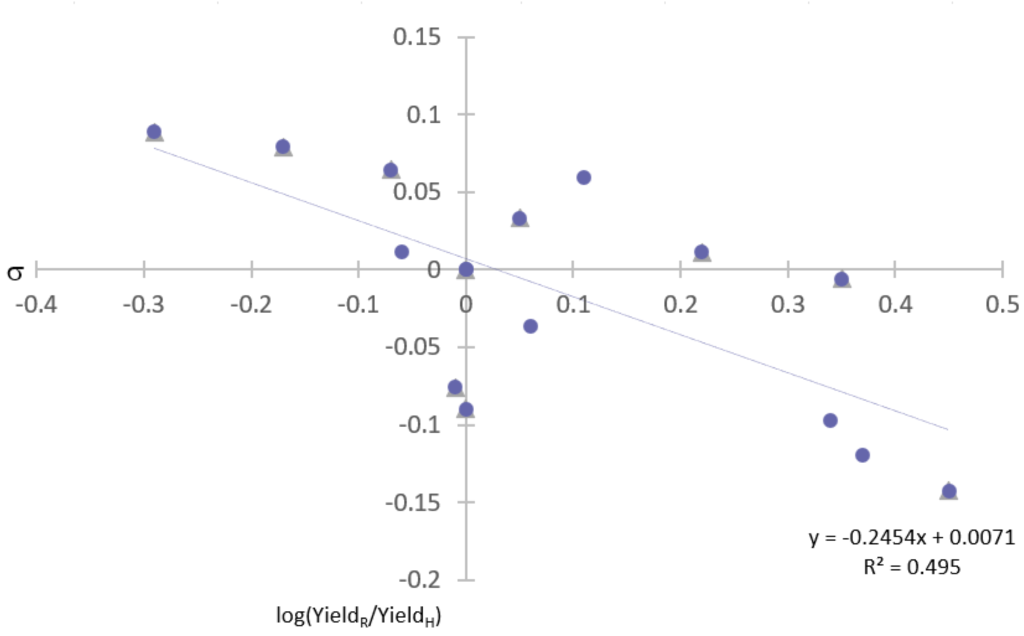

Figure S4: Hammett analysis of successful substrates.

We interpret these above data such that there is minimal electronic influence at the rate limiting step (R^2 <<0.95).

7. Procedures for control studies

7.1 Measuring the electrophilicity of bromomethyl BPin **2-Br** (Scheme 3a)

Followed according to General Procedure C using Compound **1** (27.2 mg, 0.20 mmol, 1.00 equiv) and either bromobenzene, benzyl bromide, bromomethylcyclohexane, or 2-(bromomethyl)-1,3-dioxane as the electrophile. The coupling products with bromobenzene (Compound **58)** and benzyl bromide Compound **59**) were referenced in accordance with the literature.[30-31] Analyses of the crude reaction mixtures showed that bromomethylcyclohexane and 2-(bromomethyl)-1,3-dioxane failed to undergo oxidative addition. In general, the mass balance of the reaction could be tracked by the speciation of pinacol onto Compound **1** to yield Compound **4**.

7.2 Competition study (Scheme 3b)

To an oven-dried microwave vial equipped with a stir bar was added in the following order: dry K_3PO_4 (127 mg, 0.60 mmol, 3.0 equiv), the Compound **1** (27.2 mg, 0.20 mmol, 1.00 equiv), and $Pd(PPh_3)_4$ (3.5 mg, 0.015 mmol, 1.5 mol%), then the vial was capped and purged with Ar. A separate microwave vial containing a stir bar was capped and purged thrice with Ar prior to the addition of DCE (2.0 mL, 0.1 M), then $BrCH_2BPin$ (**1**) (54 mL, 0.30 mmol, 1.5 equiv) and the phenyl (psueodo)halide (PhI, PhBr, PhOTf, or PhCl, 0.30 mmol, 1.5 equiv) and the solution stirred for 10 seconds. The liquid mixture was added to the microwave vial of solids *via* syringe prior to the addition of water (36 μL, 2.0 mmol, 10 equiv) then the reaction mixture was heated to 60 °C and stirred for 24 h. The reaction mixture was cooled to rt and diluted in CH_2Cl_2 (5 mL) then filtered through Celite, eluting with CH_2Cl_2 and concentrated at reduced pressure. Product ratios were determined using TCE as an internal standard, with the CH_3 peak of the sp^2 product (4-methyl-1,1'-biphenyl, Compound **58)** referenced in accordance with the literature.[30]

Selectivity was determined according to equation 2.

$$\text{Selectivity score} = \frac{\%\ \text{NMR yield compound } \mathbf{3}}{\%\ \text{NMR yield compound } \mathbf{58}} \quad (\mathbf{2})$$

7.3 Effect of halomethyl BPin homologation (Scheme 3c)

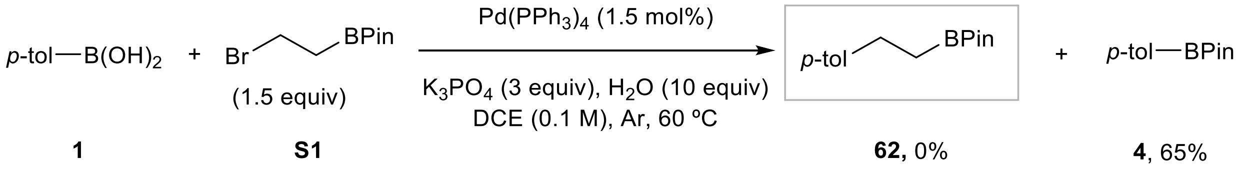

Followed according to General Procedure C using Compound **1** (27.2 mg, 0.20 mmol, 1.00 equiv) and Compound **S1** (74.7 mg, 0.30 mmol, 1.50 equiv) as the electrophile. None of the desired homologation product, Compound **63**, could be detected in the crude NMR. The crude NMR showed speciation byproduct Compound **4** in 65% yield.

7.4 Effect of diol (Scheme 3d)

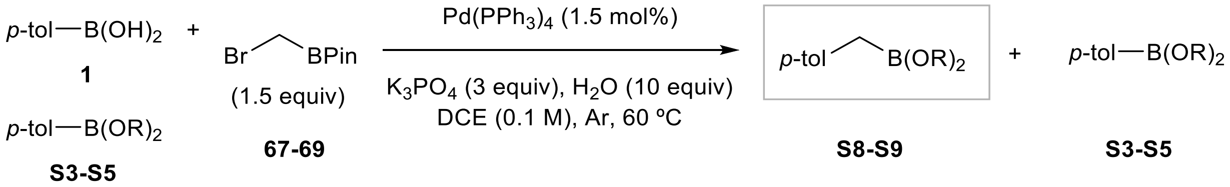

2-(4-methylbenzyl)-1,3,2-dioxaborinane (S7)

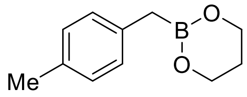

Followed according to General Procedure C using Compound **S3*** (35.2 mg, 0.20 mmol 1.00 equiv) and Compound **67** (53.6 mg, 0.30 mmol, 1.50 equiv). The crude residue (18% ^{1}H NMR yield) was subject to column chromatography on silica gel (1–3% Et_2O in hexane) where the isolation of the desired product failed due to product instability.

*When Compound **1** was used as the substrate (27.2 mg, 0.20 mmol, 1.00 equiv), the reaction afforded an 11% ^{1}H NMR yield. Mass balance was tracked by the formation of speciated ester **S3**.

5,5-dimethyl-2-(4-methylbenzyl)-1,3,2-dioxaborinane (S8)

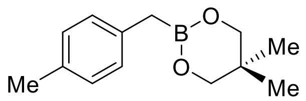

Followed according to General Procedure C using Compond **S5*** (40.8 mg, 0.20 mmol 1.00 equiv) and Compound **68** (60.8 mg, 0.30 mmol, 1.50 equiv). The crude residue (45% ^{1}H NMR yield) was subject to column chromatography on silica gel (0–2% Et_2O in hexane) where isolation of the desired product failed.

*When Compound **1** was used as the substrate (27.2 mg, 0.20 mmol, 1.00 equiv), the reaction afforded an 10% ^{1}H NMR yield. Mass balance was tracked by the formation of speciated ester **S4**.

4,6-dimethyl-2-(4-methylbenzyl)-1,3,2-dioxaborinane (S9)

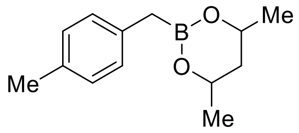

Followed according to General Procedure C using Compound **S4*** (40.8 mg, 0.20 mmol 1.00 equiv) and Compound **69** (62.1 mg, 0.30 mmol, 1.50 equiv). The crude residue (23% ^{1}H NMR yield) was subject to column chromatography on silica gel (0–2% Et_2O in hexane) where isolation of the desired product failed.

*When Compound **1** was used as the substrate (27.2 mg, 0.20 mmol, 1.00 equiv), the reaction failed. Mass balance was tracked by the formation of speciated ester **S5**.

7.5 Linchpin selectivity

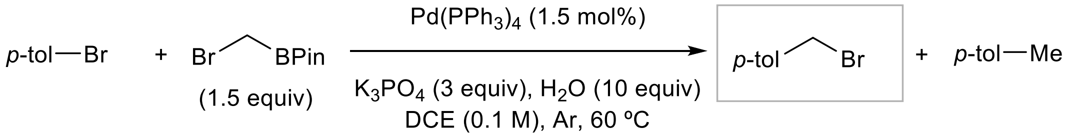

2-Br **S7** **S8**

Scheme S6: Control for the lack of **2-Br** transmetallation.

Followed according to General Procedure C using 4-bromotoluene (34.2 mg, 0.20 mmol, 1.00 equiv) as the substrate. Analysis of the crude reaction mixture showed no formation of compounds **S7** or **S8**.

7.6 Stability of carbenoid surrogate **2-Br**

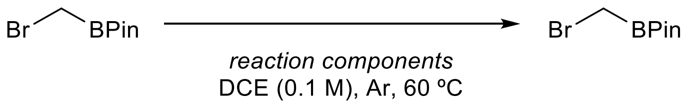

2-Br *recovery*

Entry	Added reaction components	% recovery
1	None	69
2	K_3PO_4	0
3	H_2O	39
4	$K_3PO_4 + H_2O$	0
5	$Pd(PPh_3)_4$	10
6	$K_3PO_4 + H_2O + Pd(PPh_3)_4$	0

Scheme S7: Assessing the stability of **2-Br** to the reaction conditions.

The reactions were setup using the conditions and stoichiometries outlined in General Procedure C in the absence of compound **1**.

7.7 Proposed fate of excess **2-Br**

The above controls suggested that base-mediated hydrolysis of **2-Br** formed the unstable boronic acid which would spontenously protodeboronate. This is supported by our efforts to prepare the parent boronic acid of **2-Br** independently from the BF_3K salt **64**, which resulted in degradation (**Scheme S8**).

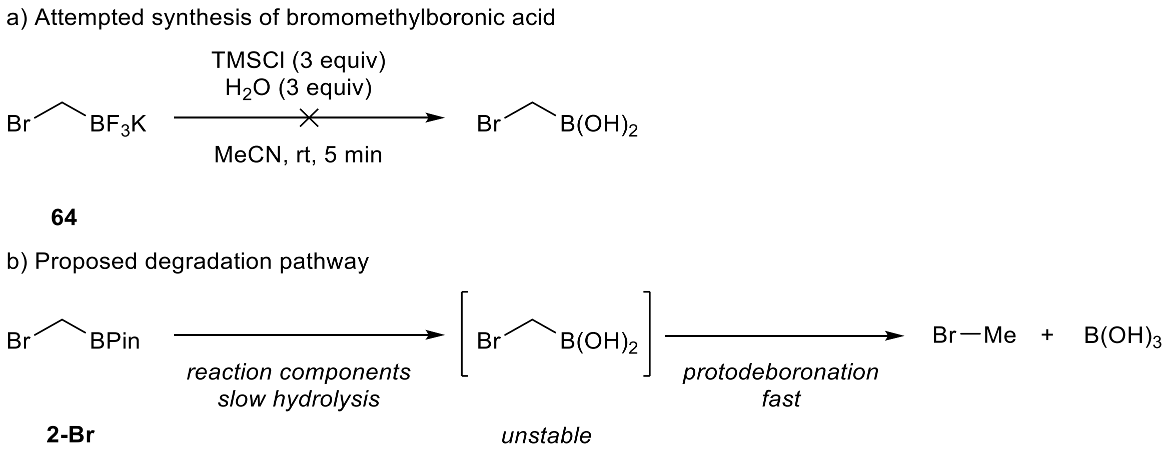

Scheme S8: Proposed degradation of **2-Br** via the unstable boronic acid.

7.8 Product stability

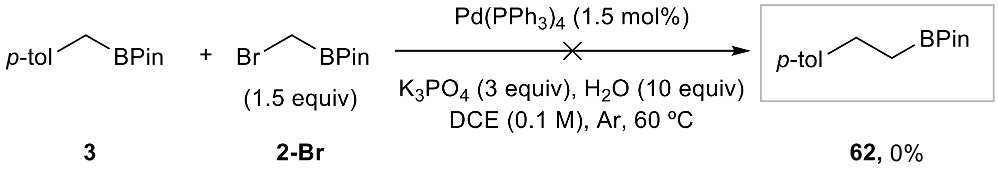

Scheme S9: Control for the lack of product (**3**) transmetallation.

7.9 Secondary methylene homologation trials

Followed according to General Procedure C using Compound **1** (27.2 mg, 0.20 mmol, 1.00 equiv) as the substrate with the below modifications (**Scheme S10**). Analysis of the crude reaction mixtures showed none of the desired product **75** and near quantitative conversion to the byproduct **4**.

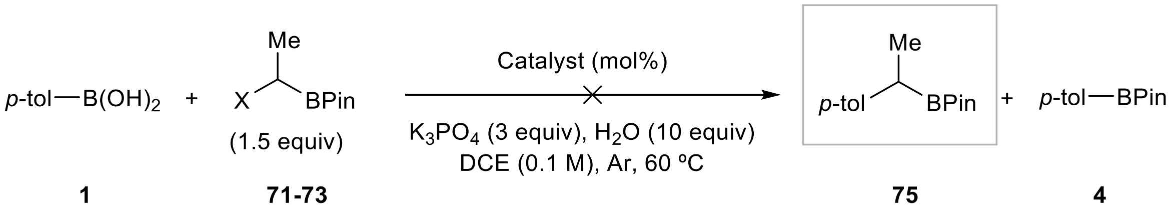

Entry	X (#)	Catalyst	Mol%
1	Cl (**71**)	$Pd(PPh_3)_4$	1.5
2	Br (**72**)	$Pd(PPh_3)_4$	1.5
3	I (**73**)	$Pd(PPh_3)_4$	1.5
4	Br (**72**)	$Pd(PPh_3)_4$	50
5	Br (**72**)	$Pd(OAc)_2$ / SPhos 1:2	1.5
6	Br (**72**)	$Pd(OAc)_2$ / SPhos 1:2	50
7	Br (**72**)	XantPhosPdG3	1.5
8	Br (**72**)	XantPhosPdG3	1.5

Scheme S10: Screen of catalyst/loading for the secondary methyelene homologation.

Conditions similar to those reported by Grevorgyan for the Heck reaction of alkylhalides were also trialed but equally failed (**Scheme S11**).

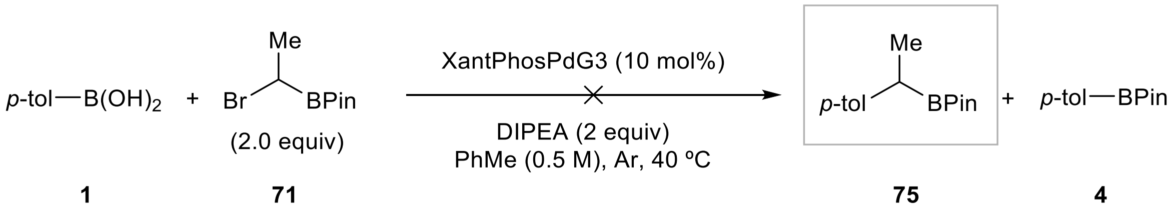

Scheme S11: Grevorgyan-type conditions for the secondary methyelene homologation.

7.10 Monitoring oxidative addition

In an Ar-filled glovebox, $Pd(PPh_3)_4$ (57.8 mg, 0.050 mmol, 1.0 equiv) was weighed and added into a J Young NMR tube followed by THF-d_8 (0.5 mL) then **2-Br** (11.0 mg, 0.050 mmol, 1.0 equiv) or **71** (11.7 mg, 0.050 mmol, 1.0 equiv). The tube was sealed, shaken and removed from the glovebox and monitored by ^{1}H NMR at 298 K.

Methylene donor **2-Br** is rapidly consumed in ~30 min at rt to deliver an oxidative addition complex (**Scheme S12**) whereas secondary methylene donor **71** remains intact with continued monitoring of over 2 h (**Scheme S13**).

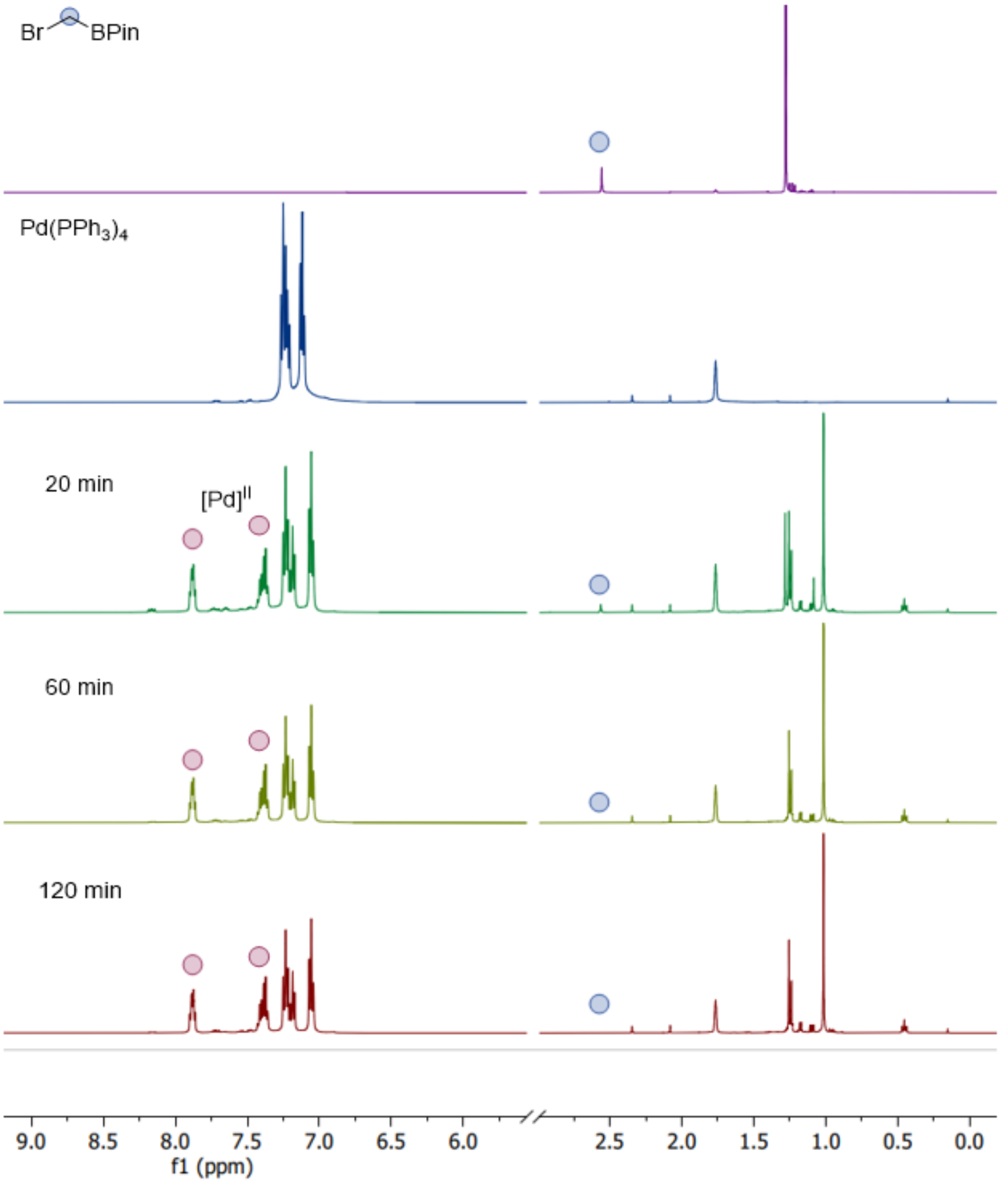

Scheme S12: Monitoring the oxidative addition of **2-Br**.

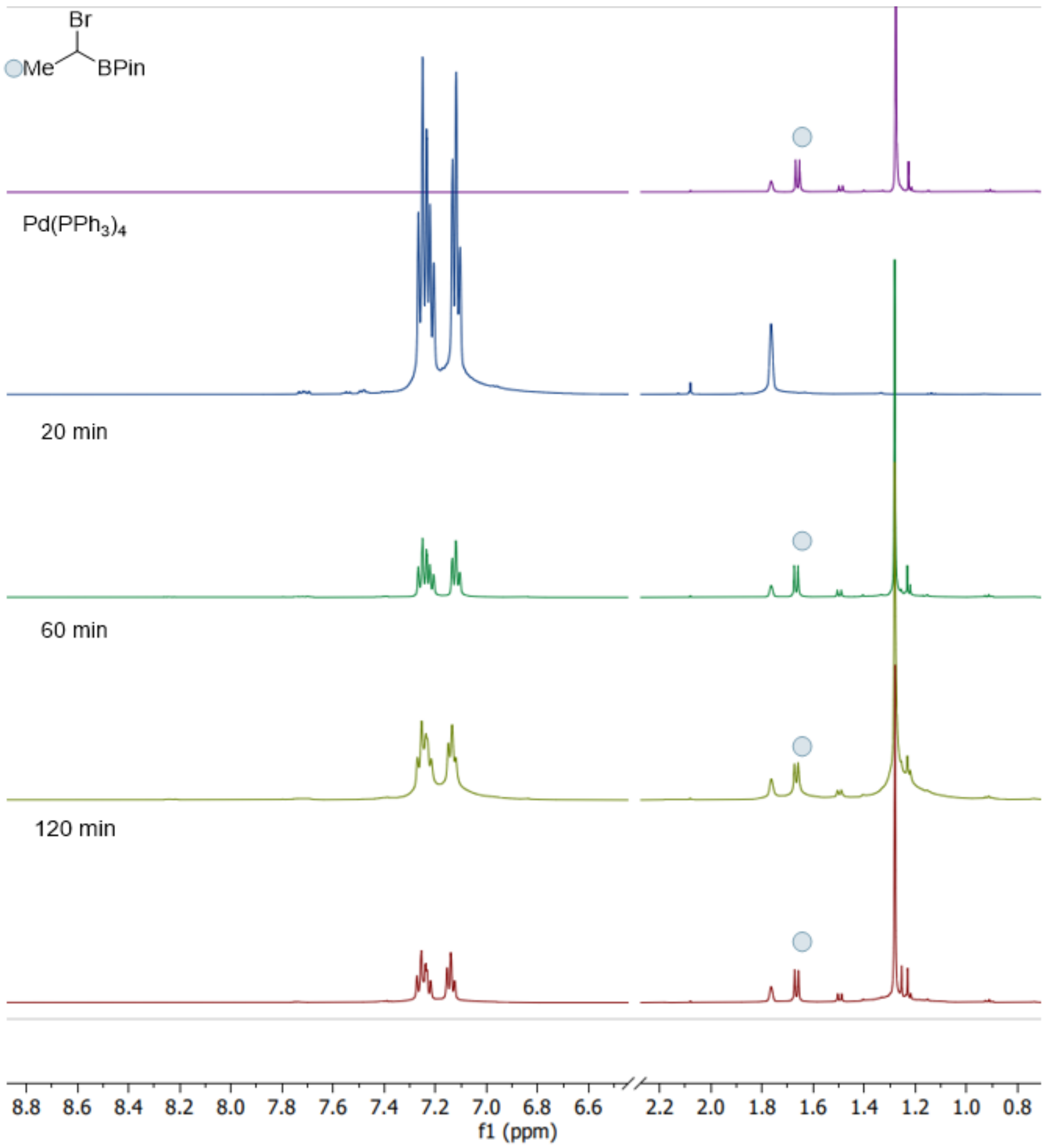

Scheme S13: Monitoring the oxidative addition of **71**.

8. Additive study

To account for the lack of conversion for substrates bearing unprotected heteroatoms, we reintroduced them to the model reaction as additives at either catalytic (5 mol%) or stoichiometric (100 mol%) quantities. In general, catalytic quantities of additives were tolerated quite well, ruling out a catalyst poisoning scenario, whereas stoichiometric quantities shut the reaction down to varying degrees (yield drop 20–90%, **Scheme S14**). This point is further clarified by the comparison of indole versus *N*-methylindole as additives. An exception to this rule was the ligand dba, where catalytic quantities decrease the yield by over 50%. As such, we can only repeat the optimized reaction using a 1.5 mol% $Pd(dba)_2$ / PPh_3 1:2 system instead of $Pd(PPh_3)_4$ when $Pd(dba)_2$ has been recrystallised from $CHCl_3$/water to remove excess dba. The reaction is very robust to chlorobenzene even if it is added to the reaction mixture before **2-Br**, further highlighting the observed chemoselectivity towards oxidative addition.

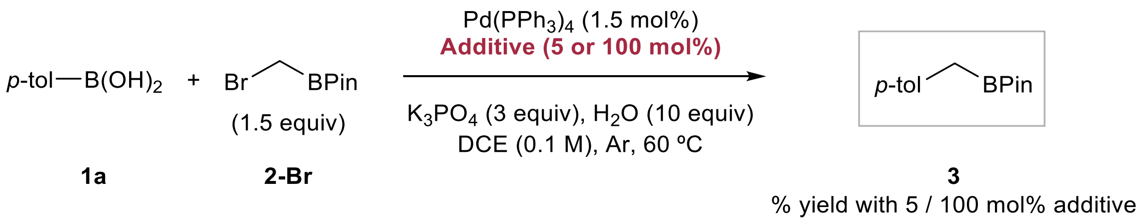

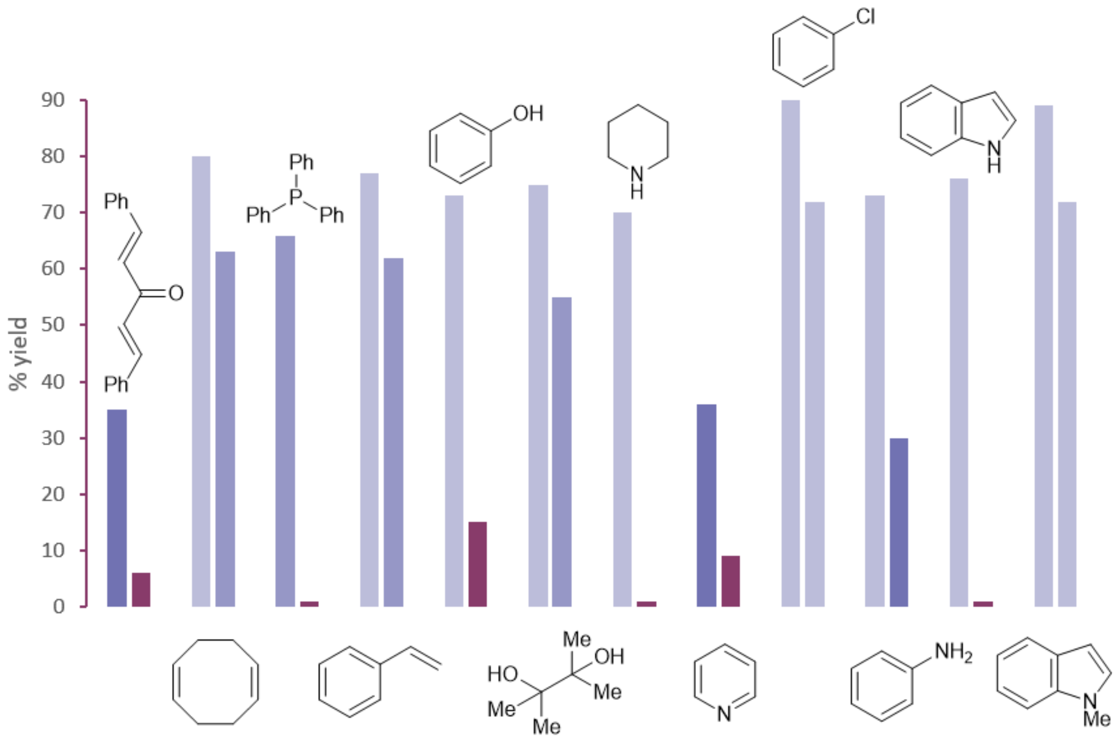

Scheme S14: Additive screen. Yield with no additive = 90%.

9. References

(1) Pulis, A. P.; Aggarwal, V. K. Synthesis of Enantioenriched Tertiary Boronic Esters from Secondary Allylic Carbamates. Application to the Synthesis of C30 Botryococcene. *J. Am. Chem. Soc.* **2012**, *134*, 7570–7574.

(2) Scaggs, W. R.; Snaddon, T. N. Enantioselective α-Allylation of Acyclic Esters Using B(Pin)-Substituted Electrophiles: Independent Regulation of Stereocontrol Elements through Cooperative Pd/Lewis Base Catalysis. *Chem. Eur. J.* **2018**, *24*, 14378–14381.

(3) Bismuto, A.; Cowley, M. J.; Thomas, S. P. *Aluminum-Catalyzed Hydroboration of Alkenes. ACS Catal.* **2018**, *8*, 2001–2005.

(4) Ranjani, G.; Nagarajan, R. Insight into Copper Catalysis: In Situ Formed Nano Cu_2O in Suzuki–Miyaura Cross-Coupling of Aryl/Indolyl Boronates. *Org. Lett.* **2017**, *19*, 3974–3977.

(5) Takahashi, G.; Shirakawa, E.; Tsuchimoto, T.; Kawakami, Y. Alkynes as Activators in the Nickel-Catalysed Addition of Organoboronates to Aldehydes. *Chem. Commun.* **2005**, *11*, 1459–1461.

(6) Xu, L.; Dong, Z.; Zhang, Q.; Deng, N.; Li, S. Y.; Xu, H.-J. Protoboration of Alkynes and Miyaura Borylation Catalyzed by Low Loadings of Palladium. *J. Org. Chem.* **2022**, *87*, 14879–14888.

(7) Kinuta, H.; Tobisu, M.; Chatani, N. Rhodium-Catalyzed Borylation of Aryl 2-Pyridyl Ethers through Cleavage of the Carbon–Oxygen Bond: Borylative Removal of the Directing Group. *J. Am. Chem. Soc.* **2015**, *137*, 1593–1600.

(8) Hume, P. A.; Furkert, D. P.; Brimble, M. A. Total Synthesis of Virgatolide B. *Org. Lett.* **2013**, *15*, 4588–4591.

(9) Dutheuil, G.; Webster, M. P.; Worthington, P. A.; Aggarwal, V. K. Stereocontrolled Synthesis of Carbon Chains Bearing Contiguous Methyl Groups by Iterative Boronic Ester Homologations: Application to the Total Synthesis of (+)-Faranal. *Angew. Chem. Int. Ed.* **2009**, *48*, 6317–6319.

(10) Endo, K.; Ohkubo, T.; Shibata, T. Chemoselective Suzuki Coupling of Diborylmethane for Facile Synthesis of Benzylboronates. *Org. Lett.* **2011**, *13*, 3368–3371.

(11) Zhu, C.; Yamane, M. Transition-Metal-Free Borylation of Aryltriazene Mediated by $BF_3 \cdot OEt_2$. *Org. Lett.* **2012**, *14*, 4560–4563.

(12) Palmer, W. N.; Obligacion, J. V.; Pappas, I.; Chirik, P. J. Cobalt-Catalyzed Benzylic Borylation: Enabling Polyborylation and Functionalization of Remote, Unactivated $C(sp^3)$–H Bonds. *J. Am. Chem. Soc.* **2016**, *138*, 766–769.

(13) Furukawa, T.; Tobisu, M.; Chatani, N. C–H Functionalization at Sterically Congested Positions by the Platinum-Catalyzed Borylation of Arenes. *J. Am. Chem. Soc.* **2015**, *137*, 12211–12214.

(14) Larsen, M. A.; Wilson, C. V.; Hartwig, J. F. Iridium-Catalyzed Borylation of Primary Benzylic C–H Bonds without a Directing Group: Scope, Mechanism, and Origins of Selectivity. *J. Am. Chem. Soc.* **2015**, *137*, 8633–8643.

(15) Pein, W. L.; Wiensch, E. M.; Montgomery, J. Nickel-Catalyzed Ipso-Borylation of Silyloxyarenes via C–O Bond Activation. *Org. Lett.* **2021**, *23*, 4588–4592.

(16) Mao, L.; Szabó, K. J.; Marder, T. B. Synthesis of Benzyl-, Allyl-, and Allenyl-Boronates via Copper-Catalyzed Borylation of Alcohols. *Org. Lett.* **2017**, *19*, 1204–1207.

(17) Atack, T. C.; Lecker, R. M.; Cook, S. P. Iron-Catalyzed Borylation of Alkyl Electrophiles. *J. Am. Chem. Soc.* **2014**, *136*, 9521–9523.

(18) Liu, L.; Wang, G.; Jiao, J.; Li, P. Sulfur-Directed Ligand-Free C–H Borylation by Iridium Catalysis. *Org. Lett.* **2017**, *19*, 6132–6135.

(19) Li, J.; Huang, C.-Y.; Ataya, M.; Khaliullin, R. Z.; Li, C. J. Direct Deoxygenative Borylation of Carboxylic Acids. *Nat. Commun.* **2021**, *12*, 4970.

(20) Wang, H.; Li, L.; Bai, X. F.; Shang, J.-Y.; Yang, K. F.; Xu, L. W. Efficient Palladium-Catalyzed C–O Hydrogenolysis of Benzylic Alcohols and Aromatic Ketones with Polymethylhydrosiloxane. *Adv. Synth. Catal.* **2013**, *355*, 341–347.

(21) Fujii, I.; Semba, K.; Nakao, Y. The Kumada–Tamao–Corriu Coupling Reaction Catalyzed by Rhodium–Aluminum Bimetallic Complexes. *Org. Lett.* **2022**, *24*, 3075–3079.

(22) Wang, P.; Chen, X.; Nie, X.; Wu, Y. Preparation Method of Para-Substituted Aryl Compound. CN111187130A, May 22, 2020.

(23) Borys, A. M.; Gil-Negrete, J. M.; Hevia, E. Atom-Efficient Transition-Metal-Free Arylation of N,O-Acetals Using Diarylzinc Reagents through Zn/Zn Cooperativity. *Chem. Commun.* **2021**, *57*, 8905–8908.

(24) Xiang, M.; Xin, Z. K.; Chen, B.; Tung, C. H.; Wu, L.-Z. Exploring the Reducing Ability of Organic Dye (Acr+-Mes) for Fluorination and Oxidation of Benzylic C(sp^3)–H Bonds under Visible Light Irradiation. *Org. Lett.* **2017**, *19*, 3009–3012.

(25) Qiao, J.-B.; Zhao, Z. Z.; Zhang, Y. Q.; Yin, K.; Tian, Z.-X.; Shu, X.-Z. Allylboronates from Vinyl Triflates and α-Chloroboronates by Reductive Nickel Catalysis. *Org. Lett.* **2020**, *22*, 5085–5089.

(26) Potter, B.; Edelstein, E. K.; Morken, J. P. Modular, Catalytic Enantioselective Construction of Quaternary Carbon Stereocenters by Sequential Cross-Coupling Reactions. *Org. Lett.* **2016**, *18*, 3286–3289.

(27) Liu, Q.; Hong, J.; Sun, B.; Bai, G.; Li, F.; Liu, G.; Yang, Y.; Mo, F. Transition-Metal-Free Borylation of Alkyl Iodides via a Radical Mechanism. *Org. Lett.* **2019**, *21*, 6597–6602.

(28) Mollner, T. A.; Isenegger, P. G.; Josephson, B.; Buchanan, C.; Lercher, L.; Oehlrich, D.; Hansen, D. F.; Mohammed, S.; Baldwin, A. J.; Gouverneur, V.; Davis, B. G. Post-Translational

Insertion of Boron in Proteins to Probe and Modulate Function. *Nat. Chem. Biol.* **2021**, *17*, 1245–1261.

(29) Hansch, Corwin.; Leo, A.; Taft, R. W. A Survey of Hammett Substituent Constants and Resonance and Field Parameters. *Chem. Rev.* **1991**, *91*, 165–195.

(30) Mu, B.; Li, J.; Han, Z.; Wu, Y. Fast Suzuki–Miyaura Cross-Coupling Reaction Catalyzed by the $Na_2Pd_2Cl_6$ Complex with Ethyl Calix[4]Aryl Acetate at Room Temperature in Aqueous Medium under Ligand-Free and Ambient Atmosphere. *J. Organomet. Chem.* **2012**, *700*, 117–124.

(31) Zhao, F.; Tan, Q.; Xiao, F.; Zhang, S.; Deng, G.-J. Palladium-Catalyzed Desulfitative Cross-Coupling Reaction of Sodium Sulfinates with Benzyl Chlorides. *Org. Lett.* **2013**, *15*, 1520–1523.
